# Supplementary material for: Sedimentary ancient DNA as part of a multimethod paleoparasitology approach reveals temporal trends in human parasitic burden in the Roman period
Source: PLoS Negl Trop Dis. 2025 Jun 10;19(6):e0013135. doi: 10.1371/journal.pntd.0013135 (PMC12151383; doi:10.1371/journal.pntd.0013135)
Supplement: S1 Appendix — (DOCX) [file pntd.0013135.s001.docx]

**S1 Appendix**

Ledger, M.L. et al. Sedimentary ancient DNA as part of a multimethod paleoparasitology approach reveals temporal trends in human parasitic burden in the Roman period. PLOS Neglected Tropical Diseases

Table of Contents:

A. Site Information and Sample Details 1

B. Sedimentary DNA Methods for Paleoparasitology 8

C. Parasite Bait Design 15

D. Results 16

E. Coprolite Identification 18

F. Review of Paleoparasitological Literature 25

G. References 39

# Site Information and Sample Details

*Çatalhöyük, Türkiye (6410–6150 BCE)*

Çatalhöyük is an early Ceramic Neolithic site in Anatolia. It is one of the earliest farming communities in Western Asia. From an infectious disease standpoint, the site is one of the best-preserved large settlement agglomerations in prehistoric Western Asia. The population density at this site was relatively high compared to other Neolithic sites at the time, which is likely to impact infection transmission. The settlement is comprised of densely clustered mudbrick houses, interspersed with external spaces used for middens, animal penning and other daily activities [1,2]. Many of these external spaces contained animal and human feces [3]. The sample included in this study was a coprolite (mineralized feces) recovered from one of these middens. It was collected during excavation of Space 329, Level South P, and radiocarbon-dated to 6410–6150 BCE. Fecal biomarker analysis indicated a human origin for this coprolite [4].

*Tell Zeidan, Syria (5800–4000 BCE)*

Tell Zeidan was a prehistoric settlement in northern Syria. The settlement was located in the Euphrates River Valley. Human remains were excavated from the site and radiocarbon dated to 6500–6000 BP [5]. Activity at the settlement spans the Halaf, Ubaid, and Late Chalcolithic periods. The site is a triple mound, oriented northwest to southeast covering 12.5 hectares. Human burials were excavated across the site between 2008–2010 [5]. These included males and females as well as juveniles and adults, though juvenile burials outnumbered adult burials. During excavation, soil was collected from the pelvis of these individuals for parasitological analysis. Pelvic soil from one individual was included in this analysis. The pelvic soil from this individual contained a *Schistosoma* sp. egg [6].

*Durrington Walls, Britain (2535–2475 BCE)*

The Neolithic settlement of Durrington Walls is located 2.8 km from Stonehenge. It is a 17-hectare henge with a settlement that pre-dates the henge by two decades [7,8]. The settlement is thought to have been occupied by builders of Stonehenge as its occupation coincides with construction of the sarsen circle and trilithons at Stonehenge [9]. Excavations of the east entrance of the site revealed numerous middens and pits in the vicinity of house floors. Analysis of ceramics, animal bone, and plant remains recovered from pits revealed evidence for feasting at the site [8]. These pits also contained coprolites. Fecal biomarkers revealed that these coprolites originated from both dogs and humans [10]. One coprolite, of likely human origin, was included in this study. Microscopic analysis of this coprolite previously revealed Capillariid eggs [10].

*Must Farm, Britain (c. 850 cal. BCE)*

Must Farm was a Late Bronze Age pile-dwelling settlement located in the Fens of East Anglia in modern-day Britain [11]. During the Bronze Age this was a low-lying wetland area which contained numerous bodies of freshwater and brackish water interspersed by pockets of dry land, with periodic fluctuations in water levels [12]. The settlement at Must Farm consisted of a series of five stilted timber structures surrounded by a palisade, built over this freshwater system. Analysis of invertebrate and plant remains at the site shows that the water beneath the settlement flowed at an extremely slow rate and contained dense aquatic to helophytic plants. Radiocarbon dates place the period of occupation of the settlement during the 9th century BCE, which is the terminal Bronze Age in southern Britain [13]. Dendrochronological evidence suggests that the settlement was constructed and inhabited for around one year, before a large fire tore through the settlement causing the structures to collapse into the channel below where they were subsequently covered in alluvial silts.

For this analysis we included sediment samples from the construction/occupation layer from the center of Structure 3 as well as sediment from upstream of the structures. Waterlogged coprolites were also collected from around the structures. Fecal biomarker analyses were conducted on the coprolites to determine if they were of human or animal origin [14]. This revealed a mix of human and canine coprolites as well as coprolites of unknown origin. We included two human coprolites and one dog coprolite in this study as suggested by lipid biomarker analysis. However, see section D for a discussion of aDNA and potential coprolite origins.

*Arlon, Belgium (250–280 CE)*

Arlon, or *Orolauno* as the site was called in the Roman period, was a Roman *vicus* in modern-day Belgium. It flourished between the 1st–3rd c. CE, and is thought to have had an economy reliant on various crafts as indicated by the many workshops that have been excavated from the site. These workshops include those for iron metallurgy, wood turning, leather processing, and glass making [15]. Around the 4th c. CE a military fortress was built on the vicus covering an area of about 5 hectares [16].

The sample included in this study came from the fill of a Roman period latrine (F05) at the site that dates between 250–280 CE. The area of the site where the latrine was found was excavated in 2003–2006 by the archeological heritage agency of the Walloon region. The latrine was found while excavating an artisanal and residential quarter of the *vicus*.

*Vagnari, Italy (1^st^–4^th^ c. CE)*

Vagnari was a Roman Imperial estate located in southern Italy near the modern-day city of Gravina in Puglia. Excavations of the site are focused on the *vicus* (village) area and associated cemetery. The Imperial Estate, established in the 1^st^ c. CE, was owned by the Roman Emperor and individuals working and living at the estate would have been enslaved or employed by the Emperor [17]. Excavations at the *vicus* at Vagnari have shed light on the major industries and production occurring at the site. There is evidence for tile production, a winery, agricultural activity, and metallurgy. It is thus expected that the *vicus* was inhabited by a range of craftsmen including millers, potters, carpenters, smiths, masons, and agricultural workers [18].

During excavation of the site, stone lined drains were found running beneath some of the rooms. Sediment was collected from these drains during excavation. Sediment from two of these drains was included in this study. These samples came from drain 5045, context 5046 and 5050.

*Villa at Vacone, Italy (2^nd^ c. BCE – 3^rd^ c. CE* *and 7^th^–8^th^ c. CE)*

The Roman villa at Vacone, Italy is located 55 km north of Rome. The villa was constructed in the 2^nd^ c. BCE, with a later period of extensive construction and renovation in the early Imperial period [19]. The villa was then abandoned in the 3^rd^ c. CE after damage likely sustained from an earthquake. This a rural Roman villa that consisted of habitation areas including a bath complex, storage and production areas [20]. There is evidence for olive oil production and later wine production at the villa [20]. Thus, there would have been agricultural work to grow necessary olives and grapes to support these industries. The site was later used again during the early medieval period, as evidenced by a number of human burials excavated from the villa that have been radiocarbon dated to the 7^th^–8^th^ c. CE.

Samples included in this study came from the Roman Imperial period villa as well as the later burials. From the Imperial period villa, one sample came from the subfloor of a potential latrine (Room 33, 1142) and three samples were collected from a *cappuccina* drain (drain 931). Samples from the drain were collected from different areas along the length of this drain. Finally, two pelvic soil samples from early medieval burials were included. One of these individuals was a male who died at the age of 40–50 years (Vac 1 Grave 5) and the other was 3–4 years at the time of death (Vac 1 Grave 1).

*Viminacium, Serbia (2^nd^–3^rd^ c. CE)*

Viminacium was a prominent site in the frontier regions of the Roman Empire. It is located in modern-day Serbia. In the Roman period it was the capital of the province of *Moesia Superior* and was a large legionary fortress. The site was established in the 1^st^ c. CE, first as a military camp with permanent stationing of a Roman legion, it later grew into a large city. The population of the city was estimated to be around 400,000 people at its peak [21]. It served as a base for other Roman forts in the region and had a number of workshops and likely supplied goods to some of these other forts [22]. During excavations at the site in 2004 a communal latrine was identified in the bath complex. This latrine had a drain through which water would flow from the baths and wash away waste deposited into the latrine. Coprolites were identified on the floor of the latrine and one was collected for analysis, a subsample of which was included in this study.

*Sardis, Türkiye (6^th^–7^th^ c. CE)*

Sardis was a prominent city within the Roman province of *Asia*, modern-day Türkiye. It was a Lydian city that later became part of the Roman Empire. The city expanded during the Roman Imperial period, and by the Late Roman period it has been estimated it had 50,000–100,000 inhabitants [23,24]. It boasted numerous monuments including the Temple of Artemis, a gymnasium, baths, and later its famed synagogue. As with other sites in the eastern Mediterranean, Sardis began to decline in the 7^th^ c. CE, with the abandonment of public spaces and the subdivision and repurposing of houses. At Sardis, this occurred around the time of an earthquake and fire in the early 7^th^ century [24].

Samples included in this study came from two drains in the city. Both sediment samples came from a city drain (Trench RT 17.1, basket 66 bottom and basket 67). This drain ran below the city center and beneath the synagogue, though it did not connect to the synagogue. These samples were collected from two different branches of the drain that later fed into one another. The drain was used between the 6^th^–7^th^ c. CE.

*Sagalassos, Türkiye (2^nd^–5^th^ c. CE)*

Sagalassos, also located in Anatolia, originated as a hilltop settlement by the end of the 5th c. BCE and was incorporated into the Roman Empire in 25 BCE after which it saw important social and economic developments. It was a major production site for pottery, supplying the surrounding regions and occasionally as far away as Egypt [25]. During the Imperial period many typical urban monuments and buildings were constructed, including an extensive Bath-Gymnasium [26]. The latter, built between the late 1^st^–2^nd^ c. CE, included a number of vaulted rooms that structurally supported parts of the complex. One of these was identified as a public latrine. This room contained channeled stones, holes for attaching seats and associated sewers attesting to its use as a latrine, open to the general public. Water from the baths was directed through the latrine to wash human excrement into the sewer system [27].

One sediment sample collected from the sewage channel in this room was included in this study. This sample has also been studied using fecal biomarkers which suggested this sample and others from the sewage channel contained human fecal material [27].

*Ephesus, Türkiye (3^rd^–6^th^ c. CE)*

Ephesus is a well-known city within the Roman province of *Asia*, modern-day *Türkiye*. Ephesus has a long history of occupation beginning in the 7^th^ millennium BCE. The Hellenistic city became part of the Roman Republic in 133 BCE and was later declared the capital of the province of *Asia*, after which the city grew extensively through the Imperial period [28,29]. Historical records give further evidence for many high-status visitors who came to Ephesus for political reasons. As the seat of the proconsul in *Asia* and a center for early Christian communities, it was a center for commerce, politics, and scholarship, particularly in medicine.

Two sediment samples from Ephesus were included in this study. The first was sediment from the drain of a public latrine associated with the adjacent Varus bath complex, also called the Baths of Scholastica [30]. This sample dates to the 6^th^ century CE. The second sample was from a private latrine (Room No. 34/34a) at one of the Terrace houses (Terrace House 2, unit 7), dated from the Augustan period to the 3^rd^ century CE [31,32]. Mineralized material adherent to the sides of the sewer connected to the latrine was scraped off and used for analysis.

*Mértola, Portugal (12^th^–13^th^ c. CE)*

A cesspit was uncovered during excavations at Encosta do Castelo in Mértola, Portugal. The cesspit is dated to the 12^th^ to early 13^th^ c. CE. The city itself was an important Medieval port city located on the Guadiana River. The city was a market town with trade connections into the Middle East and was an important manufacturer of ceramics in the region [33]. Mértola went through multiple periods as an independently-ruled Muslim municipality or *taifa* but on two occasions was controlled by Berber Muslim armies in both 1020 CE and 1144 CE. In the 13^th^ c. CE it was then taken over by Christian forces [34]. Subsequently all of al-Andalus came under Christian control. The cesspit studied was located in the Almohad quarter, on the slope of de Castle, an area that previously contained numerous buildings dating to Late Antiquity. After the 13^th^ c. a cemetery area associated with the Church was established [34]. The sample included in this study came from the pit of a latrine in one of the houses along a street in the Alhomad quarter (Fossa 5, sample 1).

*Jerusalem, Israel/Palestine (15^th^–16^th^ c. CE)*

The two samples included in this study were collected during excavations in the courtyard of the Spanish School in the Christian quarter. A stone cesspit was excavated, charcoal from the cesspit was radiocarbon dated to 1304–1403 CE which falls within the Mamluk period. The cesspit also contained pottery from the late 1400s. This indicated that the cesspit was probably used between the 15^th^–16^th^ c. CE. Sediment removed was sieved and coprolites were recovered [35]. One coprolite from the cesspit (coprolite 11) as well as a sediment sample were included in this analysis.

# Sedimentary DNA Methods for Paleoparasitology

*Workflow*

All work was completed in dedicated ancient DNA rooms in the McMaster Ancient DNA Centre. A unidirectional workflow was used where DNA was amplified in separate rooms and individuals and samples could not move backwards to previous rooms where low copy number DNA was processed. A dedicated clean room was used for reagent preparation. Subsampling and digestion occurred in one room, followed by extraction, purification, library preparation and indexing in another. Targeted enrichment was done in a dedicated room. Quantitative PCRs, size selection, and pooling were done in a dedicated high-copy number room in a separate building. Labs and surfaces are regularly cleaned with 6% sodium hypochlorite followed by ultrapure water. The inside of hoods and reusable equipment is also subject to UV irradiation.

*Subsampling, Extraction and Purification*

A 0.25g subsample was taken from each sample. This was then added to a PowerBead tube containing garnet beads (Qiagen) with a custom in-house digestion buffer. The digestion buffer contained Tris-Cl, SDS, CaCl_2_, DTT, PTB, PVP and water (see Table Bi for concentrations). PowerBead tubes were then vortexed for 15 minutes to allow the garnet beads to break up materials in the sample, including parasite eggs. Proteinase K was added after vortexing and tubes were incubated at 35° C for 19 hours with constant oscillation. PowerBead tubes were then centrifuged at 10,000g for 5 min and supernatant removed to take through to purification.

Table Bi. Digestion solution mastermix added to PowerBead tubes.

|  | **Concentration in final solution** | **Manufacturer** |
| --- | --- | --- |
| Tris-Cl | 0.05 M | Fisher Scientific |
| SDS | 1.25% | Fisher Scientific |
| CaCl_2_ | 0.025 M | Fisher Scientific |
| Proteinase K | 0.63 mg/mL | Roche |
| DTT | 250 mM | Fisher Scientific |
| PTB | 12.50 mM | Oakwood Chemical |
| PVP | 6.25% | Fisher Scientific |
| Water | Added to final volume 500 µL mastermix for each sample |  |

After digestion, samples were purified following standard procedures [36]. Binding buffer for purifications contained guanidinium hydrochloride, isopropanol, Tween-20, and sodium acetate (see Table Bii for binding buffer recipe). Binding buffer (14 mL) was mixed with 1.25 mL of digestion supernatant. Samples then underwent the cold spin inhibitor removal procedure. Each tube was placed in a centrifuge run at 4500 rpm and cooled to 4°C for 24 hours. Previous experimentation has shown that addition of the cold spin with SDS added to digestion buffer effectively removes PCR inhibitors from sediment samples and improves ancient DNA recovery [37]. After the cold spin, dark colored pellets were formed at the bottom of each centrifuge tube with remaining supernatant clear in most cases. The supernatant from these tubes was filtered through silica columns (from Roche High Pure Viral Nucleic Acid Large Volume Kit). Columns were then placed in collection tubes and DNA eluted from the silica membrane with 50 µL of EBT.

Table Bii. Binding buffer recipe.

|  | **Concentration in final solution** | **Manufacturer** |
| --- | --- | --- |
| Guanidinium hydrochloride | 5 M | Fisher Scientific |
| Isopropanol | 40% | Fisher Scientific |
| Tween-20 | 0.05% | Sigma-Aldrich |
| Sodium acetate | 0.09 M | VWR |
| Water | Added to final volume 15mL binding buffer per sample |  |

*Library Preparation*

Library preparation for sequencing on the Illumina HiSeq 1500 platform followed standard protocols with minor adjustments based on our laboratory protocols [38,39]. Each sample that had already undergone extraction and purification was taken through blunt end repair to remove overhanging ends to leave only complementary double-stranded DNA fragments to which the adapters for sequencing can be attached. The blunt end repair mastermix is detailed in Table Biii. This was mixed with the purified extracts of each sample and run through the appropriate thermocycler settings (Table Biii) for blunt end repair. Each sample was then purified using the QiaQuick Nucelotide Removal kit (Qiagen). Adapters were then ligated (see Table Biv for adapter mastermix) and run through the appropriate thermocycler settings for adapter ligation (see Table Biv). Each sample was purified using the MinElute Purification kit (Qiagen) with 20 µL EB as the eluent. In order to add complementary adapters, samples then underwent adapter fill-in (see Table Bv for mastermix and thermocycler settings).

Table Biii. Blunt end repair mastermix and thermocycler settings.

|  | **Concentration in final solution** | **Manufacturer** |
| --- | --- | --- |
| NE buffer | 1X | NEB |
| DTT | 1 mM | Fisher Scientific |
| dNTPs | 100 µM | NEB |
| ATP | 1 mM | Fisher Scientific |
| T4 polynucleotide kinase | 0.5 U/mL | NEB |
| T4 DNA polymerase | 0.1 U/mL | NEB |
| Water | Added to final volume 30 µL mastermix per sample |  |
| **Thermocycler Settings** | |  |
| Temperature | Time |  |
| 25° C | 15 min |  |
| 12° C | 15 min |  |
| 4° C | hold |  |

Table Biv. Adapter ligation mastermix and thermocycler settings. The adapter mix contains a mix of P5 (forward and reverse) and P7 (forward and reverse) adapter mixes (220 µM for each P5 and P7) with oligo hybridization buffer (1X diluted from 10X). The 10X oligo hybridization buffer contains sodium chloride (500 mM), Tris-Cl (10 mM), EDTA (1 mM) and water to 2 mL. The P5 and P7 reactions are run through thermocycler separately then combined to create the final adapter mix. Thermocycler settings are 95° C for 10 sec followed by ramp of 95° to 12° C at a rate of 0.1° per sec.

|  | **Concentration in final solution** | **Manufacturer** |
| --- | --- | --- |
| T4 DNA ligase buffer | 1X | NEB |
| PEG 4000 | 1 mM | Sigma-Aldrich |
| Adapter Mix | 100 µM | IDT |
| T4 DNA ligase | 1 mM | NEB |
| Water | Added to final volume 30 µL mastermix per sample |  |
| **Thermocycler Settings** | |  |
| Temperature | Time |  |
| 25° C | 15 min |  |
| 12° C | 15 min |  |
| 4° C | hold |  |

Table Bv. Adapter fill-in mastermix and thermocycler settings.

|  | **Concentration in final solution** | **Manufacturer** |
| --- | --- | --- |
| ThermoPol rxn buffer | 1X | NEB |
| dNTPs | 250 µM | NEB |
| BST polymerase | 0.4 U/mL | NEB |
| Water | Added to final volume 20 µL mastermix per sample |  |
| **Thermocycler Settings** | |  |
| Temperature | Time |  |
| 37° C | 30 min |  |
| 80° C | 20 min |  |
| 4° C | hold |  |

*Indexing*

Each sample was indexed for post-sequencing identification. Unique forward and reverse indexes were chosen for each sample. See Table Bvi for indexing mastermix. Each library prepped sample with indexing mastermix was amplified using a thermocycler in a separate room. A fluorescent indicator was added so that we could perform fluorescence-guided qPCR amplification, allowing libraries to be removed from the thermocycler once they reached an amplification plateau. After qPCR, each sample was purified using the MinElute Purification kit (Qiagen) with 15 µL of EBT as the eluent. After indexing, samples either went on to enrichment, or directly to pooling and sequencing for those being shotgun sequenced.

Table Bvi. Indexing mastermix and thermocycler settings

|  | **Concentration in final solution** | **Manufacturer** |
| --- | --- | --- |
| KAPA SYBR FAST qPCR mastermix | 1X | Sigma-Aldrich |
| Forward index primer | 750 nM | IDT |
| Reverse index primer | 750 nM | IDT |
| Water | Added to final volume 27.5 µL mastermix per sample |  |
| **Thermocycler Settings** | | |
|  | Temperature | Time |
| Denaturation | 95° C | 5 min |
| Cycles (10) | 95° C | 30 sec |
|  | 60° C | 45 sec |
| Final Extension | 60° C | 3 min |

*Targeted Enrichment*

Targeted enrichment was done using an RNA bait set designed specifically for this study to target enteric parasites. For further details on bait design see section C. The parasite bait set was synthesized by Daicel Arbor Biosciences and the targeted enrichment method followed their protocol (Version 4). In brief, baits were diluted to 200 ng/reaction. A hybridization mastermix (containing the RNA baits) and library mastermix were made following Arbor protocols. The library mastermix was added to our indexed samples and incubated in a thermocycler at 95° C for 5 minutes, next the hybridization mastermix was incubated at 95° C for 5 min. 18 µL of the hybridization mastermix was then added to each library mastermix containing indexed libraries. These were then incubated in a thermocycler at 55° C for 48 hours.

The RNA baits are attached to biotinylated magnetic beads, thus after incubation to allow time for baits to bind to target parasite DNA in the samples, the RNA baits can then be pelleted using a magnet. The non-target DNA was then removed. Beads attached to target DNA were washed and resuspended in EBT. Reamplification was done using reamplification mastermix and appropriate reamplification program on the thermocycler as per manufacturer protocols. Beads were again pelleted and the supernatant containing target DNA was purified using the MinElute Purification kit (Qiagen) with 15 µL of EBT as the eluent.

*Pooling, Size Selection and Sequencing*

Each sample that was enriched, or indexed without enrichment for those undergoing shotgun sequencing, was quantified using a qPCR protocol and fluorescent indicator. This quantification allowed for pooling at equimolar concentrations prior to sequencing. After pooling, size selection using an agarose gel was done to remove adapter-dimers and other potential fragments too large or small to be consistent with aDNA. The gel was excised between the 150 to 600 bp range and these excised gel plugs were purified using the MinElute Gel Extraction kit (Qiagen). Samples were then sequenced on an Illumina HiSeq 1500 platform with a 2 x 90 bp paired-end protocol at the McMaster Farncombe Institute.

*Bioinformatics*

After sequencing, reads from all samples were merged and converted to a bam file. Adapter sequences were removed and sequences were merged with *leeHom* [40]. Sequences were then mapped to the parasite reference sequences used for the bait design. Sequences less than 24 bp were filtered out. These mapped and filtered reads were string de-duplicated using *NGSeXplore* (https://github.com/ktmeaton/NGSeXplore) and passed to *BLASTn*. Using *BLASTn* the top 100 alignments were returned using the NCBI Nucleotide database downloaded to a local computer. BLAST and fasta files were passed to *MEGAN* [41] for LCA assignment. The LCA parameters used were as follows: min score 50, max expected 1 x 10^-5^, min percent identity 95, top percent 15, min support 3, min read length 0, weighted LCA algorithm, and percent to cover 80. Read counts assigned to parasite taxa are reported in the results.

Sequence data not mapped to the parasite reference sequences from each sample were also analysed. Again, these sequences were filtered to remove sequences less than 24 bp, string de-duplicated and passed to BLASTn. For these unmapped sequences only the top 10 alignments were returned. The same LCA parameters as above were used. The unmapped sequences were analysed for the Must Farm coprolites as described in section D, to determine origin of the coprolites.

Fragment length distribution and misincorporation profiles for *Ascaris* reads were produced using *mapDamage* [42] as there were sufficient number of pooled reads from all samples for this taxa to run the statistical analysis required. Raw FASTAQ files were mapped to *Ascaris lumbricoides* (mitochondrion, complete genome 14,281 bp circular DNA Accession: NC_016198.1 GI: 357018117) and samples with the highest number of reads were pooled together.

# Parasite Bait Design

The input for the bait design was mitochondrial genomes, where available, as well as a few specific nuclear genes for 130 intestinal parasite species. Appendix B includes the accession numbers for the specific sequences used.

An iterative, hierarchical approach was taken. A multiple sequence alignment was

generated for each of the rRNA, NADH, and COI genes as well the mitochondrial genome.

Alignments were performed with MAFFT [43]. A single pair-wise distance matrix for all species was generated by taking the geometric mean distance between taxa across input sequences. A neighbor joining tree was generated using the PHYLIP package [44]. Pseudogenomes for a particular species were constructed by concatenating all available target sequences for a species together. COI and NADH genes were only used if the available mitochondrial sequences did not already include them.

From here candidate probes were identified via successive iterations of unique probe

identification and collapsing neighboring pseudogenomes together. Identification was done using SA-BOND [45] to exhaustively identify all 75bp sequences which are unique to each pseudogenome. Here unique is defined as having no more than 75% similarity and no more than 20 consecutive bp of identity to any other 75-mer from a different pseudogenome. All taxa which had fewer than 20 unique probes were noted, and the pseudogenomes for those taxa and their nearest neighbors were replaced with the consensus of each pair of sister sequences. SA-BOND was then rerun on the new set of pseudogenomes noting new candidate probes generated. This process continued until all taxa had at least 20 probes at some hierarchical level, or until no new probes could be generated. In this case 10 iterations were

required.

Any candidate probes which were contiguous (offset by a single bp) were combined

together into candidate probe regions which could be tiled by selecting any of the

75bp subsequences of the region. An effort was made to balance the number of probes

across taxa in the way described by Dickson et al. [45].

The final set of 30,240 probes probe covered 121 of the 130 species targeted. For specifics on the number of probes per species and gene see Appendix B.

# Results

For detailed results of microscopy, including eggs per gram of each taxa identified, and read counts broken down by parasite taxa see Table in Appendix C. Raw sequence data has been deposited in the NCBI Sequence Read Archive, Accession PRJNA119427.

**Table Di. Quantification results from qPCR of adapter-ligated molecules after library preparation, raw read count, filtered and mapped read count, LCA assigned read count from each specimen, and percentage of LCA assigned reads assigned to Kingdom-level.**

| **Sample** | **Adapter-Ligated Molecules after Library Preparation (copies/uL)** | **Raw Read Count** | **Filtered and Mapped Read Count** | **LCA Assigned Read Count** | **Bacteria (%)** | **Fungi (%)** | **Metazoa (%)** | **Sar (%)** | **Virdiplantae (%)** |  |
| --- | --- | --- | --- | --- | --- | --- | --- | --- | --- | --- |
|  |  |  |  |  |  |  |  |  |  |  |
| MP-77 | 2.28E+07 | 5.55E+06 | 487 | 71 | 83 | 0 | 0 | 7 | 0 |  |
| MP-85 | 3.27E+06 | 4.38E+06 | 1049 | 125 | 59 | 15 | 2 | 0 | 3 |  |
| MP-74 | 1.05E+09 | 2.26E+06 | 1797 | 303 | 84 | 0 | 2 | 0 | 4 |  |
| MP-26 | 8.18E+08 | 8.23E+06 | 1854 | 861 | 93 | <1 | <1 | <1 | 2 |  |
| MP-33 | 4.45E+08 | 7.74E+06 | 1418 | 371 | 87 | 0 | 0 | 0 | 4 |  |
| MP-37 | 1.10E+09 | 7.84E+06 | 1651 | 553 | 89 | 0 | <1 | 0 | 3 |  |
| MP-12 | 9.78E+08 | 5.53E+06 | 2015 | 1065 | 85 | <1 | <1 | <1 | 7 |  |
| MP-23 | 7.70E+08 | 7.48E+06 | 2129 | 987 | 85 | <1 | 0 | 1 | 6 |  |
| MP-94 | 6.95E+08 | 2.24E+06 | 1251 | 821 | 74 | 1 | 2 | 5 | 6 |  |
| MP-39 | 1.89E+07 | 2.14E+06 | 762 | 236 | 78 | 0 | 15 | 0 | 0 |  |
| MP-86 | 1.08E+07 | 3.48E+06 | 696 | 127 | 21 | 0 | 2 | 0 | 3 |  |
| MP-87 | 4.88E+06 | 2.37E+06 | 582 | 137 | 61 | 0 | 7 | 0 | 8 |  |
| MP-88 | 2.10E+08 | 1.79E+06 | 1033 | 542 | 67 | 0 | <1 | 1 | 12 |  |
| MP-91 | 2.59E+07 | 2.59E+06 | 1055 | 326 | 59 | 2 | 4 | 0 | 12 |  |
| MP-92 | 1.02E+08 | 2.80E+06 | 1080 | 450 | 63 | <1 | 3 | <1 | 13 |  |
| MP-93 | 7.42E+07 | 1.86E+06 | 1116 | 495 | 65 | <1 | 2 | 1 | 12 |  |
| MP-45 | 4.14E+08 | 4.22E+06 | 1175 | 780 | 92 | 0 | 0 | 0 | 1 |  |
| MP-52 | 5.01E+08 | 6.43E+06 | 1435 | 429 | 81 | 0 | 3 | 2 | 4 |  |
| MP-53 | 2.56E+08 | 7.24E+06 | 1653 | 620 | 70 | <1 | 13 | 1 | 4 |  |
| MP-82 | 3.90E+05 | 1.43E+06 | 265 | 40 | 48 | 0 | 0 | 0 | 23 |  |
| MP-47 | 5.85E+07 | 4.32E+06 | 1155 | 615 | 87 | 0 | <1 | <1 | 5 |  |
| MP-49 | 7.84E+07 | 5.95E+06 | 1478 | 682 | 82 | 0 | <1 | 0 | 5 |  |
| MP-89 | 2.60E+08 | 2.27E+06 | 1086 | 601 | 50 | 3 | 1 | 1 | 26 |  |
| MP-90 | 8.44E+07 | 1.63E+06 | 884 | 543 | 57 | 3 | <1 | 0 | 20 |  |
| MP-57 | 8.92E+08 | 1.05E+07 | 2917 | 1231 | 28 | 1 | 58 | <1 | 3 |  |
| MP-63 | 2.10E+07 | 3.57E+06 | 399 | 103 | 60 | 0 | 14 | 0 | 12 |  |
| MP-65 | 1.43E+08 | 6.45E+06 | 2629 | 1144 | 52 | <1 | 38 | 1 | 2 |  |

The MEGAN results for MP-57 assigned three reads to *Ascaris lumbricoides* at the species level. Given minimal genetic diversity between *Ascaris suum* and *Ascaris lumbricoides* these reads were aligned to both of the reference sequences of these two species using bowtie2. As can be seen in Fig Di these three reads align with 100% identity to both references thus cannot be used to make a species-level identification.


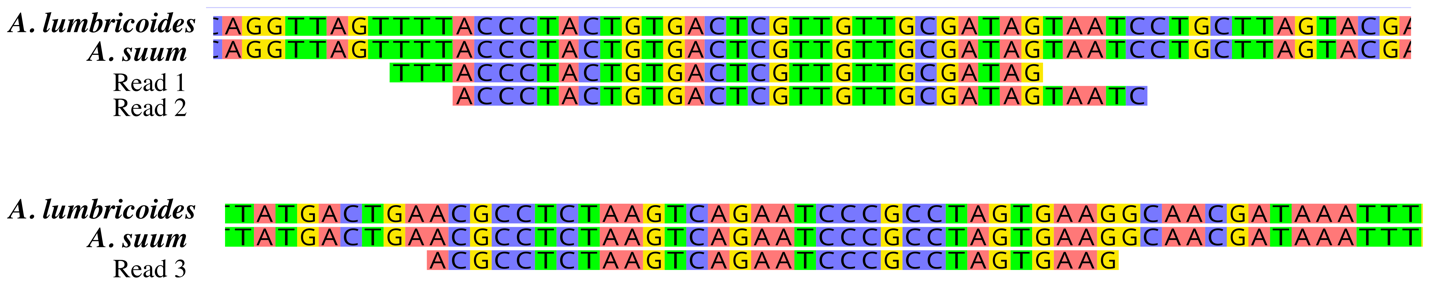


**Fig Di. Three reads assigned to Ascaris lumbricoides using MEGAN aligned to *A. lumbricoides* (GCA_015227635.1) and *A. suum* (GCA_013433145.1) reference sequences.** All three reads aligned with 100% identity to *A. lumbricoides* and *A. suum* alignment of reference sequences confirms lack of genetic diversity in these regions.

# Coprolite Identification

Neolithic and Bronze Age coprolites included in our study came from the settlement of Durrington Walls near Stonehenge in Britain, as well as the Bronze Age settlement of Must Farm in Britain. For both sites, the source of the coprolites was previously determined using established methods relying on fecal lipid biomarkers. We included coprolites that had been identified as human coprolites. However, the aDNA data provided further data for source identification and suggest that these coprolites came from dogs rather than humans.

The Must Farm coprolites were recovered from the waterlogged fluvial silts beneath, and contemporary with, the pile-built structures at the site [14]. Fecal lipid biomarker profiles indicated that some of the coprolites collected were of human origin and some were from dogs [14]. We only included three of the coprolites recovered from the site in this analysis (SF3127, SF3631, and SF4029). Two of the three coprolites underwent shotgun sequencing (SF3127 and SF4029) in addition to targeted enrichment. To identify the source of coprolites, sequence data was trimmed, merged, filtered and deduplicated as per our pipeline for parasite identification. Reads were not mapped to the parasite reference sequences. They were run through megablast to return the top 50 hits. MEGAN was then used to assign these reads to the lowest common taxonomic rank.

The Durrington Walls and Must Farm coprolites contained a high proportion of dog (*Canis lupus familiaris*) DNA which suggests that these coprolites are of dog origin (Fig Ei). Enrichment and shotgun samples from these coprolites contained between 2,789–18,146 reads assigned to *Canis lupus familiaris* (4.5–10% of LCA assigned reads). In comparison there were between 8 to 94 reads (0.02–0.06%) assigned to *Homo sapiens* in these same samples. Additionally, sediment samples from Must Farm contained very little dog DNA (11–70 reads assigned to *Canis lupus* in MP12 and MP23). The parasites recovered from these coprolites are not host specific and can infect humans and dogs. Identifying them as dog coprolites shows that dogs were infected with these parasites and their fecal material was found in the water around the site. As all of these parasites can infect humans, those living at the site would have been at high risk for infection and some likely were infected.

The coprolite from Durrington Walls (11465.1) similarly contained a very high proportion of dog DNA. In total 82,739 reads were assigned to *Canis lupus*, this was 74% of LCA-assigned reads. In comparison only 44 reads were assigned to *Homo sapiens*.

Reads assigned to *Canis* or lower taxonomic rank were extracted from MEGAN for each coprolite and run through mapDamage to confirm they were consistent with aDNA. The fragment length distribution and deamination profiles for these reads is clearly consistent with aDNA supporting the identification of these coprolites as ancient dog coprolites (see Fig Ei and Ei).


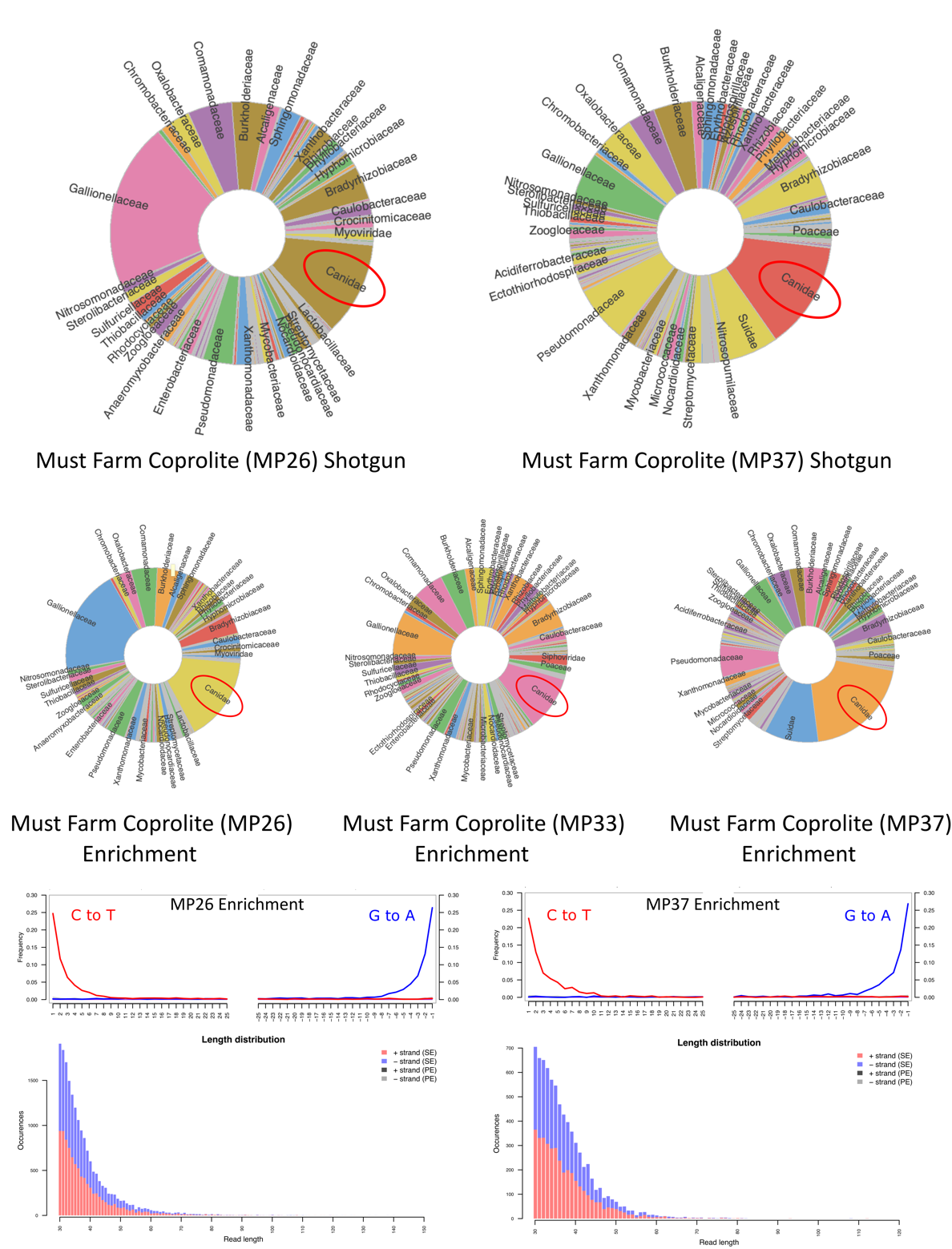


**Fig Ei. Read counts binned to Family level from Must Farm coprolites (SF3127, SF3631, and SF4029) and mapDamage plots.** Reads assigned to Canidae are circled in red.


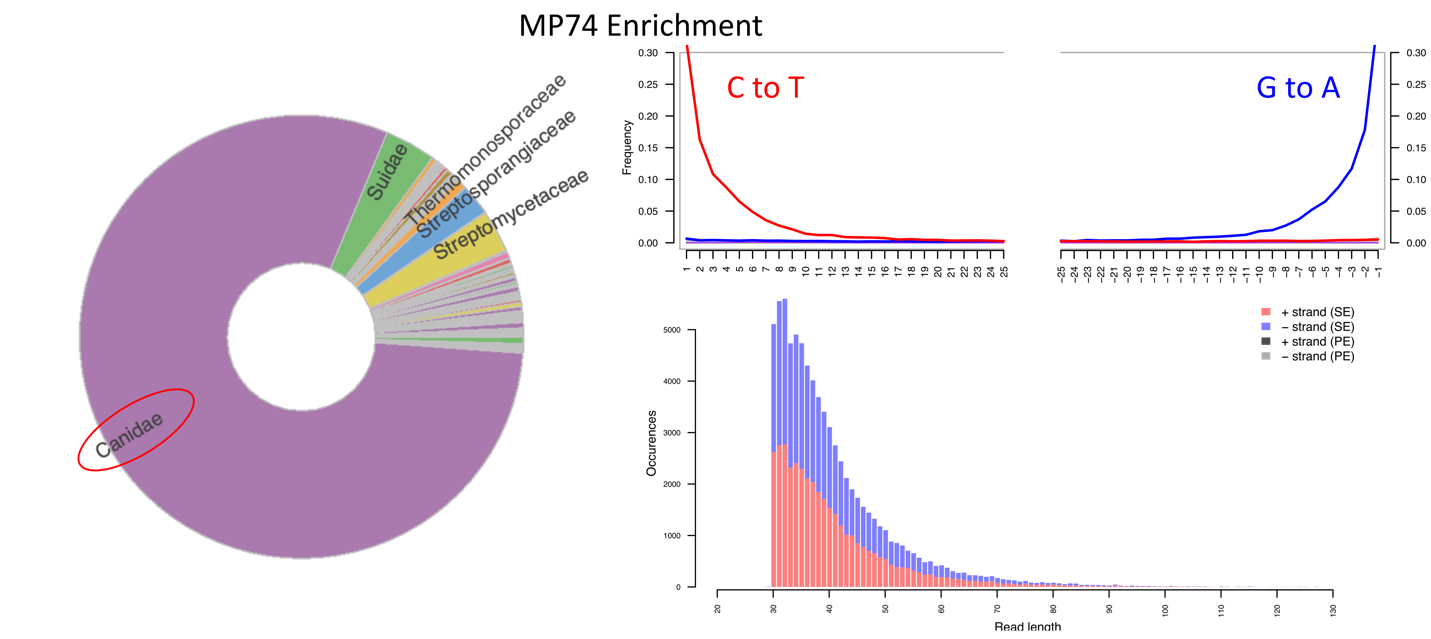


**Fig Eii. Read counts binned to Family level from Durrington Walls coprolite (11465.1) and mapDamage plots.** Reads assigned to Canidae are circled in red.

Some samples from known human contexts including latrines and pelvic soil samples had DNA of a very clear human association. Though these samples were enriched with our parasite bait set we still retain DNA that is consistent with human fecal material. DNA from *Homo sapiens* was identified and as would be expected in human fecal material reads assigned to common human bacterial gastrointestinal commensal flora. For example, in sediment from the medieval period latrine from Mértola, Portugal *Bifidobacterium* and *Clostridium* dominate the bacterial species recovered while *Ruminococcus* is seen in slightly lower amounts. This is consistent with dominant flora seen in modern human gut microbiomes from Europe [46]. Interestingly, samples which had high LCA-assigned reads to common human gut microbiota were samples where we were better able to recover parasite DNA. This suggests that these samples likely had a higher fecal content.


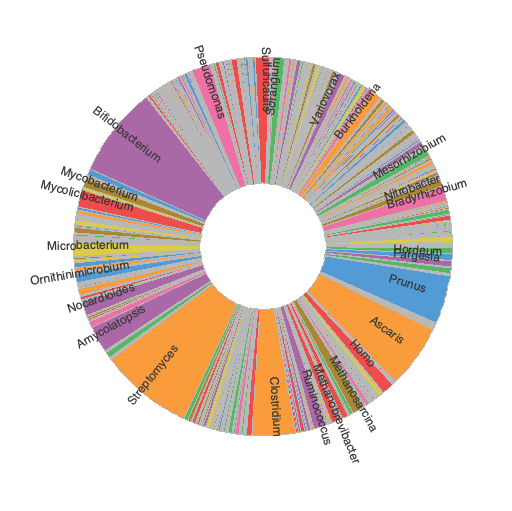


**Fig Eiii. LCA-assigned reads binned to genus-level in sediment from the medieval period latrine from Mértola (MP57).** Common bacteria found in the human gut microbiome in Europe are present including *Bifidobacterium*, *Clostridium* and *Ruminococcus* alongside *Ascaris*.


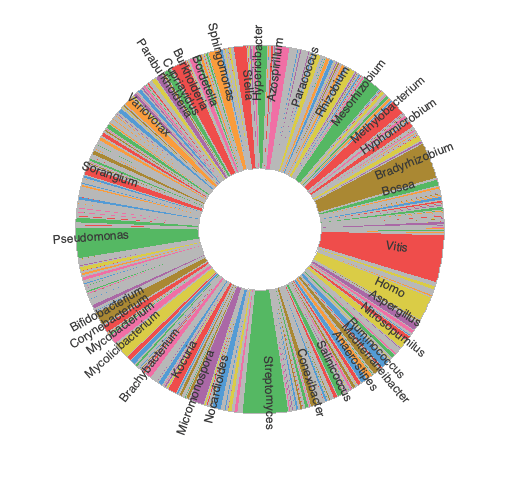

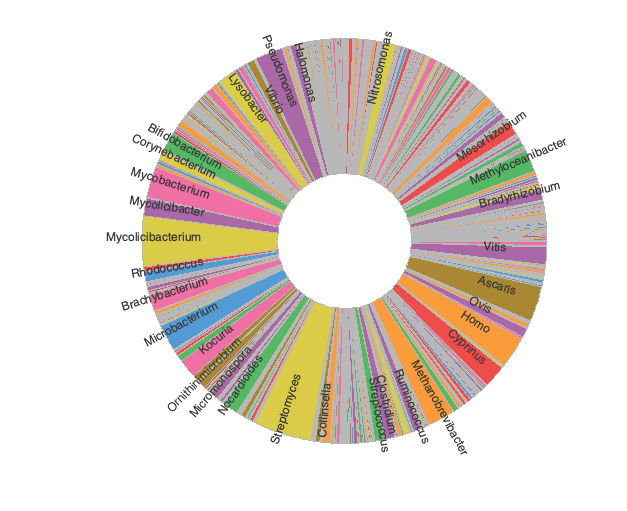


**Fig Eiv. LCA-assigned reads binned to genus-level in sediment from the medieval period coprolite from Jerusalem (MP63), top, and associated sediment (MP65), bottom.**


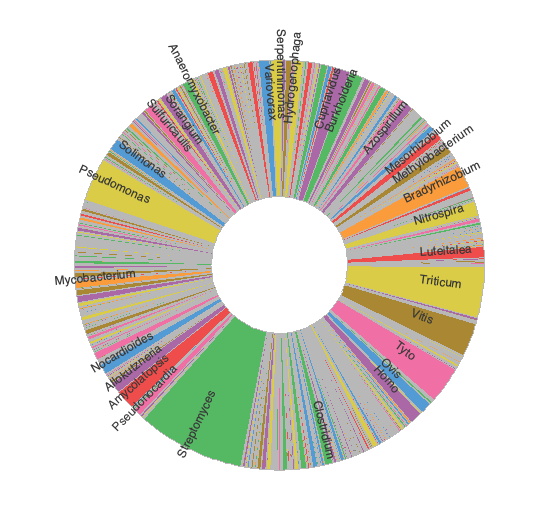


**Fig Eiv. LCA-assigned reads binned to genus-level in sediment from the Roman period latrine from Ephesus (MP49).**

# Review of Paleoparasitological Literature

Table Fi. Published results of gastrointestinal parasites from Pre-Roman (Neolithic, Bronze Age, and Iron Age) sites that are located in regions once part of the Roman Empire.

| **Country** | **Site** | **Date** | **Parasites** | **Sample Type** | **Citation** |
| --- | --- | --- | --- | --- | --- |
| Austria | Hallstatt salt mines | 800–350 BCE | *Ascaris lumbricoides* *Trichuris trichiura* | Coprolites | [47,48] |
|  | Hallein salt mine | 500–200 BCE | *Ascaris* sp.  *Dicrocoelium* sp.  *Fasciola hepatica*  *Taenia* sp.  *Trichuris trichiura* | Coprolites | [47–50] |
| Britain | Brean Down | 1940–650 BCE | *Ascaris lumbricoides* *Trichuris trichiura* | Coprolites | [51] |
|  | Bulford | Bronze Age | *Ascaris* sp.  *Taenia* sp. | Pelvic soil | [52] |
| Cyprus | Shillouroka-mbos | 8300–7000 BCE | *Ascaris lumbricoides Dibothriocephalus* sp. *Fasciola* sp.  *Taenia* sp.  *Trichuris trichiura* | Pelvic soil | [53] |
|  | Khirokitia | 7000–6000 BCE | *Ascaris lumbricoides Fasciola* sp.  *Taenia* sp.  *Trichuris trichiura* | Pelvic soil | [53] |
| Czechia | Hulín | 1600–1500 BCE | *Ancylostoma duodenale* *Ascaris lumbricoides* *Trichuris trichiura* | Pelvic soil | [54] |
| Egypt | Mummy Nakht-ROM I | 1200 BCE | *Schistosoma* sp.  *Taenia* sp.  *Trichinella spiralis* | Mummy | [55,56] |
|  | Mummies from Thebes | 1198–1150 BCE | *Schistosoma haematobium* | Mummy | [57] |
|  | Mummy Asru from Thebes | 750–525 BCE | *Echinococcus* sp. *Strongyloides* sp. | Mummy | [58] |
|  | Deir el-Medineh | 400–300 BCE | *Ascaris lumbricoides* *Dibothriocephalus* sp.  *Fasciola hepatica*  *Trichuris trichiura* | Pelvic soil | [59] |

|  | Saqqara | 400–300 BCE | *Dibothriocephalus* sp. *Fasciola* sp.  *Trichuris trichiura* | Pelvic soil | [59] |
| --- | --- | --- | --- | --- | --- |
|  | Mummy | 200–1 B.C.E | *Taenia* sp. | Mummy | [60] |
|  | Mummy Pum II | 170 BCE | *Ascaris lumbricoides* | Mummy | [61] |
| France | Clairvaux | 3600 BCE | *Ancylostomatidae*  *Dibothriocephalus* sp.  *Fasciola* sp.  *Trichuris* sp. | Sediment | [62] |
|  | Grésine, Bourget Lake | 3400–3230 BCE | *Dicrocoelium* sp. *Dibothriocephalus* sp. *Entamoeba histolytica* | Occupation sediment | [50,63,64] |
|  | Chalain | 3200–2980 BCE | *Capillaria* sp.  *Dicrocoelium* sp. *Dibothriocephalus* sp. *Fasciola hepatica*  *Taenia* sp.  *Trichuris trichiura* | Coprolites/ occupation sediment | [62,65–67] |
| Germany | Leipzig-Zwenkau | 5259–5258 BCE | *Dicrocoelium* sp. | Pit | [50] |
|  | Hornstaad-Hornle | 3917–3905 BCE | *Dicrocoelium* sp. *Dibothriocephalus* sp. *Fasciola* sp.  *Trichuris* sp. | Occupation sediment | [68] |
|  | Sipplingen | 3711-3306 BCE | *Dicrocoelium* sp. *Dibothriocephalus* sp. *Fasciola* sp.  *Taenia* sp.  *Trichuris* sp. | Occupation sediment | [68] |
|  | Wallhausen-Ziegelhütte | 3700–2900 BCE | *Capillaria* sp. *Dibothriocephalus* sp. *Fasciola* sp.  *Trichuris* sp. | Occupation sediment | [68] |
|  | Torwiesen II | 3283–3281 BCE | *Capillaria* sp. *Dibothriocephalus* sp. *Fasciola* sp.  *Taenia* sp.  *Trichuris* sp. | Occupation sediment | [68] |

|  | Seekirch-Stockwiesen | 3000–2900 BCE | *Capillaria* sp.  *Dicrocoelium* sp. *Dibothriocephalus* sp. *Fasciola* sp.  *Trichuris* sp. | Occupation sediment | [69] |
| --- | --- | --- | --- | --- | --- |
|  | Karsdorf | 3000–2000 BCE | *Fasciola hepatica* | Pelvic soil | [70] |
|  | Alleshausen-Taschenwiese | 2900–2600 BCE | *Dicrocoelium* sp. | Occupation sediment | [50] |
| Greece | Kouphovou-no | 5000–2000 BCE | *Entamoeba histolytica* | Pelvic soil | [71] |
|  | Kephala | 4000–3000 BCE | *Trichuris trichiura* | Pelvic soil | [72] |
|  | Ayia Irini | 1600–1100 BCE | *Ascaris* sp. | Pelvic soil | [73] |
| Israel/ Palestine | Armon Hanatziv | 7^th^ c. BCE | *Ascaris lumbricoides*  *Enterobius vermicularis*  *Dibothriocephalus* sp.  *Taenia* sp.  *Trichuris trichiura* | Cesspit | [74] |
|  | City of David, Jerusalem | 700–500 BCE | *Taenia* sp.  *Trichuris trichiura* | Latrine | [75] |
| Italy | Otzi mummy | 3300–3200 BCE | *Trichuris trichiura* | Mummy | [76] |
| Netherlands | Swifterbant | 3400–3230 BCE | *Capillaria* sp.  *Fasciola* sp.  *Opisthorcis* sp.  Oxyuridae  *Trichuris* sp. | Coprolites | [77] |
|  | Amsterdam | 3400–3230 BCE | *Trichuris* sp. | ? | [78,79] |
| Spain | La Draga | 5320–4980 BCE | *Ascaris* sp.  *Capillaria* sp.  *Dicrocoelium dendriticum* *Dibothriocephalus* sp. *Enterobius vermicularis*  *Maracanthorhynchus* sp.  *Taenia saginata*  *Trichuris trichiura* | Occupation sediment | [76,77] |
| Switzerland | Concise | 3700 BCE | *Dicrocoelium* sp. *Entamoeba histolytica* | Occupation sediment | [50,78] |
|  | Arbon | 3384–3370 BCE | *Ascaris* sp.  *Capillaria* sp.  *Dicrocoelium* sp. *Dioctophyma renale* *Dibothriocephalus* sp.  *Entamoeba histolytica* *Fasciola* sp.  *Opisthorchis* sp.  *Taenia* sp.  *Trichuris* sp. | Coprolites | [62,63,79] |
|  | Parkhaus-Opéra, Zürich | 3176–3153 BCE | *Ascaris* sp.  *Capillaria* sp.  *Dicrocoelium* sp. *Dibothriocephalus* sp. *Echinostoma* sp.  *Fasciola gigantica* *Taenia*/*Echinococcus* sp. *Trichuris trichiura* | Occupation sediment | [80,81] |
| Sudan | Sai Island | 700–300 BCE | *Ascaris lumbricoides* *Dibothriocephalus* sp. *Enterobius vermicularis* *Fasciola* sp.  *Schistosoma haematobium* *Schistosoma mansoni* *Taenia* sp.  *Trichuris trichiura* | Pelvic soil | [83] |
| Syria | Tell Zeidan | 4550–4050 BCE | *Schistosoma* sp. | Pelvic soil | [81,82] |

Table Fii. Published results of gastrointestinal parasites from Roman period sites

| **Country** | **Site** | **Date** | **Parasites** | **Sample Type** | **Citation** |
| --- | --- | --- | --- | --- | --- |
| Austria | Carnuntum | 101–300 CE | *Ascaris lumbricoides*  *Taenia* sp.  *Trichuris trichiura* | Sewer and latrine | [82,83] |
| Belgium | Arlon | 1–300 CE | *Ascaris* sp.  *Trichuris* sp. | Vats or pits | [15,84] |
|  | Mageroy | Roman | *Entamoeba histolytica* | Latrine | [87] |
| Britain | Leicester | 1–200 CE | *Ascaris* sp.  *Fasciola* sp.  *Trichuris* sp. | Cesspit | [85] |
|  | London, Hibernia Wharf | 1–200 CE | *Ascaris lumbricoides* *Dibothriocephalus latum* *Taenia* sp.  *Trichuris trichiura* | Well | [86] |
|  | Orton Longueville | 1–200 CE | *Echinococcus granulosus* | Cyst from skeleton | [87] |
|  | Carlisle | 1–300 CE | *Ascaris lumbricoides* *Fasciola* sp.  *Trichuris trichiura* | Occupation sediment | [88] |
|  | Ambleside | 1–400 CE | *Ascaris* sp.  *Trichuris trichiura* | Pit | [89] |
|  | Church Street Sewer, York | 1–500 CE | *Ascaris* sp.  *Trichuris* sp. | Sewer | [90] |
|  | London, 15–35 Copthall Ave. | 101–400 CE | *Dicrocoelium dendriticum* *Trichuris trichiura* | Occupation sediment | [91] |
|  | Bearsden | 142–158 CE | *Ascaris* sp.  *Trichuris* sp. | Sewer | [92] |
|  | Lincoln, Waterside NW | 301–400 CE | *Trichuris* sp. | Occupation sediment | [93] |
|  | Bleadon | Roman | *Ascaris* sp. | Pelvic soil | [93] |
|  | Canterbury | Roman | *Ascaris* sp.  *Dibothriocephalus* sp.  *Taenia sp.* | Pelvic soil | [94] |
|  | Churchill | Roman | *Ascaris* sp.  *Taenia* sp. | Pelvic soil | [95] |
|  | York | Roman | *Ascaris* sp. | Pelvic soil | [96] |
|  | Owslebury, Winchester | Roman | *Ascaris* sp.  *Dicrocoelium* sp. *Trichuris*/*Capillaria* sp. | Pit | [94] |
|  | Poundbury, Dorset | Roman | *Ascaris lumbricoides* *Trichuris trichiura* | Coffin sediment | [95] |

| Egypt | Dakhleh Oasis | 395 BCE–30 CE | *Enterobius vermicularis* | Mummy | [96] |
| --- | --- | --- | --- | --- | --- |
|  | El-Deir | 301–500 CE | *Taenia* sp. | Mummy | [97] |
| France | Bobigny hospital | 201 BCE–100 CE | *Ascaris lumbricoides* *Trichuris trichiura* | Pelvic soil | [98] |
|  | Marseille | 14 BCE–27 CE | *Dibothriocephalus* sp.  *Fasciola* sp.  *Trichuris* sp. | Occupation sediment | [99] |
|  | Lattes | 1–200 CE | *Entamoeba histolytica* | Cesspit | [101,102] |
|  | Beauvais | 1–300 CE | *Ascaris* sp.  *Capillaria sp.*  *Dicrocoelium* sp.  *Macracanthorhynchus* sp.  *Taenia*/*Echinococcus* sp. *Trichuris* sp. | Latrine | [100] |
|  | Horbourg-Wihr | 1–300 CE | *Ascaris* sp.  *Capillaria* sp.  *Dicrocoelium* sp. *Dibothriocephalus sp. Fasciola* sp. *Macracanthorhynchus* sp.  *Taenia*/*Echinococcus* sp. *Trichuris* sp. | Pits, latrines, ditches | [100] |
|  | Bordeaux | 40–51 CE | *Ascaris* sp.  *Taenia* sp.  *Trichuris* sp. | Sewer or latrine | [101] |
|  | Reims, Rue Venise | 101–200 CE | *Ascaris* sp.  *Fasciola* sp.  *Trichuris* sp. | Pits, wells, occupation sediment | [102,103] |
|  | Jaunay-Clan | 201–300 CE | *Trichuris trichiura* | Coffin sediment | [104] |
|  | La Gramiere, Castillon du Gard | 201–300 CE | *Entamoeba histolytica* | Cesspit | [63] |
|  | Amiens | 201–401 CE | *Capillaria hepatica* *Echinococcus granulosus* | Cyst from skeleton | [100] |
|  | Andilly-en-Bassigny | Roman | *Ascaris* sp.  *Fasciola* sp.  *Taenia*/*Echinococcus* sp. | ? | [100] |
|  | Autun | Roman | *Ascaris* sp.  *Capillaria* sp. *Macracanthorhynchus* sp.  *Taenia*/*Echinococcus* sp. *Trichuris* sp. | ? | [100] |
|  | Evreux | Roman | *Ascaris* sp.  *Trichuris* sp. | Pelvic soil | [106] |
|  | Lisses | Roman | *Entamoeba histolytica* | Pit | [100] |
|  | Metz | Roman | *Ascaris* sp.  *Capillaria* sp.  *Dibothriocephalus* sp.  *Fasciola* sp.  *Toxocara* sp.  *Trichuris* sp. | ? | [100] |
|  | Mikelauen-Zilo | Roman | *Dicrocoelium* sp. *Dibothriocephalus* sp. | Occupation sediment | [50,64] |
|  | Reims, Rue Carnot | Roman | *Dicrocoelium* sp. | Cesspit | [50] |
|  | Troyes, Place de la Liberation | Roman | *Dicrocoelium* sp. *Entamoeba histolytica* | Cesspit | [50,78] |
|  | Villevieille | Roman | *Ascaris* sp. | ? | [110] |
| Germany | Ladenburg | 1–200 CE | *Ascaris* sp.  *Trichuris* sp. | Latrine | [109,110] |
|  | Belginum | 101–300 CE | *Ascaris* sp.  *Capillaria* sp.  *Taenia* sp. | Pit | [111] |
|  | Künzing | 140–250 CE | *Trichuris trichiura* | Pit | [112] |
| Greece | Ayia Irini | 101–300 CE | *Ascaris lumbricoides*, *Trichuris trichiura* | Pelvic soil | [83] |
| Israel/ Palestine | Qumran | 100 BCE–68 CE | *Ascaris lumbricoides* *Dicrocoelium* sp. *Enterobius vermicularis* *Taenia* sp.  *Trichuris trichiura* | Latrine | [113] |
|  | Silwan | 1–100 CE | *Echinococcus granulosus* | Cyst from skeleton | [100] |
|  | Nahal-Mishmar Valley | 160 CE | *Trichuris trichiura* | Coprolites | [114] |
|  | Caesarea | Roman | *Ascaris lumbricoides* *Dibothriocephalus* sp. | Latrine | [117,118] |
| Italy | Pompeii | 100 BCE–79 CE | *Trichuris trichiura* | Sewer | [115] |
|  | Lucus Feroniae | 27 BCE–568 CE | *Ascaris* sp. | Pelvic soil | [114] |
|  | Portus | 25–660 CE | *Ascaris* sp.  *Fasciola* sp.  *Trichuris* sp. | Sediment cores | [117] |
|  | Gerace | 380–500 CE | *Trichuris trichiura* | Chamber pot | [115] |
|  | Roma | Roman | *Entamoeba histolytica* | ? | [120] |
| Netherlands | Uitgeest | 101 BCE–300 CE | *Trichuris* sp. | Well | [116] |
|  | Alphen on the Rhine | 1–100 CE | *Ascaris* sp.  *Taenia/Echinococcus* sp.  *Trichuris* sp. | Latrine | [117] |
|  | Valkenburg (Roman Army Camp) | 1–100 CE | *Ascaris lumbricoides* *Trichuris trichiura* | Occupation sediment | [118] |
| Switzerland | Augst | 1–100 CE | *Ascaris* sp.  *Trichuris* sp. | Latrine | [119,120] |
|  | Eschenz | 1–200 CE | *Ascaris lumbricoides*  *Trichuris trichiura* | Latrine | [121] |
|  | Vindonissa | 30–45 CE | Ascarididae  *Trichuris* sp. | Latrines | [122] |
| Türkiye | Sagalassos | 101-500 CE | *Ascaris* sp.  *Giardia duodenalis* | Latrine | [123] |

Table Fiii. Published results of parasites from Post-Roman (Late Antique and Medieval period) sites that are located in regions once part of the Roman Empire.

| **Country** | **Site** | **Date** | **Parasites** | **Sample Type** | **Citation** |
| --- | --- | --- | --- | --- | --- |
| Austria | Am Hof, Wien | Medieval | *Ascaris lumbricoides* | Sewer | [124] |
| Belgium | Namur | 9^th^–15^th^ c. CE | *Ascaris* sp.  *Dibothriocephalus* sp.  *Entamoeba histolytica*  *Fasciola hepatica*  *Taenia* sp.  *Trichuris* sp. | Cesspit | [63,125] |
|  | Nivelles | 10^th^–13^th^ c. CE | *Ascaris lumbricoides* *Trichuris trichiura* | Coprolites | [126] |
|  | Aalst | 12^th^–18^th^ c. CE | *Ascaris* sp.  *Dicrocoelium dendriticum*  *Echinostoma* sp.  *Trichuris trichiura* | Cesspit | [127] |
|  | Kartuizerstraat, Brussels | 1276–1368 CE | *Ascaris* sp.  *Capillaria* sp.  *Dicrocoelium dendriticum*  *Entamoeba histolytica*  *Fasciola hepatica*  *Giardia duodenalis*  *Trichuris trichiura* | Latrine | [128] |
|  | Oude Schaapmarkt, Ghent | 13^th^–18^th^ c. CE | *Ascaris* sp.  *Entamoeba histolytica*  *Giardia duodenalis*  *Trichuris* sp. | Cesspit | [129] |
|  | Sint Baafs Cathedral, Ghent | 13^th^–18^th^ c. CE | *Ascaris* sp.  *Giardia duodenalis*  *Trichuris* sp. | Pelvic soil and cesspit | [129] |

|  | Malines Prison | 14^th^ c. CE | *Ascaris* sp.  *Trichuris* sp. | Cesspits | [130] |
| --- | --- | --- | --- | --- | --- |
|  | Palace of the Dukes of Burgundy, Bruges | 14^th^–15^th^ c. CE | *Ascaris* sp.  *Trichuris* sp. | Cesspits | [131] |
|  | Eenmanstraat, Brussels | 14^th^–16^th^ c. CE | *Ascaris* sp.  *Dicrocoelium dendriticum*  *Entamoeba histolytica*  *Fasciola hepatica*  *Giardia duodenalis*  *Taenia* sp.  *Trichuris trichiura* | Latrine | [128] |
|  | Raversijde | 16^th^ c. CE | *Ascaris* sp.  *Capillaria* sp.  *Trichuris* sp. | Latrines | [132] |
| Britain | London | 600–871 CE | *Ascaris* sp.  *Taenia* sp.  *Trichuris trichiura* | Pit | [133] |
|  | Worcester | 680–1066 CE | *Ascaris* sp.  *Trichuris* sp. | Pelvic soil | [80] |
|  | Ipswich | 9^th^–14^th^ c. CE | *Ascaris* sp.  *Dibothriocephalus* sp.  *Taenia* sp.  *Trichuris* sp*.* | Pelvic soil | [134,135] |
|  | Lincoln, Waterside NW and Woolworth’s | 10^th^–13^th^ c. CE | *Ascaris* sp.  *Trichuris* sp*.* | Occupation sediment | [133] |
|  | All Saints Parish | 10^th^–14^th^ c. CE | *Ascaris* sp. | Pelvic soil | [134] |
|  | York (Bedern) | 10^th^–14^th^ c. CE | *Ascaris* sp.  *Trichuris* sp. | Latrine | [135] |
|  | York, 16–22 Coppergate | 10^th^–14^th^ c. CE | *Ascaris* sp.  *Trichuris* sp. | Pit | [135] |
|  | York | 1000 CE | *Ascaris* sp.  *Taenia* sp.  *Trichuris trichiura* | Coprolite | [135,136] |
|  | Winchester | 11^th^–12^th^ c. CE | *Ascaris* sp.  *Dicrocoelium dendriticum*  *Trichuris* sp. | Pit | [136,137] |
|  | London, 15–35 Copthall Ave | 11^th^–12^th^ c. CE | *Ascaris lumbricoides* *Trichuris trichiura* | Ditch | [137] |
|  | York | 11^th^–16^th^ c. CE | *Ascaris* sp.  *Trichuris* sp. | Pelvic soil | [138] |

|  | Finzels Reach, Bristol | 1150–1700 CE | *Ascaris* sp.  *Trichuris trichiura* | Waste deposit | [137] |
| --- | --- | --- | --- | --- | --- |
|  | Southampton | 13^th^–14^th^ c. CE | *Ascaris* sp.  *Trichuris* sp. | Cesspit | [138] |
|  | Augustinian Friars | 13^th^–16^th^ c. CE | *Ascaris* sp.  *Trichuris trichiura* | Pelvic soil | [134] |
|  | Southampton | 13^th^–16^th^ c. CE | *Ascaris* sp.  *Taenia* sp. | Pelvic soil | [138] |
|  | York | 14^th^–16^th^ c. CE | *Ascaris* sp.  *Trichuris* sp. | Latrine | [139] |
|  | Worcester | 15^th^ c. CE | *Ascaris* sp.  *Trichuris* sp. | Latrine | [140] |
|  | King Richard III | 1485 CE | *Ascaris lumbricoides* | Pelvic soil | [141] |
|  | Leicester | Medieval | *Ascaris* sp.  *Fasciola* sp.  *Trichuris* sp. | Cesspit | [143] |
|  | London, Hibernia Wharf | Medieval | *Ascaris lumbricoides* *Dibothriocephalus latum* *Fasciola hepatica*  *Taenia* sp.  *Trichuris trichiura* | Pit | [141] |
| Cyprus | Saranda Kolones | 1191–1222 CE | *Ascaris lumbricoides Trichuris trichiura* | Latrine | [142] |
| Czechia | Breclav-Pohansko | 850–950 CE | *Ascaris* sp.  *Trichuris trichiura* | Pelvic soil | [142] |
|  | Chrudim | 13^th^–18^th^ c. CE | *Ascaris lumbricoides* *Dibothriocephalus latum* *Enterobius vermicularis* *Fasciola hepatica*  *Giardia duodenalis* *Hymenolepis nana*  *Toxocara canis*  *Trichuris trichiura* | Cesspit | [143] |
| France | Collège de France | 4^th^–5^th^ c. CE | *Dicrocoelium* sp. | Occupation sediment | [50] |
|  | Church of Saint-Martin-au-Val, Chartres | 5^th^–9^th^ c. CE | *Ascaris lumbricoides*  *Dibothriocephalus* sp.  *Trichuris trichiura* | Pelvic soil | [145] |
|  | Mikelauen-Zilo | 10^th^–14^th^ c. CE | *Dicrocoelium* sp. | Occupation sediment | [50] |
|  | Charavine | 11^th^ c. CE | *Dicrocoelium* sp. | Coprolite | [147] |
|  | Pineuilh | 11^th^–13^th^ c. CE | *Entamoeba histolytica Giardia duodenalis* | Occupation sediment | [148] |
|  | Grand Louvre, Paris | 11^th^–16^th^ c. CE | *Ascaris lumbricoides* *Dicrocoelium dendriticum* *Trichuris trichiura* | Latrine | [149] |
|  | Ile de la Cité | 12^th^–15^th^ c. CE | *Dicrocoelium dendriticum Fasciola hepatica*  *Taenia* sp.  *Trichuris* sp. | Cesspit | [150] |
|  | Epinal | 13^th^ c. CE | *Dicrocoelium* sp. | Latrine | [50] |
|  | Villiers-le-Bel | 14^th^ c. CE | *Dicrocoelium* sp.  *Entamoeba histolytica* | Tomb sediment | [50,78] |
|  | Montbéliard | 1450–1550 CE | *Ascaris lumbricoides* *Dicrocoelium* sp. *Dibothriocephalus latum* *Schistosoma haematobium* *Schistosoma mansoni* *Trichuris trichiura* | Latrine | [50,149–151] |
|  | Rue Cardinal Lemoine, Paris | 15^th^ c. CE | *Dicrocoelium* sp. | Latrine | [50] |
|  | Rigny | 15^th^–16^th^ c. CE | *Ascaris* sp.  *Dicrocoelium sp.*  *Trichuris* sp. | Latrine | [153] |
|  | Marly-le-Roy | 1680–1715 CE | *Ascaris lumbricoides* *Fasciola hepatica*  *Taenia* sp.  *Trichuris trichiura* | Latrine | [152] |
|  | Brouage | 17^th^ c. CE | *Entamoeba histolytica* | Latrine | [153,154] |
| Germany | Lübeck | 1100–1650 CE | *Ascaris* sp. *Dibothriocephalus latum* *Taenia saginata*  *Trichuris trichiura* | Latrine | [155] |
|  | Ellwangen-Jagst | 1400–1600 CE | *Ascaris* sp.  *Trichuris trichiura* | Pelvic soil | [154] |
| Greece | Phanagoria | 8^th^–9^th^ c. CE | *Ascaris* sp.  Diphyllobothriidae  Opisthorchiidae  *Taenia sp.*  *Trichuris* sp. | Drains | [157] |
| Israel/ Palestine | Acre | 1190–1291 CE | *Ascaris lumbricoides*  *Dibothriocephalus latum*  *Entamoeba histolytica* *Giardia duodenalis*  *Taenia* sp.  *Trichuris trichiura* | Latrine | [155–157] |
|  | Jerusalem | 1450–1550 CE | *Ascaris lumbricoides*  *Dibothriocephalus* sp.  *Entamoeba histolytica*  *Giardia duodenalis*  *Taenia* sp.  *Trichuris trichiura* | Coprolites and cesspit | [156,157] |
| Italy | Uffizi Gallery, Florence | 300–500 CE | *Ascaris* sp.  *Dicrocoelium dendriticum*  *Trichuris trichiura* | Pelvic soil | [158] |
|  | Selvicciola | 300–800 CE | *Ascaris* sp.  *Taenia* sp. | Pelvic soil | [114] |
|  | Piazza Garibaldi | 10^th^–11^th^ c. CE | *Ascaris* sp.  *Capillaria* sp.  *Dicrocoelium* sp.  *Dibothriocephalus* sp.  *Taenia*/*Echinococcus* sp.  *Trichuris trichiura* | Cesspit | [161] |
| Jordan | Gerasa | 650–750 CE | *Ascaris lumbricoides* | Latrine | [162] |
| Netherlands | Leiden | 1250–1525 CE | *Ascaris* sp.  *Dicrocoelium* sp.  Diphyllobothriidae  *Echinostoma* sp.  *Fasciola* sp.  *Giardia duodenalis*  *Trichuris* sp. | Cesspit | [163] |
|  | Utrecht | 13^th^–14^th^ c. CE | *Ascaris lumbricoides Trichuris trichiura* | Cesspit | [161] |
|  | Kampen | 1350–1425 CE | *Ascaris lumbricoides* *Dibothriocephalus* sp.  *Enterobius vermicularis*  *Taenia* sp.  *Toxocara* sp.  *Trichuris trichiura* | Latrine | [164] |
| Portugal | Santarém | 9^th^-12^th^ c. CE | *Ascaris lumbricoides*  *Trichuris trichiura* | Pelvic soil | [165] |
| Spain | Camesa-Rebolledo | 6^th^–7^th^ c. CE | *Dicrocoelium* sp. | Pelvic soil | [166] |
|  | Córdoba | 10^th^–11^th^ c. CE | *Ascaris* sp. | Latrine | [167] |
|  | Collegiate-Basilica of St. Isidoro | 10^th^–13^th^ c. CE | *Ascaris lumbricoides*  *Trichuris trichiura* | Mummy | [166,167] |
| Sudan | Sedeinga | 300–1500 CE | *Ascaris lumbricoides* *Enterobius vermicularis* *Hymenolepis nana*  *Schistosoma* sp.  *Trichuris trichiura* | Pelvic soil | [168] |
|  | Wadi Halfa | 350–550 CE | *Schistosoma mansoni* | Mummy | [169,170] |
|  | Kulubnarti | 550–1500 CE | *Leishmania donovani* *Schistosoma mansoni* | Mummy | [168] |
|  | Sai Island | 1500 CE | *Hymenolepis* sp.  *Trichuris trichiura* | Pelvic soil | [58] |
| Switzerland | Chevenez | 7^th^–9^th^ c. CE | *Entamoeba histolytica* *Giardia duodenalis* | Pelvic soil | [171] |
|  | Mummy | Medieval | *Echinococcus granulosus* | Mummy | [170] |

# References

1. Bayliss A, Brock F, Farid S, Hodder I, Southon J, Taylor RE. Getting to the bottom of it all: A Bayesian approach to dating the start of Çatalhöyük. Journal of World Prehistory. 2015;28: 1–26.

2. Hodder I. Çatalhöyük: the leopard changes its spots. A summary of recent work. Anatolian Studies. 2014;64: 1–22.

3. Shillito L-M, Matthews W, Almond MJ, Bull ID. The microstratigraphy of middens: capturing daily routine in rubbish at Neolithic Çatalhöyük, Turkey. Antiquity. 2011;85: 1024–1038.

4. Ledger ML, Anastasiou E, Shillito L-M, Mackay H, Bull ID, Haddow SD, et al. Parasite infection at the early farming community of Çatalhöyük. Antiquity. 2019;93: 573–587.

5. Stein GJ. Tell Zeidan 2010-2011 Annual Report. Oriental Institute; 2011. Available: https://isac.uchicago.edu/sites/default/files/uploads/shared/docs/ar/11-20/10-11/10_11_Tell_Zeidan.pdf

6. Anastasiou E, Lorentz KO, Stein GJ, Mitchell PD. Prehistoric schistosomiasis parasite found in the Middle East. Lancet Infectious Diseases. 2014;14: 553–554.

7. Pearson MP, Cleal R, Marshall P, Needham S, Pollard J, Richards C, et al. The age of Stonehenge. Antiquity. 2007;81: 617–639.

8. Craig OE, Shillito L-M, Albarella U, Viner-Daniels S, Chan B, Cleal R, et al. Feeding Stonehenge: cuisine and consumption at the Late Neolithic site of Durrington Walls. Antiquity. 2015;89: 1096–1109.

9. Pearson MP. Stonehenge: Exploring the Greatest Stone Age Mystery. London: Simon and Schuster; 2012.

10. Mitchell PD, Anastasiou E, Whelton HL, Bull ID, Pearson MP, Shillito L-M. Intestinal parasites in the Neolithic population who built Stonehenge (Durrington Walls, 2500 BCE). Parasitology. 2022;149: 1027–1033.

11. Knight M, Ballantyne R, Brudenell M, Cooper A, Gibson D, Robinson Zeki I, editors. Must Farm pile-dwelling settlement: Volume 1. Landscape, architecture and occupation. Cambridge, England: McDonald Institute for Archaeological Research; 2024.

12. French CAI. Geoarchaeology in action: studies in soil micromorphology and landscape evolution. London: Routledge; 2003.

13. Knight M, Ballantyne R, Zeki IR, Gibson D. The Must Farm pile-dwelling settlement. Antiquity. 2019;93: 645–663.

14. Ledger ML, Grimshaw E, Fairey M, Whelton HL, Bull ID, Ballantyne R, et al. Intestinal parasites at the Late Bronze Age settlement of Must Farm, in the fens of East Anglia, UK (9th century B.C.E.). Parasitology. 2019;146: 1583–1594.

15. Defgnée A, Henrotay D, Collette O. Un atelier de foulons gallo-romains à Arlon (province du Luxembourg, Belgique). Analyses chimiques et archéobotaniques du contenu de cuves. Les Nouvelles de L’Archéologie. 2008;114: 47–52.

16. Pigière F, Henrotay D. Camels in the northern provinces of the Roman Empire. J Archaeol Sci. 2012;39: 1531–1539.

17. Small A. Vagnari: Il Villaggio, L’artigianato, La Proprietà Imperiale. Bari: Edipuglia; 2011.

18. Carroll M, editor. The Making of a Roman Imperial Estate: Archaeology in the *Vicus* at Vagnari, Puglia. Oxford: Archaeopress; 2022.

19. Bloy D, Masci G, Rice C, Franconi T, Farney GD, Notarian M. The Upper Sabina Tiberina Project. I risultati della seconda campagna di scavo a Vacone (Rieti). Lazio e Sabina. 2017;11: 57–63.

20. Franconi TV, Rice CM, Bloy D, Farney GD. Excavations at the Roman villa of Vacone (Lazio) by the Upper Sabina Tiberina Project, 2012-2018. In: Sfameni C, Volpi M, editors. Oltre la Villa: Richerche nei siti archeologici del territorio di Cottanello, Montasola, Configni e Vacone. Arbor Sapiente; 2019. pp. 109–136.

21. Golubović S, Mrdić N. Territory of Roman Viminacium from Celtic to Slavic tribes. Proceedings of the International Conference the Phenomena of Cultural Borders and Border Cultures Across the Passage of Time. Trnava: Universitas Tyrnaviensis; 2010. pp. 117–127.

22. Wilkes JJ. The Roman Danube: An archaeological survey. The Journal of Roman Studies. 2005;95: 124–225.

23. Greenwalt CH Jr. Introduction. In: Cahill ND, editor. The Lydians and Their World. Istanbul: Yapı Kredi Yayınları; 2010. pp. 1–36.

24. Rautman M. Sardis in Late Antiquity. In: Dally O, Ratté C, editors. Archaeology and the cities of Asia Minor in Late Antiquity. Ann Arbor: Kelsey Museum of Archaeology; 2011. pp. 1–26.

25. Poblome J, Torun E, Talloen P, Waelkens M, editors. Meanwhile in the Mountains: Sagalassos. Istanbul: Yapi Kredi Yayinlari Sanat; 2019.

26. Beaujean B, Claeys J, Doperé F, Poblome J. A chronological and functional assessment of the Roman Imperial Bath-Gymnasium of Sagalassos (SW Asia minor). Istanbuler Mitteilungen. 2023;73: 146–217.

27. Baeten J, Marinova E, De Laet V, Degryse P, De Vos D, Waelkens M. Faecal biomarker and archaeobotanical analyses of sediments from a public latrine shed new light on ruralisation in Sagalassos, Turkey. J Archaeol Sci. 2012;39: 1143–1159.

28. Scherrer P. The historical topography of Ephesos. J Rom archaeol. 2001;Supplement 45: 57–87.

29. Ladstätter S, Büyükkolancı M, Topal C, Aktüre Z. Ephesus. Ankara: Turkish National Commission for UNESCO; 2016.

30. Jansen G. The toilets of Ephesus. A preliminary report. In: Wiplinger G, editor. Cura Aquarum in Ephesus. Leuven: Peeters; 2006. pp. 95–98.

31. Ladstätter S. Terrace House 2 in Ephesos: An Archaeological Guide. Istanbul: Homer Kitabevi; 2013.

32. Ployer R. Archäologischer befund und funde. In: Rathmayr E, editor. *Hanghaus 2 in Ephesos Die wohneinheit 7: Baubefund, ausstattung, funde Forschungen in Ephesos 8/10*. Vienna, Austria: Verlag der österreichischen Akademie der Wissenschaften; 2016. pp. 285–309.

33. Gómez Martínez S. New perspectives in the study of Al-andalus ceramics, Mértola (Portugal) and the Mediterranean maritime routes in the Islamic period. Al-Masāq. 2009;21: 59–82.

34. Bottaini C, Martínez SG, Bordalo R, Beltrame M, Mirão J, Rafael L, et al. Islamic copper-based metal artefacts from the Garb al-Andalus. A multidisciplinary approach on the Alcáçova of Mārtulah (Mértola, South of Portugal). Heritage Science. 2022;10: 97.

35. Yeh H-Y, Prag K, Clamer C, Humbert J-B, Mitchell PD. Human intestinal parasites from a Mamluk Period cesspool in the Christian quarter of Jerusalem: Potential indicators of long distance travel in the 15th century AD. International Journal of Paleopathology. 2015;9: 69–75.

36. Dabney J, Knapp M, Glocke I, Gansauge M-T, Weihmann A, Nickel B, et al. Complete mitochondrial genome sequence of a Middle Pleistocene cave bear reconstructed from ultrashort DNA fragments. Proc Natl Acad Sci U S A. 2013;110: 15758–15763.

37. Murchie TJ, Kuch M, Duggan AT, Ledger ML, Roche K, Klunk J, et al. Optimizing extraction and targeted capture of ancient environmental DNA for reconstructing past environments using the PalaeoChip Arctic-1.0 bait-set. Quat Res. 2021;99: 305–328.

38. Meyer M, Kircher M. Illumina sequencing library preparation for highly multiplexed target capture and sequencing. Cold Spring Harb Protoc. 2010;2010: db.prot5448.

39. Kircher M, Sawyer S, Meyer M. Double indexing overcomes inaccuracies in multiplex sequencing on the Illumina platform. Nucleic Acids Res. 2012;40: e3.

40. Renaud G, Stenzel U, Kelso J. leeHom: adaptor trimming and merging for Illumina sequencing reads. Nucleic Acids Res. 2014;42: e141.

41. Huson DH, Beier S, Flade I, Górska A, El-Hadidi M, Mitra S, et al. MEGAN community edition - interactive exploration and analysis of large-scale microbiome sequencing data. PLoS Comput Biol. 2016;12: e1004957.

42. Jónsson H, Ginolhac A, Schubert M, Johnson PLF, Orlando L. mapDamage2.0: fast approximate Bayesian estimates of ancient DNA damage parameters. Bioinformatics. 2013;29: 1682–1684.

43. Katoh K, Standley D. MAFFT multiple sequence alignment software version 7: improvements in performance and usability. Mol Biol Evol. 2013;30: 772–780.

44. Felsenstein J. Phylip – phylogeny inference package (version 3.2). Cladistics. 5: 164–166.

45. Dickson ZW, Hackenberger D, Kuch M, Marzok A, Banerjee A, Rossi L, et al. Probe design for simultaneous, targeted capture of diverse metagenomic targets. Cell Rep Methods. 2021;1: 100069.

46. Parizadeh M, Arrieta M-C. The global human gut microbiome: genes, lifestyles, and diet. Trends Mol Med. 2023;29: 789–801.

47. Aspöck H, Barth FE, Flamm H, Picher O. Parasitäre Erkrankungen des Verdauungstraktes bei prähistorischen Bergleuten von Hallstatt und Hallein (Österreich). Mitteilungen der Anthropoloischen Gesellschaft in Wien. 1973;103: 41–47.

48. Aspöck H, Flamm H, Picher O. ‘Darmparasiten in men-schlichen exkrementen aus prähistorischen salzbergwerken der Hallstatt-Kultur (800–350 v. Chr). Zentralblatt für Bakteriologic und Hygiene Abt Originale A. 1973;223: 549–558.

49. Aspöck H, Auer H, Picher O. Parasites and parasitic diseases in prehistoric human populations in Central Europe. Helminthologia. 1999;36: 139–145.

50. Le Bailly M, Bouchet F. Ancient dicrocoeliosis: occurrence, distribution and migration. Acta Trop. 2010;115: 175–180.

51. Bell M. Brean Down: Excavations 1983-1987. England: English Heritage; 1990.

52. Ryan H, Flammer PG, Nicholson R, Loe L, Reeves B, Allison E, et al. Reconstructing the history of helminth prevalence in the UK. PLoS Negl Trop Dis. 2022;16: e0010312.

53. Harter-Lailheugue S, Le Mort F, Vigne J-D, Guilaine J, Le Brun A, Bouchet F. Premiéres Données Parasitologiques sur les populations humaines précéramiques chypriotes (VIII ^e^ et VII ^e^ millénaires av. J.-C.). Paléorient. 2005;31: 43–54.

54. Šebela L, Vojtkova L, Vojtek J. Intestinal Parasites in Man of Old Bronze Age. Anthropologie. 1990;28: 105–107.

55. Reyman TA, Zimmerman MR, Lewin PK. Autopsy of an Egyptian mummy (Nakht-ROM I). Canadian Medical Association Journal. 1977;117: 470–471.

56. de Boni U, Lenczner MM, Scott JW. Autopsy of an Egyptian mummy. 6. Trichinella spiralic cyst. Can Med Assoc J. 1977;117: 472.

57. Deelder AM, Miller RL, De Jonge N, Krijger FW. Detection of Schistosome Antigen in Mummies. Lancet. 1990;335: 724–725.

58. Tapp E. Disease and the Manchester Mummies: The Pathologist’s Role. In: David R, Tapp E, editors. Evidence Embalmed. Manchester: Manchester University Press; 1984. pp. 78–95.

59. Harter S. Implication de la paléoparasitologie dans l’étude des populations anciennes de la vallée du Nil et de proche-orient : Étude de cas. PhD, Université de Reims - Champagne Ardenne. 2003.

60. Bruschi F, Masetti M, Locci MT, Ciranni R, Fornaciari G. Short report: cysticercosis in an Egyptian mummy of the late Ptolemaic period. Am J Trop Med Hyg. 2006;74: 598–599.

61. Cockburn A, Barraco RA, Reyman TA, Peck WH. Autopsy of an Egyptian mummy. Science. 1975;187: 1155–1160.

62. Dommelier-Espejo S. Contribution à l’Etude Paléoparasitologique des Sites Néolithiques en Environnement Lacustre dans les Domaines Jurassien et Péri- Alpin. Bouchet F, editor. PhD, Université de Reims. 2001.

63. Gonçalves MLC, da Silva VL, de Andrade CM, Reinhard K, da Rocha GC, Le Bailly M, et al. Amoebiasis distribution in the past: first steps using an immunoassay technique. Trans R Soc Trop Med Hyg. 2004;98: 88–91.

64. Le Bailly M, Bouchet F. *Diphyllobothrium* in the past: Review and new records. International Journal of Paleopathology. 2013;3: 182–187.

65. Bouchet F, Petrequin P, Paicheler JC, Dommelier S. Première approche paléoparasitogique du site néolithique de Chalain (Jura, France). Bulletin de la Société de Pathologie Exotique. 1995;88: 265–268.

66. Bouchet F. Intestinal capillariasis in neolithic inhabitants of Chalain (Jura, France). Lancet. 1997;349: 256.

67. Dommelier S, Bentrad S, Bouchet F, Paicheler JC, Pétrequin P. Parasitoses liées à l’alimentation chez les populations du site néolithique de Chalain (Jura, France). Anthropozoologica. 1998;27: 41–49.

68. Le Bailly M. Evolution de la Relation Hôte/Parasite dans les Systèmes Lacustres Nord Alpins au Néolithique (3900–2900 BC), et Nouvelles Données dans la Détection des Paléoantigènes de Protozoa. PhD, Université de Reims Champagne-Ardenne. 2005.

69. Dittmar K, Teegen WR. The presence of *Fasciola hepatica* (Liver-fluke) in humans and cattle from a 4,500 year old archaeological site in the Saale-Unstrut Valley, Germany. Memórias do Instituto Oswaldo Cruz. 2003;98: 141–143.

70. Le Bailly M, Bouchet F. Paléoparasitologie et immunologie. L’exemple d’*Entamoeba histolytica*. ArcheoSciences. 2006;30: 129–135.

71. Anastasiou E, Papathanasiou A, Schepartz LA, Mitchell PD. Infectious disease in the ancient Aegean: Intestinal parasitic worms in the Neolithic to Roman Period inhabitants of Kea, Greece. Journal of Archaeological Science: Reports. 2018;17: 860–864.

72. Langgut D. Mid-7th century BC human parasite remains from Jerusalem. Int J Paleopathol. 2022;36: 1–6.

73. Cahill J, Reinhard K, Tarler D, Warnock P. It had to happen: Scientists examine remains of ancient bathroom. Biblical Archaeology Review. 1991;17: 64–69.

74. Aspöck H, Auer H, Picher O. *Trichuris trichiura* eggs in the neolithic glacier mummy from the Alps. Parasitol Today. 1996;12: 255–256.

75. Roever-Bonnet H, Rijpstra C, van Renesse MA, Peen CH. Helminth eggs and gregarines from coprolites from the excavations at Swifterbant. Helinium. 1979;19: 7–12.

76. Jansen J, Boersema JH. Helminth eggs from the latrines of the Olofskapel Gatehouse, Amsterdam. Paleopathol Newsl. 1972;2: 7–8.

77. Maicher C, Hoffmann A, Côté NML, Palomo Pérez A, Saña Segui M, Le Bailly M. Paleoparasitological investigations on the Neolithic lakeside settlement of La Draga (Lake Banyoles, Spain). Holocene. 2017;27: 1659–1668.

78. Le Bailly M, Bouchet F. A first attempt to retrace the history of dysentery caused by *Entamoeba histolytica*. In: Mitchell PD, editor. Sanitation, latrines and intestinal parasites in past populations. Farnham: Ashgate; 2015. pp. 219–228.

79. Le Bailly M, Leuzinger U, Bouchet F. Dioctophymidae eggs in coprolites from Neolithic site of Arbon-Bleiche 3 (Switzerland). J Parasitol. 2003;89: 1073–1076.

80. Flammer PG, Dellicour S, Preston SG, Rieger D, Warren S, Tan CKW, et al. Molecular archaeoparasitology identifies cultural changes in the Medieval Hanseatic trading centre of Lübeck. Proc R Soc B. 2018;285: 20180991.

81. Maicher C, Bleicher N, Le Bailly M. Spatializing data in paleoparasitology: Application to the study of the Neolithic lakeside settlement of Zürich-Parkhaus-Opéra, Switzerland. Holocene. 2019;29: 1198–1205.

82. Aspöck H, Feuereis I, Radbauer S. Case study: Detection of eggs of the intestinal parasite *Ascaris lumbricoides* in samples from the Roman sewers of Carnuntum. In: Jansen GCM, Koloski-Ostrow AO, Moormann EM, editors. Roman toilets: Their archaeology and cultural history. Leuven: Peeters; 2011. pp. 163–164.

83. Petznek B. A Roman cesspit from the mid-2nd century with lead price tags in the civil town of Carnuntum (Schloss Petronell/Austria). In: Hoss S, editor. Latrinae: Roman toilets in the northwestern provinces of the Roman Empire. Oxford: Archaeopress; 2018. pp. 119–126.

84. Gourevitch D, Le Bailly M, Dufour B, Bouchet F, Defgnée A, Henrotay D, et al. Hygiène, Santé et Parasites. In: Gourevitch D, editor. Pour une Archéologie de la Médecine Romaine. Paris: De Boccard; 2011. pp. 67–86.

85. Boyer P. The Parasites. In: Connor A, Buckley R, editors. Roman and Medieval Occupation of Causeway Lane, Leicester Excavations 1980 and 1991. Leicester: University of Leicester Archaeological Services; 1999. pp. 344–346.

86. Rouffignac C. Parasite egg survival and identification from Hibernia Wharf, Southwark. London Archaeologist. 1985;5: 103–105.

87. Wells C, Dallas C. Romano-British Pathology. Antiquity. 1976;50: 53–56.

88. Jones AKG, Hutchinson AR. The parasitological evidence. In: McCarthy MR, editor. The structural sequence and environmental remains from Castle Street, Carlisle: Excavations 1981–2. Kendal: Cumberland and Westmorland Antiquarian and Archaeological Society; 1991. pp. 68–72.

89. Jones AKG. Parasitological investigations on the Ambleside Roman pit. Historic England; 1985. Report No.: 4600.

90. Wilson A, Rackham DJ. Parasite Eggs. In: Buckland PC, editor. The Environmental Evidence from the Church Street Roman Sewer System. York, UK: York Archaeological Trust; 1976. pp. 32–33.

91. de Moulins D. Environmental analysis. In: Maloney C, de Moulins D, editors. The archaeology of Roman London volume I: The Upper Walbrook in the Roman Period. London: The Museum of London; 1990. pp. 85–115.

92. Knights BA, Dickson CA, Dickson JH, Breeze DJ. Evidence concerning the roman military diet at Bearsden, Scotland, in the 2nd Century AD. J Archaeol Sci. 1983;10: 139–152.

93. Carrott J, Issitt M, Kenward H, Large F, McKenna B, Skidmore P. Insect and other invertebrate remains from excavations at four sites in Lincoln (site codes: WN87, WNW88, WF89 and WO89): Technical report. Environmental Archaeology Unit, York; 1995. Report No.: 95/10.

94. Pike AW. Recovery of helminth eggs from archaeological excavations, and their possible usefulness in providing evidence for the purpose of an occupation. Nature. 1968;219: 303–304.

95. Jones AKG. Parasitological Investigations on Samples of Organic Material Associated with Human Burials at the Roman Inhumation Cemetery at Poundbury, Dorset (site code PC72-76). York, UK: Historic Buildings and Monuments Commission for England; 1987.

96. Horne PD. First evidence of enterobiasis in ancient Egypt. J Parasitol. 2002;88: 1019–1021.

97. Le Bailly M, Mouze S, da Rocha GC, Heim J-L, Lichtenberg R, Dunand F, et al. Identification of *Taenia* sp. in a mummy from a Christian Necropolis in El-Deir, Oasis of Kharga, ancient Egypt. J Parasitol. 2010;96: 213–215.

98. Rousset J-J, Heron C, Metrot P. Human helminthiasis at the Gauls. Histoire des Sciences Medicales. 1996;30: 41–46.

99. Harter-Lailheugue S. Étude paléoparasitologique du site de Marseille Quai Rive Neuve. In: Bien S, Richier A, editors. Rapport final d’Opération, Fouilles Archéologiques, 23, Quai de Rive Neuve à Marseille (Bouches-du-Rhône). Inrap Méditerranée; 2006. pp. 91–95.

100. Dufour B. Synthèse de données et nouvelle contribution à l’étude des parasites de l’époque romaine, et apports méthodologiques de l’extraction des marqueurs au traitement des résultats. Le Bailly M, editor. PhD, Université de Franche-Comte. 2015.

101. Sireix C. Origine et développment d’un quartier suburbain antique de Burdigala. In: Sireix C, editor. La Cité judiciaire un quartier suburbain de Bordeaux antique. Bordeaux: Aquitania; 2008. pp. 11–80.

102. Bouchet, F., Bentrad, S., Martin, C. Le quartier Gallo-romain de la rue de Venise à Reims, Etude paléoparasitologique. Bulletin de la Société Archéologique Champenoise. 2001;2/3: 148–150.

103. Le Bailly M, Harter S, Bouchet F. La Paléoparasitologie, à l’interface de l’archéologie et de la biologie. Archéopages. 2003;11: 12–17.

104. Dufour B, Segard M, Le Bailly M. A first case of human trichuriasis from a Roman lead coffin in France. Korean Journal of Parasitology. 2016;54: 625–629.

105. Mowlavi G, Kacki S, Dupouy-Camet J, Mobedi I, Makki M, Harandi MF, et al. Probable hepatic capillariosis and hydatidosis in an adolescent from the late Roman period buried in Amiens (France). Parasite. 2014;21: 9.

106. Goppelsröder A, Sommer CS. Die organischen Reste einer römerzeitlichen Latrinenverfüllung und anderer Befunde in Ladenburg, Merkurplatz 5. Fundberichte aus Baden-Württemberg. 1996;21: 401–412.

107. Dittmar K, Teegen W-R, Cordie-Hackenberg R. Nachweis von Eingeweideparasiteneiern in einem Abfallschacht aus dem römischen Vicus von Belginum/Wederath (Rheinland-Pfalz). Archäologisches Korrespondenzblatt. 2002;32: 415–425.

108. Specht KW. Eine interessante erdprobe aus einer abortgrube im Römerkastell Künzing. Saalburg Jahrb. 1963;21: 90–94.

109. Harter S, Bouchet F, Mumcuoglu KY, Zias JE. Toilet practices among members of the Dead Sea Scrolls Sect at Qumran (100 BCE-68 CE). Revue de Qumrân. 2004;21: 579–584.

110. Zias JE, Tabor JD, Harter-Lailheugue S. Toilets at Qumran, the Essenes, and the Scrolls: New anthropological data and old theories. Revue de Qumrân. 2006;22: 631–640.

111. Zias J, Mumcuoglu KY. Calcified Hydatid Cysts. Paleopathol Newsl. 1991;73: 7–8.

112. Witenberg. Human parasites in archaeological findings. In: Digital Library for International Research Archive | Parasites in human saeces in archaeological findings [Internet]. 1961 [cited 12 Oct 2016]. Available: http://www.dlir.org/archive/orc-exhibit/items/show/collection/4/id/2443/tag/Yedi+ot+ha-Hevrah+la-hakirat+Erets-Yisrael+ye+atikoteha+Volume+25

113. Heirbaut E, Jones AKG, Wheeler K. Archeaeometry: Methods and analysis. In: Jansen GCM, Koloski-Ostrow AO, Moormann EM, editors. Roman toilets: Their archaeology and cultural history. Leuven: Peeters; 2011. pp. 7–20.

114. Ledger ML, Micarelli I, Ward D, Prowse TL, Carroll M, Killgrove K, et al. Gastrointestinal infection in Italy during the Roman Imperial and Longobard periods: a paleoparasitological analysis of sediment from skeletal remains and sewer drains. International Journal of Paleopathology. 2021;33: 61–71.

115. Rabinow S, Wang T, Wilson RJA, Mitchell PD. Using parasite analysis to identify ancient chamber pots: An example of the fifth century CE from Gerace, Sicily, Italy. Journal of Archaeological Science: Reports. 2022; 103349.

116. Van Geel B, Buurman J, Brinkkemper O, Schelvis J, Aptroot A, van Reenen G, et al. Environmental reconstruction of a Roman Period settlement site in Uitgeest (The Netherlands), with special reference to coprophilous fungi. Journal of Archaeological Science. 2003;30: 873–883.

117. Kuijper WJ, Turner H. Diet of a Roman centurion at Alphen aan den Rijn, The Netherlands, in the first century AD. Review of Palaeobotany and Palynology. 1992;73: 187–204.

118. Jansen J, Over HJ. Observations on Helminth Infections in a Roman Army Camp. In: Corradetti A, editor. Proceedings of the 1st International Congress of Parasitology, Roma, Italy, 1964. Oxford, UK: Pergamon; 1966. p. 791.

119. Hänggi R, Zumstein A, Endriss Y. Augusta Rauricorum, Insula 22: Grabungs-und Dokumentationsstand 1988. Jahresberichte aus Augst and Kaiseraugst. 1989;10: 29–72.

120. Hufschmid T, Sütterlin H. Zu einem Lehmfachwerkbau und zwei Latrinengruben des 1. Jahrhunderts in Augst: Ergebnisse der Grabung 1991.65 im Gebiet der Insulae 51 und 53. Jahresberichte aus Augst und Kaiseraugst. 1992;13: 129–176.

121. Jauch V. Eschenz-Tasgetium Römische Abwasserkanäle und Latrinen. Frauenfeld: Hubert & Co. AG; 1997.

122. Le Bailly M, Harter S, Da Rocha GC, Bouchet F. Compte-rendu de l’étude Paléoparasitologique de Windisch-Breite 1996-1998. In: Hagendorn A, Doppler HW, Huber A, Plogmann HH, Jacomet S, Meyer-Freuler C, et al., editors. Zur Frühzeit von Vindonissa, Auswertung der Holzbauten der Grabung Windisch-Breite 1996-1998. Veröffentlichungen der Gesellschaft pro Vindonissa; 2003. p. 230.

123. Williams FS, Arnold-Foster T, Yeh H-Y, Ledger ML, Baeten J, Poblome J, et al. Intestinal parasites from the 2nd-5th century AD latrine in the Roman Baths at Sagalassos (Turkey). Int J Paleopathol. 2017;19: 37–42.

124. Auer H, Aspöck H. Helminths and helminthoses in Central Europe: diseases caused by nematodes (roundworms). Wien Med Wochenschr. 2014;164: 424–434.

125. da Rocha GC, Lailheugue SH-, Le Bailly M, Arajo A, Ferreira LF, da Serra-Freire NM, et al. Paleoparasitological remains revealed by seven historic contexts from “Place D’Armes”, Namur, Belgium. Memórias do Instituto Oswaldo Cruz. 2006;101: 43–52.

126. Rácz SE, Araújo EP, Jensen E, Mostek C, Morrow JJ, Van Hove ML, et al. Parasitology in an archaeological context: analysis of medieval burials in Nivelles, Belgium. J Archaeol Sci. 2015;53: 304–315.

127. Rabinow S, Deforce K, Mitchell PD. Continuity in intestinal parasite infection in Aalst (Belgium) from the medieval to the early modern period (12th-17th centuries). Int J Paleopathol. 2023;41: 43–49.

128. Graff A, Bennion-Pedley E, Jones AK, Ledger ML, Deforce K, Degraeve A, et al. A comparative study of parasites in three latrines from Medieval and Renaissance Brussels, Belgium (14th-17th centuries). Parasitology. 2020;147: 1443–1451.

129. Wang T, Deforce K, De Gryse J, Eggermont S, Vanoverbeke R, Mitchell PD. Evidence for parasites in burials and cesspits used by the clergy and general population of 13th-18th century Ghent, Belgium. Journal of Archaeological Science: Reports. 2024;53: 104394.

130. Troubleyn L, Kinnaer F, Ervynck A, Beeckmans L, Caluwé D, Cooremans B, et al. Consumption patterns and living conditions inside Het Steen, the late medieval prison of Malines (Mechelen, Belgium). Journal of Archaeology in the Low Countries. 2009;1–2: 5–47.

131. Deforce K. Pollen analysis of 15th century cesspits from the palace of the dukes of Burgundy in Bruges (Belgium): evidence for the use of honey from the western Mediterranean. J Archaeol Sci. 2010;37: 337–342.

132. Fernandes A, Ferriera LF, Gonçalves MLC, Bouchet F, Klein CH, Iguchi T, et al. Intestinal parasite analysis in organic sediments collected from a 16th-century Belgian archeological site. Cadernos de Saúde Pública, Rio de Janeiro. 2005;21: 329–333.

133. Tibensky K, Sidell J. The Parasite Remains. In: Malcolm G, Bowsher D, Cowie R, editors. Middle Saxon London: Excavations of the Royal Opera House 1989-99. London: Museum of London Archaeology Service; 2003. pp. 333–337.

134. Wang T, Cessford C, Dittmar JM, Inskip S, Jones PM, Mitchell PD. Intestinal parasite infection in the Augustinian friars and general population of medieval Cambridge, UK. International Journal of Paleopathology. 2022;39: 115–121.

135. Hall AR, Jones A, Kenward HK. Cereal Bran and Human Faecal Remains from Archaeological Deposits - Some Preliminary Observations. In: Proudfoot B, editor. Site, Environment and Economy. Oxford: BAR International Series 173; 1983. pp. 85–104.

136. Taylor EL. Parasitic helminths in Mediaeval remains. Vet Rec. 1955;67: 216–218.

137. Pike AW, Biddle M. Parasite Eggs in Medieval Winchester. Antiquity. 1966;40: 293–296.

138. Pike AW. Parasite Eggs. In: Platt C, Coleman-Smith R, editors. Excavations in Medieval Southampton 1953-1969. Leicester: Leicester University Press; 1975. pp. 347–348.

139. Jones AKG, Nicholson C. Recent finds of *Trichuris* and *Ascaris* ova from Britain. Paleopathol Newsl. 1988;62: 5–6.

140. Greig J. The investigation of a medieval barrel-latrine from Worcester. J Archaeol Sci. 1981;8: 265–282.

141. Mitchell PD, Yeh H-Y, Appleby J, Buckley R. The intestinal parasites of King Richard III. Lancet. 2013;382: 888.

142. Anastasiou E, Mitchell PD. Human intestinal parasites from a latrine in the 12th century Frankish castle of Saranda Kolones in Cyprus. International Journal of Paleopathology. 2013;3: 218–223.

143. Bartošová L, Ditrich O, Beneš J, Frolík J, Musil J. Paleoparasitological findings in Medieval and early modern archaeological deposits from Hradbni Street, Chrudim, Czech Republic. Interdisciplinaria Archaeologica. 2011;2: 27–38.

144. Dufour B, Portat E, Bazin B, Le Bailly M. Paleoparasitology of Merovingian Corpses buried in stone sarcophagi in the Saint-Martin-au-Val Church (Chartres, France). Korean J Parasitol. 2019;57: 613–619.

145. Bouchet F, Lavazec C, Nattier V, Dommelier S, Bentrad S, Paicheler J-C. Étude de la parasitofaune du site médiéval de Charavines (Lac du Paladru, Isère, France). Bull Soc Zool Fr. 2000;125: 205–215.

146. Le Bailly M, Gonçalves ML, Harter-Lailheugue S, Prodéo F, Araujo A, Bouchet F. New finding of *Giardia intestinalis* (Eukaryote, Metamonad) in Old World archaeological site using immunofluorescence and enzyme-linked immunosorbent assays. Mem Inst Oswaldo Cruz. 2008;103: 298–300.

147. Bouchet F. Recovery of helminth eggs from archeological excavations of the Grand Louvre (Paris, France). J Parasitol. 1995;81: 785–787.

148. Bouchet F, Audoin F, Leger N, Marchais R, Baucheron F, La Casta JM, et al. Étude parasitologique des coprolithes et des sédiments de trois ensembles clos médiévaux de la rue de Lutèce (île de la Cité) à Paris. Revue Archéométrie. 1989;13: 13–21.

149. Bouchet, Paicheler. Paléoparasitologie: présomption d’un cas de bilharziose au XVe siècle à Montbéliard (Doubs, France). Comptes rendus de l’Académie des sciences. 1995;318: 811–814.

150. Gonçalves MLC, Araújo A, Ferreira LF. Human intestinal parasites in the past: new findings and a review. Mem Inst Oswaldo Cruz. 2003;98: 103–118.

151. Bouchet F, Harter S, Paicheler JC, Aráujo A, Ferreira LF. First recovery of *Schistosoma mansoni* eggs from a latrine in Europe (15-16th centuries). J Parasitol. 2002;88: 404–405.

152. Bouchet F, Paicheler JC, Bentrad S. Analyse paléoparasitologique du contenu de la latrines F509 (site de Rigny). Revue Archéologique du Centre de la France. 1995;34: 246–247.

153. Bouchet F, Bentrad S, Paicheler J-C. Enquête épidémiologique sur les helminthiases à la cour de Louis XIV. Médecine/Sciences: Mini-Synthese. 1998;14: 463–466.

154. Slepchenko SM, Ostapenko SN, Khrustalev AV. Archaeoparasitological characteristics of drains in the Phanagoria city of the Khazar period (VIII-IX centuries AD). Journal of Archaeological Science: Reports. 2023;47: 103810.

155. Mitchell PD, Tepper Y. Intestinal parasitic worm eggs from a crusader period cesspool in the city of Acre (Israel). Levant. 2007;39: 91–95.

156. Mitchell PD, Stern E, Tepper Y. Dysentery in the crusader kingdom of Jerusalem: an ELISA analysis of two medieval latrines in the City of Acre (Israel). J Archaeol Sci. 2008;35: 1849–1853.

157. Mitchell PD, Anastasiou E, Syon D. Human intestinal parasites in crusader Acre: Evidence for migration with disease in the medieval period. International Journal of Paleopathology. 2011;1: 132–137.

158. Roche K, Capelli N, Pacciani E, Lelli P, Pallecchi P, Bianucci R, et al. Gastrointestinal parasite burden in 4th-5th c. CE Florence highlighted by microscopy and paleogenetics. Infect Genet Evol. 2021;90: 104713.

159. Bosi G, Mazzanti MB, Florenzano A, N’siala IM, Pederzoli A, Rinaldi R, et al. Seeds/fruits, pollen and parasite remains as evidence of site function: piazza Garibaldi – Parma (N Italy) in Roman and Mediaeval times. J Archaeol Sci. 2011;38: 1621–1633.

160. Florenzano A, Mercuri AM, Pederzoli A, Torri P, Bosi G, Olmi L, et al. The significance of intestinal parasite remains in pollen samples from Medieval pits in the Piazza Garibaldi of Parma, Emilia Romagna, Northern Italy. Geoarchaeology. 2012;27: 34–47.

161. Søe MJ, Nejsum P, Seersholm FV, Fredensborg BL, Habraken R, Haase K, et al. Ancient DNA from latrines in Northern Europe and the Middle East (500 BC-1700 AD) reveals past parasites and diet. PLOS One. 2018;13: e0195481.

162. Rabinow S, Wang T, van Oosten R, Meijer Y, Mitchell PD. Intestinal parasite infection and sanitation in medieval Leiden, the Low Countries. Antiquity. 2024; 1–17.

163. Boersema JH, Jansen J. Helminth infections in Medieval Utrecht. Trop Geogr Med. 1975;27: 441.

164. Cunha D, Santos AL, Matias A, Sianto L. A novel approach: combining dental enamel hypoplasia and paleoparasitological analysis in medieval Islamic individuals buried in Santarém (Portugal). Antropologia Portuguesa. 2017;34: 113–135.

165. López-Gijón R, Carnicero S, Botella-López MC, Camarós E. Zoonotic parasite infection from a funerary context: A Late Antique child case from Cantabrian Spain. Int J Paleopathol. 2023;41: 55–58.

166. Knorr DA, Smith WPW, Ledger ML, Peña-Chocarro L, Pérez-Jordà G, Clapés R, et al. Intestinal parasites in six Islamic medieval period latrines from 10th–11th century Córdoba (Spain) and 12th–13th century Mértola (Portugal). International Journal of Paleopathology. 2019;26: 75–83.

167. Hidalgo-Argüello MR, Díez Baños N, Fregeneda Grandes J, Prada Marcos E. Parasitological Analysis of Leonese Royalty from Collegiate-Basilica of St. Isidoro, León. J Parasitol. 2003;89: 738–743.

168. Miller RL, Armelagos GJ, Ikram S, De Jonge N, Krijger FW, Deelder AM. Palaeoepidemiology of *Schistosoma* infection in mummies. BMJ. 1992;304: 555–556.

169. Zink AR, Spigelman M, Schraut B, Greenblatt CL, Nerlich AG, Donoghue HD. Leishmaniasis in ancient Egypt and upper Nubia. Emerging Infectious Diseases. 2006;12: 1616–1617.

170. Hibbs AC, Secor WE, Van Gerven D, Armelagos G. Irrigation and infection: the immunoepidemiology of schistosomiasis in ancient Nubia. American Journal of Physical Anthropology. 2011;145: 290–298.

171. Baud CA, Kramar C. Soft Tissue Calcifications in Paleopathology. In: Ortner DJ, Aufderheide AC, editors. Human Paleopathology: Current Syntheses and Future Options. Washington, D.C.: Smithsonian Institution Press; 1991. pp. 87–89.
