# Supplementary material for: Sedimentary ancient DNA as part of a multimethod paleoparasitology approach reveals temporal trends in human parasitic burden in the Roman period
Source: PLoS Negl Trop Dis. 2025 Jun 10;19(6):e0013135. doi: 10.1371/journal.pntd.0013135 (PMC12151383; doi:10.1371/journal.pntd.0013135)
Supplement: Attachment — Submitted filename: PNTD-D-24-01526_reviewer_AMI_auresp_1.pdf [file pntd.0013135.s005.pdf]

PLOS Neglected Tropical Diseases

Sedimentary ancient DNA as part of a multimethod paleoparasitology approach reveals temporal trends in human parasitic burden in the Roman period

--Manuscript Draft--

**Manuscript Number:**	PNTD-D-24-01526
**Full Title:**	Sedimentary ancient DNA as part of a multimethod paleoparasitology approach reveals temporal trends in human parasitic burden in the Roman period
**Short Title:**	Sedimentary ancient DNA as part of a multimethod paleoparasitology approach
**Article Type:**	Research Article
**Keywords:**	Ancient DNA; sedimentary DNA; ELISA; microscopy; helminths; protozoa; Roman Empire
**Abstract:**	The detection of parasite infections in past populations has classically relied on microscopic analysis of sediment samples and coprolites. In recent years, additional methods have been integrated into paleoparasitology such as, enzyme-linked immunosorbent assay (ELISA) and ancient DNA (aDNA). The aim of this study was to evaluate a multimethod approach for paleoparasitology using microscopy, ELISA, and sedimentary ancient DNA (sedaDNA) with a parasite-specific targeted capture approach and high-throughput sequencing. Using 26 samples dating from c. 6000 BCE to 1500 CE that were previously analyzed with microscopy and ELISA, we aimed to more accurately detect and reconstruct parasite diversity in the Roman Empire and compare this diversity to earlier and later time periods to explore temporal changes in parasite diversity. Microscopy was found to be the most effective technique for identifying the eggs of helminths, with 8 taxa identified. ELISA was the most sensitive for detecting protozoa that cause diarrhea (notably Giardia duodenalis). Parasite DNA was recovered from 9 samples, with no parasite DNA recovered from any pre-Roman sites. Sedimentary DNA analysis identified whipworm at a site where only roundworm was visible on microscopy, and also showed that the whipworm eggs at another site came from two different species (T. trichiura and T. muris). Our results show that a multimethod approach provides the most comprehensive reconstruction of parasite diversity in past populations. In the pre-Roman period, taxonomic diversity included a mixed spectrum of zoonotic and foodborne parasites, together with whipworm, which is spread by ineffective sanitation. We see a marked change during the Roman and medieval periods with an increasing dominance of parasites transmitted by ineffective sanitation, especially roundworm, whipworm and protozoa that cause diarrheal illness.
**Additional Information:**	
**Question**	**Response**
**Government Employee**<br><br>Are you or any of the contributing authors an employee of the United States government?<br><br>Manuscripts authored by one or more US Government employees are not copyrighted, but are licensed under a CC0 Public Domain Dedication, which allows unlimited distribution and reuse of the article for any lawful purpose. This is a legal requirement for US Government employees.	No - No authors are employees of the U.S. government.

Powered by Editorial Manager® and ProduXion Manager® from Aries Systems Corporation

This will be typeset if the manuscript is accepted for publication.	
**Financial Disclosure**<br><br>Enter a financial disclosure statement that describes the sources of funding for the work included in this submission. Review the submission guidelines for detailed requirements. View published research articles from *PLOS Neglected Tropical Diseases* for specific examples.<br><br>This statement is required for submission and **will appear in the published article** if the submission is accepted. Please make sure it is accurate.<br><br>**Funded studies**<br>Enter a statement with the following details:<br>• Initials of the authors who received each award<br>• Grant numbers awarded to each author<br>• The full name of each funder<br>• URL of each funder website<br>• Did the sponsors or funders play any role in the study design, data collection and analysis, decision to publish, or preparation of the manuscript?<br><br>Did you receive funding for this work?	Yes
Please add funding details.<br>as follow-up to "**Financial Disclosure**<br><br>Enter a financial disclosure statement that describes the sources of funding for the work included in this submission. Review the submission guidelines for detailed requirements. View published research articles from *PLOS Neglected Tropical Diseases* for specific examples.<br><br>This statement is required for submission and **will appear in the published article** if the submission is accepted. Please make sure it is accurate.	This work was supported by The Social Sciences and Humanities Research Council of Canada [752-2016-2085], a Tidmarsh Cambridge Scholarship from the Cambridge Commonwealth, European and International Trust and Trinity Hall College, and the Society for the Promotion of Roman Studies to MLL.<br>TJM and HNP were funded by the CANA Foundation, the Garfield Weston Foundation, the Social Sciences and Humanities Research Council of Canada, CIFAR, and Natural Sciences and Engineering Research Council of Canada.

Powered by Editorial Manager® and ProduXion Manager® from Aries Systems Corporation

**Funded studies**<br>Enter a statement with the following details:<br>• Initials of the authors who received each award<br>• Grant numbers awarded to each author<br>• The full name of each funder<br>• URL of each funder website<br>• Did the sponsors or funders play any role in the study design, data collection and analysis, decision to publish, or preparation of the manuscript?<br><br>Did you receive funding for this work?"	
Please select the country of your main research funder (please select carefully as in some cases this is used in fee calculation).<br>as follow-up to "**Financial Disclosure**<br><br>Enter a financial disclosure statement that describes the sources of funding for the work included in this submission. Review the submission guidelines for detailed requirements. View published research articles from *PLOS Neglected Tropical Diseases* for specific examples.<br><br>This statement is required for submission and **will appear in the published article** if the submission is accepted. Please make sure it is accurate.<br><br>**Funded studies**<br>Enter a statement with the following details:<br>• Initials of the authors who received each award<br>• Grant numbers awarded to each author<br>• The full name of each funder<br>• URL of each funder website<br>• Did the sponsors or funders play any role in the study design, data collection and analysis, decision to publish, or preparation of the manuscript?<br><br>Did you receive funding for this work?"	CANADA - CA
**Competing Interests**	The authors declare no competing interests.

On behalf of all authors, disclose any competing interests that could be perceived to bias this work.<br><br>This statement will be typeset if the manuscript is accepted for publication.<br><br>Review the instructions link below and PLOS NTDs' competing interests policy to determine what information must be disclosed at submission.	
**Data Availability**<br><br>Provide a **Data Availability Statement** in the box below. This statement should detail where the data used in this submission can be accessed. This statement will be typeset if the manuscript is accepted for publication.<br><br>Before publication, authors are required to make all data underlying their findings fully available, without restriction. Review our PLOS Data Policy page for detailed information on this policy. Instructions for writing your Data Availability statement can be accessed via the Instructions link below.	The data that support the findings of this study are publicly available from the NCBI Sequence Read Archive with the submission number SUB14636687

Manuscript

Click here to access/download;Manuscript;Multimethod_Approach_Palaeopar

Sedimentary ancient DNA as part of a multimethod paleoparasitology approach reveals temporal trends in human parasitic burden in the Roman period

Marissa L. Ledger[1,2*#], Tyler J. Murchie[2,3], Zachery Dickson[4], Melanie Kuch[2], Scott D. Haddow[5], Christopher J. Knüsel[6], Gil J. Stein[7], Mike Parker Pearson[8], Rachel Ballantyne[9], Mark Knight[10], Koen Deforce[11,12], Maureen Carroll[13], Candace Rice[14], Tyler Franconi[14], Nataša Šarkić[15], Saša Redžič[16], Erica Rowan[17], Nicholas Cahill[18], Jeroen Poblome[19], Maria de Fátima Palma[20], Helmut Brückner[21], Piers D. Mitchell[1], Hendrik Poinar[2,22,23]

[1] Department of Archaeology, The Henry Wellcome Building, University of Cambridge, Cambridge, UK

[2] McMaster Ancient DNA Centre, Department of Anthropology, McMaster University, Hamilton, Ontario, Canada

[3] Hakai Institute, Heriot Bay, British Columbia, Canada

[4] Department of Biology, McMaster University, Hamilton, Ontario, Canada

[5] Department of Cross-Cultural and Regional Studies, University of Copenhagen, Copenhagen, Denmark

[6] CNRS, MC, PACEA, UMR 5199, Université de Bordeaux, Pessac, France

[7] Institute for the Study of Ancient Cultures (ISAC), University of Chicago, Chicago, Illinois, USA

[8] Institute of Archaeology, University College London, London, UK

[9] McDonald Institute for Archaeological Research, University of Cambridge, Cambridge, UK

[10] Cambridge Archaeological Unit, Department of Archaeology, University of Cambridge, Cambridge, UK

[11] Department of Archaeology, Ghent University, Ghent, Belgium

[12] Royal Belgian Institute of Natural Sciences, Brussels, Belgium

[13] Department of Archaeology, University of York, York, UK

[14] Joukowsky Institute for Archaeology & the Ancient World and Department of Classics, Brown University, Providence, Rhode Island, USA

[15] Aita Bioarchaeology, Barcelona, Spain

[16] Institute of Archaeology, Belgrade, Serbia

[17] Department of Classics, Royal Holloway, University of London, UK

[18] Department of Art History, University of Wisconsin–Madison, Wisconsin, USA

[19] Unit of Archaeology, KU Leuven, Belgium

[20] Campo Arqueológico de Mértola/CEAACP, Mértola, Portugal

[21] Institute of Geography, University of Cologne, Cologne (Köln), Germany

[22] Department of Biochemistry and Biomedical Sciences, McMaster University, Hamilton, Ontario, Canada

[23] Michael G. DeGroote Institute for Infectious Disease Research, McMaster University, Hamilton, Ontario, Canada

[#] Current Address: Department of Pathology and Molecular Medicine, McMaster University, Hamilton, Ontario, Canada

* Corresponding author

E-mail: ledgerm@mcmaster.ca (MLL)

Abstract

The detection of parasite infections in past populations has classically relied on microscopic analysis of sediment samples and coprolites. In recent years, additional methods have been integrated into paleoparasitology such as, enzyme-linked immunosorbent assay (ELISA) and ancient DNA (aDNA). The aim of this study was to evaluate a multimethod approach for paleoparasitology using microscopy, ELISA, and sedimentary ancient DNA (sedaDNA) with a parasite-specific targeted capture approach and high-throughput sequencing. Using 26 samples dating from c. 6000 BCE to 1500 CE that were previously analyzed with microscopy and ELISA, we aimed to more accurately detect and reconstruct parasite diversity in the Roman Empire and compare this diversity to earlier and later time periods to explore temporal changes in parasite diversity. Microscopy was found to be the most effective technique for identifying the eggs of helminths, with 8 taxa identified. ELISA was the most sensitive for detecting protozoa that cause diarrhea (notably *Giardia duodenalis*). Parasite DNA was recovered from 9 samples, with no parasite DNA recovered from any pre-Roman sites. Sedimentary DNA analysis identified whipworm at a site where only roundworm was visible on microscopy, and also showed that the whipworm eggs at another site came from two different species (*T. trichiura* and *T. muris*). Our results show that a multimethod approach provides the most comprehensive reconstruction of parasite diversity in past populations. In the pre-Roman period, taxonomic diversity included a mixed spectrum of zoonotic and foodborne parasites, together with whipworm, which is spread by ineffective sanitation. We see a marked change during the Roman and medieval periods with an increasing dominance of parasites transmitted by ineffective sanitation, especially roundworm, whipworm and protozoa that cause diarrheal illness.

Keywords: Ancient DNA, sedimentary DNA, ELISA, microscopy, helminths, protozoa, Roman Empire

Author Summary

A multimethod approach in paleoparasitology, combining microscopy, ELISA and aDNA, provides a more complete reconstruction of past parasite diversity. Applying this multimethod approach to a diverse set of archaeological sediment samples from 6000 BCE to 1500 CE provided insights into temporal changes in parasite diversity in the human past. Microscopy can be used as an effective screening tool for helminths in paleofecal samples, while ELISA is necessary for detection of protozoa, and sedaDNA using targeted enrichment can identify additional taxa and confirm species identification. Targeted enrichment using a comprehensive parasite bait set allows for detection of ancient human parasites and recovery of ancient parasite DNA from as little as 0.25 g of sediment. This multimethod approach reveals that parasite diversity decreased in the Roman period with a decrease in zoonotic parasites and concurrent increase in fecal-oral parasites, a pattern which is consistent during the medieval period.

Introduction

Ancient DNA (aDNA) has increasingly been used to improve the detection and characterization of ancient human pathogens [1–4]. Paleogenetic studies of ancient human pathogens have aided in the determination of the causative agents of past epidemics and endemic diseases [3,5,6], identified extinct strains of bacteria and viruses [7,8], and reconstructed ancient genomes of these organisms, allowing for the calibration of mutation rates and investigation of evolutionary histories such as changes in virulence genes [9,10]. However, many infectious diseases, including most parasites, cannot be detected using skeletal remains which are commonly used in aDNA studies. Parasites contributed to a significant burden of disease in the past, however our ability to detect and study their evolutionary history has been hampered by difficulty accessing their DNA. or coprolites Paleofeces (preserved fecal material) are the best source for recovering preserved DNA from enteric pathogens including bacteria [11,12], parasites [13–15], and viruses [16,17]. The field of paleoparasitology has a long history of using sediment samples to study enteric parasites in past populations [18]. Samples used in

Have not added as see description below, we generally include coprolites as paleofeces

see response to reviewers for explanation of distinction between paleofeces and coprolites

why the distintion?

paleoparasitology include coprolites (partially mineralized feces), pelvic sediment from burials where the intestines decomposed, and sediment containing high amounts of human fecal material such as the fill of latrines and sewer drains. Using these samples to recover genetic material and human gastrointestinal pathogens opens another route for understanding past human health, disease, and lifeways, and provides insight into a large category of pathogens that cannot be diagnosed using skeletal remains.

The use of genetic methods to detect parasites in both clinical and ancient samples has lagged behind most other pathogens, with the exception of *Plasmodium* or malaria [19–21]. For decades, the main methods used in paleoparasitology were microscopy, and to a smaller extent, enzyme-linked immunosorbent assay (ELISA, an immunological method that can detect antigens from specific organisms of interest). In recent years, the number of studies using aDNA has increased (see [20] for a review; and subsequent studies [12–14,22–28]). However, aDNA analysis contributes only a small minority of the total investigations performed within paleoparasitology.

which decades?

numbers of citation out of order

False. A number of paleogenetic contributions for the study of oxiurids, ascarids, trypanosomatids, etc., in pre-contact and historic archaeological sites from Brasil, France and Argentina were ommited

See response to reviewers for additional citations added and further clarification that we have focused on human parasites. While these studies have been done, paleogenetic work in parasitology is still minimal compared to work on bacteria

Here we present a multimethod approach for recovery of ancient parasites using sediment samples from contexts known to contain fecal material, including latrines, sewers, soil from the pelvic area of skeletons, and coprolites. Our multimethod approach incorporates sedaDNA methods using a targeted enrichment approach for parasite-specific DNA, in conjunction with microscopy and ELISA. The aim of this multimethod approach was to generate a more complete understanding of parasite diversity in Europe and the Eastern Mediterranean during the Roman Empire as compared to pre- and post-Roman periods. We analyzed paleofecal samples originating from archaeological sites temporally spanning the Neolithic through the medieval period. We used sedaDNA extraction methods previously shown to increase aDNA recovery compared to commercial kits [29], paired with targeted enrichment in order to preferentially sequence parasite DNA of interest and avoid the high sequencing costs associated with deep shotgun sequencing for low abundance targets such as pathogenic organisms. This sedaDNA approach was originally designed for palaeoecological reconstructions from permafrost—here we show that this approach can also be used to recover the aDNA of human parasites from archaeological sediments. Similarly, we show that a multimethod

Added

Reference

approach to paleoparasitology results in a more comprehensive taxonomic recovery compared to only utilizing a single method. Applying this multimethod approach to paleofecal samples from the Neolithic through medieval periods reveals trends in parasite infection that demonstrate marked change in the dominant species during the Roman period.

Materials and Methods

Materials

We analyzed archaeological sediments from latrine fill, drain fill, coprolites, and soil from the pelvic area of skeletons. All samples came from human contexts in archaeological sites ranging from the Neolithic period (ca. 7000 BCE) through the medieval period (ca. 1500 CE) in regions that were at one time part of the Roman Empire (see Fig 1). A total of 14 sites were included in the study; four from the pre-Roman period, seven from the Roman period, and three from the post-Roman or medieval period. From some sites multiple samples were studied if available, thus a total of 26 samples were included (see Fig 2). Further information about each site and samples analyzed can be found in S1 Appendix.

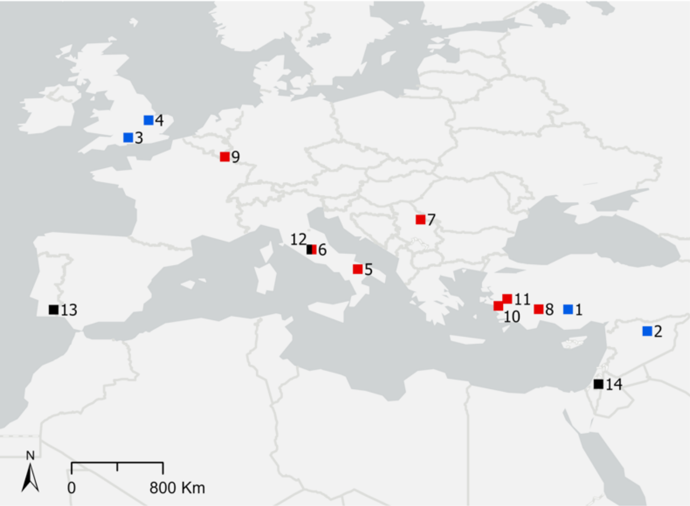

Time Period	Map Label	Site Name	Date	Sample Type
Pre-Roman	1	Çatalhöyük	6410–6150 BCE	Coprolite
	2	Tell Zeidan	4550–4050 BCE	Pelvic soil
	3	Durrington Walls	c. 2500 BCE	Coprolite
	4	Must Farm	865–840 cal BCE	Coprolite, Occupation sediment
Roman	5	Vagnari	1st–4th c. CE	Drain
	6	Vacone	2nd c. BCE– 3rd c. CE	Drain, Latrine
	7	Viminacium	2nd–3rd c. CE	Coprolite
	8	Sagalassos	2nd–5th c. CE	Latrine
	9	Arlon	250–280 CE	Latrine
	10	Ephesus	3rd–6th c. CE	Latrine
	11	Sardis	6th–7th c. CE	Drain
Post-Roman	12	Vacone	7th–8th c. CE	Pelvic soil
	13	Mértola	12th–13th c. CE	Latrine
	14	Jerusalem	15th–16th c. CE	Latrine, Coprolite

Fig 1. Map of archaeological sites studies. Map indicates location of sediment samples studied. The table on the right provides the site name, date of samples, and sample types.

Corrected

Methods

All samples were first studied and reported on using microscopy and ELISA following standard methods [30,31]. The samples selected for this study had previously yielded parasite eggs or came from sites where eggs were found in contextually associated samples. For microscopy, a 0.2 g subsample was disaggregated in 0.5% trisodium phosphate. This was then microsieved to collect material between 20 and 160 µm. This fraction of the subsample was mixed with glycerol and viewed under a light microscope at 200x and 400x magnification to identify preserved helminth eggs based on morphological characteristics.

Reference

Added

Information of microscope

Added

For ELISA, a 1 g subsample was disaggregated in 0.5% trisodium phosphate and microsieved. Given the smaller size of protozoan cysts that cause diarrhea (less than 20 µm), the material in the catchment container below the 20 µm sieve was collected and concentrated to be used in commercial ELISA kits following the manufacturer's protocols. The kits used were *GIARDIA II*™, *E. HISTOLYTICA II*™, and *CRYPTOSPORIDIUM II*™ from TECHLAB, Inc (Blacksburg, USA). These kits are designed for detection of *Giardia duodenalis*, *Entamoeba histolytica*, and *Cryptosporidium* in modern human fecal samples. They employ a monoclonal antibody design and have high sensitivity and specificity in clinical validation studies. These kits and those from other manufacturers have been used to detect protozoan antigens in ancient human fecal samples in many previous studies [31–35].

taxonomic writing

For this comment and similar comment throughout see response to reviewers

For sedaDNA, 0.25 g of material was subsampled in dedicated clean rooms at the McMaster Ancient DNA Centre. Organic material was chemically and physically disintegrated using a chemical digestion buffer paired with garnet PowerBead tubes [29]. The samples were vortexed for 15 minutes to mechanically break down the organo-mineralized content and parasite eggs. Bead beating has been shown in clinical and archaeological studies to improve DNA recovery by breaking parasite eggs [36,37]. Proteinase K was added after bead beating, and tubes were continuously rotated in an oven set to 35° C overnight. The supernatant was removed and mixed with high-volume Dabney binding buffer as per published protocols [38]. Samples were then centrifuged at 4500 rpm at 4° C following Murchie et al. [29] for a minimum of 6 hours to remove inhibitors; if supernatant was clear at this point, the sample was removed from the centrifuge, and if not, it was centrifuged further for a total of 24 hours. Centrifugation

explain

Further detail added to methods and Appendix A to address the following comments on method description.

concentration and manufactures

Found in Supplementary Material with all other concentrations and manufacturers for reagents. We added a note to direct readers to this information at the start of the methods section

at refrigerated temperatures has been shown to increase the recovery of sedaDNA from complex environmental sample types through the precipitation of enzymatic inhibitory compounds commonly found in sediment and fecal samples [29]. Most published parasite DNA studies use modern commercial extraction kits, such as the PowerSoil DNA Extraction Kit, Mo Bio Laboratories [36,39–41] or QIAmp DNA Stool Mini Kit, Qiagen [42]. In our previous work, the sedaDNA methods employed here have been shown to increase DNA recovery from soil samples by 7–20 fold compared to commercial kits [29]. The remainder of the extraction and purification followed Dabney et al. [38] with the binding buffer passed through silica columns and eluted in 50 µL elution buffer with Tween.

no methodology, discussion

Moved

DNA libraries were prepared for Illumina sequencing using a double-stranded method [43,44] with minor modifications for blunt end repair as per our laboratory protocols (see S1 Appendix). A subset of four libraries (MP12, MP26, MP37, and MP53) were indexed separately for shotgun sequencing with a targeted sequencing depth of 2 million reads per sample. Targeted enrichment for parasite DNA was carried out using in-solution hybridization capture with RNA baits designed using modern parasite genomes (see S2 Appendix for reference sequence accession numbers). The parasite bait set contained 30,240-75mer baits based on mitochondrial and nuclear gene sequences retrieved from GenBank NCBI [45,46] from a list of identified human and animal infecting parasites and protozoa. Tiled baits were designed to capture 121 parasite taxa (see S2 Appendix). A progressive tiling approach was used to balance the number of baits for each taxa as best as possible. With this approach the bait design algorithm determined the maximum and minimum tiling density possible for each bait region. Taxa with few bait regions were maximally tiled to increase bait number while taxa with many potential bait regions were minimally tiled to balance bait number per taxa. The final average tiling density per taxa ranged from 2x to 30x. The number of baits for each taxa was ultimately limited by reference sequences available and ranged from 2–922 baits per taxa. Baits were purchased from Daicel Arbor Biosciences and our enrichment protocol followed the MYbaits V4 protocol supplied by the manufacturer. The targeted enrichment approach allows for the potential recovery of any of our targeted 121 taxa (S2 Appendix), which is the largest targeted approach in paleoparasitology to date without relying on shotgun sequencing. Many

kits and equipament usec

Found in following paragraph where sequencing is described, also present in Supplementary material

paleogenetic studies in parasitology still rely on PCR which has a number of limitations. First, PCR amplicons often range between 50-600 bp, with most over 100 bp [39,47–49]. However, most aDNA fragments are smaller than 100 bp, with fragment length distribution modes generally around 40–50 bp [50,51]. Secondly, with targeted enrichment one can include baits designed to capture large genomic regions from mitochondrial and nuclear loci in one reaction.

discussion

Moved

Libraries for shotgun sequencing and targeted enrichment were quantified using qPCR and pooled to equimolar concentrations based on the molarity of each library. Pools were size-selected using electrophoresis followed by gel excision using the QIAquick Gel Extraction Kit. Paired-end sequencing was conducted using 2x90 sequencing chemistry on an Illumina HiSeq 1500 platform at the McMaster Farncombe Metagenomics Facility.

explain procedures, kit, equipament manufacter, City, here and in all the methodology

Added, also see comment above

All ancient DNA work was done in dedicated ancient DNA facilities with a unidirectional workflow, dedicated clean rooms, and standard precautions for prevention of contamination with modern DNA. For further details on ancient DNA methods see S1 Appendix.

This should be the firts topic of the methodology, and more details are needed

Moved

Bioinformatic workflow followed previously described in-house protocols for sedaDNA analysis (see [29]). In brief, *leeHom* [52] was used for trimming and merging, reads were then mapped using *network-aware-BWA* to parasite reference sequences that had been used to design the bait set. These mapped reads were size filtered to those greater than or equal to 24 bp and string de-duplicated with *NGSeXplore*. Filtered and mapped reads were run through BLASTn to return the top 100 taxonomic hits. MEGAN was then used to assign these reads to the lowest common taxonomic rank. For further detail on bioinformatic workflow see S1 Appendix.

Results

Pre-Roman Periods

Microscopic analysis of all samples described here was previously published. *Trichuris trichiura* (whipworm) was found in the human coprolite from 6410–6150 BCE Çatalhöyük, Türkiye [53]. *Schistosoma* sp. was found in pelvic soil from a burial (5800–4000 BCE) in Tell Zeidan, Syria [54]. One coprolite from the Neolithic settlement (2500 BCE) of Durrington walls

spelling corrected

near Stonehenge, Britain was included which contained Capillarid eggs [55]. This coprolite was previously identified as human in origin using fecal lipid biomarkers. Interestingly, DNA sequenced from this coprolite contained a high proportion of reads assigned to *Canis lupus familiaris* with very few reads assigned to *Homo sapiens*, this indicates that this coprolite is likely a dog coprolite rather than a human coprolite as suggested by lipid biomarkers (see S1 Appendix). At the Bronze Age site of Must Farm in Britain (865–840 BCE), sediment samples and human and dog coprolites were included. The source of these coprolites had also been previously identified using fecal lipid biomarkers [56]. One of the coprolites previously identified as human and one of dog origin underwent shotgun sequencing and those results show that both coprolites are likely from dogs given high amounts of DNA from *Canis lupus familiaris* in those samples (see S1 Appendix). Microscopy of coprolites found eggs of *Capillaria* sp., *Echinostoma* sp., fish tapeworm (*Dibothriocephalus* sp.), giant kidney worm (*Dioctophyma renale*), and whipworm (*Trichuris* sp.). Occupation layer sediment contained all of these taxa except *Echinostoma* [56].

discussion

Amended to fit in results

wrong taxonomic writting, here, and revise all the doc

sp. added. See response to reviewers

The only Pre-Roman samples studied using ELISA were those from Must Farm as there was insufficient remaining material from the other samples. The ELISA results from these samples were indeterminate as all sample wells (8 technical replicates on two separate biological replicates for each sample) were positive for all three species (*Giardia duodenalis*, *Entamoeba histolytica*, and *Cryptosporidium*). This is an exceptionally unusual result for palaeoparasitological studies and suggests a false positive result likely from cross-reactivity with another biomolecule within the samples. Furthermore, *Cryptosporidium* has never been detected in ancient samples from Europe.

No parasite DNA was recovered from the 8 Pre-Roman samples included. Extracts were quantified using qPCR after library preparation using probes designed for the ligated sequencing adapters. The quantification of adapter-ligated molecules in these libraries (ranging from 10^6 to 10^9 copies/uL) was consistent with other samples where parasite DNA was recovered (see S1 Appendix Table D.1). Additionally, the number of filtered reads mapped to the parasite reference sequences and subsequently LCA assigned were consistent with samples from the Roman and post-Roman periods (Fig 2). Within the pre-Roman samples, 59-93% of the

Metazoan could be helminth parasite, explain

Explain

Further explanation provided

LCA-assigned reads were assigned to bacteria, while less than 10% of reads were assigned to Fungi, Metazoa, Sar, and Viridiplantae with the exception of one sample where 15% of reads were assigned to Fungi. Thus, the majority of LCA-assigned reads in the pre-Roman samples were from bacteria and we were unable to recover parasite DNA from taxa of which eggs were preserved.

Roman

In the 13 Roman period samples, the parasite taxa recovered by microscopy were less diverse than the pre-Roman sites. Roundworm (*Ascaris* sp.) was recovered from 11 samples studied, whipworm eggs were found in four, and both *Capillaria* and *Dicrocoelium* in one.

wrong taxonomic writing

species given

The only protozoa recovered from Roman period sites using ELISA was *Giardia duodenalis*. *Giardia* was detected in drain samples from Vagnari and the Villa of Vacone in Italy, and in a latrine from Sagalassos in Türkiye [31].

For aDNA analysis, reads assigned to roundworm (*Ascaris* sp.) were recovered from six samples. *Ascaris* sp. eggs were found by microscopy in all of these samples. *Dicrocoelium dendriticum* DNA was also recovered from the latrine at Arlon, Belgium; *Dicrocoelium* eggs were also found by microscopy from this site. No protozoa DNA was recovered.

Post-Roman

Similar to the Roman period, the most common helminth recovered from medieval period sites using microscopic analysis was roundworm (*Ascaris* sp.) which was found in four of five samples. Whipworm was found in two samples and fish tapeworm in one sample. The only protozoa recovered using ELISA was *Giardia duodenalis*. This was found in the latrine from Jerusalem [57].

Ancient DNA from roundworm (*Ascaris*) was found in three medieval samples studied. In the latrine sediment from Mértola, Portugal some of these reads were able to be assigned to the species-level as *Ascaris lumbricoides*, the human infecting species of *Ascaris*. In addition, reads assigned to whipworm (*Trichuris* sp.) were found in the latrine from Mértola in Portugal. Interestingly, no *Trichuris* eggs were seen on microscopic analysis of this latrine sediment.

How is it possible to define species level, since these spp. are not genetically defined using most of molecular markers ?

See response to reviewers for documentation of changes made

Species-level identification was also possible for whipworm reads from the latrine in Jerusalem. Reads were not only assigned to human whipworm (*Trichuris trichiura*), but also mouse whipworm (*Trichuris muris*). These results are comparable to results of a recent aDNA study employing shotgun sequencing to characterize microbial and eukaryotic DNA in a separate subsample from this same latrine in Jerusalem. In that study, *Ascaris lumbricoides* and *Trichuris trichiura* were detected [12].

discussion

This has been left here as it does not need a full description again in the discussion, but is treated the same as comparisons made to microscopy results throughout the results section. Having it here provides the easiest way for readers to contextualize the data.

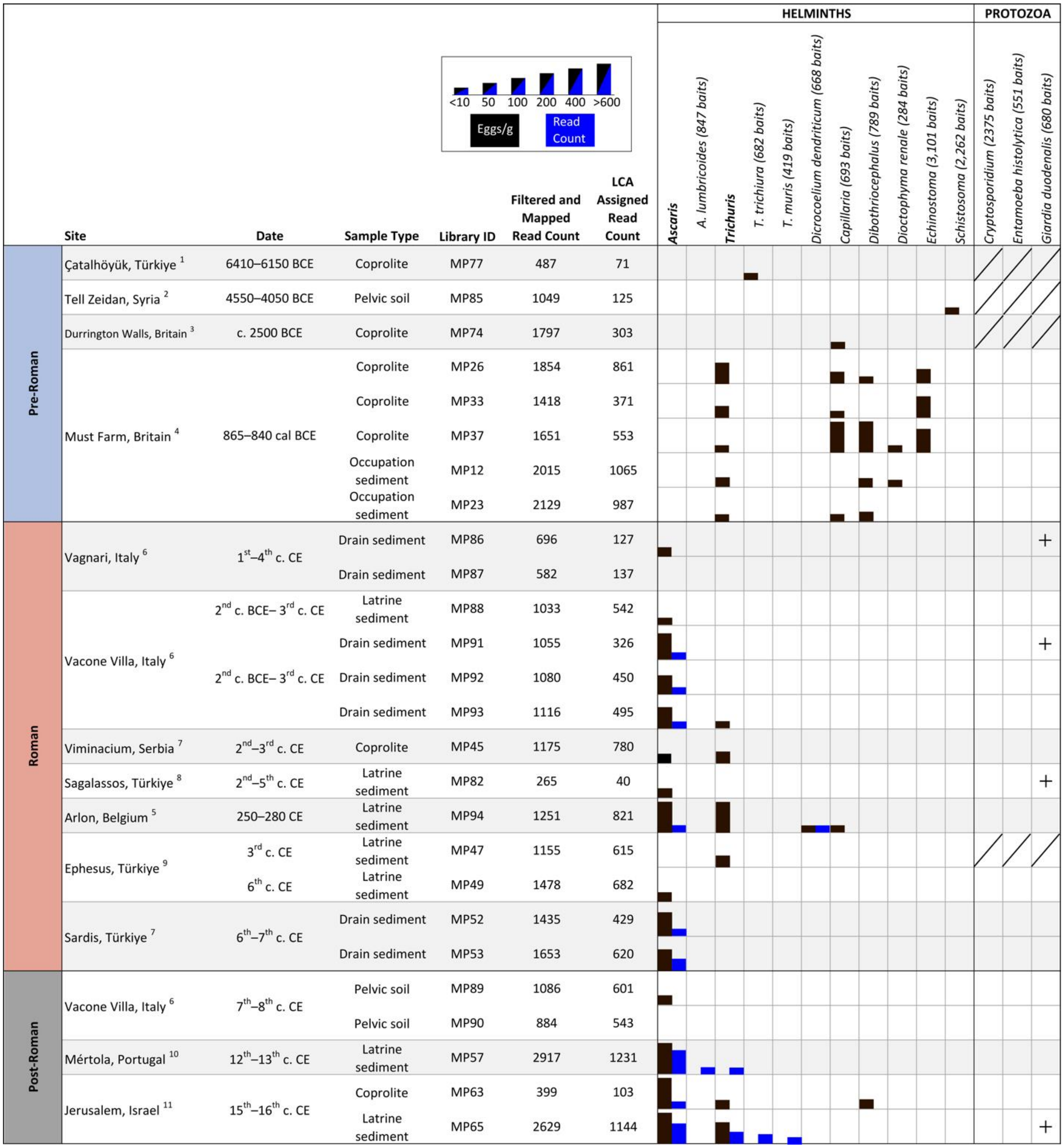

Fig 2. Summary of parasites recovered from each sample using microscopy, ELISA, and ancient DNA. Black bars represent egg concentrations identified using microscopy and blue bars represent DNA read count. Samples for which insufficient material was available for ELISA are crossed out and positive ELISA tests are marked with a +. [1] Ledger et al., 2019 [53]; [2] Anastasiou et al., 2014 [54]; [3] Mitchell et al., 2022 [55]; [4] Ledger et al., 2019 [56]; [5] Deforce et al., 2021 [58]; [6] Ledger et al., 2021 [31]; [7] Ledger et al., 2020 [59]; [8] Williams et al., 2017 [60]; [9] Ledger et al., 2018 [61]; [10] Knorr et al., 2019 [62]; [11] Yeh et al., 2015 [57].

Expansion of Results Using Ancient DNA

Shotgun sequencing on a subset of four samples was unable to recover any identifiable parasite DNA. However, using a targeted enrichment approach, parasite DNA was recovered from 9 out of 26 samples (see Fig 2 for summarized results and S3 Appendix for results by individual sample). The majority of sequenced reads assigned to human parasites were from roundworm (*Ascaris*). Additional parasite taxa from which we were able to recover DNA included the lancet liver fluke (*Dicrocoelium dendriticum*) and whipworm (*Trichuris* spp.) (Fig 2). In one of these samples (MP57 from Mértola, Portugal) whipworm eggs were not visualized by microscopy thus, aDNA analysis provides evidence for additional taxa.

The microscopic identification of *Ascaris* was replicated using aDNA analysis in six out of a total 11 Roman period samples from which *Ascaris* eggs were identified by microscopy. Three out of five medieval period samples in which *Ascaris* eggs were identified by microscopy contained *Ascaris* DNA. From both of these time periods, only three of these samples contained *Ascaris* eggs that were sufficiently preserved to still have a visible outer mamillated coat. Mamillated *Ascaris* eggs were recovered from Arlon in Belgium, Viminacium in Serbia, and the Mamluk period cesspit in Jerusalem. The mamillated coat of *Ascaris* is an important characteristic feature used for identification of the eggs, however, it can be absent in modern eggs and is often lost in archaeological contexts which makes identification challenging [63–66]. Thus, the detection of *Ascaris* DNA in samples containing decorticated and poorly preserved *Ascaris* eggs exemplifies the range of morphology that can be seen in ancient *Ascaris* eggs (Fig 5).

Figure 3 contains a comparison between the concentration of roundworm (*Ascaris*) eggs recovered by microscopy to the percent of LCA-assigned reads to Ascaridoidea or lower taxonomic rank and read count of the same. Unsurprisingly, the samples that contained the highest concentration of *Ascaris* eggs also had a higher percentage of reads assigned to *Ascaris*. For nearly all samples where *Ascaris* eggs were identified by microscopy but no DNA was recovered, the egg concentration was 30 eggs per gram or less. This suggests there may be a lower limit of detection for our sedaDNA methods which is higher than that of microscopy.

It could be interesting to discuss methodology with previous paleogenetic studies on Ascaris spp.

As we describe in the discussion we had hoped to be able to compare to other studies. However, most studies do not report egg concentrations from microscopy thus precluding us from determining if this trend is comparable to other studies

Site	Sample Type	DNA Library ID	*Ascaris* (eggs per gram)	% LCA Assigned (Ascaridoidea or Lower)	Read Count (Ascaridoidea or Lower)
Jerusalem	Cesspit sediment	MP65	1580	21.8%	249
Mértola	Latrine	MP57	1110	24.1%	297
Arlon	Latrine	MP94	990	0.5%	4
Jerusalem	Coprolite	MP63	595	5.8%	6
Vacone	Drain sediment	MP91	405	1.8%	6
Sardis	Drain sediment	MP52	305	1.2%	5
Sardis	Drain sediment	MP53	205	9.7%	60
Vacone	Drain sediment	MP93	185	1.4%	7
Vacone	Drain sediment	MP92	140	1.6%	7
Vacone	Pelvic soil	MP89	30	0.0%	0
Vagnari	Drain sediment	MP86	25	2.4%	3
Ephesus	Latrine	MP49	25	0.0%	0
Sagalassos	Latrine	MP82	20	0.0%	0
Vacone	Latrine	MP88	5	0.0%	0

Fig 3. Heatmap comparing concentration of *Ascaris* eggs recovered from each sample to percentage of LCA-assigned reads. *Ascaris* egg concentration is reported in eggs per gram based on microscopy and LCA-assigned reads include reads that were assigned to the Superfamily Ascaridoidea or lower and read count assigned to Ascaridoidea or lower. Samples are ordered in the table based on egg per gram concentration from highest to lowest concentration. colors? Description added

The sequence reads assigned to roundworm were authenticated as aDNA using *mapDamage* [67]. They showed typical deamination patterns for aDNA and fragment length distributions (Fig 4). Statistical analysis could not be done on reads from other taxa due to the low number of reads.

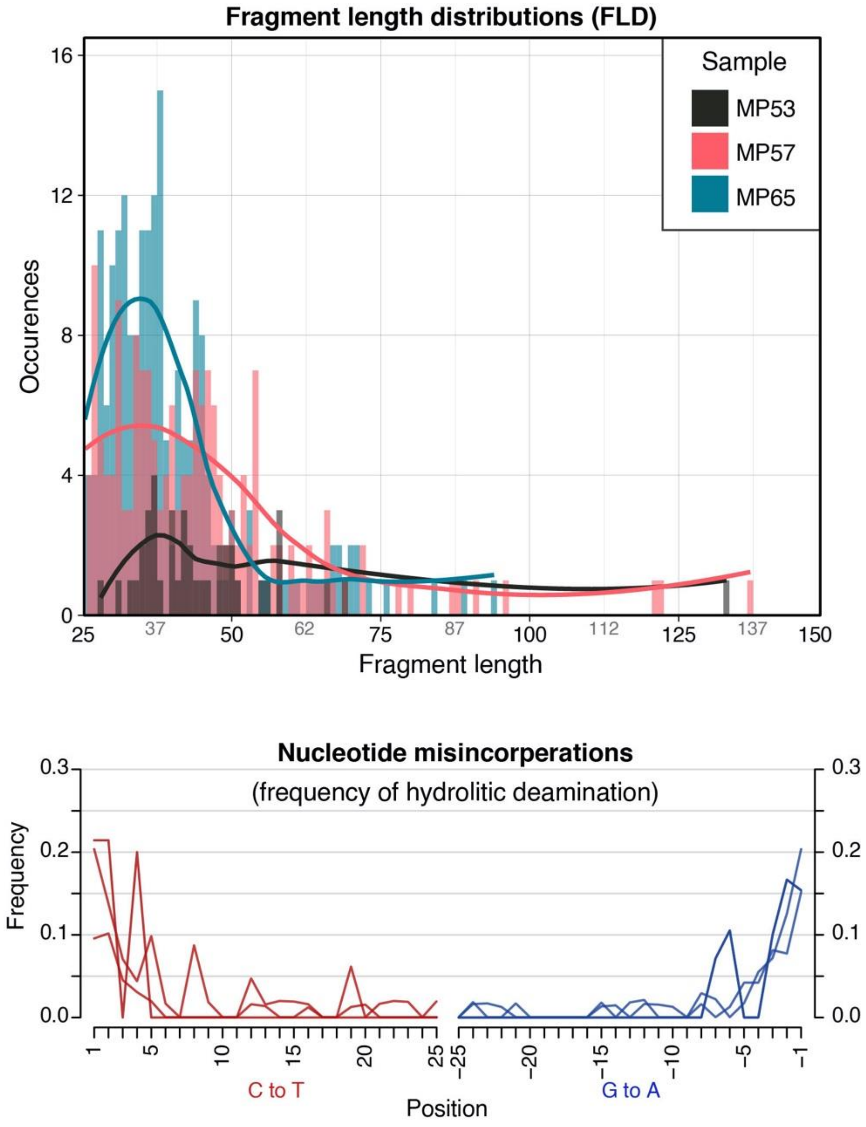

Fig 4. Deamination and fragment length distributions for *Ascaris* reads. Data generated using *mapDamage* showing C to T and G to A deamination patterns (top) and fragment length profile (bottom) for reads assigned to *Ascaris* in samples with highest read count.

Discussion

A Multimethod Approach in Paleoparasitology

Each individual method used in our study contributed complementary results to the reconstruction of parasite diversity in the past. For samples from the Neolithic and Bronze Age (where the number of surviving parasite eggs was generally low), microscopy was more

sensitive for detecting ancient parasites than was aDNA. As can be seen in Figure 2, six different taxa were recovered using microscopy and this included rare parasites in archaeological settings such as *Schistosoma, Echinostoma*, and *Dioctophyma renale*. No parasite DNA sequences were recovered in these samples using our parasite sedaDNA methods, despite all of the taxa being present in our bait set (see S2 Appendix for full list of baits); with 2,262 baits targeting *Schistosoma*, 3,101 baits for *Echinostoma*, and 284 baits for *Dioctophyma renale*. The majority of DNA recovered from these was bacterial in origin. The parasite diversity in these samples was more diverse than that seen in the Roman and medieval periods, and is further characterized by a notable absence of *Ascaris* which is the most common parasite found in later time periods. This raises the possibility that our targeted capture approach was not as efficient at detecting these parasite species as it was for *Ascaris*. Aside from parasite DNA recovery, our sedaDNA methods provided additional data for the identification of the source of coprolites studied. High read counts assigned to dog (*Canis lupus familiaris*) in the Durrington Walls and Must Farm coprolites suggests that these coprolites may in fact be from dogs not humans as previously thought. The accurate identification of coprolites is important for interpretation of parasite results.

spp. added

Roundworm (*Ascaris*) and whipworm (*Trichuris*) were the most commonly identified parasites by both microscopy and aDNA in the Roman and medieval period samples. In all Roman period samples in which parasite DNA was recovered, eggs from the assigned taxa were identified by microscopy. No additional parasites were detected using ancient DNA analysis for our Roman period sites, and aside from reads assigned to the lancet liver fluke (*Dicrocoelium dendriticum*) in the latrine from Arlon, Belgium, all other reads were from *Ascaris* and could not be assigned to a lower rank than the genus-level. This was not the case for the medieval period samples. In medieval period samples, *Ascaris* reads could be assigned to the species-level in one case, and additional taxa were recovered that were not identified using microscopy. The only time period where whipworm (*Trichuris*) DNA was recovered was from our medieval period samples, despite *Trichuris* being identified by microscopy in Roman period samples.

Combining aDNA with microscopy in paleoparasitology provides the potential to identify ancient parasites to the species-level [15,20,22]. For many parasite taxa, egg morphology

cannot be used to accurately identify eggs to the species-level [20,68,69]. We were able to do this in few cases using our targeted enrichment approach. In latrine sediment from the medieval site of Mértola, *Ascaris* reads were assigned to the species-level as *Ascaris lumbricoides*. *Ascaris lumbricoides* is classically the human infecting species of *Ascaris* which fits with identification of this species in a latrine that would contain primarily human fecal waste. However, it is not surprising that for all other samples, reads could only be assigned to the genus-level as there is minimal genetic variation between *Ascaris lumbricoides* and *Ascaris suum*, which is the species that classically is considered to infect pigs [70,71]. Additionally, two species of *Trichuris* were identified in the latrine sediment from 15th–16th c. CE Jerusalem. Both the human-infecting species, *Trichuris trichiura*, and the mouse-infecting species, *Trichuris muris*, were identified. The presence of *T. muris* in the latrine could be a result of mice having access to the latrine or indicate that material contaminated with feces from rodents was deposited in the latrine.

Explain. How does target enrichment make possible the definition at sp. level?

Done

? Asked above. Problem. Specially working with latrine samples

See response to reviewers

what variation? which genetic region?

We have expanded this disucssion to discuss genetic similarity between the two species and the current debates on species distinction

One important contribution of our study is that aDNA analysis was able to replicate identification of *Ascaris* eggs that had atypical morphology as a result of degradation and environmental conditions after egg deposition. The outer mamillated coat of *Ascaris* eggs is a key morphological feature used in identification of the eggs. The *Ascaris* eggs identified by microscopy in samples from this study were all decorticated except for those from Arlon, Belgium (Fig 5). Thus, if one is only identifying eggs with preserved mamillated coats, they will grossly underestimate the prevalence of *Ascaris* in the past. Even in modern studies, decorticated roundworm eggs can be easily misidentified [66]. Thus, the addition of aDNA analysis can confirm identification in cases of uncertainty due to taphonomic changes and poor egg preservation and allows us to more confidently explore the appearance of ancient eggs.

This statment is controversial, since the Ascaris sp. results are not from aDNA extracted directly from these isolated eggs with atypical morphology.

See response to reviewers for documentation of how this has been changed to address the reviewers concerns.

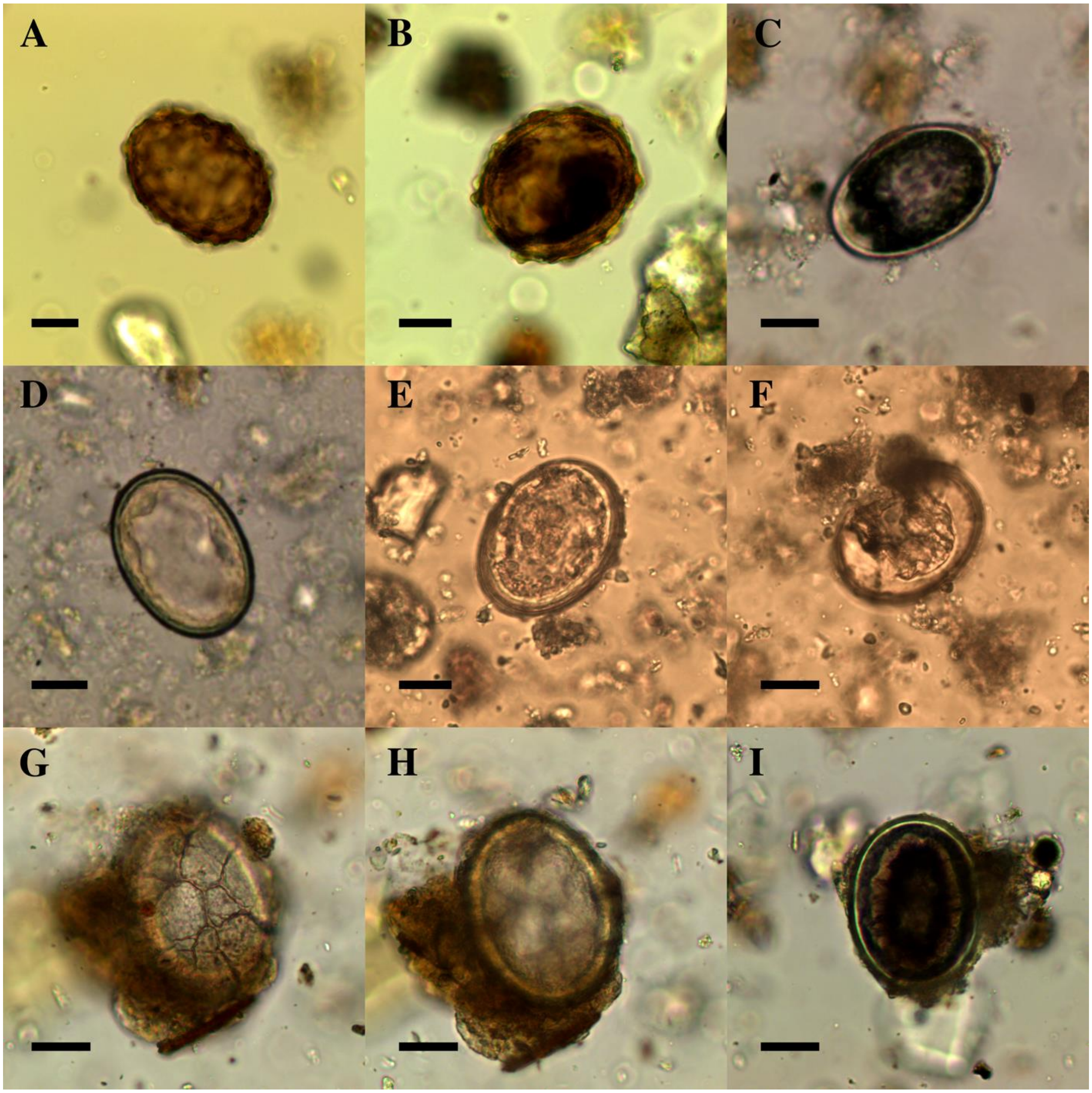

Fig 5. Variation in morphological appearance of *Ascaris* eggs identified in samples with confirmed presence of *Ascaris* using aDNA methods. (A) fertilized egg from Arlon with mamillated coat; (B) fertilized egg from Arlon with mamillated coat; (C) fertilized decorticated egg from Mértola (image credit: Delaney Knorr); (D) fertilized decorticated egg from Mértola (image credit: Delaney Knorr); (E) fertilized decorticated egg from Vacone; (F) fertilized decorticated egg from Vacone; (G and H) fertilized decorticated egg from Sardis with surface cracking; (I) fertilized decorticated egg from Sardis. Bar=? Added

The bait set designed for targeted enrichment in our study covered 121 taxa. Most studies in paleoparasitology relying on PCR methods targeted a maximum of 5 taxa or even a single taxon previously identified by microscopy [13,24,27,28,36,49]. Targeted enrichment has only previously been used in parasite aDNA research for detecting malaria (*Plasmodium* sp.) in skeletal remains [19]. On the other hand, while we are limited by coverage of the bait set and references sequences available for robust bait design, targeted enrichment allowed for recovery of parasite DNA when shotgun sequencing to an average depth of 3.3 million reads (total read counts from shotgun sequencing ranged from 2.2–4.0 million reads) was unsuccessful in recovering any parasite DNA. In published palaeoparasitological studies relying on shotgun sequencing, most samples were sequenced to a depth of at least 5 million reads with some samples being sequenced to a depth of 60 million reads [12,15,22]. The cost of sequencing to this level is not financially feasible for many studies and most aDNA and paleoparasitology labs. Additionally, in most cases less than 5% of these sequenced reads are assigned to target organisms [72]. In our samples even with targeted enrichment, <0.001% of reads were assigned to parasites. Due to the very small proportion of parasite DNA in our sediment samples (which is otherwise dominated by bacteria and currently unidentifiable DNA, along with plants and fungi), very deep shotgun sequencing would be needed to find parasite DNA in environmental samples. Paleogenetic studies in parasitology are likely to continue to lag behind that seen for other pathogens without changes that make the use of aDNA more cost effective. Microscopy is a relatively cheap and reliable method for egg identification from archaeological sediments making it a preferred method for many researchers.

While ancient DNA analysis is a promising addition to the methodological armamentarium in paleoparasitology, our results suggest that currently microscopy remains the most cost-effective and reliable screening tool in paleoparasitology. When resources allow, ELISA and aDNA can be utilized alongside microscopy to analyze a sample more comprehensively. In palaeoecological studies as well, sedaDNA has been found to function best as a complementary proxy, as other methods such as palynology and macrofossil identification tend to find similar, but non-overlapping taxa [73–75]. Microscopy continues to successfully recover parasites eggs in samples with low egg concentration. Based on our results comparing

eggs per gram concentration generated from microscopic analysis to percent of LCA-assigned reads, there appears to be a minimum concentration of eggs needed for increased chance of DNA recovery using the currently described methods. Samples that contained 30 eggs per gram or lower of *Ascaris* eggs did not contain any detectable *Ascaris* DNA. It is difficult to directly compare this to other studies as many do not report eggs per gram or had higher egg concentrations in all samples studied [13]. However, both in our study and other palaeoparasitological studies, there are cases where parasite DNA is detected in the absence of any preserved helminth eggs [15,24,36]. In these cases it is possible that taphonomic changes to the eggs have resulted in inability to identify them by microscopy but that their DNA can still be captured and sequenced. Alternatively, that DNA from adult worms themselves rather than eggs is being detected. It is also likely that eggs are not evenly distributed within a sample and studying different subsamples with microscopy and aDNA will provide different findings.

The complexities of parasite egg preservation and in turn parasite DNA preservation is poorly understood. This diverse group of organisms has variable eggshell structures, rates of embryological development, environmental niches, and are recovered from different contexts including in the gastrointestinal space as in the case of pelvic soil as well as cesspit, latrine, and drain contexts. We were able to detect parasite DNA in various sample types including pelvic soil, coprolites, and latrine or cesspit samples. We had the most success with identifying *Ascaris* using our sedaDNA methods and targeted enrichment. The ability to more easily recover *Ascaris* DNA from all samples may be related to both methodological factors as well as parasite biology. As part of its life cycle, *Ascaris* develops in soil, thus by necessity it is resistant to harsh environmental conditions. In addition, extensive reference sequences for *Ascaris* allowed us to create a robust set of baits for *Ascaris*. A total of 1,769 of 30,240 baits with average tiling density of 7x were for either *Ascaris lumbricoides* or *Ascaris suum* (see S2 Appendix for list of baits). In comparison, our bait set had very good coverage of taxa identified by microscopy for which we were not able to recover DNA. *Echinostoma* had a total of 3,213 baits (average tiling density ranged for each species from 8.4–21.8x), *Schistosoma* spp. had 2,262 baits (average tiling density ranged for each species from 8.1–21.8x), and *Dibothriocephalus* (syn.

Trichuris spp. as well

This is true, though in combination with further points made in the discussion this is our reasoning for improved detection of Ascaris, we agree that this cannot be the sole reason we would detect Ascaris rather than Trichuris

Diphyllobothrium) had 1,980 baits (average tiling density ranged for each species from 8.8–23.4x).

Parasite biology will also influence DNA recovery [24]. The size of adult worms from different helminths is variable. For example, *Ascaris lumbricoides* can grow up to 30 cm long whereas *Trichuris trichiura* is typically only 5 cm long and *Dibothriocephalus latus* can grow to 1000 cm [68]. Thus, the biomass of the adult worms would be expected to vary by taxa. This would impact the amount of DNA expected from each species in pelvic soil samples, as pelvic soil samples would contain decomposed intestinal contents including helminths causing infection at the time of death. The number of eggs excreted per day is also quite variable, with *Ascaris lumbricoides* producing around 200,000 eggs per day [76] and *Trichuris trichiura* only 18,000 eggs per day [77]. These factors may be compounded by sedaDNA preservation characteristics, which are thought to necessitate binding to sedimentary minerals [78–81]. If parasite DNA does not come in contact with sedimentary minerals before being broken down by microbes in the local microbiome, those parasite DNA fragments are unlikely to preserve and be recoverable.

But only should be considered when working with abdominal region samples

We agree and have specified in the following sentences we are discussing this concept using pelvic soil (abdominal samples).

While sedaDNA with targeted enrichment has the potential to significantly aid in palaeoparasitological research, further methodological improvements are needed to reach the taxonomic characterization already viable with the gold standard method within the field—microscopy. Furthermore, *Giardia duodenalis* antigen was detected using ELISA from four sites, and although our bait set did include protozoa, we were not able to recover any DNA sequences from *Giardia*. Thus, ELISA continues to have a unique value in that it is a useful additional method for maximizing recovery of common intestinal protozoa, which are rarely preserved well enough to be visualized using microscopy and have been detected in very few aDNA studies thus far [41]. The indeterminate results from Must Farm highlight an important limitation of ELISA which is the potential for cross-reactivity with unknown antigens that may be found in soil. Further, optimization of aDNA methods for detection of protozoa would be valuable to confirm results with a second method.

Temporal Changes in Parasites in the Roman Period

Using a multimethod approach, we aimed to produce a more accurate reconstruction of parasite infection in the Roman period. Inclusion of pre-Roman and post-Roman samples from areas that were at one time part of the Roman Empire provides important comparative data to understand how parasite diversity may have changed during the Roman period. The samples included in our study contribute a small subset of results for parasite presence in areas that were once part of the Roman Empire from the Neolithic through the medieval period. The parasite taxa recovered from the pre-Roman sites were much more diverse than any other time period. At these sites we recovered various zoonotic parasites including *Echinostoma* and fish tapeworm (*Dibothriocephalus* sp.) which are spread by ingestion of raw or undercooked fish, frogs, or mollusks, as well as *Capillaria* from two sites. This is in conjunction with one common soil-transmitted helminth, whipworm (*Trichuris trichiura*), which is spread by the fecal-oral route.

By comparison in the Roman period, we observe almost exclusively soil-transmitted helminths, roundworm (*Ascaris* sp.) and whipworm (*Trichuris trichiura*). One exception is the site of Arlon, Belgium where *Capillaria* and the lancet liver fluke (*Dicrocoelium dendriticum*) were also recovered. Similarly, in medieval period sites, soil-transmitted helminths are almost exclusively present, aside from a coprolite from Jerusalem where fish tapeworm (*Dibothriocephalus* sp.) was also found. idem

type of transmission of these parasites?

Specified

When these results are included within the published literature on parasite presence in these time periods, our results are comparable to patterns seen using a much larger dataset (see Tables F.1–F.3 in S1 Appendix for a review of published studies). The proportion of sites with whipworm is consistently high in all time periods, while roundworm appears to increase in frequency in the Roman period and remains stable at that level in the medieval period (Fig 6). Roundworm was recovered from 35% of pre-Roman sites, which increased to 67% of Roman and 79% of medieval period sites. The increase in proportion of sites where roundworm was found in the Roman and medieval periods may reflect decreased sanitation and hygiene. Numerous other factors such as increased urbanization, cultural preferences for the use of chamber pots, and agricultural practices involving the use of human feces as fertilizer may have all contributed to this increase in fecal-oral parasite presence [31,82].

Only roundworms?

Yes, as can be seen in Fig 6, there is a clear increase in Ascaris (roundworm), while there is an increase in whipworm in medieval period there is not a clear increase from the pre-roman to roman period. We have added this nuance

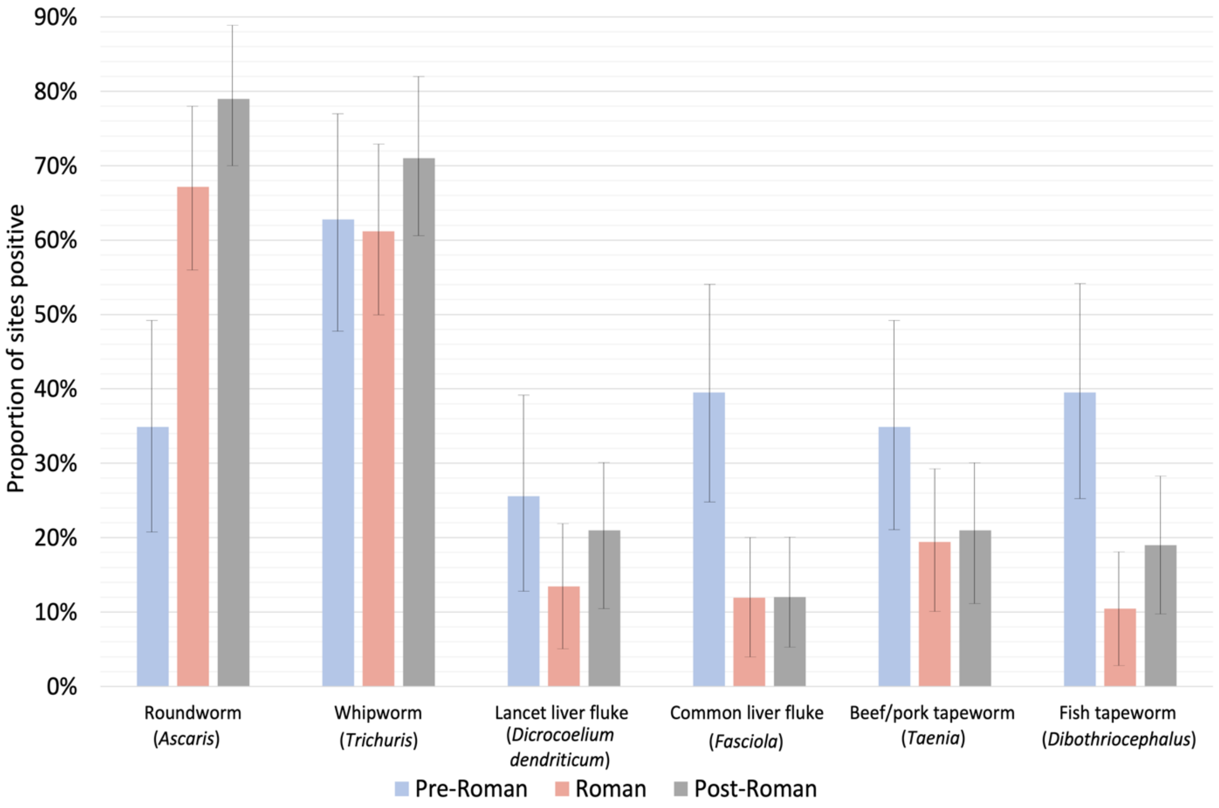

Fig 6. Temporal trends in parasite diversity before, during, and after the Roman period for the most common helminths recovered from archaeological samples. For each parasite, bars indicate the proportion of studied sites where each parasite was recovered for pre-Roman, Roman, and post-Roman sites with 95% confidence intervals. See S1 Appendix for individual studies used to generate temporal trends.

Zoonotic and foodborne parasites are different concepts. Foodborne parasites are not only related to consuming meat

Amended as suggested

Zoonotic and foodborne parasites (parasites spread from animals to humans directly or through ingestion of meat) [83], including fish tapeworm (*Dibothriocephalus* sp.), liver flukes (*Dicrocoelium dendriticum* and *Fasciola*) and beef/pork tapeworm (*Taenia* sp.) are generally more common in pre-Roman time periods compared to the later Roman and medieval periods (Fig 6). This may reflect changes to human-animal interactions and dietary preferences. It is possible that the decline in lancet liver fluke during the Roman period is an anomaly in our data as the proportion of sites where it was found in the medieval period is quite consistent with the pre-Roman period. However, it does appear that *Fasciola* liver fluke was more common in the

pre-Roman period compared to later time periods. It was found at 40% of pre-Roman sites compared to 12% of Roman and 12% of medieval sites. Both types of liver flukes in fecal samples can reflect true infection or false parasitism, usually from consumption of animal liver containing eggs [84]. In all time periods, nearly all sites where *Fasciola* was found are North of the Alps.

A similar pattern as seen for liver flukes is seen for fish tapeworm (*Dibothriocephalus* sp.). Fish tapeworm was most common in pre-Roman sites, being found at 40% of sites and then decreases to 10% in the Roman period and 19% in the medieval period. Fish tapeworm is acquired from ingestion of raw or undercooked freshwater fish in Europe. The decline in the Roman period may represent a preference for cooked fish or, alternatively, a decreased reliance on freshwater fish but rather marine fish.

There are many intricacies of transmission and determinants of infection that can be explored for each individual parasite. However, what is most striking overall is the high proportion of sites in all time periods in Europe where we find evidence for enteric parasites. Roundworm is present at 67% of Roman sites studied and whipworm is found at 61% of sites. Of course, a major limitation of this type of analysis is publication bias. However, there is no doubt that these two parasites contributed to the burden of disease in most Roman communities. Many infections with these two species can be asymptomatic; however, they can also cause gastrointestinal upset and for children result in nutrient deficiencies, stunting of growth, and intellectual disability [85].

Conclusions

The vast majority of work done in paleoparasitology or archeoparasitology has relied on microscopic analysis of sediments and palaeofaeces to identify parasites. ELISA has also been used by some groups to detect ancient protozoa. We have shown that sedaDNA methods combined with targeted enrichment can be used to detect parasite DNA in as little as 0.25 g of paleofecal sediment. A multimethod approach in paleoparasitology allows for the most complete reconstruction of past parasite diversity currently available to us. However, microscopy continues to be the most sensitive method for detecting helminths in paleofecal

samples and appears to detect helminth eggs present in lower concentrations than our sedaDNA methods are able to detect. ELISA is uniquely able to detect protozoa such as *Giardia* in our samples. In our set of samples, aDNA was able to confirm identification of *Ascaris* eggs with atypical morphology, provide species-level identification for some parasites, and detect additional parasites not seen by microscopy and ELISA. As these methods are refined and applied to additional samples, they may help facilitate a more holistic analysis of parasite phylogeography and parasite biology through time.

The two statements are not conclusive. One is just a suggestion. The sp. level identification needs explanation.

We have amended the wording to more accurately state that we have replicated identification of Ascaris in these samples using another method but removed the wording 'confirmed' as previously used.

Applying this multimethod approach to 26 coprolite, latrine, and sewer samples from the Neolithic through medieval period allowed us to characterize temporal trends in parasite infection around the rise, expansion and eventual decline of the Roman Empire. We found a trend of decreased zoonotic and foodborne parasites from the pre-Roman to Roman and medieval periods and concurrent increase in parasites spread by the fecal-oral route, especially roundworm.

Acknowledgments

We would like to thank local archaeological institutions, and excavation teams from the sites included in this study, and the official parties granting the required permissions. We thank Christa Clamer for invaluable work providing material and contextual interpretation of our results. We thank Techlab©, Blacksburg, USA for their kind donation of ELISA kits used in this research. Thanks to Brian Golding for providing access to his computational resources, and to Ana Duggan for assistance and guidance on bioinformatic workflow. Thanks to all members and affiliates of the McMaster Ancient DNA Centre for their ongoing support of this work. Numerous individuals have contributed to various stages of this work; from the site of Sagalassos we would like to thank the Research Foundation Flanders and the Research Fund of KU Leuven for supporting the research for this paper. From the Ephesus site we would like to thank the late excavation director Sabine Ladstätter, who made it possible for us to collect samples for analysis. From Vacone we would like to thank Devin Ward, Gary Farney, Dylan Bloy, and the Soprintendenza Archeologia, Belle arti e Paesaggio per l'area metropolitana di Roma e la provincia di Rieti.

Funding Sources

This work was supported by The Social Sciences and Humanities Research Council of Canada [752-2016-2085], a Tidmarsh Cambridge Scholarship from the Cambridge Commonwealth, European and International Trust and Trinity Hall College, and the Society for the Promotion of Roman Studies to MLL.

TJM and HNP wish to thank the CANA Foundation for their financial support alongside the Garfield Weston Foundation, the Social Sciences and Humanities Research Council of Canada, CIFAR, and Natural Sciences and Engineering Research Council of Canada for funding this research.

References

1. Bos KI, Kühnert D, Herbig A, Esquivel-Gomez LR, Valtueña AA, Barquera R, et al. Paleomicrobiology: Diagnosis and evolution of ancient pathogens. Annu Rev Microbiol. 2019. doi:10.1146/annurev-micro-090817-062436

2. Arriola LA, Cooper A, Weyrich LS. Palaeomicrobiology: Application of ancient DNA sequencing to better understand bacterial genome evolution and adaptation. Frontiers in Ecology and Evolution. 2020;8. doi:10.3389/fevo.2020.00040

3. Duchêne S, Ho SYW, Carmichael AG, Holmes EC, Poinar H. The recovery, interpretation and use of ancient pathogen genomes. Curr Biol. 2020;30: R1215–R1231. doi:10.1016/j.cub.2020.08.081

4. Lewis CM Jr, Akinyi MY, DeWitte SN, Stone AC. Ancient pathogens provide a window into health and well-being. Proc Natl Acad Sci U S A. 2023;120: e2209476119. doi:10.1073/pnas.2209476119

5. Wagner DM, Klunk J, Harbeck M, Devault A, Waglechner N, Sahl JW, et al. *Yersinia pestis* and the Plague of Justinian 541–543 AD: a genomic analysis. Lancet Infect Dis. 2014;14: 319–326. doi:10.1016/S1473-3099(13)70323-2

6. Key FM, Posth C, Esquivel-Gomez LR, Hübler R, Spyrou MA, Neumann GU, et al. Emergence of human-adapted *Salmonella enterica* is linked to the Neolithization process. Nat Ecol Evol. 2020;4: 324–333. doi:10.1038/s41559-020-1106-9

7. Mühlemann B, Jones TC, Damgaard P de B, Allentoft ME, Shevnina I, Logvin A, et al. Ancient hepatitis B viruses from the Bronze Age to the Medieval period. Nature. 2018;557: 418–423. doi:10.1038/s41586-018-0097-z

8. Rascovan N, Sjögren K-G, Kristiansen K, Nielsen R, Willerslev E, Desnues C, et al. Emergence and spread of basal lineages of *Yersinia pestis* during the Neolithic decline. Cell. 2019;176: 295-305.e10. doi:10.1016/j.cell.2018.11.005

9. Spyrou MA, Bos KI, Herbig A, Krause J. Ancient pathogen genomics as an emerging tool for infectious disease research. Nat Rev Genet. 2019;20: 323–340. doi:10.1038/s41576-019-0119-1

10. Eaton K, Featherstone L, Duchene S, Carmichael AG, Varlık N, Golding GB, et al. Plagued by a cryptic clock: insight and issues from the global phylogeny of *Yersinia pestis*. Commun Biol. 2023;6: 23. doi:10.1038/s42003-022-04394-6

11. Hagan RW, Hofman CA, Hübner A, Reinhard K, Schnorr S, Lewis CM Jr, et al. Comparison of extraction methods for recovering ancient microbial DNA from paleofeces. Am J Phys Anthropol. 2020;171: 275–284. doi:10.1002/ajpa.23978

12. Sabin S, Yeh H-Y, Pluskowski A, Clamer C, Mitchell PD, Bos KI. Estimating molecular preservation of the intestinal microbiome via metagenomic analyses of latrine sediments from two medieval cities. Philos Trans R Soc Lond B Biol Sci. 2020;375: 20190576. doi:10.1098/rstb.2019.0576

13. Flammer PG, Dellicour S, Preston SG, Rieger D, Warren S, Tan CKW, et al. Molecular archaeoparasitology identifies cultural changes in the Medieval Hanseatic trading centre of Lübeck. Proc R Soc B. 2018;285: 20180991. doi:10.1098/rspb.2018.0991

14. Flammer PG, Ryan H, Preston SG, Warren S, Přichystalová R, Weiss R, et al. Epidemiological insights from a large-scale investigation of intestinal helminths in Medieval Europe. PLoS Negl Trop Dis. 2020;14: e0008600. doi:10.1371/journal.pntd.0008600

15. Søe MJ, Nejsum P, Seersholm FV, Fredensborg BL, Habraken R, Haase K, et al. Ancient DNA from latrines in Northern Europe and the Middle East (500 BC-1700 AD) reveals past parasites and diet. PLOS One. 2018;13: e0195481. doi:10.1371/journal.pone.01954

16. Appelt S, Fancello L, Le Bailly M, Raoult D, Drancourt M, Desnues C. Viruses in a 14th-century coprolite. Appl Environ Microbiol. 2014;80: 2648–2655. doi:10.1128/AEM.03242-13

17. Ng TFF, Chen L-F, Zhou Y, Shapiro B, Stiller M, Heintzman PD, et al. Preservation of viral genomes in 700-y-old caribou feces from a subarctic ice patch. Proc Natl Acad Sci U S A. 2014;111: 16842–16847. doi:10.1073/pnas.1410429111

18. Ferreira LF. An introduction to paleoparasitology. In: Reinhard KJ, Araújo A, editors. Foundations of Paleoparasitology. Rio de Janeiro: Editoria Fiocruz; 2014. pp. 27–42.

19. Marciniak S, Prowse TL, Herring DA, Klunk J, Kuch M, Duggan AT, et al. *Plasmodium falciparum* malaria in 1st–2nd century CE southern Italy. Curr Biol. 2016;26: R1220–R1222. doi:10.1016/j.cub.2016.10.016

20. Côté NM-L, Le Bailly M. Palaeoparasitology and palaeogenetics: Review and perspectives for the study of ancient human parasites. Parasitology. 2018;145: 656–664. doi:10.1017/S003118201700141X

21. Selbach C, Jorge F, Dowle E, Bennett J, Chai X, Doherty J-F, et al. Parasitological research in the molecular age. Parasitology. 2019;146: 1361–1370. doi:10.1017/S0031182019000726

22. Tams KW, Jensen Søe M, Merkyte I, Valeur Seersholm F, Henriksen PS, Klingenberg S, et al. Parasitic infections and resource economy of Danish Iron Age settlement through ancient DNA sequencing. PLoS One. 2018;13: e0197399. doi:10.1371/journal.pone.0197399

23. Chessa D, Murgia M, Sias E, Deligios M, Mazzarello V, Fiamma M, et al. Metagenomics and microscope revealed *T. trichiura* and other intestinal parasites in a cesspit of an Italian nineteenth century aristocratic palace. Sci Rep. 2020;10: 12656. doi:10.1038/s41598-020-69497-8

24. Roche K, Capelli N, Pacciani E, Lelli P, Pallecchi P, Bianucci R, et al. Gastrointestinal parasite burden in 4th-5th c. CE Florence highlighted by microscopy and paleogenetics. Infect Genet Evol. 2021;90: 104713. doi:10.1016/j.meegid.2021.104713

25. Askari Z, Ruehli F, Bouwman A, Shariati V, Naddaf SR, Otranto D, et al. Genomic palaeoparasitology traced the occurrence of *Taenia asiatica* in ancient Iran (Sassanid Empire, 2th cent. CE-6th cent. CE). Sci Rep. 2022;12: 12045. doi:10.1038/s41598-022-10690-2

26. Doyle SR, Søe MJ, Nejsum P, Betson M, Cooper PJ, Peng L, et al. Population genomics of ancient and modern *Trichuris trichiura*. Nature Communications. 2022;13: 3888. Available: https://www.nature.com/articles/s41467-022-31487-x

27. Iñiguez AM, Brito L, Guedes L, Chaves SA de M. Helminth infection and human mobility in sambaquis: Paleoparasitological, paleogenetic, and microremains investigations in Jabuticabeira II, Brazil (2890 ± 55 to 1805 ± 65 BP). Holocene. 2022;32: 200–207. doi:10.1177/09596836211060490

28. Oh CS, Seo M, Lee HJ, Kim MJ, Lim D-S, Shin DH. Genetic analysis of ancient *Clonorchis sinensis* eggs attained from Goryeong Mummy of Joseon Dynasty Period. J Parasitol. 2022;108: 70–78. doi:10.1645/21-49

29. Murchie TJ, Kuch M, Duggan AT, Ledger ML, Roche K, Klunk J, et al. Optimizing extraction and targeted capture of ancient environmental DNA for reconstructing past environments

using the PalaeoChip Arctic-1.0 bait-set. Quat Res. 2021;99: 305–328. doi:10.1017/qua.2020.59

30. Anastasiou E, Mitchell PD. Simplifying the process of extracting intestinal parasite eggs from archaeological sediment samples: A comparative study of the efficacy of widely-used disaggregation techniques. International Journal of Paleopathology. 2013;3: 204–207. doi:10.1016/j.ijpp.2013.04.004

31. Ledger ML, Micarelli I, Ward D, Prowse TL, Carroll M, Killgrove K, et al. Gastrointestinal infection in Italy during the Roman Imperial and Longobard periods: A paleoparasitological analysis of sediment from skeletal remains and sewer drains. International Journal of Paleopathology. 2021;33: 61–71. doi:10.1016/j.ijpp.2021.03.001

32. Gonçalves MLC, da Silva VL, de Andrade CM, Reinhard K, da Rocha GC, Le Bailly M, et al. Amoebiasis distribution in the past: first steps using an immunoassay technique. Trans R Soc Trop Med Hyg. 2004;98: 88–91. doi:10.1016/S0035-9203(03)00011-7

33. Le Bailly M, Bouchet F. Paléoparasitologie et immunologie. L'exemple d'*Entamoeba histolytica*. ArcheoSciences. 2006;30: 129–135. doi:10.4000/archeosciences.281

34. Le Bailly M, Bouchet F. A first attempt to retrace the history of dysentery caused by *Entamoeba histolytica*. In: Mitchell PD, editor. Sanitation, latrines and intestinal parasites in past populations. Farnham: Ashgate; 2015. pp. 219–228.

35. Mitchell PD, Stern E, Tepper Y. Dysentery in the crusader kingdom of Jerusalem: an ELISA analysis of two medieval latrines in the City of Acre (Israel). J Archaeol Sci. 2008;35: 1849–1853. doi:10.1016/j.jas.2007.11.017

36. Søe MJ, Nejsum P, Fredensborg BL, Kapel CMO. DNA typing of ancient parasite eggs from environmental samples identifies human and animal worm infections in Viking-age settlement. J Parasitol. 2015;101: 57–63. doi:10.1645/14-650.1

37. Kaisar MMM, Brienen EAT, Djuardi Y, Sartono E, Yazdanbakhsh M, Verweij JJ, et al. Improved diagnosis of *Trichuris trichiura* by using a bead-beating procedure on ethanol preserved stool samples prior to DNA isolation and the performance of multiplex real-time PCR for intestinal parasites. Parasitology. 2017;144: 965–974. doi:10.1017/S0031182017000129

38. Dabney J, Knapp M, Glocke I, Gansauge M-T, Weihmann A, Nickel B, et al. Complete mitochondrial genome sequence of a Middle Pleistocene cave bear reconstructed from ultrashort DNA fragments. Proc Natl Acad Sci U S A. 2013;110: 15758–15763. doi:10.1073/pnas.1314445110

39. Côté NML, Daligault J, Pruvost M, Bennett EA, Gorgé O, Guimaraes S, et al. A new high-throughput approach to genotype ancient human gastrointestinal parasites. PLoS One. 2016;11: e0146230. doi:10.1371/journal.pone.0146230

40. Wood JR, Díaz FP, Latorre C, Wilmshurst JM, Burge OR, González F, et al. Ancient parasite DNA from late Quaternary Atacama Desert rodent middens. Quat Sci Rev. 2019;226: 106031. doi:10.1016/j.quascirev.2019.106031

41. Wiscovitch-Russo R, Rivera-Perez J, Narganes-Storde YM, García-Roldán E, Bunkley-Williams L, Cano R, et al. Pre-Columbian zoonotic enteric parasites: An insight into Puerto Rican indigenous culture diets and life styles. PLoS One. 2020;15: e0227810. doi:10.1371/journal.pone.0227810

42. Leles D, Araújo A, Ferreira LF, Vicente ACP, Iñiguez AM. Molecular paleoparasitological diagnosis of *Ascaris* sp. from coprolites: new scenery of ascariasis in pre-Columbian South America times. Mem Inst Oswaldo Cruz, Rio de Janeiro, Vol. 2008;103: 106–108.

43. Meyer M, Kircher M. Illumina sequencing library preparation for highly multiplexed target capture and sequencing. Cold Spring Harb Protoc. 2010;2010: db.prot5448. doi:10.1101/pdb.prot5448

44. Kircher M, Sawyer S, Meyer M. Double indexing overcomes inaccuracies in multiplex sequencing on the Illumina platform. Nucleic Acids Res. 2012;40: e3. doi:10.1093/nar/gkr771

45. Benson DA, Cavanaugh M, Clark K, Karsch-Mizrachi I, Lipman DJ, Ostell J, et al. GenBank. Nucleic Acids Res. 2013;41: D36-42. doi:10.1093/nar/gks1195

46. NCBI Resource Coordinators. Database resources of the National Center for Biotechnology Information. Nucleic Acids Res. 2016;44: D7-19. doi:10.1093/nar/gkv1290

47. Oh CS, Seo M, Chai JY, Lee SJ, Kim MJ, Park JB, et al. Amplification and sequencing of *Trichuris trichiura* ancient DNA extracted from archaeological sediments. J Archaeol Sci. {2010;37: 1269–1273. doi:10.1016/j.jas.2009.12.029

48. Jaeger LH, Iñiguez AM. Molecular paleoparasitological hybridization approach as effective tool for diagnosing human intestinal parasites from scarce archaeological remains. PLoS One. 2014;9: e105910. doi:10.1371/journal.pone.0105910

49. Hong JH, Oh CS, Seo M, Chai J-Y, Shin DH. Ancient *Ascaris* DNA Sequences of Cytochrome B, Cytochrome C Oxidase Subunit 1, NADH Dehydrogenase Subunit 1, and Internal Transcribed Spacer 1 Genes from Korean Joseon Mummy Feces. J Parasitol. 2017;103: 795–800. doi:10.1645/16-102

50. Sawyer S, Krause J, Guschanski K, Savolainen V, Pääbo S. Temporal patterns of nucleotide misincorporations and DNA fragmentation in ancient DNA. PLoS One. 2012;7: e34131. doi:10.1371/journal.pone.0034131

51. Kistler L, Ware R, Smith O, Collins M, Allaby RG. A new model for ancient DNA decay based on paleogenomic meta-analysis. Nucleic Acids Res. 2017;45: 6310–6320. doi:10.1093/nar/gkx361

52. Renaud G, Stenzel U, Kelso J. leeHom: adaptor trimming and merging for Illumina sequencing reads. Nucleic Acids Res. 2014;42: e141. doi:10.1093/nar/gku699

53. Ledger ML, Anastasiou E, Shillito L-M, Mackay H, Bull ID, Haddow SD, et al. Parasite infection at the early farming community of Çatalhöyük. Antiquity. 2019;93: 573–587. doi:10.15184/aqy.2019.61

54. Anastasiou E, Lorentz KO, Stein GJ, Mitchell PD. Prehistoric schistosomiasis parasite found in the Middle East. Lancet Infectious Diseases. 2014;14: 553–554. doi:10.1016/S1473-3099(14)70794-7

55. Mitchell PD, Anastasiou E, Whelton HL, Bull ID, Pearson MP, Shillito L-M. Intestinal parasites in the Neolithic population who built Stonehenge (Durrington Walls, 2500 BCE). Parasitology. 2022;149: 1027–1033. doi:10.1017/S0031182022000476

56. Ledger ML, Grimshaw E, Fairey M, Whelton HL, Bull ID, Ballantyne R, et al. Intestinal parasites at the Late Bronze Age settlement of Must Farm, in the fens of East Anglia, UK (9th century B.C.E.). Parasitology. 2019;146: 1583–1594. doi:10.1017/S0031182019001021

57. Yeh H-Y, Prag K, Clamer C, Humbert J-B, Mitchell PD. Human intestinal parasites from a Mamluk Period cesspool in the Christian quarter of Jerusalem: Potential indicators of long distance travel in the 15th century AD. International Journal of Paleopathology. 2015;9: 69–75. doi:10.1016/j.ijpp.2015.02.003

58. Deforce K, Ledger ML, Derreumaux M, Goffette Q, Henrotay D, Pigière F, et al. Diet, hygiene and health in Roman period northern Gaul: A multidisciplinary study of a latrine from an artisan household in the vicus Orolaunum (Arlon, southern Belgium, c. 250–280

CE). Journal of Archaeological Science: Reports. 2021;35: 102761. doi:10.1016/j.jasrep.2020.102761

59. Ledger ML, Rowan E, Marques FG, Sigmier JH, Šarkić N, Redžić S, et al. Intestinal parasitic infection in the Eastern Roman Empire during the Imperial Period and Late Antiquity. Am J Archaeol. 2020;124: 631–657. doi:10.3764/aja.124.4.0631

60. Williams FS, Arnold-Foster T, Yeh H-Y, Ledger ML, Baeten J, Poblome J, et al. Intestinal parasites from the 2nd-5th century AD latrine in the Roman Baths at Sagalassos (Turkey). Int J Paleopathol. 2017;19: 37–42. doi:10.1016/j.ijpp.2017.09.002

61. Ledger ML, Stock F, Schwaiger H, Knipping M, Brückner H, Ladstätter S, et al. Intestinal parasites from public and private latrines and the harbour canal in Roman Period Ephesus, Turkey (1st c. BCE to 6th c. CE). Journal of Archaeological Science: Reports. 2018;21: 289–297. doi:10.1016/j.jasrep.2018.07.013

62. Knorr DA, Smith WPW, Ledger ML, Peña-Chocarro L, Pérez-Jordà G, Clapés R, et al. Intestinal parasites in six Islamic medieval period latrines from 10th–11th century Córdoba (Spain) and 12th–13th century Mértola (Portugal). International Journal of Paleopathology. 2019;26: 75–83. doi:10.1016/j.ijpp.2019.06.004

63. Roche K, Pacciani E, Bianucci R, Le Bailly M. Assessing the parasitic burden in a Late Antique Florentine emergency burial site. Korean J Parasitol. 2019;57: 587–593. doi:10.3347/kjp.2019.57.6.587

64. Mitchell PD, Yeh H-Y, Appleby J, Buckley R. The intestinal parasites of King Richard III. Lancet. 2013;382: 888. doi:10.1016/S0140-6736(13)61757-2

65. Oh K-S, Kim G-T, Ahn K-S, Shin S-S. Effects of disinfectants on larval development of *Ascaris suum* eggs. Korean J Parasitol. 2016;54: 103–107. doi:10.3347/kjp.2016.54.1.103

66. Maurelli MP, Alves LC, Aggarwal CS, Cociancic P, Levecke B, Cools P, et al. *Ascaris lumbricoides* eggs or artefacts? A diagnostic conundrum. Parasitology. 2021;148: 1554–1559. doi:10.1017/S0031182021001256

67. Jónsson H, Ginolhac A, Schubert M, Johnson PLF, Orlando L. mapDamage2.0: fast approximate Bayesian estimates of ancient DNA damage parameters. Bioinformatics. 2013;29: 1682–1684. doi:10.1093/bioinformatics/btt193

68. Garcia LS. Diagnostic Medical Parasitology. Washington, DC: ASM Press; 2016.

69. Maicher C, Hoffmann A, Côté NML, Palomo Pérez A, Saña Segui M, Le Bailly M. Paleoparasitological investigations on the Neolithic lakeside settlement of La Draga (Lake Banyoles, Spain). Holocene. 2017;27: 1659–1668. doi:10.1177/0959683617702236

70. Liu G-H, Wu C-Y, Song H-Q, Wei S-J, Xu M-J, Lin R-Q, et al. Comparative analyses of the complete mitochondrial genomes of *Ascaris lumbricoides* and *Ascaris suum* from humans and pigs. Gene. 2012;492: 110–116. doi:10.1016/j.gene.2011.10.043

71. Søe MJ, Kapel CMO, Nejsum P. *Ascaris* from humans and pigs appear to be reproductively isolated species. PLoS Negl Trop Dis. 2016;10: e0004855. doi:10.1371/journal.pntd.0004855

72. Marciniak S, Klunk J, Devault A, Enk J, Poinar HN. Ancient human genomics: the methodology behind reconstructing evolutionary pathways. J Hum Evol. 2015;79: 21–34. doi:10.1016/j.jhevol.2014.11.003

73. Pedersen MW, Ginolhac A, Orlando L, Olsen J, Andersen K, Holm J, et al. A comparative study of ancient environmental DNA to pollen and macrofossils from lake sediments reveals taxonomic overlap and additional plant taxa. Quat Sci Rev. 2013;75: 161–168. doi:10.1016/j.quascirev.2013.06.006

74. Rawlence NJ, Lowe DJ, Wood JR, Young JM, Churchman GJ, Huang Y-T, et al. Using palaeoenvironmental DNA to reconstruct past environments: progress and prospects. J Quat Sci. 2014;29: 610–626. doi:10.1002/jqs.2740

75. Parducci L, Väliranta M, Salonen JS, Ronkainen T, Matetovici I, Fontana SL, et al. Proxy comparison in ancient peat sediments: pollen, macrofossil and plant DNA. Philos Trans R Soc Lond B Biol Sci. 2015;370: 20130382. doi:10.1098/rstb.2013.0382

76. Phuphisut O, Poodeepiyasawat A, Yoonuan T, Watthanakulpanich D, Chotsiri P, Reamtong O, et al. Transcriptome profiling of male and female *Ascaris lumbricoides* reproductive tissues. Parasit Vectors. 2022;15: 477. doi:10.1186/s13071-022-05602-2

77. Hansen EP, Tejedor AM, Thamsborg SM, Alstrup Hansen TV, Dahlerup JF, Nejsum P. Faecal egg counts and expulsion dynamics of the whipworm, *Trichuris trichiura* following self-infection. J Helminthol. 2016;90: 298–302. doi:10.1017/S0022149X1500019X

78. Cai P, Huang Q, Zhang X, Chen H. Adsorption of DNA on clay minerals and various colloidal particles from an Alfisol. Soil Biol Biochem. 2006;38: 471–476. doi:10.1016/j.soilbio.2005.05.019

79. Saeki K, Sakai M, Wada S-I. DNA adsorption on synthetic and natural allophanes. Appl Clay Sci. 2010;50: 493–497. doi:10.1016/j.clay.2010.09.015

80. Kanbar HJ, Olajos F, Englund G, Holmboe M. Geochemical identification of potential DNA-hotspots and DNA-infrared fingerprints in lake sediments. Appl Geochem. 2020;122: 104728. doi:10.1016/j.apgeochem.2020.104728

81. Kjær KH, Winther Pedersen M, De Sanctis B, De Cahsan B, Korneliussen TS, Michelsen CS, et al. A 2-million-year-old ecosystem in Greenland uncovered by environmental DNA. Nature. 2022;612: 283–291. doi:10.1038/s41586-022-05453-y

82. Mitchell PD. The Roman World. In: Mitchell PD, editor. Parasites in Past Civilizations and their Impact Upon Health. Cambridge: Cambridge University Press; 2023. pp. 53–64.

83. Ledger ML, Mitchell PD. Tracing zoonotic parasite infections throughout human evolution. Int J Osteoarchaeol. 2022;32: 553–564. doi:10.1002/oa.2786

84. Reinhard KJ. Parasitology as an interpretive tool in archaeology. Am Antiq. 1992;57: 231–245. doi:10.2307/280729

85. Else KJ, Keiser J, Holland CV, Grencis RK, Sattelle DB, Fujiwara RT, et al. Whipworm and roundworm infections. Nat Rev Dis Primers. 2020;6: 44. doi:10.1038/s41572-020-0171-3

Supporting Information

S1 Appendix. Appendix including materials, methods, and parasite literature review.

S2 Appendix. Appendix including parasite bait accession numbers and bait description.

S3 Appendix. Detailed results.

Figure 1

Click here to access/download;Figure;Fig1_SiteLocation.tiff

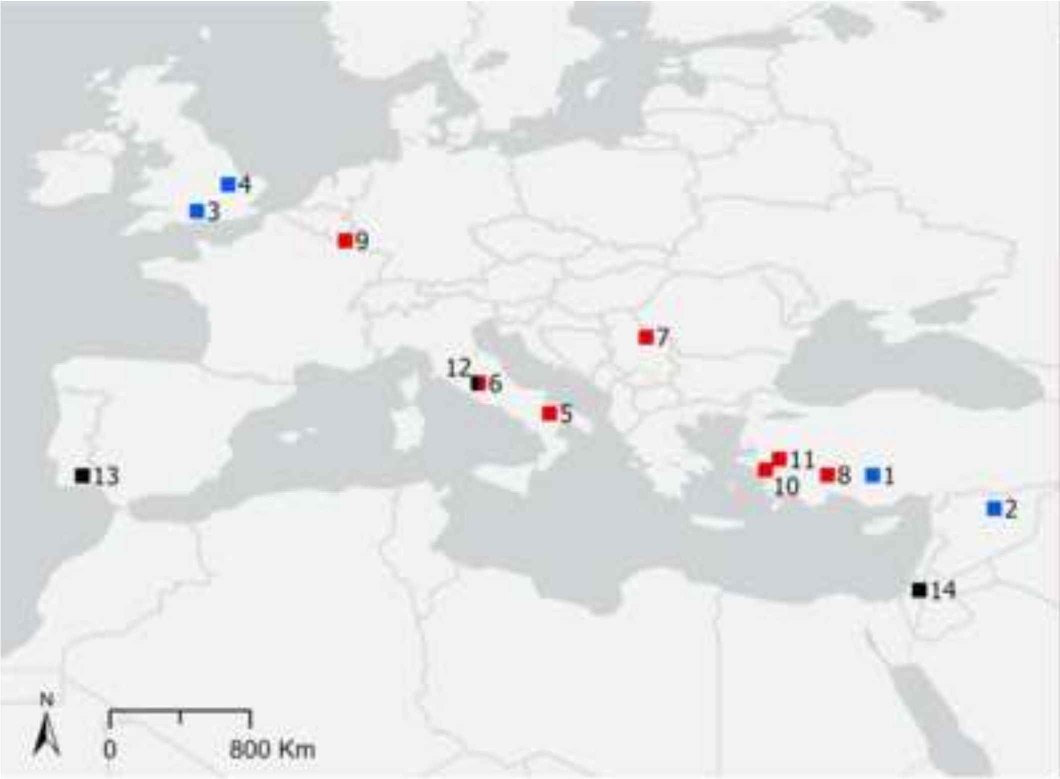

Time Period	Map Label	Site Name	Date	Sample Type
Pre-Roman	1	Çatalhöyük	6410–6150 BCE	Coprolite
	2	Tell Zeidan	4550–4050 BCE	Pelvic soil
	3	Durrington Walls	c. 2500 BCE	Coprolite
	4	Must Farm	865–840 cal BCE	Coprolite, Occupation sediment
Roman	5	Vagnari	1st–4th c. CE	Drain
	6	Vacone	2nd c. BCE– 3rd c. CE	Drain, Latrine
	7	Viminacium	2nd–3rd c. CE	Coprolite
	8	Sagalassos	2nd–5th c. CE	Latrine
	9	Arlon	250–280 CE	Latrine
	10	Ephesus	3rd–6th c. CE	Latrine
	11	Sardis	6th–7th c. CE	Drain
Post-Roman	12	Vacone	7th–8th c. CE	Pelvic soil
	13	Mértola	12th–13th c. CE	Latrine
	14	Jerusalem	15th–16th c. CE	Latrine, Coprolite

Figure 2

Click here to access/download;Figure;Fig2_ResultsFigure.png

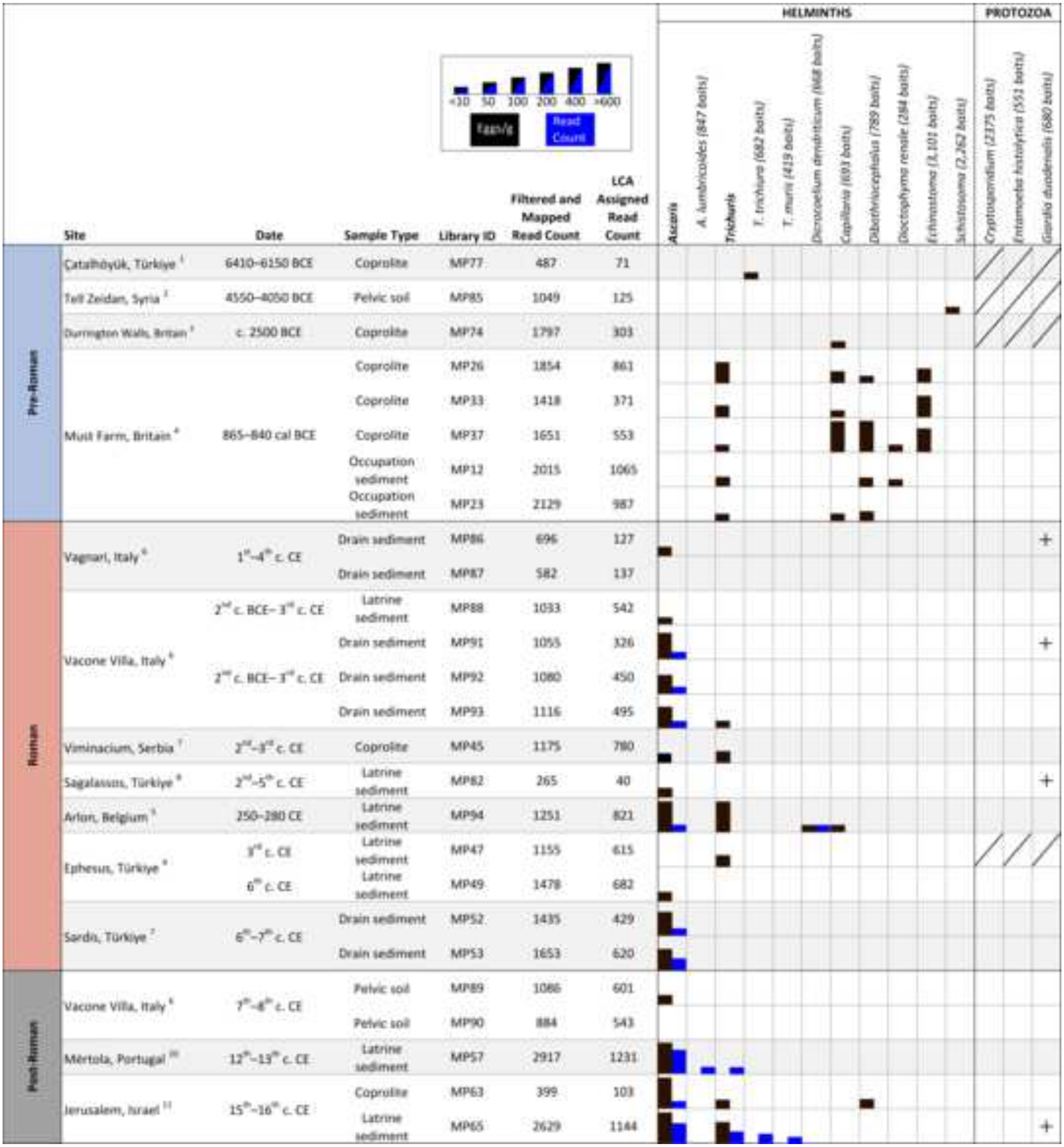

Figure 3

Click here to access/download;Figure;Fig3_HeatMap.tiff

Site	Sample Type	DNA Library ID	*Ascaris* (eggs per gram)	% LCA Assigned (Ascaridoidea or Lower)	Read Count (Ascaridoidea or Lower)
Jerusalem	Cesspit sediment	MP65	1580	21.8%	249
Mértola	Latrine	MP57	1110	24.1%	297
Arlon	Latrine	MP94	990	0.5%	4
Jerusalem	Coprolite	MP63	595	5.8%	6
Vacone	Drain sediment	MP91	405	1.8%	6
Sardis	Drain sediment	MP52	305	1.2%	5
Sardis	Drain sediment	MP53	205	9.7%	60
Vacone	Drain sediment	MP93	185	1.4%	7
Vacone	Drain sediment	MP92	140	1.6%	7
Vacone	Pelvic soil	MP89	30	0.0%	0
Vagnari	Drain sediment	MP86	25	2.4%	3
Ephesus	Latrine	MP49	25	0.0%	0
Sagalassos	Latrine	MP82	20	0.0%	0
Vacone	Latrine	MP88	5	0.0%	0

Figure 4

Click here to access/download;Figure;Fig4_mapDamage.tiff

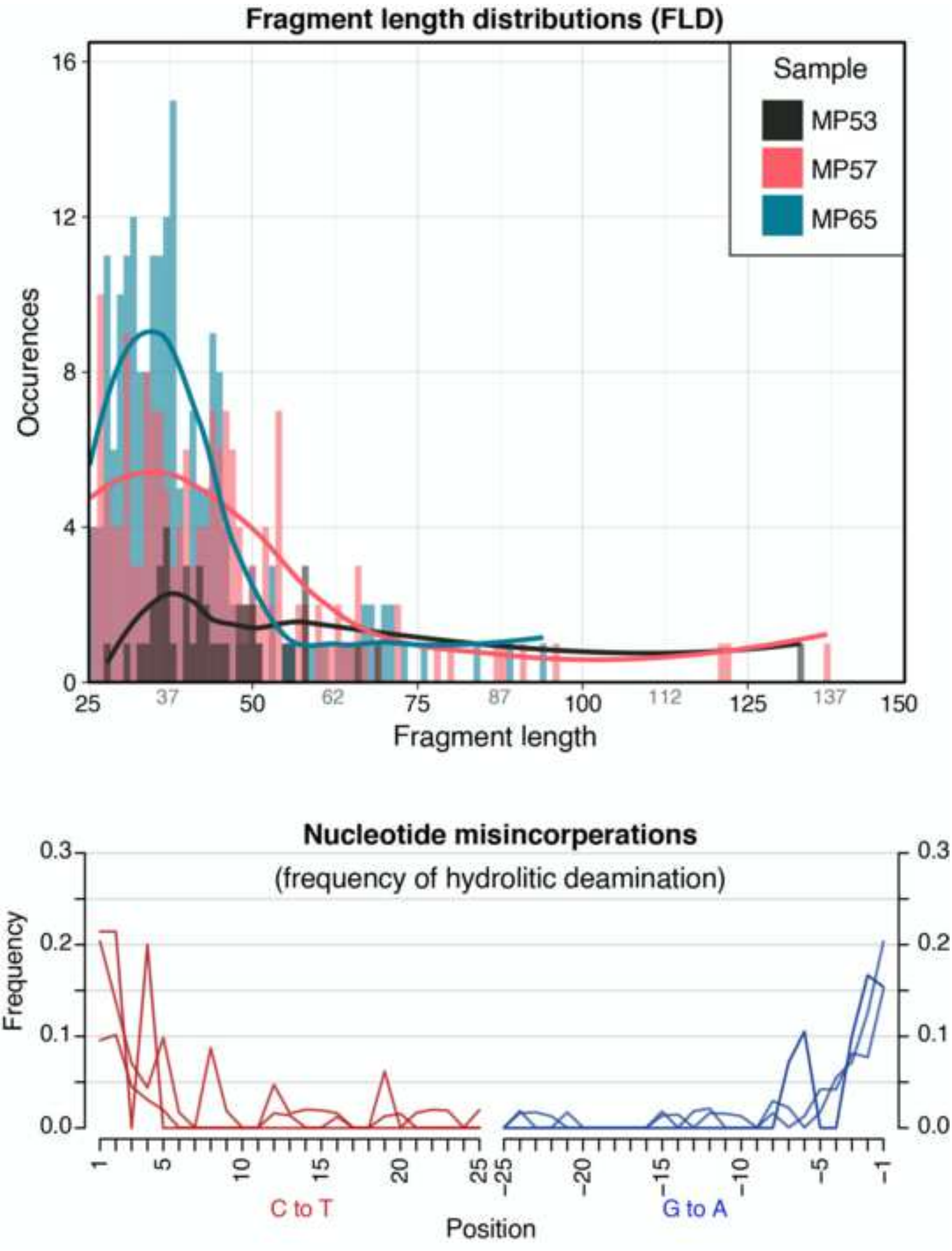

Figure 5

Click here to access/download;Figure;Fig5_Ascaris.png

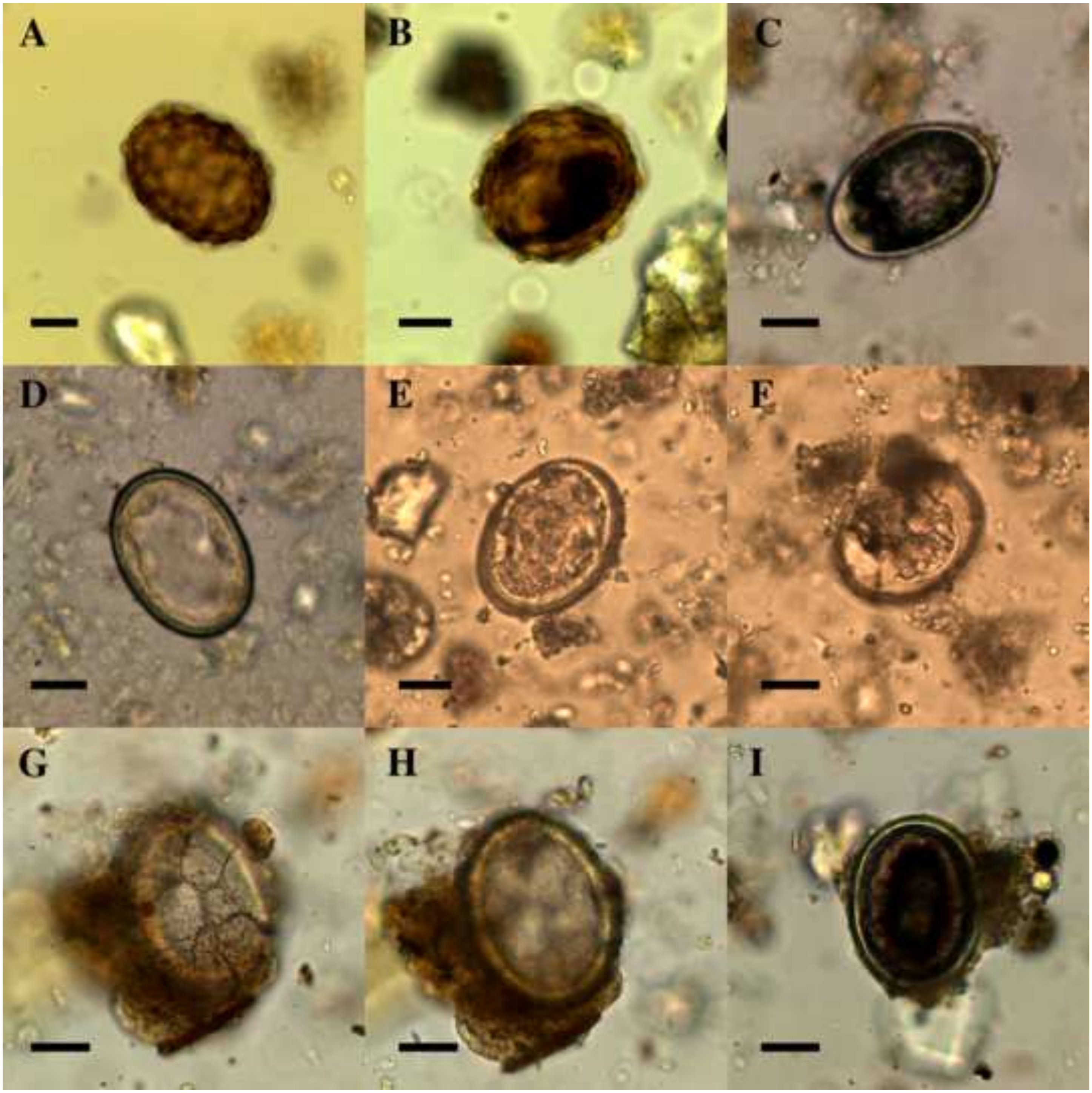

Figure 6

Click here to access/download;Figure;Fig6_TemporalChanges.png

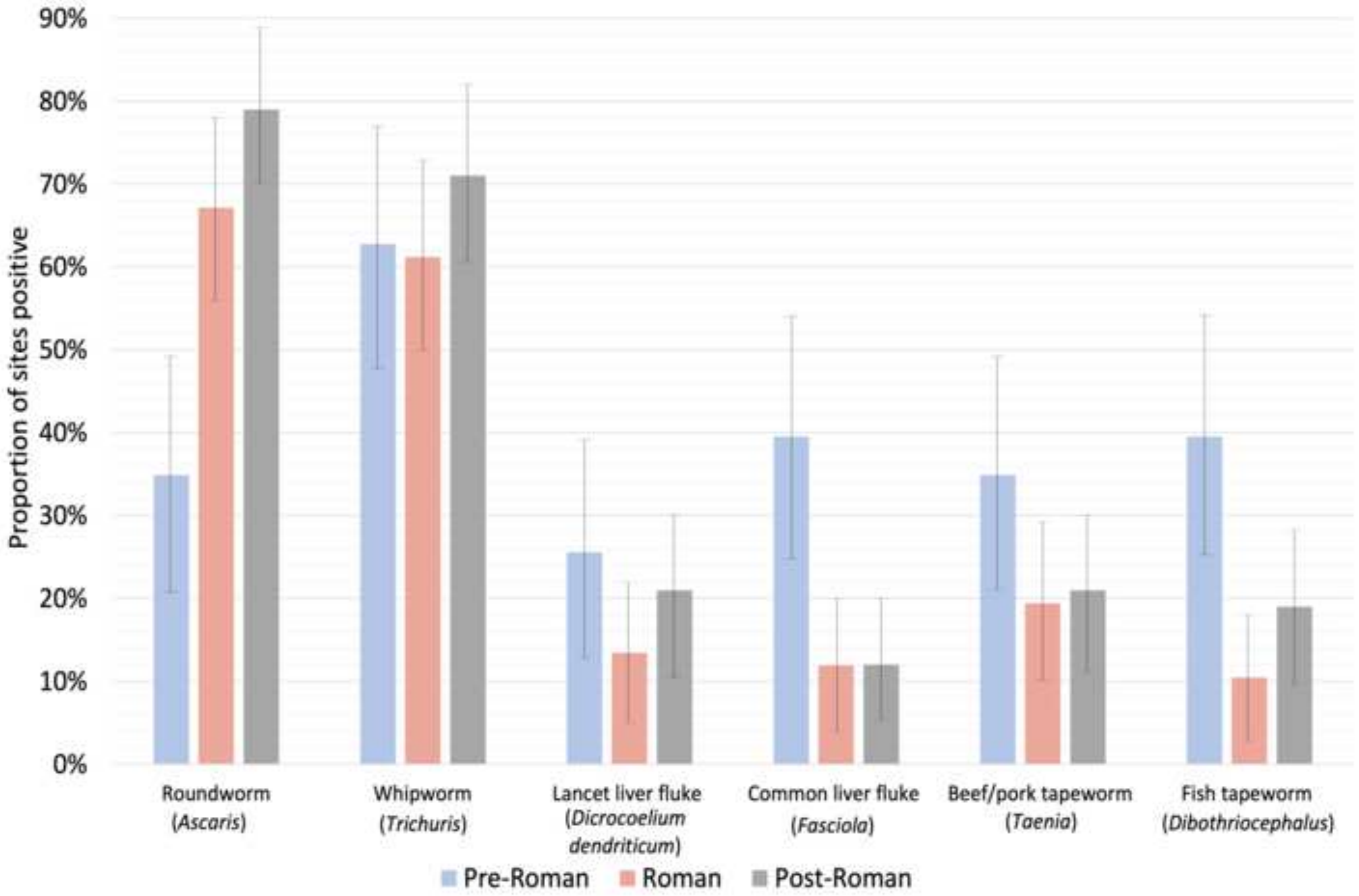

Click here to access/download
Supporting Information
S1_Appendix_Materials_Methods_TemporalData_RefsFormatted.docx

Click here to access/download
Supporting Information
S2_Appendix_ParasiteBaits.xlsx

Click here to access/download
Supporting Information
S3_Appendix_Results.xlsx
