# Supplementary material for: Sedimentary ancient DNA as part of a multimethod paleoparasitology approach reveals temporal trends in human parasitic burden in the Roman period
Source: PLoS Negl Trop Dis. 2025 Jun 10;19(6):e0013135. doi: 10.1371/journal.pntd.0013135 (PMC12151383; doi:10.1371/journal.pntd.0013135)
Supplement: Attachment — Submitted filename: PNTD-D-24-01526_R1_reviewer AMI2.pdf [file pntd.0013135.s006.pdf]

PLOS Neglected Tropical Diseases

Sedimentary ancient DNA as part of a multimethod paleoparasitology approach reveals temporal trends in human parasitic burden in the Roman period

--Manuscript Draft--

**Manuscript Number:**	PNTD-D-24-01526R1
**Full Title:**	Sedimentary ancient DNA as part of a multimethod paleoparasitology approach reveals temporal trends in human parasitic burden in the Roman period
**Short Title:**	Sedimentary ancient DNA as part of a multimethod paleoparasitology approach
**Article Type:**	Research Article
**Keywords:**	ancient DNA; sedimentary DNA; ELISA; microscopy; helminths; protozoa; Roman Empire
**Abstract:**	The detection of parasite infections in past populations has classically relied on microscopic analysis of sediment samples and coprolites. In recent years, additional methods have been integrated into paleoparasitology such as, enzyme-linked immunosorbent assay (ELISA) and ancient DNA (aDNA). The aim of this study was to evaluate a multimethod approach for paleoparasitology using microscopy, ELISA, and sedimentary ancient DNA (sedaDNA) with a parasite-specific targeted capture approach and high-throughput sequencing. Using 26 samples dating from c. 6400 BCE to 1500 CE that were previously analyzed with microscopy and ELISA, we aimed to more accurately detect and reconstruct parasite diversity in the Roman Empire and compare this diversity to earlier and later time periods to explore temporal changes in parasite diversity. Microscopy was found to be the most effective technique for identifying the eggs of helminths, with 8 taxa identified. ELISA was the most sensitive for detecting protozoa that cause diarrhea (notably Giardia duodenalis). Parasite DNA was recovered from 9 samples, with no parasite DNA recovered from any pre-Roman sites. Sedimentary DNA analysis identified whipworm at a site where only roundworm was visible on microscopy, and also revealed that the whipworm eggs at another site came from two different species (T. trichiura and T. muris). Our results show that a multimethod approach provides the most comprehensive reconstruction of parasite diversity in past populations. In the pre-Roman period, taxonomic diversity included a mixed spectrum of zoonotic parasites, together with whipworm, which is spread by ineffective sanitation. We see a marked change during the Roman and medieval periods with an increasing dominance of parasites transmitted by ineffective sanitation, especially roundworm, whipworm and protozoa that cause diarrheal illness.
**Additional Information:**	
**Question**	**Response**
**Government Employee**<br><br>Are you or any of the contributing authors an employee of the United States government?<br><br>Manuscripts authored by one or more US Government employees are not copyrighted, but are licensed under a CC0 Public Domain Dedication, which allows unlimited distribution and reuse of the article for any lawful purpose. This is a legal requirement for US Government employees.	No - No authors are employees of the U.S. government.

This will be typeset if the manuscript is accepted for publication.	
**Financial Disclosure**<br><br>Enter a financial disclosure statement that describes the sources of funding for the work included in this submission. Review the submission guidelines for detailed requirements. View published research articles from *PLOS Neglected Tropical Diseases* for specific examples.<br><br>This statement is required for submission and **will appear in the published article** if the submission is accepted. Please make sure it is accurate.<br><br>**Funded studies**<br>Enter a statement with the following details:<br>• Initials of the authors who received each award<br>• Grant numbers awarded to each author<br>• The full name of each funder<br>• URL of each funder website<br>• Did the sponsors or funders play any role in the study design, data collection and analysis, decision to publish, or preparation of the manuscript?<br><br>Did you receive funding for this work?	Yes
Please add funding details.<br>as follow-up to "**Financial Disclosure**<br><br>Enter a financial disclosure statement that describes the sources of funding for the work included in this submission. Review the submission guidelines for detailed requirements. View published research articles from *PLOS Neglected Tropical Diseases* for specific examples.<br><br>This statement is required for submission and **will appear in the published article** if the submission is accepted. Please make sure it is accurate.	This work was supported by the Social Sciences and Humanities Research Council of Canada (752-2016-2085 to MLL), a Tidmarsh Cambridge Scholarship from the Cambridge Commonwealth, European and International Trust and Trinity Hall College, and the Society for the Promotion of Roman Studies (to MLL), a CANA Foundation grant (to TJM and HNP), the Garfield Weston Foundation (to TJM), the Social Sciences and Humanities Research Council of Canada (767-2016-2288 to TJM), the Canadian Institute for Advanced Research, and the Natural Sciences and Engineering Research Council of Canada (to HP). The funders had no role in study design, data collection and analysis, decision to publish, or preparation of the manuscript.

Powered by Editorial Manager® and ProduXion Manager® from Aries Systems Corporation

**Funded studies**<br>Enter a statement with the following details:<br>• Initials of the authors who received each award<br>• Grant numbers awarded to each author<br>• The full name of each funder<br>• URL of each funder website<br>• Did the sponsors or funders play any role in the study design, data collection and analysis, decision to publish, or preparation of the manuscript?<br><br>Did you receive funding for this work?"	
Please select the country of your main research funder (please select carefully as in some cases this is used in fee calculation).<br>as follow-up to "**Financial Disclosure**<br><br>Enter a financial disclosure statement that describes the sources of funding for the work included in this submission. Review the submission guidelines for detailed requirements. View published research articles from *PLOS Neglected Tropical Diseases* for specific examples.<br><br>This statement is required for submission and **will appear in the published article** if the submission is accepted. Please make sure it is accurate.<br><br>**Funded studies**<br>Enter a statement with the following details:<br>• Initials of the authors who received each award<br>• Grant numbers awarded to each author<br>• The full name of each funder<br>• URL of each funder website<br>• Did the sponsors or funders play any role in the study design, data collection and analysis, decision to publish, or preparation of the manuscript?<br><br>Did you receive funding for this work?"	CANADA - CA
**Competing Interests**	The authors have declared that no competing interests exist

On behalf of all authors, disclose any competing interests that could be perceived to bias this work.<br><br>This statement will be typeset if the manuscript is accepted for publication.<br><br>Review the instructions link below and PLOS NTDs' competing interests policy to determine what information must be disclosed at submission.	
**Data Availability**<br><br>Provide a **Data Availability Statement** in the box below. This statement should detail where the data used in this submission can be accessed. This statement will be typeset if the manuscript is accepted for publication.<br><br>Before publication, authors are required to make all data underlying their findings fully available, without restriction. Review our PLOS Data Policy page for detailed information on this policy. Instructions for writing your Data Availability statement can be accessed via the Instructions link below.	The data that support the findings of this study are publicly available from the NCBI Sequence Read Archive with the accession PRJNA1194279.

Powered by Editorial Manager® and ProduXion Manager® from Aries Systems Corporation

Manuscript

Click here to access/download;Manuscript;Multimethod_Approach_Palaeopar

Sedimentary ancient DNA as part of a multimethod paleoparasitology approach reveals temporal trends in human parasitic burden in the Roman period

Marissa L. Ledger[1,2*#], Tyler J. Murchie[2,3], Zachery Dickson[4], Melanie Kuch[2], Scott D. Haddow[5], Christopher J. Knüsel[6], Gil J. Stein[7], Mike Parker Pearson[8], Rachel Ballantyne[9], Mark Knight[10], Koen Deforce[11,12], Maureen Carroll[13], Candace Rice[14], Tyler Franconi[14], Nataša Šarkić[15], Saša Redžič[16], Erica Rowan[17], Nicholas Cahill[18], Jeroen Poblome[19], Maria de Fátima Palma[20], Helmut Brückner[21], Piers D. Mitchell[1], Hendrik Poinar[2,22,23]

[1] Department of Archaeology, The Henry Wellcome Building, University of Cambridge, Cambridge, UK

[2] McMaster Ancient DNA Centre, Department of Anthropology, McMaster University, Hamilton, Ontario, Canada

[3] Hakai Institute, Heriot Bay, British Columbia, Canada

[4] Department of Biology, McMaster University, Hamilton, Ontario, Canada

[5] Department of Cross-Cultural and Regional Studies, University of Copenhagen, Copenhagen, Denmark

[6] CNRS, MC, PACEA, UMR 5199, Université de Bordeaux, Pessac, France

[7] Institute for the Study of Ancient Cultures (ISAC), University of Chicago, Chicago, Illinois, USA

[8] Institute of Archaeology, University College London, London, UK

[9] McDonald Institute for Archaeological Research, University of Cambridge, Cambridge, UK

[10] Cambridge Archaeological Unit, Department of Archaeology, University of Cambridge, Cambridge, UK

[11] Department of Archaeology, Ghent University, Ghent, Belgium

[12] Royal Belgian Institute of Natural Sciences, Brussels, Belgium

[13] Department of Archaeology, University of York, York, UK

[14] Joukowsky Institute for Archaeology & the Ancient World and Department of Classics, Brown University, Providence, Rhode Island, USA

[15] Aita Bioarchaeology, Barcelona, Spain

[16] Institute of Archaeology, Belgrade, Serbia
[17] Department of Classics, Royal Holloway, University of London, UK
[18] Department of Art History, University of Wisconsin–Madison, Wisconsin, USA
[19] Unit of Archaeology, KU Leuven, Belgium
[20] Campo Arqueológico de Mértola/CEAACP, Mértola, Portugal
[21] Institute of Geography, University of Cologne, Cologne (Köln), Germany
[22] Department of Biochemistry and Biomedical Sciences, McMaster University, Hamilton, Ontario, Canada
[23] Michael G. DeGroote Institute for Infectious Disease Research, McMaster University, Hamilton, Ontario, Canada

[#] Current Address: Department of Pathology and Molecular Medicine, McMaster University, Hamilton, Ontario, Canada

* Corresponding author
E-mail: ledgerm@mcmaster.ca (MLL)

Abstract

The detection of parasite infections in past populations has classically relied on microscopic analysis of sediment samples and coprolites. In recent years, additional methods have been integrated into paleoparasitology such as, enzyme-linked immunosorbent assay (ELISA) and ancient DNA (aDNA). The aim of this study was to evaluate a multimethod approach for paleoparasitology using microscopy, ELISA, and sedimentary ancient DNA (sedaDNA) with a parasite-specific targeted capture approach and high-throughput sequencing. Using 26 samples dating from c. 6400 BCE to 1500 CE that were previously analyzed with microscopy and ELISA, we aimed to more accurately detect and reconstruct parasite diversity in the Roman Empire and compare this diversity to earlier and later time periods to explore temporal changes in parasite diversity. Microscopy was found to be the most effective technique for identifying the eggs of helminths, with 8 taxa identified. ELISA was the most sensitive for detecting protozoa that cause diarrhea (notably *Giardia duodenalis*). Parasite DNA was recovered from 9 samples, with no parasite DNA recovered from any pre-Roman sites. Sedimentary DNA analysis identified whipworm at a site where only roundworm was visible on microscopy, and also revealed that the whipworm eggs at another site came from two different species (Trichuris ~~*T.*~~ *trichiura* and *T. muris*). Our results show that a multimethod approach provides the most comprehensive reconstruction of parasite diversity in past populations. In the pre-Roman period, taxonomic diversity included a mixed spectrum of zoonotic parasites, together with whipworm, which is spread by ineffective sanitation. We see a marked change during the Roman and medieval periods with an increasing dominance of parasites transmitted by ineffective sanitation, especially roundworm, whipworm and protozoa that cause diarrheal illness.

Keywords: Ancient DNA, sedimentary DNA, ELISA, microscopy, helminths, protozoa, Roman Empire

Author Summary

A multimethod approach in paleoparasitology, combining microscopy, ELISA and aDNA, provides a more complete reconstruction of past parasite diversity. Applying this multimethod approach to a diverse set of archeological sediment samples from 6400 BCE to 1500 CE provided insights into temporal changes in parasite diversity in the human past. Microscopy can be used as an effective screening tool for helminths in paleofecal samples, while ELISA is necessary for detection of protozoa, and sedaDNA using targeted enrichment can identify additional taxa and confirm species identification. Targeted enrichment using a comprehensive parasite bait set allows for detection of ancient human parasites and recovery of ancient parasite DNA from as little as 0.25 g of sediment. This multimethod approach reveals that parasite diversity decreased in the Roman period due to a decrease in zoonotic parasites and concurrent increase in fecal-oral parasites, a pattern which is consistent during the medieval period.

Introduction

Ancient DNA (aDNA) has increasingly been used to improve the detection and characterization of ancient human pathogens [1–4]. Paleogenetic studies of ancient human pathogens have aided in the determination of the causative agents of past epidemics and endemic diseases [3,5,6], identified extinct strains of bacteria and viruses [7,8], and reconstructed ancient genomes of these organisms, allowing for the calibration of mutation rates and investigation of evolutionary histories such as changes in virulence genes [9,10]. However, many infectious diseases, including most parasites, cannot be detected using skeletal remains which are commonly used in aDNA studies. Parasites contributed to a significant burden of disease in the past, however our ability to detect and study their evolutionary history has been hampered by difficulty accessing their DNA. Paleofeces (preserved fecal material) is the best source for recovering preserved DNA from enteric pathogens including bacteria [11,12], parasites [13–15], and viruses [16,17]. The field of paleoparasitology has a long history of using sediment samples to study enteric parasites in past populations [18]. Samples used in

paleoparasitology include coprolites (mineralized feces), pelvic sediment from burials where the intestines decomposed, and sediment containing high amounts of human fecal material such as the fill of latrines and sewer drains. Using these samples to recover genetic material and human gastrointestinal pathogens opens another route for understanding past human health, disease, and lifeways, and provides insight into a large category of pathogens that cannot be diagnosed using skeletal remains.

The use of genetic methods to detect human-infecting parasites in both clinical and ancient samples has lagged behind most other pathogens [19,20]. The main methods used in paleoparasitology are microscopy, and to a smaller extent, enzyme-linked immunosorbent assay (ELISA, an immunological method that can detect antigens from specific organisms of interest). In recent years, the number of studies using aDNA to detect human-infecting parasites has increased (see [20] for a review; and subsequent studies [12,13,15,21–30]). However, aDNA analysis contributes only a small minority of the total investigations performed within paleoparasitology and many studies still rely on a small number of PCR targets.

Here we present a multimethod approach for recovery of ancient parasites using sediment samples from contexts known to contain fecal material, including latrines, sewers, soil from the pelvic area of skeletons, and coprolites. Our multimethod approach incorporates sedaDNA methods using a targeted enrichment approach for parasite-specific DNA, in conjunction with microscopy and ELISA. The aim of this multimethod approach was to generate a more complete understanding of parasite diversity in Europe and the Eastern Mediterranean during the Roman Empire as compared to pre- and post-Roman periods. We analyzed paleofecal samples originating from archeological sites temporally spanning the Neolithic through the medieval period. We used sedaDNA extraction methods previously shown to increase aDNA recovery by 7–20 fold compared to commercial kits [31], paired with targeted enrichment in order to preferentially sequence parasite DNA of interest and avoid the high sequencing costs associated with deep shotgun sequencing for low abundance targets such as pathogenic organisms. This sedaDNA approach was originally designed for paleoecological reconstructions from permafrost [31]—here we show that this approach can also be used to recover the aDNA of human parasites from archeological sediments. Similarly, we show that a

multimethod approach to paleoparasitology results in a more comprehensive taxonomic recovery compared to only utilizing a single method. Applying this multimethod approach to paleofecal samples from the Neolithic through medieval periods reveals trends in parasite infection that demonstrate marked change in the dominant species during the Roman period.

Materials and Methods

Materials

We analyzed archeological sediments from latrine fill, drain fill, coprolites, and soil from the pelvic area of skeletons. All samples came from human contexts in archeological sites ranging from the Neolithic period (ca. 6400 BCE) through the medieval period (ca. 1500 CE) in regions that were at one time part of the Roman Empire (see Fig 1). A total of 14 sites were included in the study; four from the pre-Roman period, seven from the Roman period, and three from the post-Roman or medieval period. From some sites multiple samples were studied if available, thus a total of 26 samples were included (see Fig 2). Further information about each site and samples analyzed can be found in S1 Appendix.

< **Fig 1. Map of archeological sites studied.** Map indicates the location of sediment samples studied. The table on the right provides the site name, date of samples, and sample types. Map created using ArcGIS software by Esri, basemap available at https://arcg.is/efffD. ArcGIS and ArcMap are the intellectual property of Esri and are used herein under license. Copyright Esri. All rights reserved. Sources: Esri, TomTom, Garmin, FAO, NOAA, USGS, OpenStreetMap contributors, and the GIS User Community.>

Methods

All samples were first studied and reported on using microscopy and ELISA following standard methods [32,33]. The samples selected for this study had previously yielded parasite eggs or came from sites where eggs were found in contextually associated samples [33–43]. For microscopy, a 0.2 g subsample was disaggregated in 0.5% trisodium phosphate. This was then

microsieved to collect material between 20 and 160 µm. This fraction of the subsample was mixed with glycerol and viewed under a light microscope (Olympus BX40F) at 200x and 400x magnification to identify preserved helminth eggs based on morphological characteristics.

For ELISA, a 1 g subsample was disaggregated in 0.5% trisodium phosphate and microsieved. Given the smaller size of protozoan cysts (less than 20 µm), the material in the catchment container below the 20 µm sieve was collected and concentrated to be used in commercial ELISA kits following the manufacturer's protocols. The kits used were *GIARDIA II*, *E. HISTOLYTICA II*, and *CRYPTOSPORIDIUM II* from TECHLAB, Inc (Blacksburg, USA). These kits are designed for detection of *Giardia duodenalis*, *Entamoeba histolytica*, and *Cryptosporidium* spp. in modern human fecal samples. These kits and those from other manufacturers have been used to detect protozoan antigens in ancient human fecal samples in many previous studies [33,44–47].

For sedaDNA, all work was done in dedicated ancient DNA facilities. No modern molecular work is performed in these labs. A unidirectional workflow is followed with work starting in the reagent cleanroom and proceeding through extraction rooms followed by amplification rooms. Standard precautions to prevent lab and sample contamination in aDNA were followed including wearing full suit, gloves, and mask. All surfaces are regularly cleaned with 6% sodium hypochlorite and hoods are exposed to UV radiation. The sedaDNA methods used are described below, for further details see S1 Appendix.

First, 0.25 g of material was subsampled. Organic and inorganic material was chemically and physically disintegrated to release DNA using a lysis buffer (see S1 Appendix for reagents and concentrations) placed with the subsample in garnet PowerBead tubes (Qiagen) already containing 750 µL 181 mM $NaPO_4$ and 121 mM guanidinium isothiocyanate with garnet beads for physical disruption [31]. The samples were vortexed for 15 minutes to mechanically break down the organo-mineralized content and parasite eggs. Bead beating has been shown in clinical and archeological studies to improve DNA recovery by breaking parasite eggs [48,49]. Proteinase K was added after bead beating, and tubes were continuously rotated in an oven set to 35° C overnight. The supernatant was removed and mixed with high-volume Dabney binding buffer as per published protocols [50]. Samples were then centrifuged at 4500 rpm at 4° C

following Murchie et al. [31] for a minimum of 6 hours to remove inhibitors; if supernatant was clear at this point, the sample was removed from the centrifuge, and if not, it was centrifuged further for a total of 24 hours. Centrifugation at refrigerated temperatures has been shown to increase the recovery of sedaDNA from complex environmental sample types through the precipitation of enzymatic inhibitory compounds commonly found in sediment and fecal samples [31]. The remainder of the extraction and purification followed Dabney et al. [50] with the binding buffer passed through silica columns and eluted in 50 µL elution buffer.

DNA libraries were prepared for Illumina sequencing using a double-stranded method [51,52] with minor modifications for blunt end repair as per our laboratory protocols (see S1 Appendix). A subset of four libraries (MP12, MP26, MP37, and MP53) were indexed separately for shotgun sequencing with a targeted sequencing depth of 2 million reads per sample. Targeted enrichment for parasite DNA was carried out using in-solution hybridization capture with RNA baits designed using modern parasite genomes (see S2 Appendix for reference sequence accession numbers). The parasite bait set contained 30,240-75mer baits based on mitochondrial and nuclear gene sequences retrieved from GenBank NCBI [53,54] from a list of identified human and animal infecting parasites and protozoa. Tiled baits were designed to capture 121 parasite taxa (see S2 Appendix). A progressive tiling approach was used to balance the number of baits for each taxa as best as possible. With this approach the bait design algorithm determined the maximum and minimum tiling density possible for each bait region. Taxa with few bait regions were maximally tiled to increase bait number while taxa with many potential bait regions were minimally tiled to balance bait number per taxa. The final average tiling density per taxa ranged from 2x to 30x. The number of baits for each taxa was ultimately limited by reference sequences available and ranged from 2–922 baits per taxa. Baits were purchased from Daicel Arbor Biosciences and our enrichment protocol followed the MYbaits V4 protocol supplied by the manufacturer.

Libraries for shotgun sequencing and targeted enrichment were quantified using qPCR and pooled to equimolar concentrations based on the molarity of each library. Pools were size-selected using electrophoresis followed by gel excision using the QIAquick Gel Extraction Kit

(Qiagen). Paired-end sequencing was conducted using 2x90 sequencing chemistry on an Illumina HiSeq 1500 platform at the McMaster Farncombe Metagenomics Facility.

Bioinformatic workflow followed previously described in-house protocols for sedaDNA analysis (see [31]). In brief, *leeHom* [55] was used for trimming and merging, reads were then mapped using *network-aware-BWA* to the parasite reference sequences that had been used to design the bait set. These mapped reads were size filtered to those greater than or equal to 24 bp and string de-duplicated with *NGSeXplore*. Filtered and mapped reads were run through BLASTn to return the top 100 taxonomic hits. MEGAN was then used to assign these reads to the lowest common taxonomic rank. For further detail on bioinformatic workflow see S1 Appendix. Raw sequence data has been deposited in the NCBI Sequence Read Archive (PRJNA1194279).

Results

Pre-Roman Periods

Microscopic analysis of all samples described here was previously published. *Trichuris trichiura* (whipworm) was found in the human coprolite from 6410–6150 BCE Çatalhöyük, Türkiye [38]. *Schistosoma* sp. was found in pelvic soil from a burial (5800–4000 BCE) in Tell Zeidan, Syria [34]. One coprolite from the Neolithic settlement (2500 BCE) of Durrington walls near Stonehenge, Britain was included which contained Capillariid eggs [43]. This coprolite was previously identified as human in origin using fecal lipid biomarkers. Interestingly, DNA sequenced from this coprolite contained a high proportion of reads assigned to *Canis lupus familiaris* with very few reads assigned to *Homo sapiens* (see S1 Appendix). At the Bronze Age site of Must Farm in Britain (c. 850 BCE), sediment samples and human and dog coprolites were included. The source of these coprolites had also been previously identified using fecal lipid biomarkers [39]. One of the coprolites previously identified as human and one of dog origin underwent shotgun sequencing and were shown to contain high amounts of DNA from *Canis lupus familiaris* (see S1 Appendix). Microscopy of coprolites found eggs of *Capillaria* sp., *Echinostoma* sp., fish tapeworm (*Dibothriocephalus* sp.), giant kidney worm (*Dioctophyma*

renale), and whipworm (*Trichuris* sp.). Occupation layer sediment contained all of these taxa except *Echinostoma* sp. [39].

The only Pre-Roman samples studied using ELISA were those from Must Farm as there was insufficient remaining material from the other samples. The ELISA results from these samples were indeterminate as all sample wells (8 technical replicates on two separate biological replicates for each sample) were positive for all three species (*Giardia duodenalis*, *Entamoeba histolytica*, and *Cryptosporidium* spp.). This is an exceptionally unusual result for paleoparasitological studies and suggests a false positive result likely from cross-reactivity with another biomolecule within the samples. Furthermore, *Cryptosporidium* sp. has never been detected in ancient samples from Europe.

No parasite DNA was recovered from the 8 Pre-Roman samples included. Extracts were quantified using qPCR after library preparation using probes designed for the ligated sequencing adapters. The quantification of adapter-ligated molecules in these libraries (ranging from 10^6 to 10^9 copies/uL) was consistent with other samples where parasite DNA was recovered (see S1 Appendix Table D.1). Additionally, the number of filtered reads mapped to the parasite reference sequences and subsequently LCA assigned were consistent with samples from the Roman and post-Roman periods (Fig 2). Within the pre-Roman samples, 59-93% of the LCA-assigned reads were assigned to bacteria, while less than 10% of reads were assigned to Fungi, Metazoa, Sar, and Viridiplantae with the exception of one sample where 15% of reads were assigned to Fungi. Though reads assigned to Metazoa and Sar may be from parasites, these reads could not be assigned below the Clade level (e.g. Stramenopiles, Alveolata, Bilateria) thus are relatively uninformative. Thus, the majority of LCA-assigned reads in the pre-Roman samples were from bacteria and we were unable to recover parasite DNA from taxa of which eggs were preserved.

Roman

In the 13 Roman period samples, the parasite taxa recovered by microscopy were less diverse than the pre-Roman sites. Roundworm (*Ascaris* sp.) was recovered from 11 samples

studied, whipworm eggs were found in four, and both *Capillaria* sp. and *Dicrocoelium dendriticum* in one.

The only protozoa recovered from Roman period sites using ELISA was *Giardia duodenalis*. *Giardia* was detected in drain samples from Vagnari and the Villa of Vacone in Italy, and in a latrine from Sagalassos in Türkiye [33].

For aDNA analysis, reads assigned to roundworm (*Ascaris* sp.) were recovered from six samples. *Ascaris* sp. eggs were found by microscopy in all of these samples. *Dicrocoelium dendriticum* DNA was also recovered from the latrine at Arlon, Belgium; *Dicrocoelium* eggs were also found by microscopy from this site. No protozoa DNA was recovered.

Post-Roman

Similar to the Roman period, the most common helminth recovered from medieval period sites using microscopic analysis was roundworm (*Ascaris* sp.) which was found in four of five samples. Whipworm was found in two samples and fish tapeworm in one sample. The only protozoa recovered using ELISA was *Giardia duodenalis*. This was found in the latrine from Jerusalem [35].

Ancient DNA from roundworm (*Ascaris*) was found in three medieval samples studied. In the latrine sediment from Mértola, Portugal three of these reads were assigned to the species-level as *Ascaris lumbricoides*, the human infecting species of *Ascaris*, using MEGAN. However, when they were mapped to the reference genomes of both *Ascaris lumbricoides* (GCA_015227635.1) and *Ascaris suum* (GCA_013433145.1) these reads had 100% identity with both species as would be expected based on the limited genetic diversity between these species (see S1 Appendix). In addition, reads assigned to whipworm (*Trichuris* sp.) were found in the latrine from Mértola in Portugal. Interestingly, no *Trichuris* sp. eggs were seen on microscopic analysis of this latrine sediment. Species-level identification was possible for whipworm reads from the latrine in Jerusalem. Reads were not only assigned to human whipworm (*Trichuris trichiura*), but also mouse whipworm (*Trichuris muris*). These results are comparable to results of a recent aDNA study employing shotgun sequencing to characterize

microbial and eukaryotic DNA in a separate subsample from this same latrine in Jerusalem. In that study, *Ascaris lumbricoides* and *Trichuris trichiura* were detected [12].

<**Fig 2. Summary of parasites recovered from each sample using microscopy, ELISA, and ancient DNA.** Black bars represent egg concentrations identified using microscopy and blue bars represent DNA read count. Samples for which insufficient material was available for ELISA are crossed out and positive ELISA tests are marked with a +. [1] Ledger et al., 2019 [38]; [2] Anastasiou et al., 2014 [34]; [3] Mitchell et al., 2022 [43]; [4] Ledger et al., 2019 [39]; [5] Deforce et al., 2021 [42]; [6] Ledger et al., 2021 [33]; [7] Ledger et al., 2020 [41]; [8] Williams et al., 2017 [36]; [9] Ledger et al., 2018 [37]; [10] Knorr et al., 2019 [40]; [11] Yeh et al., 2015 [35].>

Expansion of Results Using Ancient DNA

Shotgun sequencing on a subset of four samples was unable to recover any identifiable parasite DNA. However, using a targeted enrichment approach, parasite DNA was recovered from 9 out of 26 samples (see Fig 2 for summarized results and S3 Appendix for results by individual sample). The majority of sequenced reads assigned to human parasites were from roundworm (*Ascaris*). Additional parasite taxa from which we were able to recover DNA included the lancet liver fluke (*Dicrocoelium dendriticum*) and whipworm (*Trichuris* spp.) (Fig 2). In one of these samples (MP57 from Mértola, Portugal) whipworm eggs were not visualized by microscopy thus, aDNA analysis provides evidence for additional taxa.

The microscopic identification of *Ascaris* was replicated using aDNA analysis in six out of a total 11 Roman period samples from which *Ascaris* eggs were identified by microscopy. Three out of five medieval period samples in which *Ascaris* eggs were identified by microscopy contained *Ascaris* DNA. From both of these time periods, only three of these samples contained *Ascaris* eggs that were sufficiently preserved to still have a visible outer mamillated coat. Mamillated *Ascaris* eggs were recovered from Arlon in Belgium, Viminacium in Serbia, and the Mamluk period cesspit in Jerusalem. The mamillated coat of *Ascaris* is an important characteristic feature used for identification of the eggs, however, it can be absent in modern eggs and is often lost in archeological contexts which makes identification challenging [56–59]. *Ascaris* DNA was detected in samples containing decorticated and poorly preserved *Ascaris* eggs as can be seen in Fig 5.

Figure 3 contains a comparison between the concentration of roundworm (*Ascaris*) eggs recovered by microscopy to the percent of LCA-assigned reads to Ascaridoidea or lower taxonomic rank and read count of the same. Unsurprisingly, the samples that contained the highest concentration of *Ascaris* eggs also had a higher percentage of reads assigned to *Ascaris*. For nearly all samples where *Ascaris* eggs were identified by microscopy but no DNA was recovered, the egg concentration was 30 eggs per gram or less.

<**Fig 3. Heatmap comparing concentration of *Ascaris* eggs recovered from each sample to percentage of LCA-assigned reads.** *Ascaris* egg concentration is reported in eggs per gram based on microscopy and LCA-assigned reads include reads that were assigned to the Superfamily Ascaridoidea or lower and read count assigned to Ascaridoidea or lower. Samples are ordered in the table based on egg per gram concentration from highest to lowest concentration with dark red background for the lowest concentrations, yellow/orange mid-range and green the highest concentrations.>

The sequence reads assigned to roundworm were authenticated as aDNA using *mapDamage* [60]. They showed typical deamination patterns for aDNA and fragment length distributions (Fig 4). Statistical analysis could not be done on reads from other taxa due to the low number of reads.

<**Fig 4. Deamination and fragment length distributions for *Ascaris* reads.** Data generated using *mapDamage* showing C to T and G to A deamination patterns (top) and fragment length profile (bottom) for reads assigned to *Ascaris* in samples with highest read count.>

Discussion

A Multimethod Approach in Paleoparasitology

Each individual method used in our study contributed complementary results to the reconstruction of parasite diversity in the past. For samples from the Neolithic and Bronze Age (where the number of surviving parasite eggs was generally low), microscopy was more sensitive for detecting ancient parasites than was aDNA. As can be seen in Figure 2, six different taxa were recovered using microscopy and this included rare parasites in archeological settings such as *Schistosoma* spp., *Echinostoma* spp., and *Dioctophyma renale*. No parasite DNA sequences were recovered in these samples using our parasite sedaDNA methods, despite all of the taxa being present in our bait set (see S2 Appendix for full list of baits); with 2,262 baits targeting *Schistosoma*, 3,101 baits for *Echinostoma*, and 284 baits for *Dioctophyma renale*. The majority of DNA recovered from these was bacterial in origin. The parasite diversity in these samples was more diverse than that seen in the Roman and medieval periods, and is further characterized by a notable absence of *Ascaris* which is the most common parasite found in later time periods. This raises the possibility that our targeted capture approach was not as efficient at detecting these parasite species as it was for *Ascaris*. Aside from parasite DNA recovery, our sedaDNA methods provided additional data for the identification of the source of coprolites studied. High read counts assigned to dog (*Canis lupus familiaris*) in the Durrington Walls and Must Farm coprolites suggests that these coprolites may in fact be from dogs not humans as previously thought. The accurate identification of coprolites is important for interpretation of parasite results.

Roundworm (*Ascaris* spp.) and whipworm (*Trichuris* spp.) were the most commonly identified parasites by both microscopy and aDNA in the Roman and medieval period samples. In all Roman period samples in which parasite DNA was recovered, eggs from the assigned taxa were identified by microscopy. No additional parasites were detected using ancient DNA analysis for our Roman period sites, and aside from reads assigned to the lancet liver fluke (*Dicrocoelium dendriticum*) in the latrine from Arlon, Belgium, all other reads were from *Ascaris* and could not be assigned to a lower rank than the genus-level. This was not the case for the medieval period samples. In medieval period samples additional taxa were recovered that were not identified using microscopy. The only time period where whipworm (*Trichuris* spp.) DNA

was recovered was from our medieval period samples, despite *Trichuris* sp. being identified by microscopy in Roman period samples.

Combining aDNA with microscopy in paleoparasitology provides the potential to identify ancient parasites to the species-level [14,19,21]. For many parasite taxa, egg morphology cannot be used to accurately identify eggs to the species-level [19,61,62]. We were able to do this in one case using our targeted enrichment approach. Our bait design used a hierarchical approach where baits specific to the species-level were included when possible as well as baits for higher taxonomic levels to increase our ability to capture preserved sequences even outside species-level resolution. This design was used to increase our ability to capture parasite DNA while also providing the opportunity to make species-level identification if appropriate sequences were present. Two species of *Trichuris* were identified in the latrine sediment from 15th–16th c. CE Jerusalem. Both the human-infecting species, *Trichuris trichiura*, and the mouse-infecting species, *Trichuris muris*, were identified. The presence of *T. muris* in the latrine could be a result of mice having access to the latrine or indicate that material contaminated with feces from rodents was deposited in the latrine. In latrine sediment from the medieval site of Mértola, *Ascaris* reads were assigned to the species-level as *Ascaris lumbricoides* by MEGAN, however on further interrogation of these reads we find that they are not unique to *Ascaris lumbricoides* but also match equally well to *Ascaris suum* (classically considered to infect pigs). Given extremely little genetic difference between these two species, it is not surprising that for all other samples, reads could only be assigned to the genus-level [63,64]. Attempts at molecular characterization of *A. suum* and *A. lumbricoides* have revealed very low divergence rates in ITS1 and mitochondrial regions (~0.01%) [65,66]. Comparison of full mitochondrial genomes of *A. lumbricoides* and *A. suum* from China showed 98.1% sequence identity [63]. In addition, species-designation has been questioned on the basis of experimental studies showing that the life-cycle of both species can be completed in either host [67–69]. Thus, the studies above and other authors have questioned the species distinction between *A. lumbricoides* and *A. suum* [70].

One important contribution of our study is that aDNA analysis was able to replicate identification of *Ascaris* spp. samples that contained eggs which had lost their mamillated coat as

a result of degradation and environmental conditions after egg deposition. The outer mamillated coat of *Ascaris* eggs is a key morphological feature used in identification of the eggs. The *Ascaris* eggs identified by microscopy in samples from this study were all decorticated except for those from Arlon, Belgium (Fig 5). Thus, if one is only identifying eggs with preserved mamillated coats, they will grossly underestimate the prevalence of *Ascaris* in the past. We cannot be certain from which source our *Ascaris* DNA stems. As it did not come from the eggs directly, it is possible that *Ascaris* DNA was present in the sediment outside of these decorticated eggs. However, given the correlation of egg concentrations with DNA content in the samples (see Fig 3), it is most likely that this DNA originated from the eggs themselves. Importantly, the overall morphological consistency of these eggs with decorticated *Ascaris* eggs would argue in favor of the origins of these DNA molecules. Even in modern studies, decorticated roundworm eggs can be easily misidentified [59]. Thus, the addition of aDNA analysis can confirm identification in cases of uncertainty due to taphonomic changes and poor egg preservation and allows us to more confidently explore the morphologic appearance of ancient eggs.

<Fig 5. Variation in morphological appearance of *Ascaris* eggs identified in samples with confirmed presence of *Ascaris* using aDNA methods. (A) fertilized egg from Arlon with mamillated coat; (B) fertilized egg from Arlon with mamillated coat; (C) fertilized decorticated egg from Mértola (image credit: Delaney Knorr); (D) fertilized decorticated egg from Mértola (image credit: Delaney Knorr); (E) fertilized decorticated egg from Vacone; (F) fertilized decorticated egg from Vacone; (G and H) fertilized decorticated egg from Sardis with surface cracking; (I) fertilized decorticated egg from Sardis. Scale bars indicate 20 µm.>

The targeted enrichment approach taken allows for the potential recovery of any of the 121 taxa included in the bait set (S2 Appendix), which is the largest targeted approach in paleoparasitology to date without relying on shotgun sequencing. Many paleogenetic studies in parasitology still rely on PCR which has a number of limitations. First, PCR amplicons often

range between 50-600 bp, with most over 100 bp [71–74]. However, most aDNA fragments are smaller than 100 bp, with fragment length distribution modes generally around 40–50 bp [75,76]. Secondly, with targeted enrichment one can include baits designed to capture large genomic regions from mitochondrial and nuclear loci in one reaction. Most studies in paleoparasitology relying on PCR methods targeted a maximum of 5 taxa or even a single taxon previously identified by microscopy [13,26,29,30,48,74]. Targeted enrichment has only previously been used in parasite aDNA research for detecting malaria (*Plasmodium* sp.) in skeletal remains [77]. On the other hand, while we are limited by coverage of the bait set and references sequences available for robust bait design, targeted enrichment allowed for recovery of parasite DNA when shotgun sequencing to an average depth of 3.3 million reads (total read counts from shotgun sequencing ranged from 2.2–4.0 million reads) was unsuccessful in recovering any parasite DNA. In published paleoparasitological studies relying on shotgun sequencing, most samples were sequenced to a depth of at least 5 million reads with some samples being sequenced to a depth of 60 million reads [12,14,21]. The cost of sequencing to this level is not financially feasible for many studies and most aDNA and paleoparasitology labs. Additionally, in most cases less than 5% of these sequenced reads are assigned to target organisms [78]. In our samples even with targeted enrichment, <0.001% of reads were assigned to parasites. Due to the very small proportion of parasite DNA in our sediment samples (which is otherwise dominated by bacteria and currently unidentifiable DNA, along with plants and fungi), very deep shotgun sequencing would be needed to find parasite DNA in environmental samples. Paleogenetic studies in parasitology are likely to continue to lag behind that seen for other pathogens without changes that make the use of aDNA more cost effective. Microscopy is a relatively cheap and reliable method for egg identification from archeological sediments making it a preferred method for many researchers.

While ancient DNA analysis is a promising addition to the methodological armamentarium in paleoparasitology, our results suggest that currently microscopy remains the most cost-effective and reliable screening tool in paleoparasitology. When resources allow, ELISA and aDNA can be utilized alongside microscopy to analyze a sample more comprehensively. In paleoecological studies as well, sedaDNA has been found to function best

as a complementary proxy, as other methods such as palynology and macrofossil identification tend to find similar, but non-overlapping taxa [79–81]. Microscopy continues to successfully recover parasites eggs in samples with low egg concentration. Based on our results comparing eggs per gram concentration generated from microscopic analysis to percent of LCA-assigned reads, there appears to be a minimum concentration of eggs needed for increased chance of DNA recovery using the currently described methods. Samples that contained 30 eggs per gram or lower of *Ascaris* eggs did not contain any detectable *Ascaris* DNA. It is difficult to directly compare this to other studies as many do not report eggs per gram or had higher egg concentrations in all samples studied [13]. However, both in our study and other paleoparasitological studies, there are cases where parasite DNA is detected in the absence of any preserved helminth eggs [14,26,48]. In these cases it is possible that taphonomic changes to the eggs have resulted in inability to identify them by microscopy but that their DNA can still be captured and sequenced. Alternatively, that DNA from adult worms themselves rather than eggs is being detected. It is also likely that eggs are not evenly distributed within a sample and studying different subsamples with microscopy and aDNA will provide different findings.

The complexities of parasite egg preservation and in turn parasite DNA preservation is poorly understood. This diverse group of organisms has variable eggshell structures, rates of embryological development, environmental niches, and are recovered from different contexts including in the gastrointestinal space as in the case of pelvic soil as well as cesspit, latrine, and drain contexts. We were able to detect parasite DNA in various sample types including pelvic soil, coprolites, and latrine or cesspit samples. We had the most success with identifying *Ascaris* using our sedaDNA methods and targeted enrichment. The ability to more easily recover *Ascaris* DNA from all samples may be related to both methodological factors as well as parasite biology. As part of its life cycle, *Ascaris* develops in soil, thus by necessity it is resistant to harsh environmental conditions. In addition, extensive reference sequences for *Ascaris* allowed us to create a robust set of baits for *Ascaris*. A total of 1,769 of 30,240 baits with average tiling density of 7x were for either *Ascaris lumbricoides* or *Ascaris suum* (see S2 Appendix for list of baits). In comparison, our bait set had very good coverage of taxa identified by microscopy for which we were not able to recover DNA. *Echinostoma* spp. had a total of 3,213 baits (average

tiling density ranged for each species from 8.4–21.8x), *Schistosoma* spp. had 2,262 baits (average tiling density ranged for each species from 8.1–21.8x), and *Dibothriocephalus* spp. (syn. *Diphyllobothrium* spp.) had 1,980 baits (average tiling density ranged for each species from 8.8–23.4x).

Parasite biology will also influence DNA recovery [26]. The size of adult worms from different helminths is variable. For example, *Ascaris lumbricoides* can grow up to 30 cm long whereas *Trichuris trichiura* is typically only 5 cm long and *Dibothriocephalus latus* can grow to 1000 cm [61]. Thus, the biomass of the adult worms would be expected to vary by taxa. This would impact the amount of DNA expected from each species in pelvic soil samples, as pelvic soil samples would contain decomposed intestinal contents including helminths causing infection at the time of death. The number of eggs excreted per day is also quite variable, with *Ascaris lumbricoides* producing around 200,000 eggs per day [82] and *Trichuris trichiura* only 18,000 eggs per day [83]. These factors may be compounded by sedaDNA preservation characteristics, which are thought to necessitate binding to sedimentary minerals [84–87]. If parasite DNA does not come in contact with sedimentary minerals before being broken down by microbes in the local microbiome, those parasite DNA fragments are unlikely to preserve and be recoverable.

While sedaDNA with targeted enrichment has the potential to significantly aid in paleoparasitological research, further methodological improvements are needed to reach the taxonomic characterization already viable with the gold standard method within the field—microscopy. Furthermore, *Giardia duodenalis* antigen was detected using ELISA from four sites, and although our bait set did include protozoa, we were not able to recover any DNA sequences from *Giardia duodenalis*. Thus, assuming that the ELISA signal is real, it does continue to have a unique value in that it is a useful additional method for maximizing recovery of common intestinal protozoa, which are rarely preserved well enough to be visualized using microscopy and have only rarely been detected in aDNA studies thus far [25]. However, the indeterminate results from Must Farm highlight an important limitation of ELISA which is the potential for cross-reactivity with unknown antigens that may be found in soil. Commercial ELISA kits used in this and other studies have very high sensitivity and specificity (>97%) and in

their validation there was no cross-reactivity reported for other parasite species tested. However, further exploration of how these performance characteristics change when applied to archaeological samples is needed. Further, optimization of aDNA methods for detection of protozoa would be valuable to confirm ELISA results with a second method.

Temporal Changes in Parasites in the Roman Period

Using a multimethod approach, we aimed to produce a more accurate reconstruction of parasite infection in the Roman period. Inclusion of pre-Roman and post-Roman samples from areas that were at one time part of the Roman Empire provides important comparative data to understand how parasite diversity may have changed during the Roman period. The samples included in our study contribute a small subset of results for parasite presence in areas that were once part of the Roman Empire from the Neolithic through the medieval period. The parasite taxa recovered from the pre-Roman sites were much more diverse than any other time period. At these sites we recovered various zoonotic parasites including *Echinostoma* sp. and fish tapeworm (*Dibothriocephalus* sp.) which are spread by ingestion of raw or undercooked fish, frogs, or mollusks, as well as *Capillaria* sp. from two sites. This is in conjunction with one common soil-transmitted helminth, whipworm (*Trichuris trichiura*), which is spread by the fecal-oral route.

By comparison in the Roman period, we observe almost exclusively soil-transmitted helminths, roundworm (*Ascaris* sp.) and whipworm (*Trichuris trichiura*). One exception is the site of Arlon, Belgium where *Capillaria* sp. and the lancet liver fluke (*Dicrocoelium dendriticum*) were also recovered. These two taxa of liver flukes are zoonotic parasites that likely represent false parasitism as a result of ingestion of liver from infected animals [88–91]. Though true infections can occur in humans they are rare. Similarly, in medieval period sites, soil-transmitted helminths are almost exclusively present, aside from a coprolite from Jerusalem where fish tapeworm (*Dibothriocephalus* sp.) was also found.

When these results are included within the published literature on parasite presence in these time periods, our results are comparable to patterns seen using a much larger dataset (see Tables F.1–F.3 in S1 Appendix for a review of published studies). The proportion of sites

with whipworm is consistently high in all time periods, while roundworm appears to increase in frequency in the Roman period and remains stable at that level in the medieval period (Fig 6). Roundworm was recovered from 35% of pre-Roman sites, which increased to 67% of Roman and 79% of medieval period sites. The increase in proportion of sites where roundworm was found in the Roman and medieval periods, and whipworm in the medieval period may reflect decreased sanitation and hygiene. Numerous other factors such as increased urbanization, cultural preferences for the use of chamber pots, and agricultural practices involving the use of human feces as fertilizer may have all contributed to this increase in fecal-oral parasite presence [33,92].

<Fig 6. Temporal trends in parasite diversity before, during, and after the Roman period for the most common helminths recovered from archeological samples. For each parasite, bars indicate the proportion of studied sites where each parasite was recovered for pre-Roman, Roman, and post-Roman sites with 95% confidence intervals. See S1 Appendix for individual studies used to generate temporal trends.>

Zoonotic parasites (parasites spread from animals to humans directly or through ingestion of meat) [93], including fish tapeworm (*Dibothriocephalus* sp.), liver flukes (*Dicrocoelium dendriticum* and *Fasciola hepatica*) and beef/pork tapeworm (*Taenia* sp.) are generally more common in pre-Roman time periods compared to the later Roman and medieval periods (Fig 6). This may reflect changes to human-animal interactions and dietary preferences. It is possible that the decline in lancet liver fluke during the Roman period is an anomaly in our data as the proportion of sites where it was found in the medieval period is quite consistent with the pre-Roman period. However, it does appear that *Fasciola* liver fluke was more common in the pre-Roman period compared to later time periods. It was found at 40% of pre-Roman sites compared to 12% of Roman and 12% of medieval sites. Both types of liver flukes in fecal samples can reflect true infection or false parasitism, usually from

consumption of animal liver containing eggs [94]. In all time periods, nearly all sites where *Fasciola hepatica* was found are North of the Alps.

A similar pattern as seen for liver flukes is seen for fish tapeworm (*Dibothriocephalus* sp.). Fish tapeworm was most common in pre-Roman sites, being found at 40% of sites and then decreases to 10% in the Roman period and 19% in the medieval period. Fish tapeworm is acquired from ingestion of raw or undercooked freshwater fish in Europe. The decline in the Roman period may represent a preference for cooked fish or, alternatively, a decreased reliance on freshwater fish but rather marine fish.

There are many intricacies of transmission and determinants of infection that can be explored for each individual parasite. However, what is most striking overall is the high proportion of sites in all time periods in Europe where we find evidence for enteric parasites. Roundworm is present at 67% of Roman sites studied and whipworm is found at 61% of sites. Of course, a major limitation of this type of analysis is publication bias. However, there is no doubt that these two parasites contributed to the burden of disease in most Roman communities. Many infections with these two species can be asymptomatic; however, they can also cause gastrointestinal upset and for children result in nutrient deficiencies, stunting of growth, and intellectual disability [95].

Conclusions

The vast majority of work done in paleoparasitology or archeoparasitology has relied on microscopic analysis of sediments and paleofeces to identify parasites. ELISA has also been used by some groups to detect ancient protozoa. We have shown that sedaDNA methods combined with targeted enrichment can be used to detect parasite DNA in as little as 0.25 g of paleofecal sediment. A multimethod approach in paleoparasitology allows for the most complete reconstruction of past parasite diversity currently available to us. However, microscopy continues to be the most sensitive method for detecting helminths in paleofecal samples and appears to detect helminth eggs present in lower concentrations than our sedaDNA methods are able to detect. ELISA is uniquely able to detect protozoa such as *Giardia duodenalis* in our samples. In our set of samples, aDNA replicated identification of *Ascaris* presence in samples

with decorticated eggs that can be challenging to identify, provided species-level identification in one case, and detected additional parasites not seen by microscopy and ELISA. However, further work optimizing sedaDNA methods for the field of paleoparasitology are needed. As these methods are refined and applied to additional samples, they may help facilitate a more holistic analysis of parasite phylogeography and parasite biology through time.

Applying this multimethod approach to 26 coprolite, latrine, and sewer samples from the Neolithic through medieval period allowed us to characterize temporal trends in parasite infection around the rise, expansion and eventual decline of the Roman Empire. We found a trend of decreased zoonotic parasites from the pre-Roman to Roman and medieval periods and concurrent increase in parasites spread by the fecal-oral route, especially roundworm.

Acknowledgments

We would like to thank local archeological institutions, and excavation teams from the sites included in this study, and the official parties granting the required permissions. We thank Techlab, Blacksburg, USA for their kind donation of ELISA kits used in this research. We thank Christa Clamer for providing material and contextual interpretation of our results. Thanks to Brian Golding for providing access to his computational resources, and to Ana Duggan for assistance and guidance on bioinformatic workflow. Thanks to all members and affiliates of the McMaster Ancient DNA Centre for their ongoing support of this work. Numerous individuals have contributed to various stages of this work; from the site of Sagalassos we would like to thank the Research Foundation Flanders and the Research Fund of KU Leuven for supporting the research for this paper. From the site of Ephesus we would like to thank the late excavation director Sabine Ladstätter, who made it possible for us to collect samples for analysis. From Vacone we would like to thank Devin Ward, Gary Farney, Dylan Bloy, and the Soprintendenza Archeologia, Belle arti e Paesaggio per l'area metropolitana di Roma e la provincia di Rieti.

Funding Sources

This work was supported by the Social Sciences and Humanities Research Council of Canada (752-2016-2085 to MLL), a Tidmarsh Cambridge Scholarship from the Cambridge

Commonwealth, European and International Trust and Trinity Hall College, and the Society for the Promotion of Roman Studies (to MLL), a CANA Foundation grant (to TJM and HNP), the Garfield Weston Foundation (to TJM), the Social Sciences and Humanities Research Council of Canada (767-2016-2288 to TJM), the Canadian Institute for Advanced Research, and the Natural Sciences and Engineering Research Council of Canada (to HP). The funders had no role in study design, data collection and analysis, decision to publish, or preparation of the manuscript.

References

1. Bos KI, Kühnert D, Herbig A, Esquivel-Gomez LR, Valtueña AA, Barquera R, et al. Paleomicrobiology: diagnosis and evolution of ancient pathogens. Annu Rev Microbiol. 2019. doi:10.1146/annurev-micro-090817-062436

2. Arriola LA, Cooper A, Weyrich LS. Palaeomicrobiology: application of ancient DNA sequencing to better understand bacterial genome evolution and adaptation. Frontiers in Ecology and Evolution. 2020;8. doi:10.3389/fevo.2020.00040

3. Duchêne S, Ho SYW, Carmichael AG, Holmes EC, Poinar H. The recovery, interpretation and use of ancient pathogen genomes. Curr Biol. 2020;30: R1215–R1231. doi:10.1016/j.cub.2020.08.081

4. Lewis CM Jr, Akinyi MY, DeWitte SN, Stone AC. Ancient pathogens provide a window into health and well-being. Proc Natl Acad Sci U S A. 2023;120: e2209476119. doi:10.1073/pnas.2209476119

5. Wagner DM, Klunk J, Harbeck M, Devault A, Waglechner N, Sahl JW, et al. *Yersinia pestis* and the Plague of Justinian 541–543 AD: a genomic analysis. Lancet Infect Dis. 2014;14: 319–326. doi:10.1016/S1473-3099(13)70323-2

6. Key FM, Posth C, Esquivel-Gomez LR, Hübler R, Spyrou MA, Neumann GU, et al. Emergence of human-adapted *Salmonella enterica* is linked to the Neolithization process. Nat Ecol Evol. 2020;4: 324–333. doi:10.1038/s41559-020-1106-9

7. Mühlemann B, Jones TC, Damgaard P de B, Allentoft ME, Shevnina I, Logvin A, et al. Ancient hepatitis B viruses from the Bronze Age to the Medieval period. Nature. 2018;557: 418–423. doi:10.1038/s41586-018-0097-z

8. Rascovan N, Sjögren K-G, Kristiansen K, Nielsen R, Willerslev E, Desnues C, et al. Emergence and spread of basal lineages of *Yersinia pestis* during the Neolithic decline. Cell. 2019;176: 295-305.e10. doi:10.1016/j.cell.2018.11.005

9. Spyrou MA, Bos KI, Herbig A, Krause J. Ancient pathogen genomics as an emerging tool for infectious disease research. Nat Rev Genet. 2019;20: 323–340. doi:10.1038/s41576-019-0119-1

10. Eaton K, Featherstone L, Duchene S, Carmichael AG, Varlık N, Golding GB, et al. Plagued by a cryptic clock: insight and issues from the global phylogeny of *Yersinia pestis*. Commun Biol. 2023;6: 23. doi:10.1038/s42003-022-04394-6

11. Hagan RW, Hofman CA, Hübner A, Reinhard K, Schnorr S, Lewis CM Jr, et al. Comparison of extraction methods for recovering ancient microbial DNA from paleofeces. Am J Phys Anthropol. 2020;171: 275–284. doi:10.1002/ajpa.23978

12. Sabin S, Yeh H-Y, Pluskowski A, Clamer C, Mitchell PD, Bos KI. Estimating molecular preservation of the intestinal microbiome via metagenomic analyses of latrine sediments from two medieval cities. Philos Trans R Soc Lond B Biol Sci. 2020;375: 20190576. doi:10.1098/rstb.2019.0576

13. Flammer PG, Dellicour S, Preston SG, Rieger D, Warren S, Tan CKW, et al. Molecular archaeoparasitology identifies cultural changes in the Medieval Hanseatic trading centre of Lübeck. Proc R Soc B. 2018;285: 20180991. doi:10.1098/rspb.2018.0991

14. Søe MJ, Nejsum P, Seersholm FV, Fredensborg BL, Habraken R, Haase K, et al. Ancient DNA from latrines in Northern Europe and the Middle East (500 BC-1700 AD) reveals past parasites and diet. PLOS One. 2018;13: e0195481. doi:10.1371/journal.pone.01954

15. Flammer PG, Ryan H, Preston SG, Warren S, Přichystalová R, Weiss R, et al. Epidemiological insights from a large-scale investigation of intestinal helminths in Medieval Europe. PLoS Negl Trop Dis. 2020;14: e0008600. doi:10.1371/journal.pntd.0008600

16. Appelt S, Fancello L, Le Bailly M, Raoult D, Drancourt M, Desnues C. Viruses in a 14th-century coprolite. Appl Environ Microbiol. 2014;80: 2648–2655. doi:10.1128/AEM.03242-13

17. Ng TFF, Chen L-F, Zhou Y, Shapiro B, Stiller M, Heintzman PD, et al. Preservation of viral genomes in 700-y-old caribou feces from a subarctic ice patch. Proc Natl Acad Sci U S A. 2014;111: 16842–16847. doi:10.1073/pnas.1410429111

18. Ferreira LF. An introduction to paleoparasitology. In: Reinhard KJ, Araújo A, editors. Foundations of Paleoparasitology. Rio de Janeiro: Editoria Fiocruz; 2014. pp. 27–42.

19. Côté NM-L, Le Bailly M. Palaeoparasitology and palaeogenetics: review and perspectives for the study of ancient human parasites. Parasitology. 2018;145: 656–664. doi:10.1017/S003118201700141X

20. Selbach C, Jorge F, Dowle E, Bennett J, Chai X, Doherty J-F, et al. Parasitological research in the molecular age. Parasitology. 2019;146: 1361–1370. doi:10.1017/S0031182019000726

21. Tams KW, Jensen Søe M, Merkyte I, Valeur Seersholm F, Henriksen PS, Klingenberg S, et al. Parasitic infections and resource economy of Danish Iron Age settlement through ancient DNA sequencing. PLoS One. 2018;13: e0197399. doi:10.1371/journal.pone.0197399

22. Chessa D, Murgia M, Sias E, Deligios M, Mazzarello V, Fiamma M, et al. Metagenomics and microscope revealed *T. trichiura* and other intestinal parasites in a cesspit of an Italian nineteenth century aristocratic palace. Sci Rep. 2020;10: 12656. doi:10.1038/s41598-020-69497-8

23. Guedes L, Borba VH, Camacho M, Neto J, Dias O, Iñiguez AM. African helminth infection out of Africa: paleoparasitological and paleogenetic investigations in Pretos Novos

cemetery, Rio de Janeiro, Brazil (1769-1830). Acta Trop. 2020;205: 105399. doi:10.1016/j.actatropica.2020.105399

24. Beltrame MO, Pruzzo C, Sanabria R, Pérez A, Mora MS. First report of pre-Hispanic *Fasciola hepatica* from South America revealed by ancient DNA. Parasitology. 2020;147: 371–375. doi:10.1017/S0031182019001719

25. Wiscovitch-Russo R, Rivera-Perez J, Narganes-Storde YM, García-Roldán E, Bunkley-Williams L, Cano R, et al. Pre-Columbian zoonotic enteric parasites: an insight into Puerto Rican indigenous culture diets and life styles. PLoS One. 2020;15: e0227810. doi:10.1371/journal.pone.0227810

26. Roche K, Capelli N, Pacciani E, Lelli P, Pallecchi P, Bianucci R, et al. Gastrointestinal parasite burden in 4th-5th c. CE Florence highlighted by microscopy and paleogenetics. Infect Genet Evol. 2021;90: 104713. doi:10.1016/j.meegid.2021.104713

27. Askari Z, Ruehli F, Bouwman A, Shariati V, Naddaf SR, Otranto D, et al. Genomic palaeoparasitology traced the occurrence of *Taenia asiatica* in ancient Iran (Sassanid Empire, 2th cent. CE-6th cent. CE). Sci Rep. 2022;12: 12045. doi:10.1038/s41598-022-10690-2

28. Doyle SR, Søe MJ, Nejsum P, Betson M, Cooper PJ, Peng L, et al. Population genomics of ancient and modern *Trichuris trichiura*. Nature Communications. 2022;13: 3888. Available: https://www.nature.com/articles/s41467-022-31487-x

29. Iñiguez AM, Brito L, Guedes L, Chaves SA de M. Helminth infection and human mobility in sambaquis: paleoparasitological, paleogenetic, and microremains investigations in Jabuticabeira II, Brazil (2890 ± 55 to 1805 ± 65 BP). Holocene. 2022;32: 200–207. doi:10.1177/09596836211060490

30. Oh CS, Seo M, Lee HJ, Kim MJ, Lim D-S, Shin DH. Genetic analysis of ancient *Clonorchis sinensis* eggs attained from Goryeong Mummy of Joseon Dynasty Period. J Parasitol. 2022;108: 70–78. doi:10.1645/21-49

31. Murchie TJ, Kuch M, Duggan AT, Ledger ML, Roche K, Klunk J, et al. Optimizing extraction and targeted capture of ancient environmental DNA for reconstructing past environments using the PalaeoChip Arctic-1.0 bait-set. Quat Res. 2021;99: 305–328. doi:10.1017/qua.2020.59

32. Anastasiou E, Mitchell PD. Simplifying the process of extracting intestinal parasite eggs from archaeological sediment samples: a comparative study of the efficacy of widely-used disaggregation techniques. International Journal of Paleopathology. 2013;3: 204–207. doi:10.1016/j.ijpp.2013.04.004

33. Ledger ML, Micarelli I, Ward D, Prowse TL, Carroll M, Killgrove K, et al. Gastrointestinal infection in Italy during the Roman Imperial and Longobard periods: a paleoparasitological analysis of sediment from skeletal remains and sewer drains. International Journal of Paleopathology. 2021;33: 61–71. doi:10.1016/j.ijpp.2021.03.001

34. Anastasiou E, Lorentz KO, Stein GJ, Mitchell PD. Prehistoric schistosomiasis parasite found in the Middle East. Lancet Infectious Diseases. 2014;14: 553–554. doi:10.1016/S1473-3099(14)70794-7

35. Yeh H-Y, Prag K, Clamer C, Humbert J-B, Mitchell PD. Human intestinal parasites from a Mamluk Period cesspool in the Christian quarter of Jerusalem: Potential indicators of long distance travel in the 15th century AD. International Journal of Paleopathology. 2015;9: 69–75. doi:10.1016/j.ijpp.2015.02.003

36. Williams FS, Arnold-Foster T, Yeh H-Y, Ledger ML, Baeten J, Poblome J, et al. Intestinal parasites from the 2nd-5th century AD latrine in the Roman Baths at Sagalassos (Turkey). Int J Paleopathol. 2017;19: 37–42. doi:10.1016/j.ijpp.2017.09.002

37. Ledger ML, Stock F, Schwaiger H, Knipping M, Brückner H, Ladstätter S, et al. Intestinal parasites from public and private latrines and the harbour canal in Roman Period Ephesus, Turkey (1st c. BCE to 6th c. CE). Journal of Archaeological Science: Reports. 2018;21: 289–297. doi:10.1016/j.jasrep.2018.07.013

38. Ledger ML, Anastasiou E, Shillito L-M, Mackay H, Bull ID, Haddow SD, et al. Parasite infection at the early farming community of Çatalhöyük. Antiquity. 2019;93: 573–587. doi:10.15184/aqy.2019.61

39. Ledger ML, Grimshaw E, Fairey M, Whelton HL, Bull ID, Ballantyne R, et al. Intestinal parasites at the Late Bronze Age settlement of Must Farm, in the fens of East Anglia, UK (9th century B.C.E.). Parasitology. 2019;146: 1583–1594. doi:10.1017/S0031182019001021

40. Knorr DA, Smith WPW, Ledger ML, Peña-Chocarro L, Pérez-Jordà G, Clapés R, et al. Intestinal parasites in six Islamic medieval period latrines from 10th–11th century Córdoba (Spain) and 12th–13th century Mértola (Portugal). International Journal of Paleopathology. 2019;26: 75–83. doi:10.1016/j.ijpp.2019.06.004

41. Ledger ML, Rowan E, Marques FG, Sigmier JH, Šarkić N, Redžić S, et al. Intestinal parasitic infection in the Eastern Roman Empire during the Imperial Period and Late Antiquity. Am J Archaeol. 2020;124: 631–657. doi:10.3764/aja.124.4.0631

42. Deforce K, Ledger ML, Derreumaux M, Goffette Q, Henrotay D, Pigière F, et al. Diet, hygiene and health in Roman period northern Gaul: a multidisciplinary study of a latrine from an artisan household in the vicus Orolaunum (Arlon, southern Belgium, c. 250–280 CE). Journal of Archaeological Science: Reports. 2021;35: 102761. doi:10.1016/j.jasrep.2020.102761

43. Mitchell PD, Anastasiou E, Whelton HL, Bull ID, Pearson MP, Shillito L-M. Intestinal parasites in the Neolithic population who built Stonehenge (Durrington Walls, 2500 BCE). Parasitology. 2022;149: 1027–1033. doi:10.1017/S0031182022000476

44. Gonçalves MLC, da Silva VL, de Andrade CM, Reinhard K, da Rocha GC, Le Bailly M, et al. Amoebiasis distribution in the past: first steps using an immunoassay technique. Trans R Soc Trop Med Hyg. 2004;98: 88–91. doi:10.1016/S0035-9203(03)00011-7

45. Le Bailly M, Bouchet F. Paléoparasitologie et immunologie. L’exemple d’*Entamoeba histolytica*. ArcheoSciences. 2006;30: 129–135. doi:10.4000/archeosciences.281

46. Mitchell PD, Stern E, Tepper Y. Dysentery in the crusader kingdom of Jerusalem: an ELISA analysis of two medieval latrines in the City of Acre (Israel). J Archaeol Sci. 2008;35: 1849–1853. doi:10.1016/j.jas.2007.11.017

47. Le Bailly M, Bouchet F. A first attempt to retrace the history of dysentery caused by *Entamoeba histolytica*. In: Mitchell PD, editor. Sanitation, latrines and intestinal parasites in past populations. Farnham: Ashgate; 2015. pp. 219–228.

48. Søe MJ, Nejsum P, Fredensborg BL, Kapel CMO. DNA typing of ancient parasite eggs from environmental samples identifies human and animal worm infections in Viking-age settlement. J Parasitol. 2015;101: 57–63. doi:10.1645/14-650.1

49. Kaisar MMM, Brienen EAT, Djuardi Y, Sartono E, Yazdanbakhsh M, Verweij JJ, et al. Improved diagnosis of *Trichuris trichiura* by using a bead-beating procedure on ethanol preserved stool samples prior to DNA isolation and the performance of multiplex real-time PCR for intestinal parasites. Parasitology. 2017;144: 965–974. doi:10.1017/S0031182017000129

50. Dabney J, Knapp M, Glocke I, Gansauge M-T, Weihmann A, Nickel B, et al. Complete mitochondrial genome sequence of a Middle Pleistocene cave bear reconstructed from ultrashort DNA fragments. Proc Natl Acad Sci U S A. 2013;110: 15758–15763. doi:10.1073/pnas.1314445110

51. Meyer M, Kircher M. Illumina sequencing library preparation for highly multiplexed target capture and sequencing. Cold Spring Harb Protoc. 2010;2010: db.prot5448. doi:10.1101/pdb.prot5448

52. Kircher M, Sawyer S, Meyer M. Double indexing overcomes inaccuracies in multiplex sequencing on the Illumina platform. Nucleic Acids Res. 2012;40: e3. doi:10.1093/nar/gkr771

53. Benson DA, Cavanaugh M, Clark K, Karsch-Mizrachi I, Lipman DJ, Ostell J, et al. GenBank. Nucleic Acids Res. 2013;41: D36-42. doi:10.1093/nar/gks1195

54. NCBI Resource Coordinators. Database resources of the National Center for Biotechnology Information. Nucleic Acids Res. 2016;44: D7-19. doi:10.1093/nar/gkv1290

55. Renaud G, Stenzel U, Kelso J. leeHom: adaptor trimming and merging for Illumina sequencing reads. Nucleic Acids Res. 2014;42: e141. doi:10.1093/nar/gku699

56. Roche K, Pacciani E, Bianucci R, Le Bailly M. Assessing the parasitic burden in a Late Antique Florentine emergency burial site. Korean J Parasitol. 2019;57: 587–593. doi:10.3347/kjp.2019.57.6.587

57. Mitchell PD, Yeh H-Y, Appleby J, Buckley R. The intestinal parasites of King Richard III. Lancet. 2013;382: 888. doi:10.1016/S0140-6736(13)61757-2

58. Oh K-S, Kim G-T, Ahn K-S, Shin S-S. Effects of disinfectants on larval development of *Ascaris suum* eggs. Korean J Parasitol. 2016;54: 103–107. doi:10.3347/kjp.2016.54.1.103

59. Maurelli MP, Alves LC, Aggarwal CS, Cociancic P, Levecke B, Cools P, et al. *Ascaris lumbricoides* eggs or artefacts? A diagnostic conundrum. Parasitology. 2021;148: 1554–1559. doi:10.1017/S0031182021001256

60. Jónsson H, Ginolhac A, Schubert M, Johnson PLF, Orlando L. mapDamage2.0: fast approximate Bayesian estimates of ancient DNA damage parameters. Bioinformatics. 2013;29: 1682–1684. doi:10.1093/bioinformatics/btt193

61. Garcia LS. Diagnostic Medical Parasitology. Washington, DC: ASM Press; 2016.

62. Maicher C, Hoffmann A, Côté NML, Palomo Pérez A, Saña Segui M, Le Bailly M. Paleoparasitological investigations on the Neolithic lakeside settlement of La Draga (Lake Banyoles, Spain). Holocene. 2017;27: 1659–1668. doi:10.1177/0959683617702236

63. Liu G-H, Wu C-Y, Song H-Q, Wei S-J, Xu M-J, Lin R-Q, et al. Comparative analyses of the complete mitochondrial genomes of *Ascaris lumbricoides* and *Ascaris suum* from humans and pigs. Gene. 2012;492: 110–116. doi:10.1016/j.gene.2011.10.043

64. Søe MJ, Kapel CMO, Nejsum P. *Ascaris* from humans and pigs appear to be reproductively isolated species. PLoS Negl Trop Dis. 2016;10: e0004855. doi:10.1371/journal.pntd.0004855

65. Leles D, Araújo A, Vicente ACP, Iñiguez AM. ITS1 intra-individual variability of *Ascaris* isolates from Brazil. Parasitol Int. 2010;59: 93–96. doi:10.1016/j.parint.2009.10.001

66. Iñiguez AM, Leles D, Jaeger LH, Carvalho-Costa FA, Araújo A, Amazonas Research Group. Genetic characterisation and molecular epidemiology of *Ascaris* spp. from humans and pigs in Brazil. Trans R Soc Trop Med Hyg. 2012;106: 604–612. doi:10.1016/j.trstmh.2012.06.009

67. Criscione CD, Anderson JD, Sudimack D, Peng W, Jha B, Williams-Blangero S, et al. Disentangling hybridization and host colonization in parasitic roundworms of humans and pigs. Proc Biol Sci. 2007;274: 2669–2677. doi:10.1098/rspb.2007.0877

68. Nejsum P, Betson M, Bendall RP, Thamsborg SM, Stothard JR. Assessing the zoonotic potential of *Ascaris suum* and *Trichuris suis*: looking to the future from an analysis of the past. J Helminthol. 2012;86: 148–155. doi:10.1017/S0022149X12000193

69. Betson M, Nejsum P, Bendall RP, Deb RM, Stothard JR. Molecular epidemiology of ascariasis: a global perspective on the transmission dynamics of *Ascaris* in people and pigs. J Infect Dis. 2014;210: 932–941. doi:10.1093/infdis/jiu193

70. Alves EB da S, Conceição MJ, Leles D. *Ascaris lumbricoides* , *Ascaris suum*, or “*Ascaris lumbrisuum*”? J Infect Dis. 2016;213: 1355–1355. doi:10.1093/infdis/jiw027

71. Oh CS, Seo M, Chai JY, Lee SJ, Kim MJ, Park JB, et al. Amplification and sequencing of *Trichuris trichiura* ancient DNA extracted from archaeological sediments. J Archaeol Sci. {2010;37: 1269–1273. doi:10.1016/j.jas.2009.12.029

72. Jaeger LH, Iñiguez AM. Molecular paleoparasitological hybridization approach as effective tool for diagnosing human intestinal parasites from scarce archaeological remains. PLoS One. 2014;9: e105910. doi:10.1371/journal.pone.0105910

73. Côté NML, Daligault J, Pruvost M, Bennett EA, Gorgé O, Guimaraes S, et al. A new high-throughput approach to genotype ancient human gastrointestinal parasites. PLoS One. 2016;11: e0146230. doi:10.1371/journal.pone.0146230

74. Hong JH, Oh CS, Seo M, Chai J-Y, Shin DH. Ancient *Ascaris* DNA sequences of Cytochrome B, Cytochrome C Oxidase Subunit 1, NADH Dehydrogenase Subunit 1, and Internal Transcribed Spacer 1 genes from Korean Joseon Mummy feces. J Parasitol. 2017;103: 795–800. doi:10.1645/16-102

75. Sawyer S, Krause J, Guschanski K, Savolainen V, Pääbo S. Temporal patterns of nucleotide misincorporations and DNA fragmentation in ancient DNA. PLoS One. 2012;7: e34131. doi:10.1371/journal.pone.0034131

76. Kistler L, Ware R, Smith O, Collins M, Allaby RG. A new model for ancient DNA decay based on paleogenomic meta-analysis. Nucleic Acids Res. 2017;45: 6310–6320. doi:10.1093/nar/gkx361

77. Marciniak S, Prowse TL, Herring DA, Klunk J, Kuch M, Duggan AT, et al. *Plasmodium falciparum* malaria in 1st–2nd century CE southern Italy. Curr Biol. 2016;26: R1220–R1222. doi:10.1016/j.cub.2016.10.016

78. Marciniak S, Klunk J, Devault A, Enk J, Poinar HN. Ancient human genomics: the methodology behind reconstructing evolutionary pathways. J Hum Evol. 2015;79: 21–34. doi:10.1016/j.jhevol.2014.11.003

79. Pedersen MW, Ginolhac A, Orlando L, Olsen J, Andersen K, Holm J, et al. A comparative study of ancient environmental DNA to pollen and macrofossils from lake sediments reveals taxonomic overlap and additional plant taxa. Quat Sci Rev. 2013;75: 161–168. doi:10.1016/j.quascirev.2013.06.006

80. Rawlence NJ, Lowe DJ, Wood JR, Young JM, Churchman GJ, Huang Y-T, et al. Using palaeoenvironmental DNA to reconstruct past environments: Progress and prospects. J Quat Sci. 2014;29: 610–626. doi:10.1002/jqs.2740

81. Parducci L, Väliranta M, Salonen JS, Ronkainen T, Matetovici I, Fontana SL, et al. Proxy comparison in ancient peat sediments: pollen, macrofossil and plant DNA. Philos Trans R Soc Lond B Biol Sci. 2015;370: 20130382. doi:10.1098/rstb.2013.0382

82. Phuphisut O, Poodeepiyasawat A, Yoonuan T, Watthanakulpanich D, Chotsiri P, Reamtong O, et al. Transcriptome profiling of male and female *Ascaris lumbricoides* reproductive tissues. Parasit Vectors. 2022;15: 477. doi:10.1186/s13071-022-05602-2

83. Hansen EP, Tejedor AM, Thamsborg SM, Alstrup Hansen TV, Dahlerup JF, Nejsum P. Faecal egg counts and expulsion dynamics of the whipworm, *Trichuris trichiura* following self-infection. J Helminthol. 2016;90: 298–302. doi:10.1017/S0022149X1500019X

84. Cai P, Huang Q, Zhang X, Chen H. Adsorption of DNA on clay minerals and various colloidal particles from an Alfisol. Soil Biol Biochem. 2006;38: 471–476. doi:10.1016/j.soilbio.2005.05.019

85. Saeki K, Sakai M, Wada S-I. DNA adsorption on synthetic and natural allophanes. Appl Clay Sci. 2010;50: 493–497. doi:10.1016/j.clay.2010.09.015

86. Kanbar HJ, Olajos F, Englund G, Holmboe M. Geochemical identification of potential DNA-hotspots and DNA-infrared fingerprints in lake sediments. Appl Geochem. 2020;122: 104728. doi:10.1016/j.apgeochem.2020.104728

87. Kjær KH, Winther Pedersen M, De Sanctis B, De Cahsan B, Korneliussen TS, Michelsen CS, et al. A 2-million-year-old ecosystem in Greenland uncovered by environmental DNA. Nature. 2022;612: 283–291. doi:10.1038/s41586-022-05453-y

88. Gonçalves MLC, Araújo A, Ferreira LF. Human intestinal parasites in the past: new findings and a review. Mem Inst Oswaldo Cruz. 2003;98: 103–118.

89. Fugassa MH, Beltrame MO, Sardella NH, Civalero MT, Aschero C. Paleoparasitological results from coprolites dated at the Pleistocene–Holocene transition as source of paleoecological evidence in Patagonia. J Archaeol Sci. 2010;37: 880–884. doi:10.1016/j.jas.2009.11.018

90. Le Bailly M, Bouchet F. Ancient dicrocoeliosis: occurrence, distribution and migration. Acta Trop. 2010;115: 175–180. doi:10.1016/j.actatropica.2010.03.004

91. Cabeza-Barrera I, Cabezas-Fernández T, Salas Coronas J, Vázquez Villegas J, Cobo F. *Dicrocoelium dendriticum*: an emerging spurious infection in a geographic area with a high level of immigration. Ann Trop Med Parasitol. 2011;105: 403–406. doi:10.1179/1364859411Y.0000000029

92. Mitchell PD. The Roman World. In: Mitchell PD, editor. Parasites in Past Civilizations and their Impact Upon Health. Cambridge: Cambridge University Press; 2023. pp. 53–64.

93. Ledger ML, Mitchell PD. Tracing zoonotic parasite infections throughout human evolution. Int J Osteoarchaeol. 2022;32: 553–564. doi:10.1002/oa.2786

94. Reinhard KJ. Parasitology as an interpretive tool in archaeology. Am Antiq. 1992;57: 231–245. doi:10.2307/280729

95. Else KJ, Keiser J, Holland CV, Grencis RK, Sattelle DB, Fujiwara RT, et al. Whipworm and roundworm infections. Nat Rev Dis Primers. 2020;6: 44. doi:10.1038/s41572-020-0171-3

Supporting Information

S1 Appendix. Materials, methods, and parasite literature review.
S2 Appendix. Parasite bait accession numbers and bait description.
S3 Appendix. Detailed results.

List of Figures

Fig 1. Map of archeological sites studied. Map indicates the location of sediment samples studied. The table on the right provides the site name, date of samples, and sample types. Map

created using ArcGIS software by Esri. ArcGIS and ArcMap are the intellectual property of Esri and are used herein under license. Copyright Esri. All rights reserved. Sources: Esri, TomTom, Garmin, FAO, NOAA, USGS, OpenStreetMap contributors, and the GIS User Community.

Fig 2. Summary of parasites recovered from each sample using microscopy, ELISA, and ancient DNA. Black bars represent egg concentrations identified using microscopy and blue bars represent DNA read count. Samples for which insufficient material was available for ELISA are crossed out and positive ELISA tests are marked with a +. [1]Ledger et al., 2019 [39]; [2]Anastasiou et al., 2014 [35]; [3]Mitchell et al., 2022 [44]; [4]Ledger et al., 2019 [40]; [5]Deforce et al., 2021 [43]; [6]Ledger et al., 2021 [34]; [7]Ledger et al., 2020 [42]; [8]Williams et al., 2017 [37]; [9]Ledger et al., 2018 [38]; [10]Knorr et al., 2019 [41]; [11]Yeh et al., 2015 [36].

Fig 3. Heatmap comparing concentration of *Ascaris* eggs recovered from each sample to percentage of LCA-assigned reads. *Ascaris* egg concentration is reported in eggs per gram based on microscopy and LCA-assigned reads include reads that were assigned to the Superfamily Ascaridoidea or lower and read count assigned to Ascaridoidea or lower. Samples are ordered in the table based on egg per gram concentration from highest to lowest concentration with dark red background for the lowest concentrations, yellow/orange mid-range and green the highest concentrations.

Fig 4. Deamination and fragment length distributions for *Ascaris* reads. Data generated using *mapDamage* showing C to T and G to A deamination patterns (top) and fragment length profile (bottom) for reads assigned to *Ascaris* in samples with highest read count.

Fig 5. Variation in morphological appearance of *Ascaris* eggs identified in samples with confirmed presence of *Ascaris* using aDNA methods. (A) fertilized egg from Arlon with mamillated coat; (B) fertilized egg from Arlon with mamillated coat; (C) fertilized decorticated egg from Mértola (image credit: Delaney Knorr); (D) fertilized decorticated egg from Mértola (image credit: Delaney Knorr); (E) fertilized decorticated egg from Vacone; (F) fertilized

decorticated egg from Vacone; (G and H) fertilized decorticated egg from Sardis with surface cracking; (I) fertilized decorticated egg from Sardis. Scale bars indicate 20 μm.

Fig 6. Temporal trends in parasite diversity before, during, and after the Roman period for the most common helminths recovered from archeological samples. For each parasite, bars indicate the proportion of studied sites where each parasite was recovered for pre-Roman, Roman, and post-Roman sites with 95% confidence intervals. See S1 Appendix for individual studies used to generate temporal trends.

Figure 2

Click here to access/download;Figure;Fig2_ResultsFigure_Finalized.tif

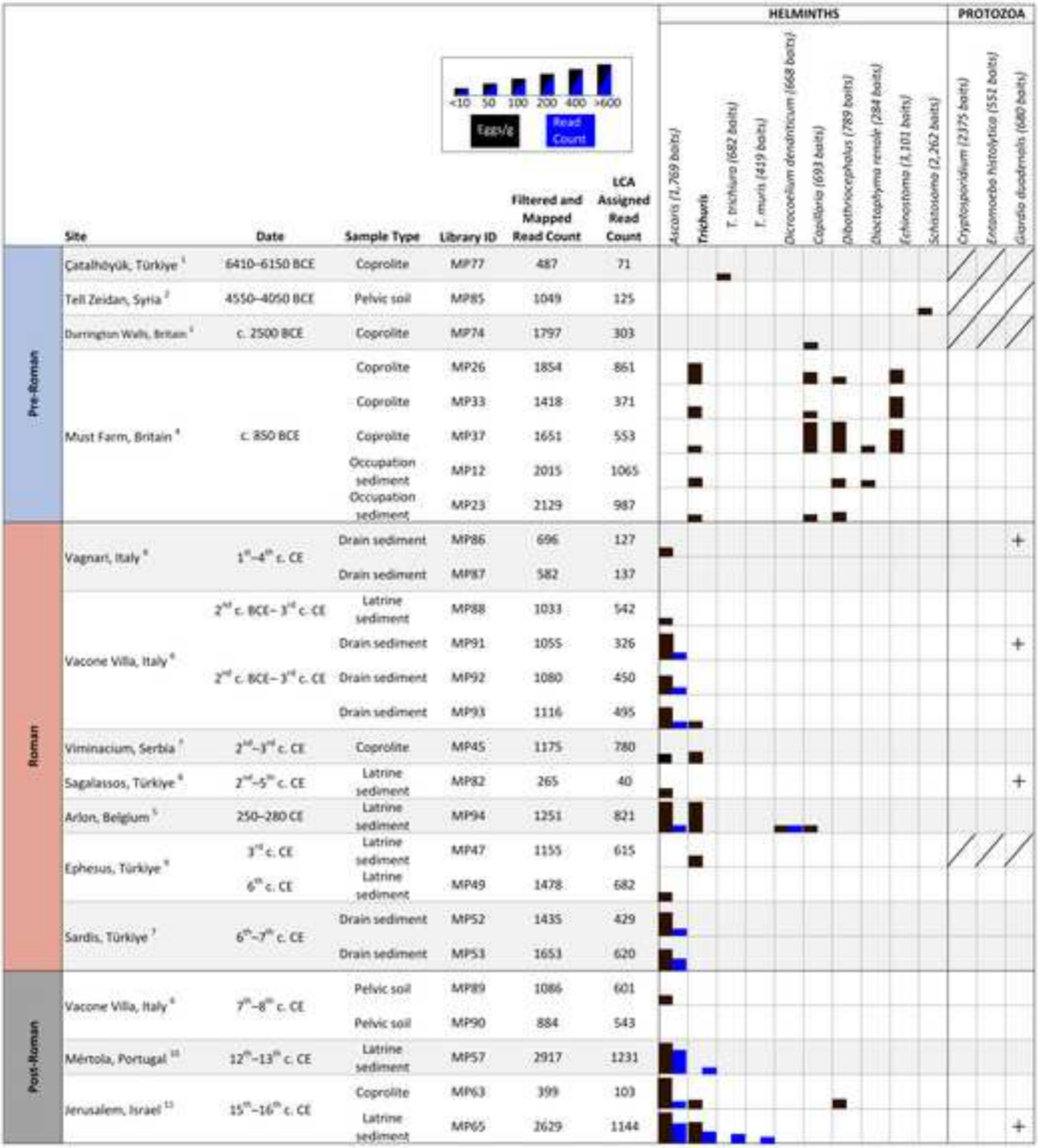

Figure 3

Click here to access/download;Figure;Fig3_HeatMap.tif

Site	Sample Type	DNA Library ID	*Ascaris* (eggs per gram)	% LCA Assigned (Ascaridoidea or Lower)	Read Count (Ascaridoidea or Lower)
Jerusalem	Cesspit sediment	MP65	1580	21.8%	249
Mértola	Latrine	MP57	1110	24.1%	297
Arlon	Latrine	MP94	990	0.5%	4
Jerusalem	Coprolite	MP63	595	5.8%	6
Vacone	Drain sediment	MP91	405	1.8%	6
Sardis	Drain sediment	MP52	305	1.2%	5
Sardis	Drain sediment	MP53	205	9.7%	60
Vacone	Drain sediment	MP93	185	1.4%	7
Vacone	Drain sediment	MP92	140	1.6%	7
Vacone	Pelvic soil	MP89	30	0.0%	0
Vagnari	Drain sediment	MP86	25	2.4%	3
Ephesus	Latrine	MP49	25	0.0%	0
Sagalassos	Latrine	MP82	20	0.0%	0
Vacone	Latrine	MP88	5	0.0%	0

Figure 4

Click here to access/download;Figure;Fig4_mapDamage.tif

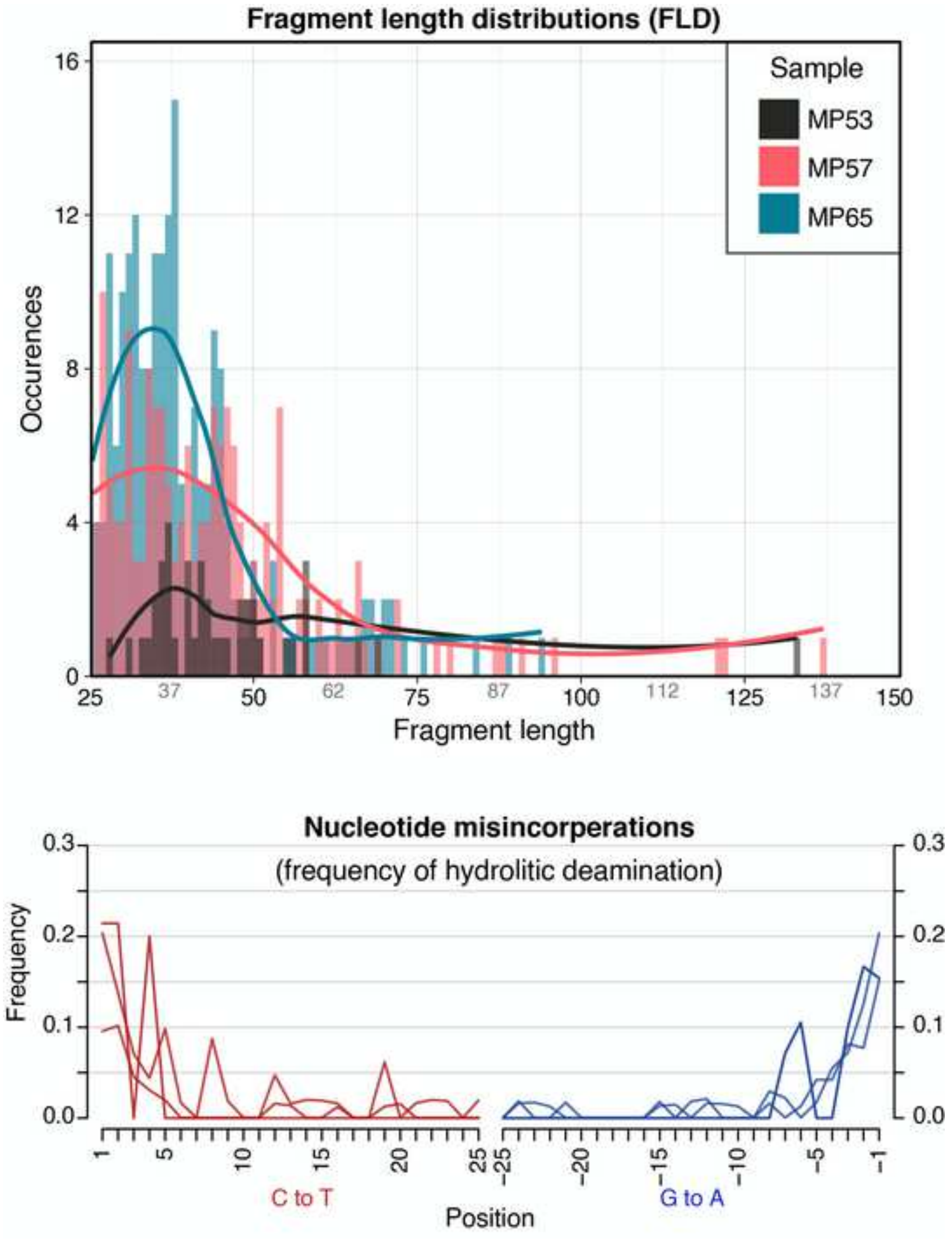

Figure 5

Click here to access/download;Figure;Fig5_Ascaris.tif

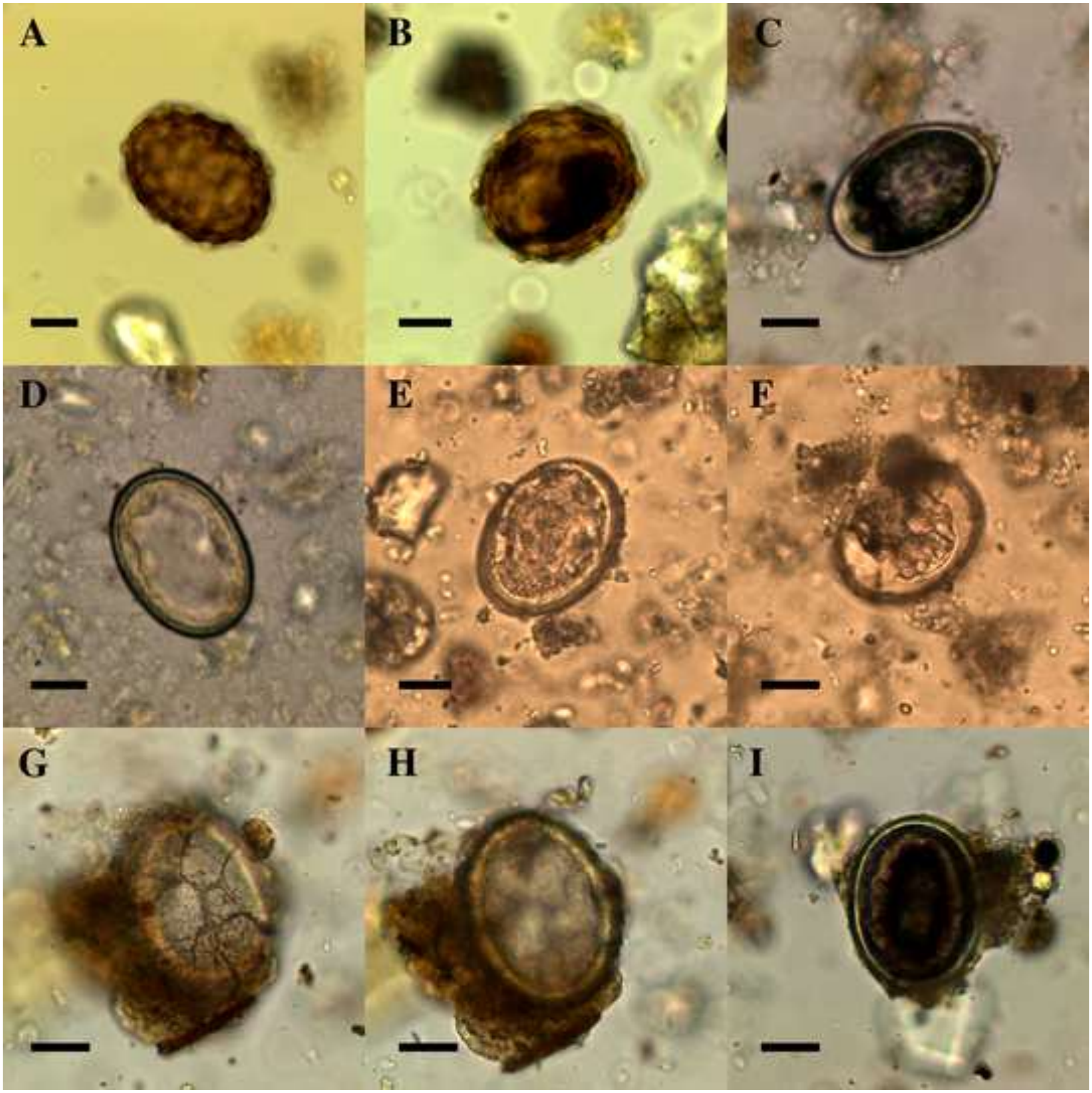

Figure 6

Click here to access/download;Figure;Fig6_TemporalChanges.tif

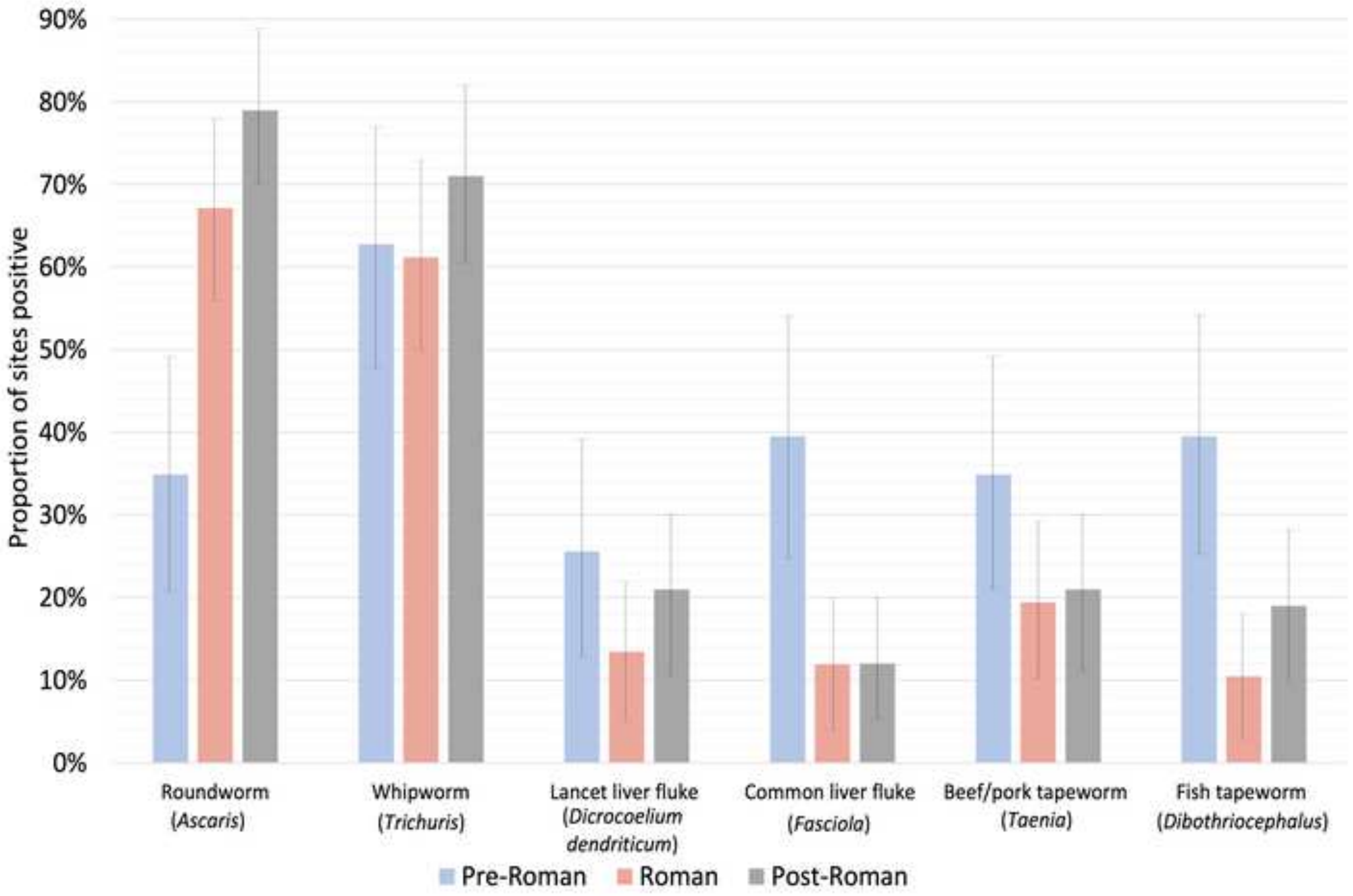

Figure 1 Click here to access/download;Figure;Fig1_SiteLocationUpdate.tif

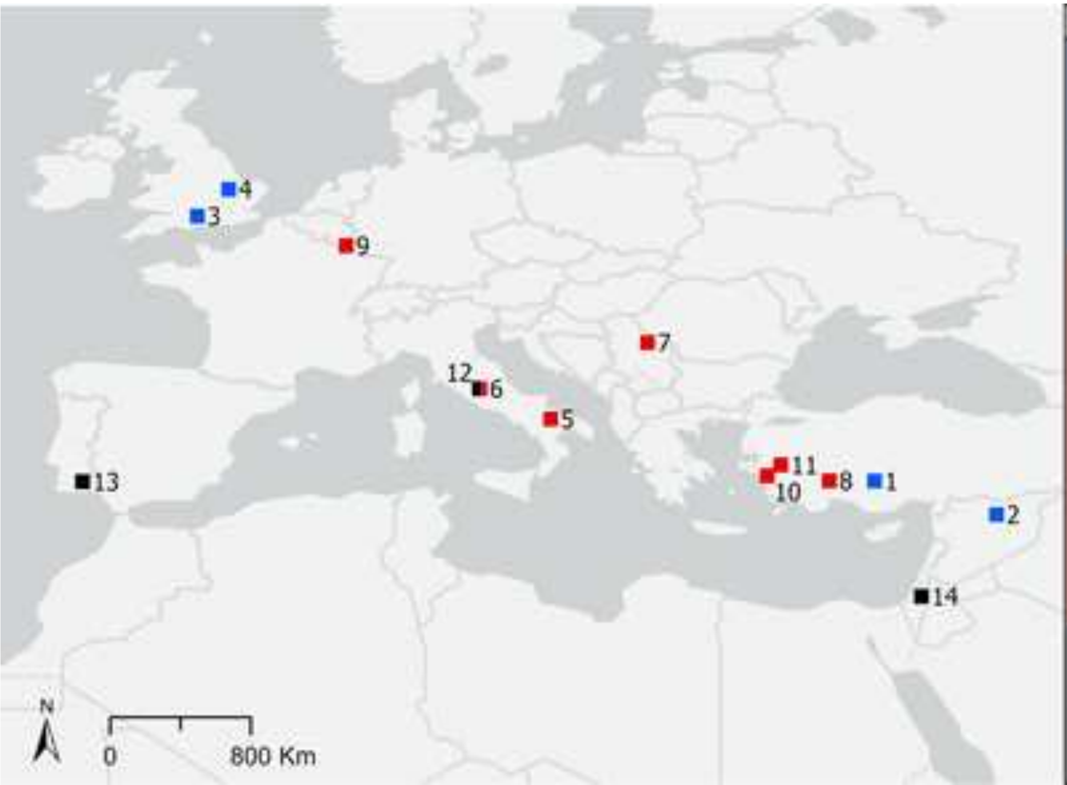

Time Period	Map Label	Site Name	Date	Sample Type
Pre-Roman	1	Çatalhöyük	6410–6150 BCE	Coprolite
	2	Tell Zeidan	4550–4050 BCE	Pelvic soil
	3	Durrington Walls	c. 2500 BCE	Coprolite
	4	Must Farm	c. 850 BCE	Coprolite, Occupation sediment
Roman	5	Vagnari	1st–4th c. CE	Drain
	6	Vacone	2nd c. BCE– 3rd c. CE	Drain, Latrine
	7	Viminacium	2nd–3rd c. CE	Coprolite
	8	Sagalassos	2nd–5th c. CE	Latrine
	9	Arlon	250–280 CE	Latrine
	10	Ephesus	3rd–6th c. CE	Latrine
	11	Sardis	6th–7th c. CE	Drain
Post-Roman	12	Vacone	7th–8th c. CE	Pelvic soil
	13	Mértola	12th–13th c. CE	Latrine
	14	Jerusalem	15th–16th c. CE	Latrine, Coprolite

Supporting Information S2

Click here to access/download
Supporting Information
S2_Appendix_ParasiteBaits.xlsx

Click here to access/download
Supporting Information
S3_Appendix_Results.xlsx

Click here to access/download
Supporting Information
S1_Appendix_Materials_Methods_TemporalData.docx

Response to Reviewers
PNTD-D-24-01526
Sedimentary ancient DNA as part of a multimethod paleoparasitology approach reveals temporal trends in human parasitic burden in the Roman period
PLOS Neglected Tropical Diseases

Comments to the author:
Please note that one of the reviews is uploaded as an attachment.
Response: Comments requiring detailed response are detailed below. We have also added comments to the attached pdf to clearly indicate the changes that were made for each comment.

Reviewers' comments:

Reviewer's Responses to Questions
Key Review Criteria Required for Acceptance?
As you describe the new analyses required for acceptance, please consider the following:

Methods
-Are the objectives of the study clearly articulated with a clear testable hypothesis stated?
-Is the study design appropriate to address the stated objectives?
-Is the population clearly described and appropriate for the hypothesis being tested?
-Is the sample size sufficient to ensure adequate power to address the hypothesis being tested?
-Were correct statistical analysis used to support conclusions?
-Are there concerns about ethical or regulatory requirements being met?

Reviewer #1: This work is well designed and followed valuable points of views to illustrate results to the readers. Those who are interested to work on paleoparasitology will encourage to expand their program in to all three possibilities of identifying parasites of ancient times.
As the last word I feel well satisfied by this manuscript. I would like to congratulate the organizers of this very good team work

Reviewer #2: -Are the objectives of the study clearly articulated with a clear testable hypothesis stated? Yes
-Is the study design appropriate to address the stated objectives? Yes
-Is the population clearly described and appropriate for the hypothesis being tested? Yes
-Is the sample size sufficient to ensure adequate power to address the hypothesis being tested?
There is a very small sample size in this study, however, this tends to be the case when

working with ancient DNA. The limitation is clearly recognized in the study.
-Were correct statistical analysis used to support conclusions? Yes
-Are there concerns about ethical or regulatory requirements being met? No

Reviewer #3: (No Response)

Results
-Does the analysis presented match the analysis plan?
-Are the results clearly and completely presented?
-Are the figures (Tables, Images) of sufficient quality for clarity?
Reviewer #1: (No Response)
Reviewer #2: -Does the analysis presented match the analysis plan? Yes

-Are the results clearly and completely presented? Yes
-Are the figures (Tables, Images) of sufficient quality for clarity? Yes. Nevertheless, data from supplementary figures F1 to F3 and Figure 2 could have been combined in a presence/absence heatmap, showing the congruence between this study's findings and that of published results.
Response: Thank you for the thoughtful comment, please find our detailed response to this suggestion raised again below.

Reviewer #3: (No Response)

Conclusions
-Are the conclusions supported by the data presented?
-Are the limitations of analysis clearly described?
-Do the authors discuss how these data can be helpful to advance our understanding of the topic under study?
-Is public health relevance addressed?

Reviewer #1: (No Response)

Reviewer #2: -Are the conclusions supported by the data presented? Yes
-Are the limitations of analysis clearly described? The authors highlight the constraints of their limited sample size and make grounded conclusions accordingly.
-Do the authors discuss how these data can be helpful to advance our understanding of the topic under study? Yes
-Is public health relevance addressed? Yes

Reviewer #3: (No Response)

Editorial and Data Presentation Modifications?

Use this section for editorial suggestions as well as relatively minor modifications of existing data that would enhance clarity. If the only modifications needed are minor and/or editorial, you may wish to recommend “Minor Revision” or “Accept”.

Reviewer #1: (No Response)

Reviewer #2: An additional figure in the article illustrating the presence or absence of parasites in previous studies alongside this study's findings would have been helpful to the reader, highlighting the congruence of results.
However, this is just a suggestion, and it does not condition the suitability of the article.
Response: Thank you for your thoughtful consideration of the presentation of the data and suggestion for additional figures. We have decided not to add an additional figure summarizing presence and absences of parasites in previously published data, as in a simplified form we think this would be repetition of Figure 6, which aims to show presence of the most commonly identified species through time (and which covers nearly all species we identified in our samples). To include the full range of species (from all 168 sites) would result in a very complex figure or significant summarization (as has already been done) with careful statistical consideration given the inherent biases in this data. We feel that to do this well, we would need to provide a good explanation of statistical methods and limitations within the text thus lengthening the article to be beyond the current scope. One of the main reasons it is difficult to quantify these results in any further detail than we have currently done is due to publication bias. We expect there have been many studies undertaken which did not yield preserved parasite eggs thus were never published. While we can make inferences on which parasites predominated over time based on their repeated detection in archaeological samples it is much more difficult to understand what the absence of certain species indicates (e.g. lack of appropriate samples being studied, true absence, poor preservation of some species, varied preservation conditions in different geographic areas, etc.)

Reviewer #3: (No Response)

Summary and General Comments

Use this section to provide overall comments, discuss strengths/weaknesses of the study, novelty, significance, general execution and scholarship. You may also include additional comments for the author, including concerns about dual publication, research ethics, or publication ethics. If requesting major revision, please articulate the new experiments that are needed.

Reviewer #1: (No Response)

Reviewer #2: The article looks into the parasitic load of pre-Roman, Roman and post-Roman populations in Europe through the analysis of coprolites. Using a combination of microscopy, ELISA and (antigens) and aDNA shotgun sequencing the study compares

each method and recommends a combination of all three for optimal parasitic diagnostics.

The article is very well structured and easy to follow in a very detailed and concise manner. The discussion is particularly insightful and does not overreach conclusionwise. However, ELISA is very briefly discussed in the paper despite it being recognized as one of the three pilar methods for parasite diagnostics. The lack of discussion of ELISA findings and recognizing a bias/contamination in some ELISA results, despite no explanation or correction to this given in the paper or the appendix, makes ELISA substantially less convincing than microscopy and aDNA.
The analysis was straight to the point. From the appendix it is also clear that very thorough research was done on published Paleoparasitological studies, providing records of parasites found in these studies throughout time. The study could have added this information to a figure in the same format as Figure 2, but presence or abscence of a parasite as opposed to quantitative analyses, including the findings of previous works, giving a more in-depth geospatial representation of parasitic loads through time from this study and previous studies.

Furthermore, the authors have declared the data will be publicly available. This has not been done yet, but is expected shortly after submission.
Response: We fully agree that the results from ELISA have been less convincing than the other methods and the inability to replicate them with any aDNA analyses thus far raises questions about the specificity and sensitivity of these assays. One of the main reasons for presenting the indeterminate results from Must Farm is to highlight that this should be an area of focus for future research. However, we note that this result is very unusual compared to previous studies. We have highlighted in the discussion some features of the validation of the kits we used and our recommendation for further research to better understand the limitations of ELISA in paleoparasitology.
"Furthermore, *Giardia duodenalis* antigen was detected using ELISA from four sites, and although our bait set did include protozoa, we were not able to recover any DNA sequences from *Giardia duodenalis*. Thus, assuming that the ELISA signal is real, it does continue to have a unique value in that it is a useful additional method for maximizing recovery of common intestinal protozoa, which are rarely preserved well enough to be visualized using microscopy and have only rarely been detected in aDNA studies thus far [26]. However, the indeterminate results from Must Farm highlight an important limitation of ELISA which is the potential for cross-reactivity with unknown antigens that may be found in soil. Commercial ELISA kits used in this and other studies have very high sensitivity and specificity and in their validation there was no cross-reactivity reported for other parasite species tested. However, further exploration of how these performance characteristics change when applied to archaeological samples is needed. Further, optimization of aDNA methods for detection of protozoa would be valuable to confirm ELISA results with a second method."

We have discussed above the recommendation for creating an additional figure similar to Figure 2 that includes all previous publications, but given the amount of data that

would need to be included and the inherent biases in this data we do not feel this fits within the scope of the current paper.

The raw data has been submitted to NCBI SRA and accession number provided in the manuscript.

Reviewer #3: (the pdf with the correction is in the attachment)
The article provides a clear, concise, and relevant study on paleoparasitology and paleogenomics. The authors successfully develop a context that is well-researched and coherent, establishing the objectives of the study. However, there is information that is basically part of the results and discussion on the topic of Methodology, as well as phrases of discussion on the topic of Results; I marked some of these phrases in the pdf file. Regrettably, it is stated in the introduction that the paleogenetic analysis of parasites has been little addressed in the past, the authors ignore an extensive contribution of studies mainly in archaeological and historical sites, especially in Brazil, but also Chile and Argentina. The taxonomic writing should be corrected along with the manuscript; I also made some corrections in the pdf file.
Response: Thank you for your very thoughtful consideration of the manuscript and suggestions that have certainly improved the manuscript.

We have moved the noted sections from the methods/results to the discussion.

In the list of previously published studies using paleogenetic methods in parasitology we cite the most recent review on this topic which does include the extensive work done in South America and we follow this with a list of key papers published since that time, one of which is Iniguez et al., 2022 a study on material from Brazil. We do, however, recognize that there have been additional publications, especially those focused on non-human parasites that we have not cited and thus we have added additional examples (Beltrame et al., 2020; Guedes et al., 2020; Wiscovitch-Russo et al., 2020). We have also further specified that our review is focused on human paleoparasitology which is one of the reasons that all of the studies undertaken in Central and South America have not been included.

We have also reattached the marked-up pdf with responses to each individual comment made by Reviewer 3. Below we have included a few responses that were too long to include in the marked-up pdf.

I have only two main worries.

1) How the species Ascaris lumbricoides and A. suum were identified since these two parasites are not genetically separated using most of the molecular markers?
   Response: Thank you for your very thoughtful review and suggestions. We have provided more explanation and further analysis of these reads to respond to your question. Using our bioinformatic approach which made use of MEGAN to taxonomically assign reads, 3 reads from MP-57 were assigned to *A. lumbricoides*. However, as you know the taxonomic distinction of *Ascaris suum* and *Ascaris lumbricoides* has been questioned thus we mapped these reads to

both of the reference sequences from these two species and found that reads have 100% identity to both *A. suum* and *A. lumbricoides* thus providing uncertainty in their assignment to the species-level. We have amended the results section as follows “In the latrine sediment from Mértola, Portugal three of these reads were assigned to the species-level as Ascaris lumbricoides, the human infecting species of Ascaris using MEGAN. However, when they were mapped to the reference genomes of both *Ascaris lumbricoides* (GCA_015227635.1) and *Ascaris suum* (GCA_013433145.1) these reads had 100% identity with both species as would be expected based on the limited genetic diversity between these species (see S1 Appendix)”

We have also further explored this in the discussion. “In latrine sediment from the medieval site of Mértola, *Ascaris* reads were assigned to the species-level as *Ascaris lumbricoides* by MEGAN, however on further interrogation of these reads we find that they are not unique to *Ascaris lumbricoides* but also match equally well to *Ascaris suum* (classically considered to infect pigs). Given extremely little genetic differences between these species, it is not surprising that for all other samples, reads could only be assigned to the genus-level [64,65] and in the majority of our samples we recovered *Ascaris* DNA. Attempts at molecular characterization of *A. suum* and *A. lumbricoides* have revealed very low divergence rates in ITS1 and mitochondrial regions (~0.01%) [66,67]. Comparison of full mitochondrial genomes of *A. lumbricoides* and *A. suum* from China showed 98.1% sequence identity [64]. In addition, species-designation has been questioned on the basis of experimental studies showing that the life-cycle of both species can be completed in either host [68–70]. Thus, the studies above and other authors have questioned the species distinction between *A. lumbricoides* and *A. suum* [71].”
Finally, we have made sure these results are clearly reflected in the conclusions.

2) The identification of Ascaris spp. from atypical morphology eggs is controversial since the DNA was not extracted specifically from these atypical eggs but from the sediment that could be, in fact, positive to the parasite.
Response: This is an important point, as the reason for extracting from sediment directly rather than isolated eggs was to increase the potential to recover DNA from parasites from which eggs were not preserved or preserved well enough to be identified or would have been sieved out of the sample during concentration of eggs with sieving. While we agree that the DNA recovered could have been from the sediment and not the decorticated eggs themselves (and made sure to indicate this in the text), it seems most likely that they represent the preserved *Ascaris* eggs as in most cases the only parasite sequences recovered from these samples were identified as *Ascaris*. In comparison to freshly preserved modern eggs the eggs can be challenging to identify as many are decorticated and mineralized, however the morphology of the decorticated eggs is consistent with *Ascaris* as has been discussed in the numerous publications on these sites previously. Similarly, the correlation between amount of DNA recovered and concentration of eggs would support the origin of the DNA being from these

eggs. We have however, noted the limitation in our methodological approach and ensured that we are not suggesting we have confirmed without a doubt that the DNA is from the atypical eggs.

We have added the following to the discussion, “We cannot be certain from which source our *Ascaris* DNA stems. As it did not come from the eggs directly, it is possible that Ascaris DNA was present in the sediment outside of these decorticated eggs. However, given the correlation of egg concentrations with DNA content in the samples (see Fig 3), it is most likely that this DNA originated from the eggs themselves. Importantly, the overall morphological consistency of these eggs with decorticated *Ascaris* eggs would argue in favor of the origins of these DNA molecules. Even in modern studies, decorticated roundworm eggs can be easily misidentified [59]. Thus, the addition of aDNA analysis can confirm identification in cases of uncertainty due to taphonomic changes and poor egg preservation and allows us to more confidently explore the morphologic appearance of ancient eggs.” We have also made sure the wording of the conclusions reflects this possibility.

Additional responses to comments in the attached pdf from Reviewer 3:

- All directly suggested changes were made. In the points to follow we respond to suggestions that were not very minor (adding a citation, spelling, taxonomic suggestion).
- It was questioned why we made a distinction between paleofeces and coprolites. The reason for this is that coprolites generally refer to mineralized or fossilized feces and in most cases the material we are working with is not mineralized (as in the samples from Must Farm) thus paleofeces is a broader term that can apply to the diversity of samples that we studied. While some researchers may further argue that coprolites are fully fossilized thus would not contain biological (e.g. DNA) material, we have retained use of the word coprolite when working with material representing an individual defecation event (for ease of understanding how this is different from latrine sediment and pelvic sediment) as is commonly done in the literature.
- It was asked to explain chemical digestion buffer, as this is a very standard approach for any molecular studies we have changed this to ‘lysis buffer’ which is a more commonly used term and explained that its use is to breakdown organic material and release DNA, we have also added a reference to the Appendix at the start of our methods section where the full reagent list and concentrations of each can be found.
- It was requested to have kit, equipment and manufacturer added into the methods section. This information can be found in the Appendix where the methods are explained in detail (8 pages in length) so that they can be replicated. In addition, it was requested to add the concentration of only one reagent, Proteinase K, as we have not done this for any other reagents we have left this in the Appendix. We have added the manufacturer of each reagent to the Appendix where they are listed. We have additionally indicated at the start of the methods section that further details can be found in the Appendix.

- On Line 268 (marked up pdf) it was noted that Metazoa and Sar could contain parasites. We agree and have further explained that the reads assigned to these Kingdoms could not be assigned below the Clade-level thus while they might in fact be reads from parasites they are relatively uninformative.
- In all instances of Genus name listed without a species Reviewer 3 has highlighted these and provided the comment “wrong taxonomic writing”, we have for ease of reading not always added sp. or spp. after a Genus name. However, in most cases highlighted by Reviewer 3 these have now been added for clarification. The exception to this is for *Ascaris* because of the debate discussed in the paper on whether we have two separate species *A. lumbricoides* and *A. suum* or one and we simply cannot assign our DNA reads to either species. We recognize there may be some debate amongst researchers if it is acceptable to use a Genus name without a species name or abbreviation but for ease of reading, we prefer to use this convention. This is consistent with other publications in PLOS Neglected Tropical Diseases. See for example: Søe, M. J., Kapel, C. M. O., & Nejsum, P. (2016). *Ascaris* from humans and pigs appear to be reproductively isolated species. *PLoS Neglected Tropical Diseases*, *10*(9), e0004855. https://doi.org/10.1371/journal.pntd.0004855

PLOS Neglected Tropical Diseases

Sedimentary ancient DNA as part of a multimethod paleoparasitology approach reveals temporal trends in human parasitic burden in the Roman period

--Manuscript Draft--

**Manuscript Number:**	PNTD-D-24-01526
**Full Title:**	Sedimentary ancient DNA as part of a multimethod paleoparasitology approach reveals temporal trends in human parasitic burden in the Roman period
**Short Title:**	Sedimentary ancient DNA as part of a multimethod paleoparasitology approach
**Article Type:**	Research Article
**Keywords:**	Ancient DNA; sedimentary DNA; ELISA; microscopy; helminths; protozoa; Roman Empire
**Abstract:**	The detection of parasite infections in past populations has classically relied on microscopic analysis of sediment samples and coprolites. In recent years, additional methods have been integrated into paleoparasitology such as, enzyme-linked immunosorbent assay (ELISA) and ancient DNA (aDNA). The aim of this study was to evaluate a multimethod approach for paleoparasitology using microscopy, ELISA, and sedimentary ancient DNA (sedaDNA) with a parasite-specific targeted capture approach and high-throughput sequencing. Using 26 samples dating from c. 6000 BCE to 1500 CE that were previously analyzed with microscopy and ELISA, we aimed to more accurately detect and reconstruct parasite diversity in the Roman Empire and compare this diversity to earlier and later time periods to explore temporal changes in parasite diversity. Microscopy was found to be the most effective technique for identifying the eggs of helminths, with 8 taxa identified. ELISA was the most sensitive for detecting protozoa that cause diarrhea (notably Giardia duodenalis). Parasite DNA was recovered from 9 samples, with no parasite DNA recovered from any pre-Roman sites. Sedimentary DNA analysis identified whipworm at a site where only roundworm was visible on microscopy, and also showed that the whipworm eggs at another site came from two different species (T. trichiura and T. muris). Our results show that a multimethod approach provides the most comprehensive reconstruction of parasite diversity in past populations. In the pre-Roman period, taxonomic diversity included a mixed spectrum of zoonotic and foodborne parasites, together with whipworm, which is spread by ineffective sanitation. We see a marked change during the Roman and medieval periods with an increasing dominance of parasites transmitted by ineffective sanitation, especially roundworm, whipworm and protozoa that cause diarrheal illness.
**Additional Information:**	
**Question**	**Response**
**Government Employee**<br><br>Are you or any of the contributing authors an employee of the United States government?<br><br>Manuscripts authored by one or more US Government employees are not copyrighted, but are licensed under a CC0 Public Domain Dedication, which allows unlimited distribution and reuse of the article for any lawful purpose. This is a legal requirement for US Government employees.	No - No authors are employees of the U.S. government.

This will be typeset if the manuscript is accepted for publication.	
**Financial Disclosure**<br><br>Enter a financial disclosure statement that describes the sources of funding for the work included in this submission. Review the submission guidelines for detailed requirements. View published research articles from *PLOS Neglected Tropical Diseases* for specific examples.<br><br>This statement is required for submission and **will appear in the published article** if the submission is accepted. Please make sure it is accurate.<br><br>**Funded studies**<br>Enter a statement with the following details:<br>• Initials of the authors who received each award<br>• Grant numbers awarded to each author<br>• The full name of each funder<br>• URL of each funder website<br>• Did the sponsors or funders play any role in the study design, data collection and analysis, decision to publish, or preparation of the manuscript?<br><br>Did you receive funding for this work?	Yes
Please add funding details.<br>as follow-up to "**Financial Disclosure**<br><br>Enter a financial disclosure statement that describes the sources of funding for the work included in this submission. Review the submission guidelines for detailed requirements. View published research articles from *PLOS Neglected Tropical Diseases* for specific examples.<br><br>This statement is required for submission and **will appear in the published article** if the submission is accepted. Please make sure it is accurate.	This work was supported by The Social Sciences and Humanities Research Council of Canada [752-2016-2085], a Tidmarsh Cambridge Scholarship from the Cambridge Commonwealth, European and International Trust and Trinity Hall College, and the Society for the Promotion of Roman Studies to MLL.<br>TJM and HNP were funded by the CANA Foundation, the Garfield Weston Foundation, the Social Sciences and Humanities Research Council of Canada, CIFAR, and Natural Sciences and Engineering Research Council of Canada.

**Funded studies**<br>Enter a statement with the following details:<br>• Initials of the authors who received each award<br>• Grant numbers awarded to each author<br>• The full name of each funder<br>• URL of each funder website<br>• Did the sponsors or funders play any role in the study design, data collection and analysis, decision to publish, or preparation of the manuscript?<br><br>Did you receive funding for this work?"	
Please select the country of your main research funder (please select carefully as in some cases this is used in fee calculation).<br>as follow-up to "**Financial Disclosure**<br><br>Enter a financial disclosure statement that describes the sources of funding for the work included in this submission. Review the submission guidelines for detailed requirements. View published research articles from *PLOS Neglected Tropical Diseases* for specific examples.<br><br>This statement is required for submission and **will appear in the published article** if the submission is accepted. Please make sure it is accurate.<br><br>**Funded studies**<br>Enter a statement with the following details:<br>• Initials of the authors who received each award<br>• Grant numbers awarded to each author<br>• The full name of each funder<br>• URL of each funder website<br>• Did the sponsors or funders play any role in the study design, data collection and analysis, decision to publish, or preparation of the manuscript?<br><br>Did you receive funding for this work?"	CANADA - CA
**Competing Interests**	The authors declare no competing interests.

On behalf of all authors, disclose any competing interests that could be perceived to bias this work.<br><br>This statement will be typeset if the manuscript is accepted for publication.<br><br>Review the instructions link below and PLOS NTDs' competing interests policy to determine what information must be disclosed at submission.	
**Data Availability**<br><br>Provide a **Data Availability Statement** in the box below. This statement should detail where the data used in this submission can be accessed. This statement will be typeset if the manuscript is accepted for publication.<br><br>Before publication, authors are required to make all data underlying their findings fully available, without restriction. Review our PLOS Data Policy page for detailed information on this policy. Instructions for writing your Data Availability statement can be accessed via the Instructions link below.	The data that support the findings of this study are publicly available from the NCBI Sequence Read Archive with the submission number SUB14636687

Powered by Editorial Manager® and ProduXion Manager® from Aries Systems Corporation

Manuscript

Click here to access/download;Manuscript;Multimethod_Approach_Palaeopar

Sedimentary ancient DNA as part of a multimethod paleoparasitology approach reveals temporal trends in human parasitic burden in the Roman period

Marissa L. Ledger[1,2*#], Tyler J. Murchie[2,3], Zachery Dickson[4], Melanie Kuch[2], Scott D. Haddow[5], Christopher J. Knüsel[6], Gil J. Stein[7], Mike Parker Pearson[8], Rachel Ballantyne[9], Mark Knight[10], Koen Deforce[11,12], Maureen Carroll[13], Candace Rice[14], Tyler Franconi[14], Nataša Šarkić[15], Saša Redžič[16], Erica Rowan[17], Nicholas Cahill[18], Jeroen Poblome[19], Maria de Fátima Palma[20], Helmut Brückner[21], Piers D. Mitchell[1], Hendrik Poinar[2,22,23]

[1] Department of Archaeology, The Henry Wellcome Building, University of Cambridge, Cambridge, UK

[2] McMaster Ancient DNA Centre, Department of Anthropology, McMaster University, Hamilton, Ontario, Canada

[3] Hakai Institute, Heriot Bay, British Columbia, Canada

[4] Department of Biology, McMaster University, Hamilton, Ontario, Canada

[5] Department of Cross-Cultural and Regional Studies, University of Copenhagen, Copenhagen, Denmark

[6] CNRS, MC, PACEA, UMR 5199, Université de Bordeaux, Pessac, France

[7] Institute for the Study of Ancient Cultures (ISAC), University of Chicago, Chicago, Illinois, USA

[8] Institute of Archaeology, University College London, London, UK

[9] McDonald Institute for Archaeological Research, University of Cambridge, Cambridge, UK

[10] Cambridge Archaeological Unit, Department of Archaeology, University of Cambridge, Cambridge, UK

[11] Department of Archaeology, Ghent University, Ghent, Belgium

[12] Royal Belgian Institute of Natural Sciences, Brussels, Belgium

[13] Department of Archaeology, University of York, York, UK

[14] Joukowsky Institute for Archaeology & the Ancient World and Department of Classics, Brown University, Providence, Rhode Island, USA

[15] Aita Bioarchaeology, Barcelona, Spain

[16] Institute of Archaeology, Belgrade, Serbia
[17] Department of Classics, Royal Holloway, University of London, UK
[18] Department of Art History, University of Wisconsin–Madison, Wisconsin, USA
[19] Unit of Archaeology, KU Leuven, Belgium
[20] Campo Arqueológico de Mértola/CEAACP, Mértola, Portugal
[21] Institute of Geography, University of Cologne, Cologne (Köln), Germany
[22] Department of Biochemistry and Biomedical Sciences, McMaster University, Hamilton, Ontario, Canada
[23] Michael G. DeGroote Institute for Infectious Disease Research, McMaster University, Hamilton, Ontario, Canada

[#] Current Address: Department of Pathology and Molecular Medicine, McMaster University, Hamilton, Ontario, Canada

* Corresponding author
E-mail: ledgerm@mcmaster.ca (MLL)

Abstract

The detection of parasite infections in past populations has classically relied on microscopic analysis of sediment samples and coprolites. In recent years, additional methods have been integrated into paleoparasitology such as, enzyme-linked immunosorbent assay (ELISA) and ancient DNA (aDNA). The aim of this study was to evaluate a multimethod approach for paleoparasitology using microscopy, ELISA, and sedimentary ancient DNA (sedaDNA) with a parasite-specific targeted capture approach and high-throughput sequencing. Using 26 samples dating from c. 6000 BCE to 1500 CE that were previously analyzed with microscopy and ELISA, we aimed to more accurately detect and reconstruct parasite diversity in the Roman Empire and compare this diversity to earlier and later time periods to explore temporal changes in parasite diversity. Microscopy was found to be the most effective technique for identifying the eggs of helminths, with 8 taxa identified. ELISA was the most sensitive for detecting protozoa that cause diarrhea (notably *Giardia duodenalis*). Parasite DNA was recovered from 9 samples, with no parasite DNA recovered from any pre-Roman sites. Sedimentary DNA analysis identified whipworm at a site where only roundworm was visible on microscopy, and also showed that the whipworm eggs at another site came from two different species (*T. trichiura* and *T. muris*). Our results show that a multimethod approach provides the most comprehensive reconstruction of parasite diversity in past populations. In the pre-Roman period, taxonomic diversity included a mixed spectrum of zoonotic and foodborne parasites, together with whipworm, which is spread by ineffective sanitation. We see a marked change during the Roman and medieval periods with an increasing dominance of parasites transmitted by ineffective sanitation, especially roundworm, whipworm and protozoa that cause diarrheal illness.

Keywords: Ancient DNA, sedimentary DNA, ELISA, microscopy, helminths, protozoa, Roman Empire

Author Summary

A multimethod approach in paleoparasitology, combining microscopy, ELISA and aDNA, provides a more complete reconstruction of past parasite diversity. Applying this multimethod approach to a diverse set of archaeological sediment samples from 6000 BCE to 1500 CE provided insights into temporal changes in parasite diversity in the human past. Microscopy can be used as an effective screening tool for helminths in paleofecal samples, while ELISA is necessary for detection of protozoa, and sedaDNA using targeted enrichment can identify additional taxa and confirm species identification. Targeted enrichment using a comprehensive parasite bait set allows for detection of ancient human parasites and recovery of ancient parasite DNA from as little as 0.25 g of sediment. This multimethod approach reveals that parasite diversity decreased in the Roman period with a decrease in zoonotic parasites and concurrent increase in fecal-oral parasites, a pattern which is consistent during the medieval period.

Introduction

Ancient DNA (aDNA) has increasingly been used to improve the detection and characterization of ancient human pathogens [1–4]. Paleogenetic studies of ancient human pathogens have aided in the determination of the causative agents of past epidemics and endemic diseases [3,5,6], identified extinct strains of bacteria and viruses [7,8], and reconstructed ancient genomes of these organisms, allowing for the calibration of mutation rates and investigation of evolutionary histories such as changes in virulence genes [9,10]. However, many infectious diseases, including most parasites, cannot be detected using skeletal remains which are commonly used in aDNA studies. Parasites contributed to a significant burden of disease in the past, however our ability to detect and study their evolutionary history has been hampered by difficulty accessing their DNA. or coprolites Paleofeces (preserved fecal material) are the best source for recovering preserved DNA from enteric pathogens including bacteria [11,12], parasites [13–15], and viruses [16,17]. The field of paleoparasitology has a long history of using sediment samples to study enteric parasites in past populations [18]. Samples used in

Have not added as see description below, we generally include coprolites as paleofeces

see response to reviewers for explanation of distinction between paleofeces and coprolites

why the distintion?

paleoparasitology include coprolites (partially mineralized feces), pelvic sediment from burials where the intestines decomposed, and sediment containing high amounts of human fecal material such as the fill of latrines and sewer drains. Using these samples to recover genetic material and human gastrointestinal pathogens opens another route for understanding past human health, disease, and lifeways, and provides insight into a large category of pathogens that cannot be diagnosed using skeletal remains.

The use of genetic methods to detect parasites in both clinical and ancient samples has lagged behind most other pathogens, with the exception of *Plasmodium* or malaria [19–21]. For decades, the main methods used in paleoparasitology were microscopy, and to a smaller extent, enzyme-linked immunosorbent assay (ELISA, an immunological method that can detect antigens from specific organisms of interest). In recent years, the number of studies using aDNA has increased (see [20] for a review; and subsequent studies [12–14,22–28]). However, aDNA analysis contributes only a small minority of the total investigations performed within paleoparasitology.

which decades?

False. A number of paleogenetic contributions for the study of oxiurids, ascarids, trypanosomatids, etc., in pre-contact and historic archaeological sites from Brasil, France and Argentina were ommited

numbers of citation out of order

See response to reviewers for additional citations added and further clarification that we have focused on human parasites. While these studies have been done, paleogenetic work in parasitology is still minimal compared to work on bacteria

Here we present a multimethod approach for recovery of ancient parasites using sediment samples from contexts known to contain fecal material, including latrines, sewers, soil from the pelvic area of skeletons, and coprolites. Our multimethod approach incorporates sedaDNA methods using a targeted enrichment approach for parasite-specific DNA, in conjunction with microscopy and ELISA. The aim of this multimethod approach was to generate a more complete understanding of parasite diversity in Europe and the Eastern Mediterranean during the Roman Empire as compared to pre- and post-Roman periods. We analyzed paleofecal samples originating from archaeological sites temporally spanning the Neolithic through the medieval period. We used sedaDNA extraction methods previously shown to increase aDNA recovery compared to commercial kits [29], paired with targeted enrichment in order to preferentially sequence parasite DNA of interest and avoid the high sequencing costs associated with deep shotgun sequencing for low abundance targets such as pathogenic organisms. This sedaDNA approach was originally designed for palaeoecological reconstructions from permafrost—here we show that this approach can also be used to recover the aDNA of human parasites from archaeological sediments. Similarly, we show that a multimethod

Added

Reference

approach to paleoparasitology results in a more comprehensive taxonomic recovery compared to only utilizing a single method. Applying this multimethod approach to paleofecal samples from the Neolithic through medieval periods reveals trends in parasite infection that demonstrate marked change in the dominant species during the Roman period.

Materials and Methods

Materials

We analyzed archaeological sediments from latrine fill, drain fill, coprolites, and soil from the pelvic area of skeletons. All samples came from human contexts in archaeological sites ranging from the Neolithic period (ca. 7000 BCE) through the medieval period (ca. 1500 CE) in regions that were at one time part of the Roman Empire (see Fig 1). A total of 14 sites were included in the study; four from the pre-Roman period, seven from the Roman period, and three from the post-Roman or medieval period. From some sites multiple samples were studied if available, thus a total of 26 samples were included (see Fig 2). Further information about each site and samples analyzed can be found in S1 Appendix.

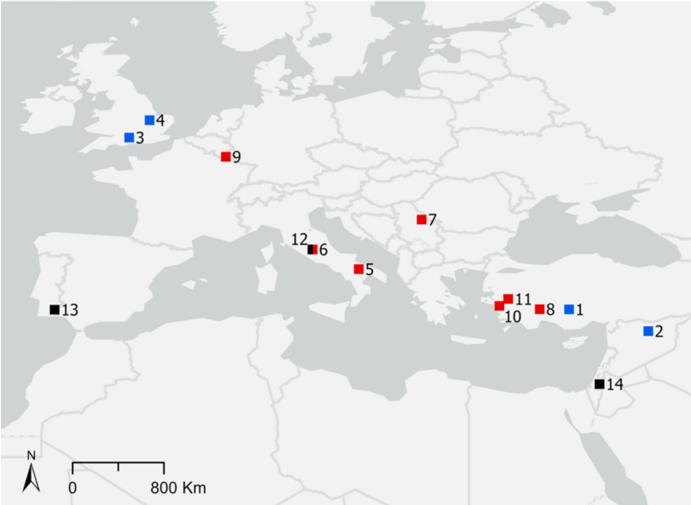

Time Period	Map Label	Site Name	Date	Sample Type
Pre-Roman	1	Çatalhöyük	6410–6150 BCE	Coprolite
	2	Tell Zeidan	4550–4050 BCE	Pelvic soil
	3	Durrington Walls	c. 2500 BCE	Coprolite
	4	Must Farm	865–840 cal BCE	Coprolite, Occupation sediment
Roman	5	Vagnari	1st–4th c. CE	Drain
	6	Vacone	2nd c. BCE– 3rd c. CE	Drain, Latrine
	7	Viminacium	2nd–3rd c. CE	Coprolite
	8	Sagalassos	2nd–5th c. CE	Latrine
	9	Arlon	250–280 CE	Latrine
	10	Ephesus	3rd–6th c. CE	Latrine
	11	Sardis	6th–7th c. CE	Drain
Post-Roman	12	Vacone	7th–8th c. CE	Pelvic soil
	13	Mértola	12th–13th c. CE	Latrine
	14	Jerusalem	15th–16th c. CE	Latrine, Coprolite

Fig 1. Map of archaeological sites studies. Map indicates location of sediment samples studied. The table on the right provides the site name, date of samples, and sample types. Corrected

Methods

All samples were first studied and reported on using microscopy and ELISA following standard methods [30,31]. The samples selected for this study had previously yielded parasite eggs or came from sites where eggs were found in contextually associated samples. For microscopy, a 0.2 g subsample was disaggregated in 0.5% trisodium phosphate. This was then microsieved to collect material between 20 and 160 µm. This fraction of the subsample was mixed with glycerol and viewed under a light microscope at 200x and 400x magnification to identify preserved helminth eggs based on morphological characteristics.

Reference

Added

Information of microscope

Added

For ELISA, a 1 g subsample was disaggregated in 0.5% trisodium phosphate and microsieved. Given the smaller size of protozoan cysts that cause diarrhea (less than 20 µm), the material in the catchment container below the 20 µm sieve was collected and concentrated to be used in commercial ELISA kits following the manufacturer's protocols. The kits used were *GIARDIA II*™, *E. HISTOLYTICA II*™, and *CRYPTOSPORIDIUM II*™ from TECHLAB, Inc (Blacksburg, USA). These kits are designed for detection of *Giardia duodenalis*, *Entamoeba histolytica*, and *Cryptosporidium* in modern human fecal samples. They employ a monoclonal antibody design and have high sensitivity and specificity in clinical validation studies. These kits and those from other manufacturers have been used to detect protozoan antigens in ancient human fecal samples in many previous studies [31–35].

taxonomic writing

For this comment and similar comment throughout see response to reviewers

For sedaDNA, 0.25 g of material was subsampled in dedicated clean rooms at the McMaster Ancient DNA Centre. Organic material was chemically and physically disintegrated using a chemical digestion buffer paired with garnet PowerBead tubes [29]. The samples were vortexed for 15 minutes to mechanically break down the organo-mineralized content and parasite eggs. Bead beating has been shown in clinical and archaeological studies to improve DNA recovery by breaking parasite eggs [36,37]. Proteinase K was added after bead beating, and tubes were continuously rotated in an oven set to 35° C overnight. The supernatant was removed and mixed with high-volume Dabney binding buffer as per published protocols [38]. Samples were then centrifuged at 4500 rpm at 4° C following Murchie et al. [29] for a minimum of 6 hours to remove inhibitors; if supernatant was clear at this point, the sample was removed from the centrifuge, and if not, it was centrifuged further for a total of 24 hours. Centrifugation

explain

Further detail added to methods and Appendix A to address the following comments on method description.

concentration and manufactures

Found in Supplementary Material with all other concentrations and manufacturers for reagents. We added a note to direct readers to this information at the start of the methods section

at refrigerated temperatures has been shown to increase the recovery of sedaDNA from complex environmental sample types through the precipitation of enzymatic inhibitory compounds commonly found in sediment and fecal samples [29]. Most published parasite DNA studies use modern commercial extraction kits, such as the PowerSoil DNA Extraction Kit, Mo Bio Laboratories [36,39–41] or QIAmp DNA Stool Mini Kit, Qiagen [42]. In our previous work, the sedaDNA methods employed here have been shown to increase DNA recovery from soil samples by 7–20 fold compared to commercial kits [29]. The remainder of the extraction and purification followed Dabney et al. [38] with the binding buffer passed through silica columns and eluted in 50 µL elution buffer with Tween.

no methodology, discussion

Moved

DNA libraries were prepared for Illumina sequencing using a double-stranded method [43,44] with minor modifications for blunt end repair as per our laboratory protocols (see S1 Appendix). A subset of four libraries (MP12, MP26, MP37, and MP53) were indexed separately for shotgun sequencing with a targeted sequencing depth of 2 million reads per sample. Targeted enrichment for parasite DNA was carried out using in-solution hybridization capture with RNA baits designed using modern parasite genomes (see S2 Appendix for reference sequence accession numbers). The parasite bait set contained 30,240-75mer baits based on mitochondrial and nuclear gene sequences retrieved from GenBank NCBI [45,46] from a list of identified human and animal infecting parasites and protozoa. Tiled baits were designed to capture 121 parasite taxa (see S2 Appendix). A progressive tiling approach was used to balance the number of baits for each taxa as best as possible. With this approach the bait design algorithm determined the maximum and minimum tiling density possible for each bait region. Taxa with few bait regions were maximally tiled to increase bait number while taxa with many potential bait regions were minimally tiled to balance bait number per taxa. The final average tiling density per taxa ranged from 2x to 30x. The number of baits for each taxa was ultimately limited by reference sequences available and ranged from 2–922 baits per taxa. Baits were purchased from Daicel Arbor Biosciences and our enrichment protocol followed the MYbaits V4 protocol supplied by the manufacturer. The targeted enrichment approach allows for the potential recovery of any of our targeted 121 taxa (S2 Appendix), which is the largest targeted approach in paleoparasitology to date without relying on shotgun sequencing. Many

kits and equipament used

Found in following paragraph where sequencing is described, also present in Supplementary material

paleogenetic studies in parasitology still rely on PCR which has a number of limitations. First, PCR amplicons often range between 50-600 bp, with most over 100 bp [39,47–49]. However, most aDNA fragments are smaller than 100 bp, with fragment length distribution modes generally around 40–50 bp [50,51]. Secondly, with targeted enrichment one can include baits designed to capture large genomic regions from mitochondrial and nuclear loci in one reaction.

discussion

Moved

Libraries for shotgun sequencing and targeted enrichment were quantified using qPCR and pooled to equimolar concentrations based on the molarity of each library. Pools were size-selected using electrophoresis followed by gel excision using the QIAquick Gel Extraction Kit. Paired-end sequencing was conducted using 2x90 sequencing chemistry on an Illumina HiSeq 1500 platform at the McMaster Farncombe Metagenomics Facility.

explain procedures, kit, equipament manufacter, City, here and in all the methodology

Added, also see comment above

All ancient DNA work was done in dedicated ancient DNA facilities with a unidirectional workflow, dedicated clean rooms, and standard precautions for prevention of contamination with modern DNA. For further details on ancient DNA methods see S1 Appendix.

This should be the firts topic of the methodology, and more details are needed

Moved

Bioinformatic workflow followed previously described in-house protocols for sedaDNA analysis (see [29]). In brief, *leeHom* [52] was used for trimming and merging, reads were then mapped using *network-aware-BWA* to parasite reference sequences that had been used to design the bait set. These mapped reads were size filtered to those greater than or equal to 24 bp and string de-duplicated with *NGSeXplore*. Filtered and mapped reads were run through BLASTn to return the top 100 taxonomic hits. MEGAN was then used to assign these reads to the lowest common taxonomic rank. For further detail on bioinformatic workflow see S1 Appendix.

Results

Pre-Roman Periods

Microscopic analysis of all samples described here was previously published. *Trichuris trichiura* (whipworm) was found in the human coprolite from 6410–6150 BCE Çatalhöyük, Türkiye [53]. *Schistosoma* sp. was found in pelvic soil from a burial (5800–4000 BCE) in Tell Zeidan, Syria [54]. One coprolite from the Neolithic settlement (2500 BCE) of Durrington walls

spelling corrected

near Stonehenge, Britain was included which contained Capillarid eggs [55]. This coprolite was previously identified as human in origin using fecal lipid biomarkers. Interestingly, DNA sequenced from this coprolite contained a high proportion of reads assigned to *Canis lupus familiaris* with very few reads assigned to *Homo sapiens*, this indicates that this coprolite is likely a dog coprolite rather than a human coprolite as suggested by lipid biomarkers (see S1 Appendix). At the Bronze Age site of Must Farm in Britain (865–840 BCE), sediment samples and human and dog coprolites were included. The source of these coprolites had also been previously identified using fecal lipid biomarkers [56]. One of the coprolites previously identified as human and one of dog origin underwent shotgun sequencing and those results show that both coprolites are likely from dogs given high amounts of DNA from *Canis lupus familiaris* in those samples (see S1 Appendix). Microscopy of coprolites found eggs of *Capillaria* sp., *Echinostoma* sp., fish tapeworm (*Dibothriocephalus* sp.), giant kidney worm (*Dioctophyma renale*), and whipworm (*Trichuris* sp.). Occupation layer sediment contained all of these taxa except *Echinostoma* [56].

discussion
Amended to fit in results

wrong taxonomic writting, here, and revise all the doc

sp. added. See response to reviewers

The only Pre-Roman samples studied using ELISA were those from Must Farm as there was insufficient remaining material from the other samples. The ELISA results from these samples were indeterminate as all sample wells (8 technical replicates on two separate biological replicates for each sample) were positive for all three species (*Giardia duodenalis*, *Entamoeba histolytica*, and *Cryptosporidium*). This is an exceptionally unusual result for palaeoparasitological studies and suggests a false positive result likely from cross-reactivity with another biomolecule within the samples. Furthermore, *Cryptosporidium* has never been detected in ancient samples from Europe.

No parasite DNA was recovered from the 8 Pre-Roman samples included. Extracts were quantified using qPCR after library preparation using probes designed for the ligated sequencing adapters. The quantification of adapter-ligated molecules in these libraries (ranging from 10^6 to 10^9 copies/uL) was consistent with other samples where parasite DNA was recovered (see S1 Appendix Table D.1). Additionally, the number of filtered reads mapped to the parasite reference sequences and subsequently LCA assigned were consistent with samples from the Roman and post-Roman periods (Fig 2). Within the pre-Roman samples, 59-93% of the

LCA-assigned reads were assigned to bacteria, while less than 10% of reads were assigned to

Metazoan could be helminth parasite, explain

Further explanation provided

Fungi, Metazoa, Sar, and Viridiplantae with the exception of one sample where 15% of reads

Explain

were assigned to Fungi. Thus, the majority of LCA-assigned reads in the pre-Roman samples were from bacteria and we were unable to recover parasite DNA from taxa of which eggs were preserved.

Roman

In the 13 Roman period samples, the parasite taxa recovered by microscopy were less diverse than the pre-Roman sites. Roundworm (*Ascaris* sp.) was recovered from 11 samples studied, whipworm eggs were found in four, and both *Capillaria* and *Dicrocoelium* in one.

wrong taxonomic writing

species given

The only protozoa recovered from Roman period sites using ELISA was *Giardia duodenalis*. *Giardia* was detected in drain samples from Vagnari and the Villa of Vacone in Italy, and in a latrine from Sagalassos in Türkiye [31].

For aDNA analysis, reads assigned to roundworm (*Ascaris* sp.) were recovered from six samples. *Ascaris* sp. eggs were found by microscopy in all of these samples. *Dicrocoelium dendriticum* DNA was also recovered from the latrine at Arlon, Belgium; *Dicrocoelium* eggs were also found by microscopy from this site. No protozoa DNA was recovered.

Post-Roman

Similar to the Roman period, the most common helminth recovered from medieval period sites using microscopic analysis was roundworm (*Ascaris* sp.) which was found in four of five samples. Whipworm was found in two samples and fish tapeworm in one sample. The only protozoa recovered using ELISA was *Giardia duodenalis*. This was found in the latrine from Jerusalem [57].

Ancient DNA from roundworm (*Ascaris*) was found in three medieval samples studied. In the latrine sediment from Mértola, Portugal some of these reads were able to be assigned to the species-level as *Ascaris lumbricoides*, the human infecting species of *Ascaris*. In addition, reads assigned to whipworm (*Trichuris* sp.) were found in the latrine from Mértola in Portugal. Interestingly, no *Trichuris* eggs were seen on microscopic analysis of this latrine sediment.

How is it possible to define species level, since these spp. are not genetically defined using most of molecular markers ?

See response to reviewers for documentation of changes made

Species-level identification was also possible for whipworm reads from the latrine in Jerusalem. Reads were not only assigned to human whipworm (*Trichuris trichiura*), but also mouse whipworm (*Trichuris muris*). These results are comparable to results of a recent aDNA study employing shotgun sequencing to characterize microbial and eukaryotic DNA in a separate subsample from this same latrine in Jerusalem. In that study, *Ascaris lumbricoides* and *Trichuris trichiura* were detected [12].

discussion

This has been left here as it does not need a full description again in the discussion, but is treated the same as comparisons made to microscopy results throughout the results section. Having it here provides the easiest way for readers to contextualize the data.

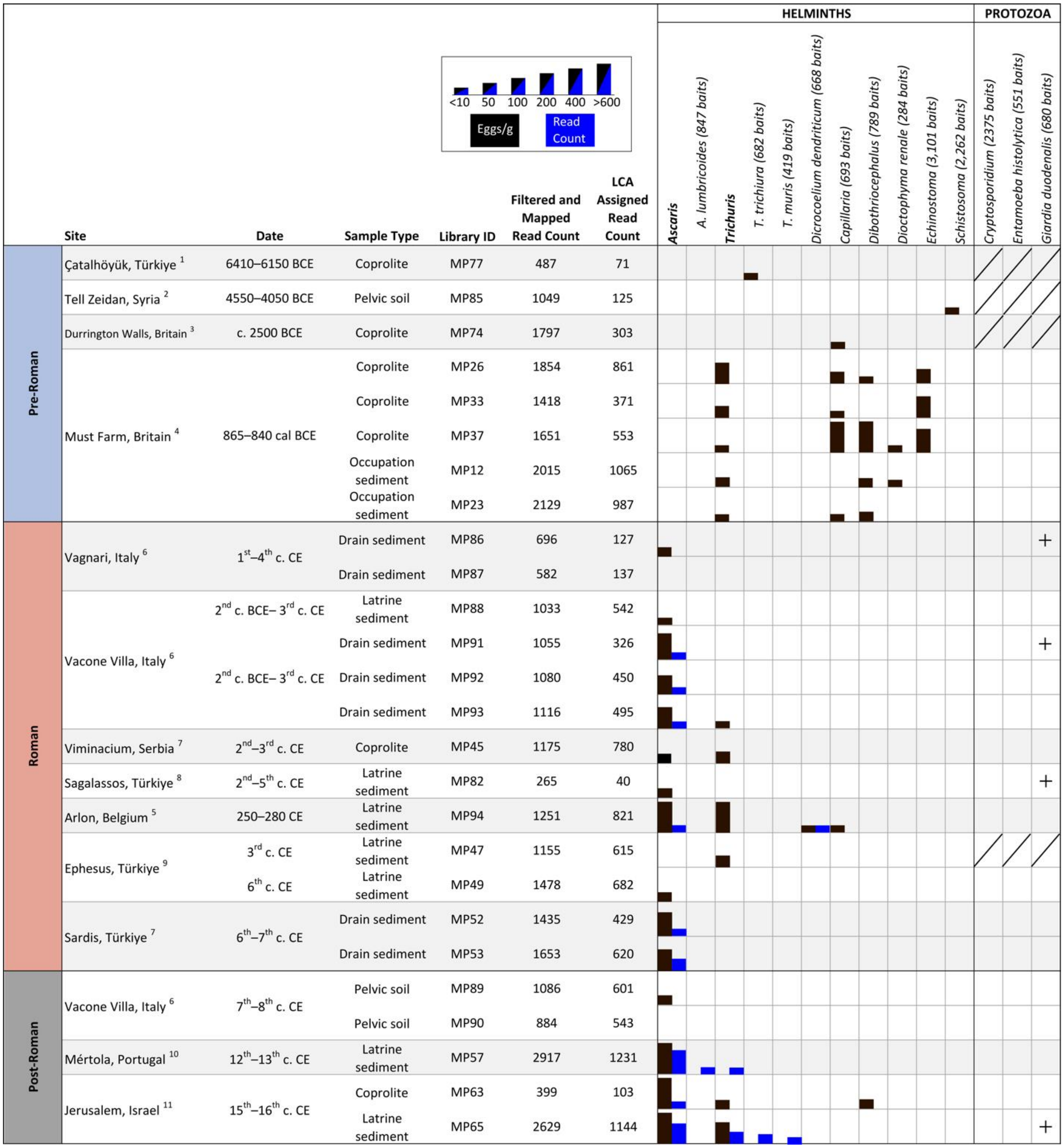

Fig 2. Summary of parasites recovered from each sample using microscopy, ELISA, and ancient DNA. Black bars represent egg concentrations identified using microscopy and blue bars represent DNA read count. Samples for which insufficient material was available for ELISA are crossed out and positive ELISA tests are marked with a +. [1] Ledger et al., 2019 [53]; [2] Anastasiou et al., 2014 [54]; [3] Mitchell et al., 2022 [55]; [4] Ledger et al., 2019 [56]; [5] Deforce et al., 2021 [58]; [6] Ledger et al., 2021 [31]; [7] Ledger et al., 2020 [59]; [8] Williams et al., 2017 [60]; [9] Ledger et al., 2018 [61]; [10] Knorr et al., 2019 [62]; [11] Yeh et al., 2015 [57].

Expansion of Results Using Ancient DNA

Shotgun sequencing on a subset of four samples was unable to recover any identifiable parasite DNA. However, using a targeted enrichment approach, parasite DNA was recovered from 9 out of 26 samples (see Fig 2 for summarized results and S3 Appendix for results by individual sample). The majority of sequenced reads assigned to human parasites were from roundworm (*Ascaris*). Additional parasite taxa from which we were able to recover DNA included the lancet liver fluke (*Dicrocoelium dendriticum*) and whipworm (*Trichuris* spp.) (Fig 2). In one of these samples (MP57 from Mértola, Portugal) whipworm eggs were not visualized by microscopy thus, aDNA analysis provides evidence for additional taxa.

The microscopic identification of *Ascaris* was replicated using aDNA analysis in six out of a total 11 Roman period samples from which *Ascaris* eggs were identified by microscopy. Three out of five medieval period samples in which *Ascaris* eggs were identified by microscopy contained *Ascaris* DNA. From both of these time periods, only three of these samples contained *Ascaris* eggs that were sufficiently preserved to still have a visible outer mamillated coat. Mamillated *Ascaris* eggs were recovered from Arlon in Belgium, Viminacium in Serbia, and the Mamluk period cesspit in Jerusalem. The mamillated coat of *Ascaris* is an important characteristic feature used for identification of the eggs, however, it can be absent in modern eggs and is often lost in archaeological contexts which makes identification challenging [63–66]. Thus, the detection of *Ascaris* DNA in samples containing decorticated and poorly preserved *Ascaris* eggs exemplifies the range of morphology that can be seen in ancient *Ascaris* eggs (Fig 5).

Figure 3 contains a comparison between the concentration of roundworm (*Ascaris*) eggs recovered by microscopy to the percent of LCA-assigned reads to Ascaridoidea or lower taxonomic rank and read count of the same. Unsurprisingly, the samples that contained the highest concentration of *Ascaris* eggs also had a higher percentage of reads assigned to *Ascaris*. For nearly all samples where *Ascaris* eggs were identified by microscopy but no DNA was recovered, the egg concentration was 30 eggs per gram or less. This suggests there may be a lower limit of detection for our sedaDNA methods which is higher than that of microscopy.

It could be interesting to discuss methodology with previous paleogenetic studies on Ascaris spp.

As we describe in the discussion we had hoped to be able to compare to other studies. However, most studies do not report egg concentrations from microscopy thus precluding us from determining if this trend is comparable to other studies

Site	Sample Type	DNA Library ID	*Ascaris* (eggs per gram)	% LCA Assigned (Ascaridoidea or Lower)	Read Count (Ascaridoidea or Lower)
Jerusalem	Cesspit sediment	MP65	1580	21.8%	249
Mértola	Latrine	MP57	1110	24.1%	297
Arlon	Latrine	MP94	990	0.5%	4
Jerusalem	Coprolite	MP63	595	5.8%	6
Vacone	Drain sediment	MP91	405	1.8%	6
Sardis	Drain sediment	MP52	305	1.2%	5
Sardis	Drain sediment	MP53	205	9.7%	60
Vacone	Drain sediment	MP93	185	1.4%	7
Vacone	Drain sediment	MP92	140	1.6%	7
Vacone	Pelvic soil	MP89	30	0.0%	0
Vagnari	Drain sediment	MP86	25	2.4%	3
Ephesus	Latrine	MP49	25	0.0%	0
Sagalassos	Latrine	MP82	20	0.0%	0
Vacone	Latrine	MP88	5	0.0%	0

Fig 3. Heatmap comparing concentration of *Ascaris* eggs recovered from each sample to percentage of LCA-assigned reads. *Ascaris* egg concentration is reported in eggs per gram based on microscopy and LCA-assigned reads include reads that were assigned to the Superfamily Ascaridoidea or lower and read count assigned to Ascaridoidea or lower. Samples are ordered in the table based on egg per gram concentration from highest to lowest concentration. colors? Description added

The sequence reads assigned to roundworm were authenticated as aDNA using *mapDamage* [67]. They showed typical deamination patterns for aDNA and fragment length distributions (Fig 4). Statistical analysis could not be done on reads from other taxa due to the low number of reads.

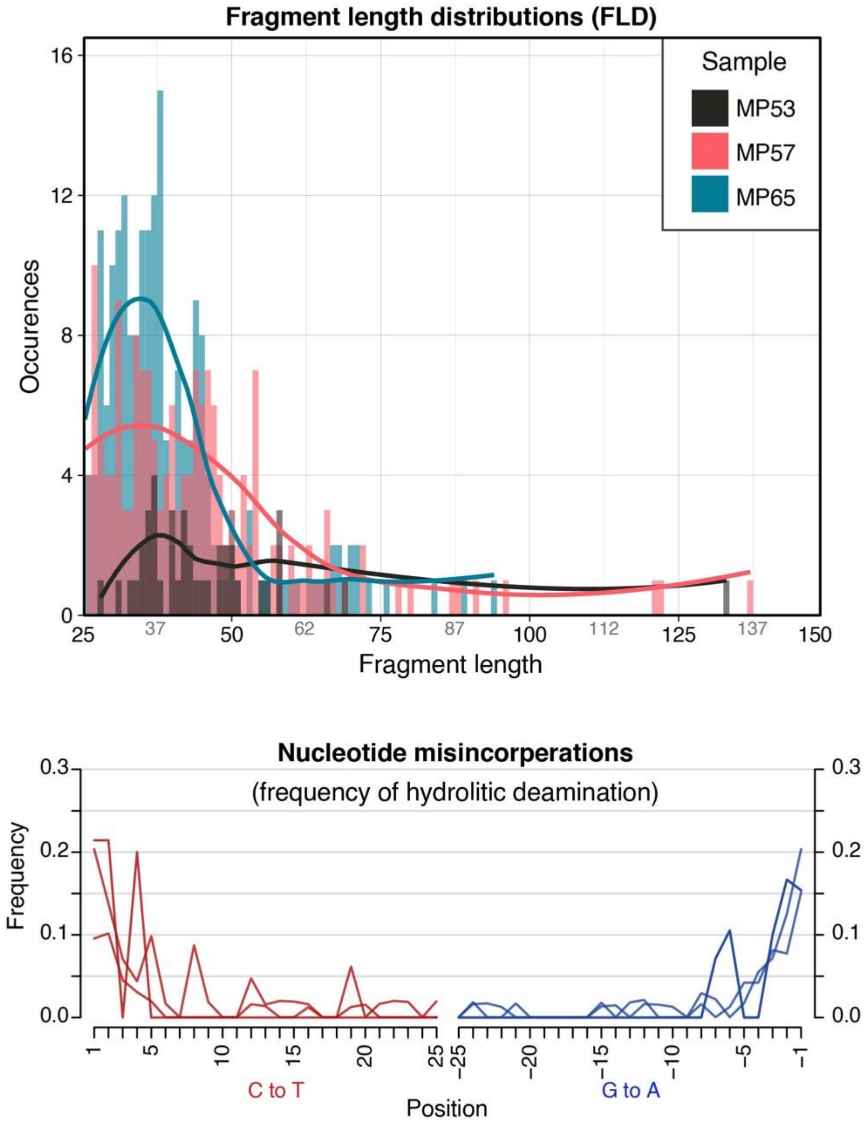

Fig 4. Deamination and fragment length distributions for *Ascaris* reads. Data generated using *mapDamage* showing C to T and G to A deamination patterns (top) and fragment length profile (bottom) for reads assigned to *Ascaris* in samples with highest read count.

Discussion

A Multimethod Approach in Paleoparasitology

Each individual method used in our study contributed complementary results to the reconstruction of parasite diversity in the past. For samples from the Neolithic and Bronze Age (where the number of surviving parasite eggs was generally low), microscopy was more

sensitive for detecting ancient parasites than was aDNA. As can be seen in Figure 2, six different taxa were recovered using microscopy and this included rare parasites in archaeological settings such as *Schistosoma, Echinostoma*, and *Dioctophyma renale*. No parasite DNA sequences were recovered in these samples using our parasite sedaDNA methods, despite all of the taxa being present in our bait set (see S2 Appendix for full list of baits); with 2,262 baits targeting *Schistosoma*, 3,101 baits for *Echinostoma*, and 284 baits for *Dioctophyma renale*. The majority of DNA recovered from these was bacterial in origin. The parasite diversity in these samples was more diverse than that seen in the Roman and medieval periods, and is further characterized by a notable absence of *Ascaris* which is the most common parasite found in later time periods. This raises the possibility that our targeted capture approach was not as efficient at detecting these parasite species as it was for *Ascaris*. Aside from parasite DNA recovery, our sedaDNA methods provided additional data for the identification of the source of coprolites studied. High read counts assigned to dog (*Canis lupus familiaris*) in the Durrington Walls and Must Farm coprolites suggests that these coprolites may in fact be from dogs not humans as previously thought. The accurate identification of coprolites is important for interpretation of parasite results.

spp. added

Roundworm (*Ascaris*) and whipworm (*Trichuris*) were the most commonly identified parasites by both microscopy and aDNA in the Roman and medieval period samples. In all Roman period samples in which parasite DNA was recovered, eggs from the assigned taxa were identified by microscopy. No additional parasites were detected using ancient DNA analysis for our Roman period sites, and aside from reads assigned to the lancet liver fluke (*Dicrocoelium dendriticum*) in the latrine from Arlon, Belgium, all other reads were from *Ascaris* and could not be assigned to a lower rank than the genus-level. This was not the case for the medieval period samples. In medieval period samples, *Ascaris* reads could be assigned to the species-level in one case, and additional taxa were recovered that were not identified using microscopy. The only time period where whipworm (*Trichuris*) DNA was recovered was from our medieval period samples, despite *Trichuris* being identified by microscopy in Roman period samples.

Combining aDNA with microscopy in paleoparasitology provides the potential to identify ancient parasites to the species-level [15,20,22]. For many parasite taxa, egg morphology

cannot be used to accurately identify eggs to the species-level [20,68,69]. We were able to do this in few cases using our targeted enrichment approach. In latrine sediment from the medieval site of Mértola, *Ascaris* reads were assigned to the species-level as *Ascaris lumbricoides*. *Ascaris lumbricoides* is classically the human infecting species of *Ascaris* which fits with identification of this species in a latrine that would contain primarily human fecal waste. However, it is not surprising that for all other samples, reads could only be assigned to the genus-level as there is minimal genetic variation between *Ascaris lumbricoides* and *Ascaris suum*, which is the species that classically is considered to infect pigs [70,71]. Additionally, two species of *Trichuris* were identified in the latrine sediment from 15th–16th c. CE Jerusalem. Both the human-infecting species, *Trichuris trichiura*, and the mouse-infecting species, *Trichuris muris*, were identified. The presence of *T. muris* in the latrine could be a result of mice having access to the latrine or indicate that material contaminated with feces from rodents was deposited in the latrine.

One important contribution of our study is that aDNA analysis was able to replicate identification of *Ascaris* eggs that had atypical morphology as a result of degradation and environmental conditions after egg deposition. The outer mamillated coat of *Ascaris* eggs is a key morphological feature used in identification of the eggs. The *Ascaris* eggs identified by microscopy in samples from this study were all decorticated except for those from Arlon, Belgium (Fig 5). Thus, if one is only identifying eggs with preserved mamillated coats, they will grossly underestimate the prevalence of *Ascaris* in the past. Even in modern studies, decorticated roundworm eggs can be easily misidentified [66]. Thus, the addition of aDNA analysis can confirm identification in cases of uncertainty due to taphonomic changes and poor egg preservation and allows us to more confidently explore the appearance of ancient eggs.

Explain. How does target enrichment make possible the definition at sp. level?

Done

? Asked above. Problem. Specially working with latrine samples

See response to reviewers

what variation? which genetic region?

We have expanded this disucssion to discuss genetic similarity between the two species and the current debates on species distinction

This statment is controversial, since the Ascaris sp. results are not from aDNA extracted directly from these isolated eggs with atypical morphology.

See response to reviewers for documentation of how this has been changed to address the reviewers concerns.

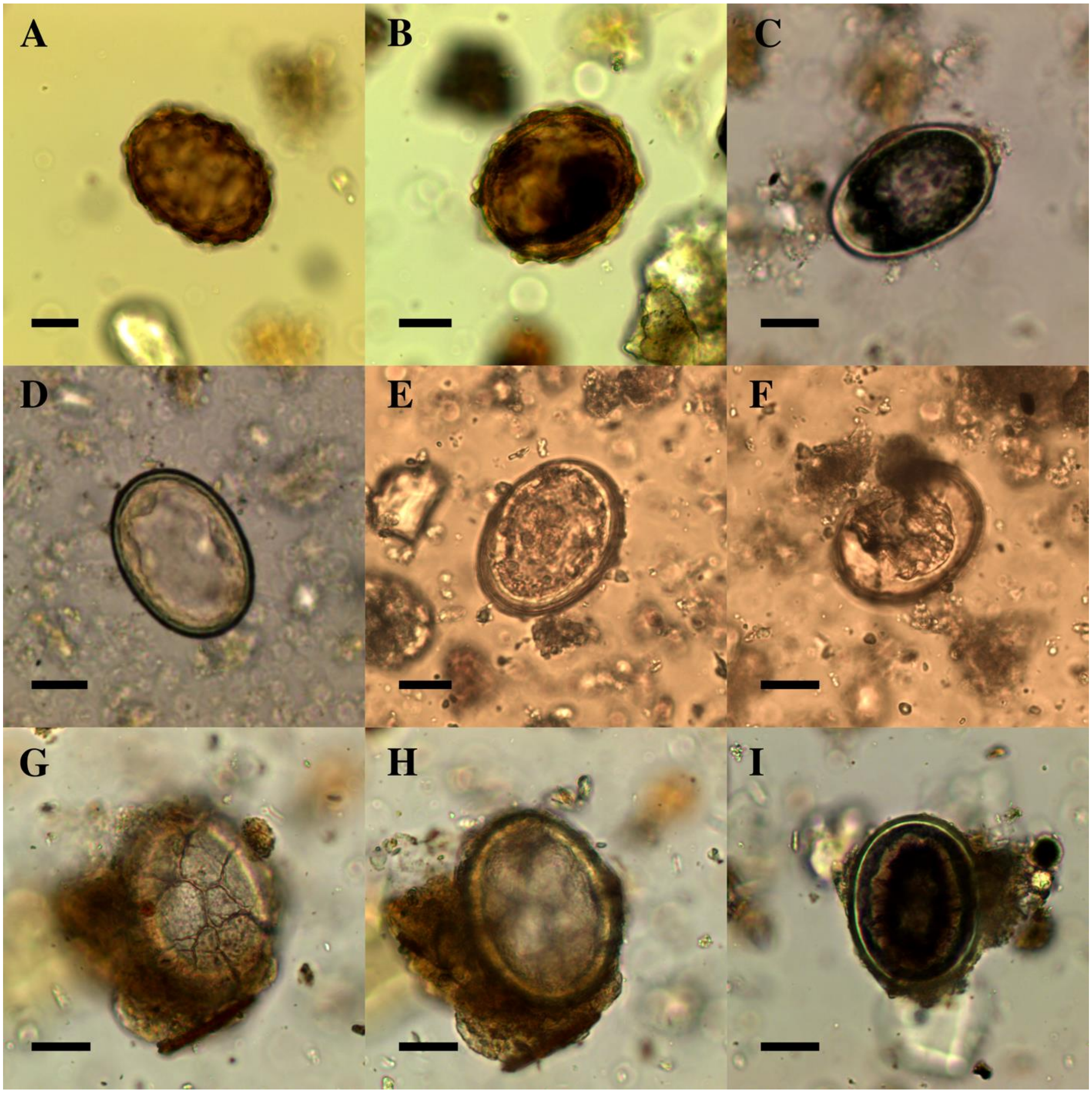

Fig 5. Variation in morphological appearance of *Ascaris* eggs identified in samples with confirmed presence of *Ascaris* using aDNA methods. (A) fertilized egg from Arlon with mamillated coat; (B) fertilized egg from Arlon with mamillated coat; (C) fertilized decorticated egg from Mértola (image credit: Delaney Knorr); (D) fertilized decorticated egg from Mértola (image credit: Delaney Knorr); (E) fertilized decorticated egg from Vacone; (F) fertilized decorticated egg from Vacone; (G and H) fertilized decorticated egg from Sardis with surface cracking; (I) fertilized decorticated egg from Sardis. Bar=? Added

The bait set designed for targeted enrichment in our study covered 121 taxa. Most studies in paleoparasitology relying on PCR methods targeted a maximum of 5 taxa or even a single taxon previously identified by microscopy [13,24,27,28,36,49]. Targeted enrichment has only previously been used in parasite aDNA research for detecting malaria (*Plasmodium* sp.) in skeletal remains [19]. On the other hand, while we are limited by coverage of the bait set and references sequences available for robust bait design, targeted enrichment allowed for recovery of parasite DNA when shotgun sequencing to an average depth of 3.3 million reads (total read counts from shotgun sequencing ranged from 2.2–4.0 million reads) was unsuccessful in recovering any parasite DNA. In published palaeoparasitological studies relying on shotgun sequencing, most samples were sequenced to a depth of at least 5 million reads with some samples being sequenced to a depth of 60 million reads [12,15,22]. The cost of sequencing to this level is not financially feasible for many studies and most aDNA and paleoparasitology labs. Additionally, in most cases less than 5% of these sequenced reads are assigned to target organisms [72]. In our samples even with targeted enrichment, <0.001% of reads were assigned to parasites. Due to the very small proportion of parasite DNA in our sediment samples (which is otherwise dominated by bacteria and currently unidentifiable DNA, along with plants and fungi), very deep shotgun sequencing would be needed to find parasite DNA in environmental samples. Paleogenetic studies in parasitology are likely to continue to lag behind that seen for other pathogens without changes that make the use of aDNA more cost effective. Microscopy is a relatively cheap and reliable method for egg identification from archaeological sediments making it a preferred method for many researchers.

While ancient DNA analysis is a promising addition to the methodological armamentarium in paleoparasitology, our results suggest that currently microscopy remains the most cost-effective and reliable screening tool in paleoparasitology. When resources allow, ELISA and aDNA can be utilized alongside microscopy to analyze a sample more comprehensively. In palaeoecological studies as well, sedaDNA has been found to function best as a complementary proxy, as other methods such as palynology and macrofossil identification tend to find similar, but non-overlapping taxa [73–75]. Microscopy continues to successfully recover parasites eggs in samples with low egg concentration. Based on our results comparing

eggs per gram concentration generated from microscopic analysis to percent of LCA-assigned reads, there appears to be a minimum concentration of eggs needed for increased chance of DNA recovery using the currently described methods. Samples that contained 30 eggs per gram or lower of *Ascaris* eggs did not contain any detectable *Ascaris* DNA. It is difficult to directly compare this to other studies as many do not report eggs per gram or had higher egg concentrations in all samples studied [13]. However, both in our study and other palaeoparasitological studies, there are cases where parasite DNA is detected in the absence of any preserved helminth eggs [15,24,36]. In these cases it is possible that taphonomic changes to the eggs have resulted in inability to identify them by microscopy but that their DNA can still be captured and sequenced. Alternatively, that DNA from adult worms themselves rather than eggs is being detected. It is also likely that eggs are not evenly distributed within a sample and studying different subsamples with microscopy and aDNA will provide different findings.

The complexities of parasite egg preservation and in turn parasite DNA preservation is poorly understood. This diverse group of organisms has variable eggshell structures, rates of embryological development, environmental niches, and are recovered from different contexts including in the gastrointestinal space as in the case of pelvic soil as well as cesspit, latrine, and drain contexts. We were able to detect parasite DNA in various sample types including pelvic soil, coprolites, and latrine or cesspit samples. We had the most success with identifying *Ascaris* using our sedaDNA methods and targeted enrichment. The ability to more easily recover *Ascaris* DNA from all samples may be related to both methodological factors as well as parasite biology. As part of its life cycle, *Ascaris* develops in soil, thus by necessity it is resistant to harsh environmental conditions. In addition, extensive reference sequences for *Ascaris* allowed us to create a robust set of baits for *Ascaris*. A total of 1,769 of 30,240 baits with average tiling density of 7x were for either *Ascaris lumbricoides* or *Ascaris suum* (see S2 Appendix for list of baits). In comparison, our bait set had very good coverage of taxa identified by microscopy for which we were not able to recover DNA. *Echinostoma* had a total of 3,213 baits (average tiling density ranged for each species from 8.4–21.8x), *Schistosoma* spp. had 2,262 baits (average tiling density ranged for each species from 8.1–21.8x), and *Dibothriocephalus* (syn.

Trichuris spp. as well

This is true, though in combination with further points made in the discussion this is our reasoning for improved detection of Ascaris, we agree that this cannot be the sole reason we would detect Ascaris rather than Trichuris

Diphyllobothrium) had 1,980 baits (average tiling density ranged for each species from 8.8–23.4x).

Parasite biology will also influence DNA recovery [24]. The size of adult worms from different helminths is variable. For example, *Ascaris lumbricoides* can grow up to 30 cm long whereas *Trichuris trichiura* is typically only 5 cm long and *Dibothriocephalus latus* can grow to 1000 cm [68]. Thus, the biomass of the adult worms would be expected to vary by taxa. This would impact the amount of DNA expected from each species in pelvic soil samples, as pelvic soil samples would contain decomposed intestinal contents including helminths causing infection at the time of death. The number of eggs excreted per day is also quite variable, with *Ascaris lumbricoides* producing around 200,000 eggs per day [76] and *Trichuris trichiura* only 18,000 eggs per day [77]. These factors may be compounded by sedaDNA preservation characteristics, which are thought to necessitate binding to sedimentary minerals [78–81]. If parasite DNA does not come in contact with sedimentary minerals before being broken down by microbes in the local microbiome, those parasite DNA fragments are unlikely to preserve and be recoverable.

But only should be considered when working with abdominal region samples

We agree and have specified in the following sentences we are discussing this concept using pelvic soil (abdominal samples).

While sedaDNA with targeted enrichment has the potential to significantly aid in palaeoparasitological research, further methodological improvements are needed to reach the taxonomic characterization already viable with the gold standard method within the field—microscopy. Furthermore, *Giardia duodenalis* antigen was detected using ELISA from four sites, and although our bait set did include protozoa, we were not able to recover any DNA sequences from *Giardia*. Thus, ELISA continues to have a unique value in that it is a useful additional method for maximizing recovery of common intestinal protozoa, which are rarely preserved well enough to be visualized using microscopy and have been detected in very few aDNA studies thus far [41]. The indeterminate results from Must Farm highlight an important limitation of ELISA which is the potential for cross-reactivity with unknown antigens that may be found in soil. Further, optimization of aDNA methods for detection of protozoa would be valuable to confirm results with a second method.

Temporal Changes in Parasites in the Roman Period

Using a multimethod approach, we aimed to produce a more accurate reconstruction of parasite infection in the Roman period. Inclusion of pre-Roman and post-Roman samples from areas that were at one time part of the Roman Empire provides important comparative data to understand how parasite diversity may have changed during the Roman period. The samples included in our study contribute a small subset of results for parasite presence in areas that were once part of the Roman Empire from the Neolithic through the medieval period. The parasite taxa recovered from the pre-Roman sites were much more diverse than any other time period. At these sites we recovered various zoonotic parasites including *Echinostoma* and fish tapeworm (*Dibothriocephalus* sp.) which are spread by ingestion of raw or undercooked fish, frogs, or mollusks, as well as *Capillaria* from two sites. This is in conjunction with one common soil-transmitted helminth, whipworm (*Trichuris trichiura*), which is spread by the fecal-oral route.

By comparison in the Roman period, we observe almost exclusively soil-transmitted helminths, roundworm (*Ascaris* sp.) and whipworm (*Trichuris trichiura*). One exception is the site of Arlon, Belgium where *Capillaria* and the lancet liver fluke (*Dicrocoelium dendriticum*) were also recovered. Similarly, in medieval period sites, soil-transmitted helminths are almost exclusively present, aside from a coprolite from Jerusalem where fish tapeworm (*Dibothriocephalus* sp.) was also found. idem

type of transmission of these parasites?

Specified

When these results are included within the published literature on parasite presence in these time periods, our results are comparable to patterns seen using a much larger dataset (see Tables F.1–F.3 in S1 Appendix for a review of published studies). The proportion of sites with whipworm is consistently high in all time periods, while roundworm appears to increase in frequency in the Roman period and remains stable at that level in the medieval period (Fig 6). Roundworm was recovered from 35% of pre-Roman sites, which increased to 67% of Roman and 79% of medieval period sites. The increase in proportion of sites where roundworm was found in the Roman and medieval periods may reflect decreased sanitation and hygiene. Numerous other factors such as increased urbanization, cultural preferences for the use of chamber pots, and agricultural practices involving the use of human feces as fertilizer may have all contributed to this increase in fecal-oral parasite presence [31,82].

Only roundworms?

Yes, as can be seen in Fig 6, there is a clear increase in Ascaris (roundworm), while there is an increase in whipworm in medieval period there is not a clear increase from the pre-roman to roman period. We have added this nuance

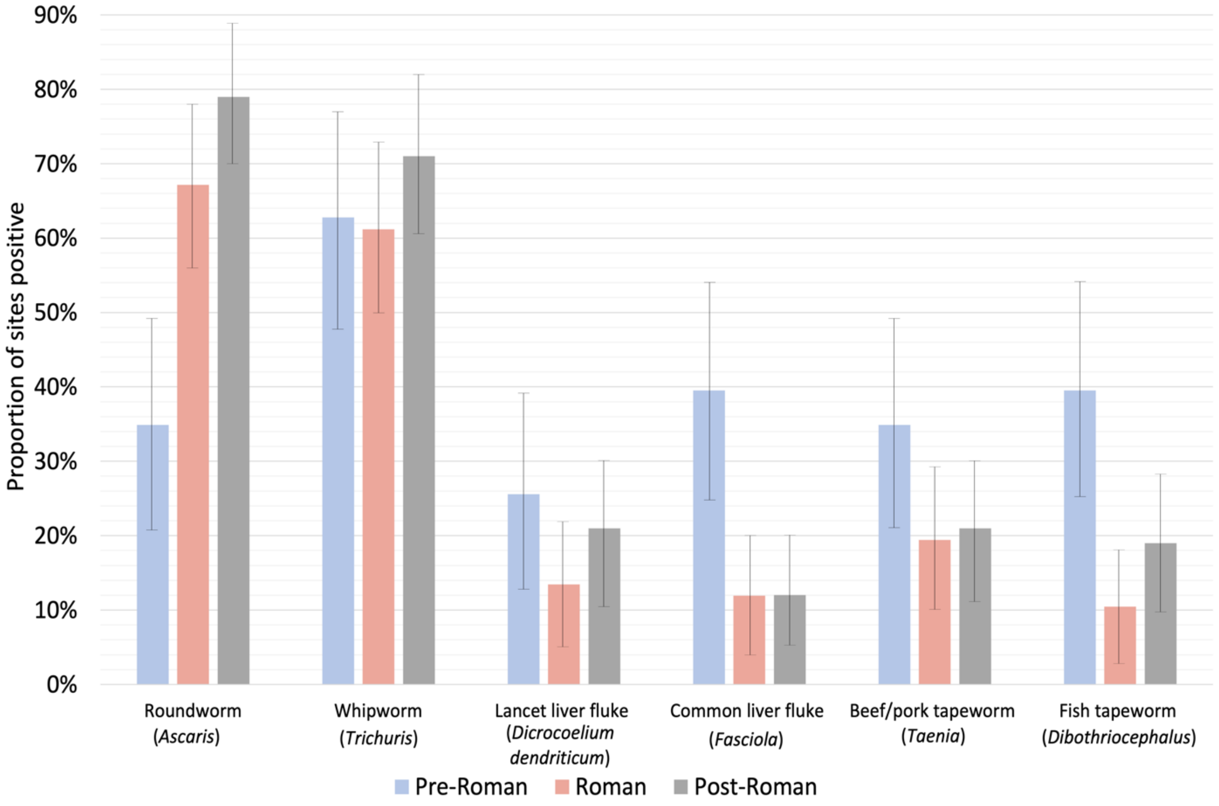

Fig 6. Temporal trends in parasite diversity before, during, and after the Roman period for the most common helminths recovered from archaeological samples. For each parasite, bars indicate the proportion of studied sites where each parasite was recovered for pre-Roman, Roman, and post-Roman sites with 95% confidence intervals. See S1 Appendix for individual studies used to generate temporal trends.

Zoonotic and foodborne parasites are different concepts. Foodborne parasites are not only related to consuming meat

Amended as suggested

Zoonotic and foodborne parasites (parasites spread from animals to humans directly or through ingestion of meat) [83], including fish tapeworm (*Dibothriocephalus* sp.), liver flukes (*Dicrocoelium dendriticum* and *Fasciola*) and beef/pork tapeworm (*Taenia* sp.) are generally more common in pre-Roman time periods compared to the later Roman and medieval periods (Fig 6). This may reflect changes to human-animal interactions and dietary preferences. It is possible that the decline in lancet liver fluke during the Roman period is an anomaly in our data as the proportion of sites where it was found in the medieval period is quite consistent with the pre-Roman period. However, it does appear that *Fasciola* liver fluke was more common in the

pre-Roman period compared to later time periods. It was found at 40% of pre-Roman sites compared to 12% of Roman and 12% of medieval sites. Both types of liver flukes in fecal samples can reflect true infection or false parasitism, usually from consumption of animal liver containing eggs [84]. In all time periods, nearly all sites where *Fasciola* was found are North of the Alps.

A similar pattern as seen for liver flukes is seen for fish tapeworm (*Dibothriocephalus* sp.). Fish tapeworm was most common in pre-Roman sites, being found at 40% of sites and then decreases to 10% in the Roman period and 19% in the medieval period. Fish tapeworm is acquired from ingestion of raw or undercooked freshwater fish in Europe. The decline in the Roman period may represent a preference for cooked fish or, alternatively, a decreased reliance on freshwater fish but rather marine fish.

There are many intricacies of transmission and determinants of infection that can be explored for each individual parasite. However, what is most striking overall is the high proportion of sites in all time periods in Europe where we find evidence for enteric parasites. Roundworm is present at 67% of Roman sites studied and whipworm is found at 61% of sites. Of course, a major limitation of this type of analysis is publication bias. However, there is no doubt that these two parasites contributed to the burden of disease in most Roman communities. Many infections with these two species can be asymptomatic; however, they can also cause gastrointestinal upset and for children result in nutrient deficiencies, stunting of growth, and intellectual disability [85].

Conclusions

The vast majority of work done in paleoparasitology or archeoparasitology has relied on microscopic analysis of sediments and palaeofaeces to identify parasites. ELISA has also been used by some groups to detect ancient protozoa. We have shown that sedaDNA methods combined with targeted enrichment can be used to detect parasite DNA in as little as 0.25 g of paleofecal sediment. A multimethod approach in paleoparasitology allows for the most complete reconstruction of past parasite diversity currently available to us. However, microscopy continues to be the most sensitive method for detecting helminths in paleofecal

samples and appears to detect helminth eggs present in lower concentrations than our sedaDNA methods are able to detect. ELISA is uniquely able to detect protozoa such as *Giardia* in our samples. In our set of samples, aDNA was able to confirm identification of *Ascaris* eggs with atypical morphology, provide species-level identification for some parasites, and detect additional parasites not seen by microscopy and ELISA. As these methods are refined and applied to additional samples, they may help facilitate a more holistic analysis of parasite phylogeography and parasite biology through time.

The two statements are not conclusive. One is just a suggestion. The sp. level identification needs explanation.

We have amended the wording to more accurately state that we have replicated identification of Ascaris in these samples using another method but removed the wording 'confirmed' as previously used.

Applying this multimethod approach to 26 coprolite, latrine, and sewer samples from the Neolithic through medieval period allowed us to characterize temporal trends in parasite infection around the rise, expansion and eventual decline of the Roman Empire. We found a trend of decreased zoonotic and foodborne parasites from the pre-Roman to Roman and medieval periods and concurrent increase in parasites spread by the fecal-oral route, especially roundworm.

Acknowledgments

We would like to thank local archaeological institutions, and excavation teams from the sites included in this study, and the official parties granting the required permissions. We thank Christa Clamer for invaluable work providing material and contextual interpretation of our results. We thank Techlab©, Blacksburg, USA for their kind donation of ELISA kits used in this research. Thanks to Brian Golding for providing access to his computational resources, and to Ana Duggan for assistance and guidance on bioinformatic workflow. Thanks to all members and affiliates of the McMaster Ancient DNA Centre for their ongoing support of this work. Numerous individuals have contributed to various stages of this work; from the site of Sagalassos we would like to thank the Research Foundation Flanders and the Research Fund of KU Leuven for supporting the research for this paper. From the Ephesus site we would like to thank the late excavation director Sabine Ladstätter, who made it possible for us to collect samples for analysis. From Vacone we would like to thank Devin Ward, Gary Farney, Dylan Bloy, and the Soprintendenza Archeologia, Belle arti e Paesaggio per l'area metropolitana di Roma e la provincia di Rieti.

Funding Sources

This work was supported by The Social Sciences and Humanities Research Council of Canada [752-2016-2085], a Tidmarsh Cambridge Scholarship from the Cambridge Commonwealth, European and International Trust and Trinity Hall College, and the Society for the Promotion of Roman Studies to MLL.

TJM and HNP wish to thank the CANA Foundation for their financial support alongside the Garfield Weston Foundation, the Social Sciences and Humanities Research Council of Canada, CIFAR, and Natural Sciences and Engineering Research Council of Canada for funding this research.

References

1. Bos KI, Kühnert D, Herbig A, Esquivel-Gomez LR, Valtueña AA, Barquera R, et al. Paleomicrobiology: Diagnosis and evolution of ancient pathogens. Annu Rev Microbiol. 2019. doi:10.1146/annurev-micro-090817-062436

2. Arriola LA, Cooper A, Weyrich LS. Palaeomicrobiology: Application of ancient DNA sequencing to better understand bacterial genome evolution and adaptation. Frontiers in Ecology and Evolution. 2020;8. doi:10.3389/fevo.2020.00040

3. Duchêne S, Ho SYW, Carmichael AG, Holmes EC, Poinar H. The recovery, interpretation and use of ancient pathogen genomes. Curr Biol. 2020;30: R1215–R1231. doi:10.1016/j.cub.2020.08.081

4. Lewis CM Jr, Akinyi MY, DeWitte SN, Stone AC. Ancient pathogens provide a window into health and well-being. Proc Natl Acad Sci U S A. 2023;120: e2209476119. doi:10.1073/pnas.2209476119

5. Wagner DM, Klunk J, Harbeck M, Devault A, Waglechner N, Sahl JW, et al. *Yersinia pestis* and the Plague of Justinian 541–543 AD: a genomic analysis. Lancet Infect Dis. 2014;14: 319–326. doi:10.1016/S1473-3099(13)70323-2

6. Key FM, Posth C, Esquivel-Gomez LR, Hübler R, Spyrou MA, Neumann GU, et al. Emergence of human-adapted *Salmonella enterica* is linked to the Neolithization process. Nat Ecol Evol. 2020;4: 324–333. doi:10.1038/s41559-020-1106-9

7. Mühlemann B, Jones TC, Damgaard P de B, Allentoft ME, Shevnina I, Logvin A, et al. Ancient hepatitis B viruses from the Bronze Age to the Medieval period. Nature. 2018;557: 418–423. doi:10.1038/s41586-018-0097-z

8. Rascovan N, Sjögren K-G, Kristiansen K, Nielsen R, Willerslev E, Desnues C, et al. Emergence and spread of basal lineages of *Yersinia pestis* during the Neolithic decline. Cell. 2019;176: 295-305.e10. doi:10.1016/j.cell.2018.11.005

9. Spyrou MA, Bos KI, Herbig A, Krause J. Ancient pathogen genomics as an emerging tool for infectious disease research. Nat Rev Genet. 2019;20: 323–340. doi:10.1038/s41576-019-0119-1

10. Eaton K, Featherstone L, Duchene S, Carmichael AG, Varlık N, Golding GB, et al. Plagued by a cryptic clock: insight and issues from the global phylogeny of *Yersinia pestis*. Commun Biol. 2023;6: 23. doi:10.1038/s42003-022-04394-6

11. Hagan RW, Hofman CA, Hübner A, Reinhard K, Schnorr S, Lewis CM Jr, et al. Comparison of extraction methods for recovering ancient microbial DNA from paleofeces. Am J Phys Anthropol. 2020;171: 275–284. doi:10.1002/ajpa.23978

12. Sabin S, Yeh H-Y, Pluskowski A, Clamer C, Mitchell PD, Bos KI. Estimating molecular preservation of the intestinal microbiome via metagenomic analyses of latrine sediments from two medieval cities. Philos Trans R Soc Lond B Biol Sci. 2020;375: 20190576. doi:10.1098/rstb.2019.0576

13. Flammer PG, Dellicour S, Preston SG, Rieger D, Warren S, Tan CKW, et al. Molecular archaeoparasitology identifies cultural changes in the Medieval Hanseatic trading centre of Lübeck. Proc R Soc B. 2018;285: 20180991. doi:10.1098/rspb.2018.0991

14. Flammer PG, Ryan H, Preston SG, Warren S, Přichystalová R, Weiss R, et al. Epidemiological insights from a large-scale investigation of intestinal helminths in Medieval Europe. PLoS Negl Trop Dis. 2020;14: e0008600. doi:10.1371/journal.pntd.0008600

15. Søe MJ, Nejsum P, Seersholm FV, Fredensborg BL, Habraken R, Haase K, et al. Ancient DNA from latrines in Northern Europe and the Middle East (500 BC-1700 AD) reveals past parasites and diet. PLOS One. 2018;13: e0195481. doi:10.1371/journal.pone.01954

16. Appelt S, Fancello L, Le Bailly M, Raoult D, Drancourt M, Desnues C. Viruses in a 14th-century coprolite. Appl Environ Microbiol. 2014;80: 2648–2655. doi:10.1128/AEM.03242-13

17. Ng TFF, Chen L-F, Zhou Y, Shapiro B, Stiller M, Heintzman PD, et al. Preservation of viral genomes in 700-y-old caribou feces from a subarctic ice patch. Proc Natl Acad Sci U S A. 2014;111: 16842–16847. doi:10.1073/pnas.1410429111

18. Ferreira LF. An introduction to paleoparasitology. In: Reinhard KJ, Araújo A, editors. Foundations of Paleoparasitology. Rio de Janeiro: Editoria Fiocruz; 2014. pp. 27–42.

19. Marciniak S, Prowse TL, Herring DA, Klunk J, Kuch M, Duggan AT, et al. *Plasmodium falciparum* malaria in 1st–2nd century CE southern Italy. Curr Biol. 2016;26: R1220–R1222. doi:10.1016/j.cub.2016.10.016

20. Côté NM-L, Le Bailly M. Palaeoparasitology and palaeogenetics: Review and perspectives for the study of ancient human parasites. Parasitology. 2018;145: 656–664. doi:10.1017/S003118201700141X

21. Selbach C, Jorge F, Dowle E, Bennett J, Chai X, Doherty J-F, et al. Parasitological research in the molecular age. Parasitology. 2019;146: 1361–1370. doi:10.1017/S0031182019000726

22. Tams KW, Jensen Søe M, Merkyte I, Valeur Seersholm F, Henriksen PS, Klingenberg S, et al. Parasitic infections and resource economy of Danish Iron Age settlement through ancient DNA sequencing. PLoS One. 2018;13: e0197399. doi:10.1371/journal.pone.0197399

23. Chessa D, Murgia M, Sias E, Deligios M, Mazzarello V, Fiamma M, et al. Metagenomics and microscope revealed *T. trichiura* and other intestinal parasites in a cesspit of an Italian nineteenth century aristocratic palace. Sci Rep. 2020;10: 12656. doi:10.1038/s41598-020-69497-8

24. Roche K, Capelli N, Pacciani E, Lelli P, Pallecchi P, Bianucci R, et al. Gastrointestinal parasite burden in 4th-5th c. CE Florence highlighted by microscopy and paleogenetics. Infect Genet Evol. 2021;90: 104713. doi:10.1016/j.meegid.2021.104713

25. Askari Z, Ruehli F, Bouwman A, Shariati V, Naddaf SR, Otranto D, et al. Genomic palaeoparasitology traced the occurrence of *Taenia asiatica* in ancient Iran (Sassanid Empire, 2th cent. CE-6th cent. CE). Sci Rep. 2022;12: 12045. doi:10.1038/s41598-022-10690-2

26. Doyle SR, Søe MJ, Nejsum P, Betson M, Cooper PJ, Peng L, et al. Population genomics of ancient and modern *Trichuris trichiura*. Nature Communications. 2022;13: 3888. Available: https://www.nature.com/articles/s41467-022-31487-x

27. Iñiguez AM, Brito L, Guedes L, Chaves SA de M. Helminth infection and human mobility in sambaquis: Paleoparasitological, paleogenetic, and microremains investigations in Jabuticabeira II, Brazil (2890 ± 55 to 1805 ± 65 BP). Holocene. 2022;32: 200–207. doi:10.1177/09596836211060490

28. Oh CS, Seo M, Lee HJ, Kim MJ, Lim D-S, Shin DH. Genetic analysis of ancient *Clonorchis sinensis* eggs attained from Goryeong Mummy of Joseon Dynasty Period. J Parasitol. 2022;108: 70–78. doi:10.1645/21-49

29. Murchie TJ, Kuch M, Duggan AT, Ledger ML, Roche K, Klunk J, et al. Optimizing extraction and targeted capture of ancient environmental DNA for reconstructing past environments

using the PalaeoChip Arctic-1.0 bait-set. Quat Res. 2021;99: 305–328. doi:10.1017/qua.2020.59

30. Anastasiou E, Mitchell PD. Simplifying the process of extracting intestinal parasite eggs from archaeological sediment samples: A comparative study of the efficacy of widely-used disaggregation techniques. International Journal of Paleopathology. 2013;3: 204–207. doi:10.1016/j.ijpp.2013.04.004

31. Ledger ML, Micarelli I, Ward D, Prowse TL, Carroll M, Killgrove K, et al. Gastrointestinal infection in Italy during the Roman Imperial and Longobard periods: A paleoparasitological analysis of sediment from skeletal remains and sewer drains. International Journal of Paleopathology. 2021;33: 61–71. doi:10.1016/j.ijpp.2021.03.001

32. Gonçalves MLC, da Silva VL, de Andrade CM, Reinhard K, da Rocha GC, Le Bailly M, et al. Amoebiasis distribution in the past: first steps using an immunoassay technique. Trans R Soc Trop Med Hyg. 2004;98: 88–91. doi:10.1016/S0035-9203(03)00011-7

33. Le Bailly M, Bouchet F. Paléoparasitologie et immunologie. L'exemple d'*Entamoeba histolytica*. ArcheoSciences. 2006;30: 129–135. doi:10.4000/archeosciences.281

34. Le Bailly M, Bouchet F. A first attempt to retrace the history of dysentery caused by *Entamoeba histolytica*. In: Mitchell PD, editor. Sanitation, latrines and intestinal parasites in past populations. Farnham: Ashgate; 2015. pp. 219–228.

35. Mitchell PD, Stern E, Tepper Y. Dysentery in the crusader kingdom of Jerusalem: an ELISA analysis of two medieval latrines in the City of Acre (Israel). J Archaeol Sci. 2008;35: 1849–1853. doi:10.1016/j.jas.2007.11.017

36. Søe MJ, Nejsum P, Fredensborg BL, Kapel CMO. DNA typing of ancient parasite eggs from environmental samples identifies human and animal worm infections in Viking-age settlement. J Parasitol. 2015;101: 57–63. doi:10.1645/14-650.1

37. Kaisar MMM, Brienen EAT, Djuardi Y, Sartono E, Yazdanbakhsh M, Verweij JJ, et al. Improved diagnosis of *Trichuris trichiura* by using a bead-beating procedure on ethanol preserved stool samples prior to DNA isolation and the performance of multiplex real-time PCR for intestinal parasites. Parasitology. 2017;144: 965–974. doi:10.1017/S0031182017000129

38. Dabney J, Knapp M, Glocke I, Gansauge M-T, Weihmann A, Nickel B, et al. Complete mitochondrial genome sequence of a Middle Pleistocene cave bear reconstructed from ultrashort DNA fragments. Proc Natl Acad Sci U S A. 2013;110: 15758–15763. doi:10.1073/pnas.1314445110

39. Côté NML, Daligault J, Pruvost M, Bennett EA, Gorgé O, Guimaraes S, et al. A new high-throughput approach to genotype ancient human gastrointestinal parasites. PLoS One. 2016;11: e0146230. doi:10.1371/journal.pone.0146230

40. Wood JR, Díaz FP, Latorre C, Wilmshurst JM, Burge OR, González F, et al. Ancient parasite DNA from late Quaternary Atacama Desert rodent middens. Quat Sci Rev. 2019;226: 106031. doi:10.1016/j.quascirev.2019.106031

41. Wiscovitch-Russo R, Rivera-Perez J, Narganes-Storde YM, García-Roldán E, Bunkley-Williams L, Cano R, et al. Pre-Columbian zoonotic enteric parasites: An insight into Puerto Rican indigenous culture diets and life styles. PLoS One. 2020;15: e0227810. doi:10.1371/journal.pone.0227810

42. Leles D, Araújo A, Ferreira LF, Vicente ACP, Iñiguez AM. Molecular paleoparasitological diagnosis of *Ascaris* sp. from coprolites: new scenery of ascariasis in pre-Columbian South America times. Mem Inst Oswaldo Cruz, Rio de Janeiro, Vol. 2008;103: 106–108.

43. Meyer M, Kircher M. Illumina sequencing library preparation for highly multiplexed target capture and sequencing. Cold Spring Harb Protoc. 2010;2010: db.prot5448. doi:10.1101/pdb.prot5448

44. Kircher M, Sawyer S, Meyer M. Double indexing overcomes inaccuracies in multiplex sequencing on the Illumina platform. Nucleic Acids Res. 2012;40: e3. doi:10.1093/nar/gkr771

45. Benson DA, Cavanaugh M, Clark K, Karsch-Mizrachi I, Lipman DJ, Ostell J, et al. GenBank. Nucleic Acids Res. 2013;41: D36-42. doi:10.1093/nar/gks1195

46. NCBI Resource Coordinators. Database resources of the National Center for Biotechnology Information. Nucleic Acids Res. 2016;44: D7-19. doi:10.1093/nar/gkv1290

47. Oh CS, Seo M, Chai JY, Lee SJ, Kim MJ, Park JB, et al. Amplification and sequencing of *Trichuris trichiura* ancient DNA extracted from archaeological sediments. J Archaeol Sci. {2010;37: 1269–1273. doi:10.1016/j.jas.2009.12.029

48. Jaeger LH, Iñiguez AM. Molecular paleoparasitological hybridization approach as effective tool for diagnosing human intestinal parasites from scarce archaeological remains. PLoS One. 2014;9: e105910. doi:10.1371/journal.pone.0105910

49. Hong JH, Oh CS, Seo M, Chai J-Y, Shin DH. Ancient *Ascaris* DNA Sequences of Cytochrome B, Cytochrome C Oxidase Subunit 1, NADH Dehydrogenase Subunit 1, and Internal Transcribed Spacer 1 Genes from Korean Joseon Mummy Feces. J Parasitol. 2017;103: 795–800. doi:10.1645/16-102

50. Sawyer S, Krause J, Guschanski K, Savolainen V, Pääbo S. Temporal patterns of nucleotide misincorporations and DNA fragmentation in ancient DNA. PLoS One. 2012;7: e34131. doi:10.1371/journal.pone.0034131

51. Kistler L, Ware R, Smith O, Collins M, Allaby RG. A new model for ancient DNA decay based on paleogenomic meta-analysis. Nucleic Acids Res. 2017;45: 6310–6320. doi:10.1093/nar/gkx361

52. Renaud G, Stenzel U, Kelso J. leeHom: adaptor trimming and merging for Illumina sequencing reads. Nucleic Acids Res. 2014;42: e141. doi:10.1093/nar/gku699

53. Ledger ML, Anastasiou E, Shillito L-M, Mackay H, Bull ID, Haddow SD, et al. Parasite infection at the early farming community of Çatalhöyük. Antiquity. 2019;93: 573–587. doi:10.15184/aqy.2019.61

54. Anastasiou E, Lorentz KO, Stein GJ, Mitchell PD. Prehistoric schistosomiasis parasite found in the Middle East. Lancet Infectious Diseases. 2014;14: 553–554. doi:10.1016/S1473-3099(14)70794-7

55. Mitchell PD, Anastasiou E, Whelton HL, Bull ID, Pearson MP, Shillito L-M. Intestinal parasites in the Neolithic population who built Stonehenge (Durrington Walls, 2500 BCE). Parasitology. 2022;149: 1027–1033. doi:10.1017/S0031182022000476

56. Ledger ML, Grimshaw E, Fairey M, Whelton HL, Bull ID, Ballantyne R, et al. Intestinal parasites at the Late Bronze Age settlement of Must Farm, in the fens of East Anglia, UK (9th century B.C.E.). Parasitology. 2019;146: 1583–1594. doi:10.1017/S0031182019001021

57. Yeh H-Y, Prag K, Clamer C, Humbert J-B, Mitchell PD. Human intestinal parasites from a Mamluk Period cesspool in the Christian quarter of Jerusalem: Potential indicators of long distance travel in the 15th century AD. International Journal of Paleopathology. 2015;9: 69–75. doi:10.1016/j.ijpp.2015.02.003

58. Deforce K, Ledger ML, Derreumaux M, Goffette Q, Henrotay D, Pigière F, et al. Diet, hygiene and health in Roman period northern Gaul: A multidisciplinary study of a latrine from an artisan household in the vicus Orolaunum (Arlon, southern Belgium, c. 250–280

CE). Journal of Archaeological Science: Reports. 2021;35: 102761. doi:10.1016/j.jasrep.2020.102761

59. Ledger ML, Rowan E, Marques FG, Sigmier JH, Šarkić N, Redžić S, et al. Intestinal parasitic infection in the Eastern Roman Empire during the Imperial Period and Late Antiquity. Am J Archaeol. 2020;124: 631–657. doi:10.3764/aja.124.4.0631

60. Williams FS, Arnold-Foster T, Yeh H-Y, Ledger ML, Baeten J, Poblome J, et al. Intestinal parasites from the 2nd-5th century AD latrine in the Roman Baths at Sagalassos (Turkey). Int J Paleopathol. 2017;19: 37–42. doi:10.1016/j.ijpp.2017.09.002

61. Ledger ML, Stock F, Schwaiger H, Knipping M, Brückner H, Ladstätter S, et al. Intestinal parasites from public and private latrines and the harbour canal in Roman Period Ephesus, Turkey (1st c. BCE to 6th c. CE). Journal of Archaeological Science: Reports. 2018;21: 289–297. doi:10.1016/j.jasrep.2018.07.013

62. Knorr DA, Smith WPW, Ledger ML, Peña-Chocarro L, Pérez-Jordà G, Clapés R, et al. Intestinal parasites in six Islamic medieval period latrines from 10th–11th century Córdoba (Spain) and 12th–13th century Mértola (Portugal). International Journal of Paleopathology. 2019;26: 75–83. doi:10.1016/j.ijpp.2019.06.004

63. Roche K, Pacciani E, Bianucci R, Le Bailly M. Assessing the parasitic burden in a Late Antique Florentine emergency burial site. Korean J Parasitol. 2019;57: 587–593. doi:10.3347/kjp.2019.57.6.587

64. Mitchell PD, Yeh H-Y, Appleby J, Buckley R. The intestinal parasites of King Richard III. Lancet. 2013;382: 888. doi:10.1016/S0140-6736(13)61757-2

65. Oh K-S, Kim G-T, Ahn K-S, Shin S-S. Effects of disinfectants on larval development of *Ascaris suum* eggs. Korean J Parasitol. 2016;54: 103–107. doi:10.3347/kjp.2016.54.1.103

66. Maurelli MP, Alves LC, Aggarwal CS, Cociancic P, Levecke B, Cools P, et al. *Ascaris lumbricoides* eggs or artefacts? A diagnostic conundrum. Parasitology. 2021;148: 1554–1559. doi:10.1017/S0031182021001256

67. Jónsson H, Ginolhac A, Schubert M, Johnson PLF, Orlando L. mapDamage2.0: fast approximate Bayesian estimates of ancient DNA damage parameters. Bioinformatics. 2013;29: 1682–1684. doi:10.1093/bioinformatics/btt193

68. Garcia LS. Diagnostic Medical Parasitology. Washington, DC: ASM Press; 2016.

69. Maicher C, Hoffmann A, Côté NML, Palomo Pérez A, Saña Segui M, Le Bailly M. Paleoparasitological investigations on the Neolithic lakeside settlement of La Draga (Lake Banyoles, Spain). Holocene. 2017;27: 1659–1668. doi:10.1177/0959683617702236

70. Liu G-H, Wu C-Y, Song H-Q, Wei S-J, Xu M-J, Lin R-Q, et al. Comparative analyses of the complete mitochondrial genomes of *Ascaris lumbricoides* and *Ascaris suum* from humans and pigs. Gene. 2012;492: 110–116. doi:10.1016/j.gene.2011.10.043

71. Søe MJ, Kapel CMO, Nejsum P. *Ascaris* from humans and pigs appear to be reproductively isolated species. PLoS Negl Trop Dis. 2016;10: e0004855. doi:10.1371/journal.pntd.0004855

72. Marciniak S, Klunk J, Devault A, Enk J, Poinar HN. Ancient human genomics: the methodology behind reconstructing evolutionary pathways. J Hum Evol. 2015;79: 21–34. doi:10.1016/j.jhevol.2014.11.003

73. Pedersen MW, Ginolhac A, Orlando L, Olsen J, Andersen K, Holm J, et al. A comparative study of ancient environmental DNA to pollen and macrofossils from lake sediments reveals taxonomic overlap and additional plant taxa. Quat Sci Rev. 2013;75: 161–168. doi:10.1016/j.quascirev.2013.06.006

74. Rawlence NJ, Lowe DJ, Wood JR, Young JM, Churchman GJ, Huang Y-T, et al. Using palaeoenvironmental DNA to reconstruct past environments: progress and prospects. J Quat Sci. 2014;29: 610–626. doi:10.1002/jqs.2740

75. Parducci L, Väliranta M, Salonen JS, Ronkainen T, Matetovici I, Fontana SL, et al. Proxy comparison in ancient peat sediments: pollen, macrofossil and plant DNA. Philos Trans R Soc Lond B Biol Sci. 2015;370: 20130382. doi:10.1098/rstb.2013.0382

76. Phuphisut O, Poodeepiyasawat A, Yoonuan T, Watthanakulpanich D, Chotsiri P, Reamtong O, et al. Transcriptome profiling of male and female *Ascaris lumbricoides* reproductive tissues. Parasit Vectors. 2022;15: 477. doi:10.1186/s13071-022-05602-2

77. Hansen EP, Tejedor AM, Thamsborg SM, Alstrup Hansen TV, Dahlerup JF, Nejsum P. Faecal egg counts and expulsion dynamics of the whipworm, *Trichuris trichiura* following self-infection. J Helminthol. 2016;90: 298–302. doi:10.1017/S0022149X1500019X

78. Cai P, Huang Q, Zhang X, Chen H. Adsorption of DNA on clay minerals and various colloidal particles from an Alfisol. Soil Biol Biochem. 2006;38: 471–476. doi:10.1016/j.soilbio.2005.05.019

79. Saeki K, Sakai M, Wada S-I. DNA adsorption on synthetic and natural allophanes. Appl Clay Sci. 2010;50: 493–497. doi:10.1016/j.clay.2010.09.015

80. Kanbar HJ, Olajos F, Englund G, Holmboe M. Geochemical identification of potential DNA-hotspots and DNA-infrared fingerprints in lake sediments. Appl Geochem. 2020;122: 104728. doi:10.1016/j.apgeochem.2020.104728

81. Kjær KH, Winther Pedersen M, De Sanctis B, De Cahsan B, Korneliussen TS, Michelsen CS, et al. A 2-million-year-old ecosystem in Greenland uncovered by environmental DNA. Nature. 2022;612: 283–291. doi:10.1038/s41586-022-05453-y

82. Mitchell PD. The Roman World. In: Mitchell PD, editor. Parasites in Past Civilizations and their Impact Upon Health. Cambridge: Cambridge University Press; 2023. pp. 53–64.

83. Ledger ML, Mitchell PD. Tracing zoonotic parasite infections throughout human evolution. Int J Osteoarchaeol. 2022;32: 553–564. doi:10.1002/oa.2786

84. Reinhard KJ. Parasitology as an interpretive tool in archaeology. Am Antiq. 1992;57: 231–245. doi:10.2307/280729

85. Else KJ, Keiser J, Holland CV, Grencis RK, Sattelle DB, Fujiwara RT, et al. Whipworm and roundworm infections. Nat Rev Dis Primers. 2020;6: 44. doi:10.1038/s41572-020-0171-3

Supporting Information

S1 Appendix. Appendix including materials, methods, and parasite literature review.

S2 Appendix. Appendix including parasite bait accession numbers and bait description.

S3 Appendix. Detailed results.

Figure 1

Click here to access/download;Figure;Fig1_SiteLocation.tiff

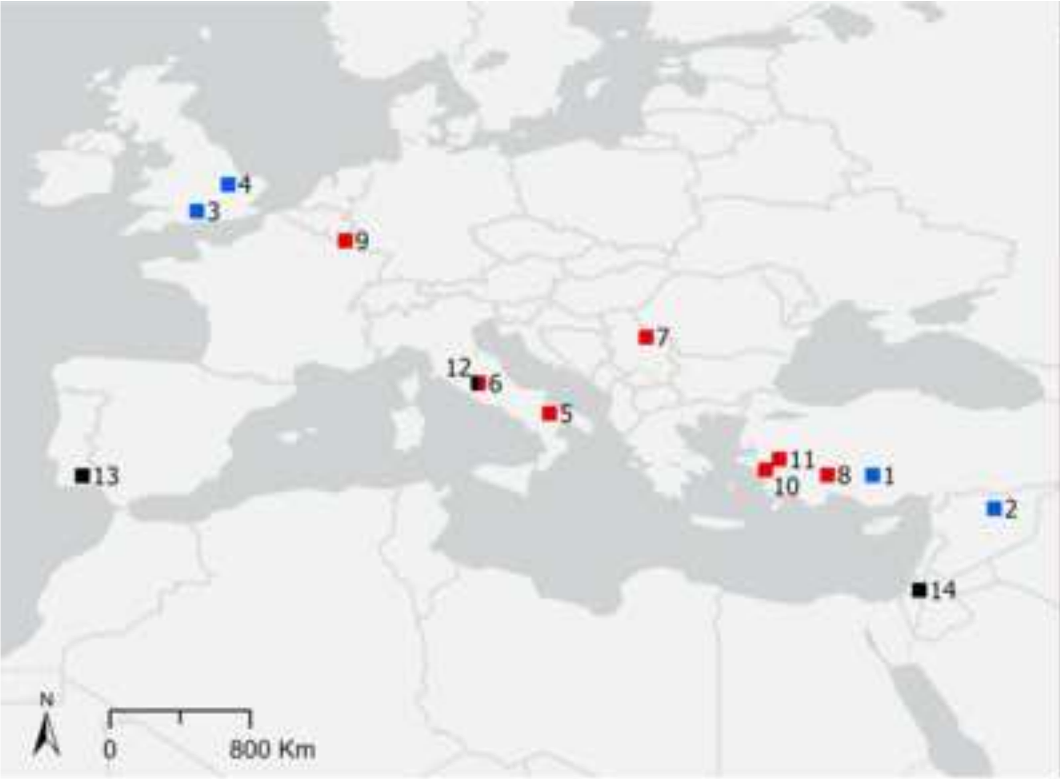

Time Period	Map Label	Site Name	Date	Sample Type
Pre-Roman	1	Çatalhöyük	6410–6150 BCE	Coprolite
	2	Tell Zeidan	4550–4050 BCE	Pelvic soil
	3	Durrington Walls	c. 2500 BCE	Coprolite
	4	Must Farm	865–840 cal BCE	Coprolite, Occupation sediment
Roman	5	Vagnari	1st–4th c. CE	Drain
	6	Vacone	2nd c. BCE– 3rd c. CE	Drain, Latrine
	7	Viminacium	2nd–3rd c. CE	Coprolite
	8	Sagalassos	2nd–5th c. CE	Latrine
	9	Arlon	250–280 CE	Latrine
	10	Ephesus	3rd–6th c. CE	Latrine
	11	Sardis	6th–7th c. CE	Drain
Post-Roman	12	Vacone	7th–8th c. CE	Pelvic soil
	13	Mértola	12th–13th c. CE	Latrine
	14	Jerusalem	15th–16th c. CE	Latrine, Coprolite

Figure 2

Click here to access/download;Figure;Fig2_ResultsFigure.png

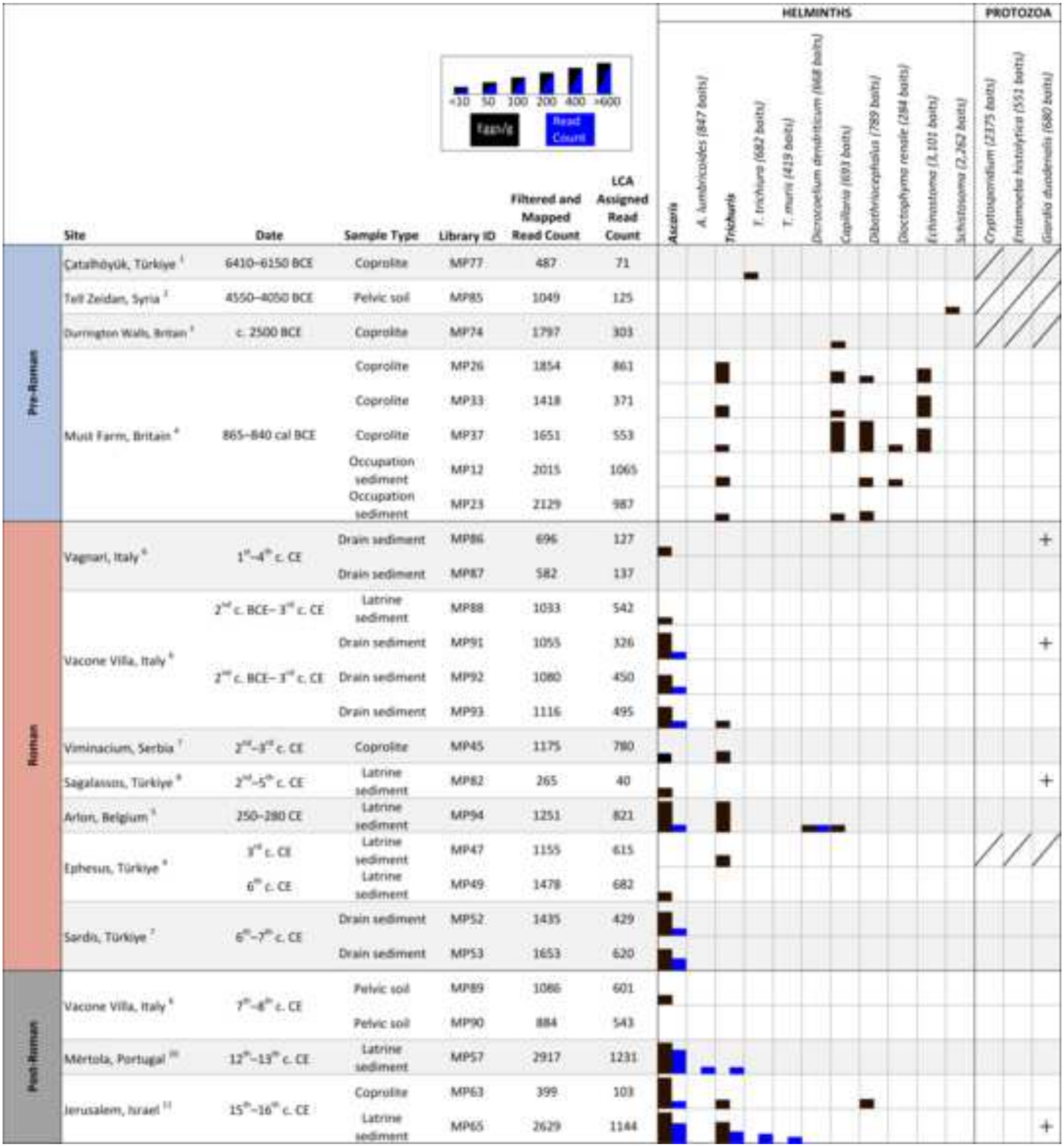

 Click here to access/download;Figure;Fig3_HeatMap.tiff

Site	Sample Type	DNA Library ID	*Ascaris* (eggs per gram)	% LCA Assigned (Ascaridoidea or Lower)	Read Count (Ascaridoidea or Lower)
Jerusalem	Cesspit sediment	MP65	1580	21.8%	249
Mértola	Latrine	MP57	1110	24.1%	297
Arlon	Latrine	MP94	990	0.5%	4
Jerusalem	Coprolite	MP63	595	5.8%	6
Vacone	Drain sediment	MP91	405	1.8%	6
Sardis	Drain sediment	MP52	305	1.2%	5
Sardis	Drain sediment	MP53	205	9.7%	60
Vacone	Drain sediment	MP93	185	1.4%	7
Vacone	Drain sediment	MP92	140	1.6%	7
Vacone	Pelvic soil	MP89	30	0.0%	0
Vagnari	Drain sediment	MP86	25	2.4%	3
Ephesus	Latrine	MP49	25	0.0%	0
Sagalassos	Latrine	MP82	20	0.0%	0
Vacone	Latrine	MP88	5	0.0%	0

Figure 4

Click here to access/download;Figure;Fig4_mapDamage.tiff

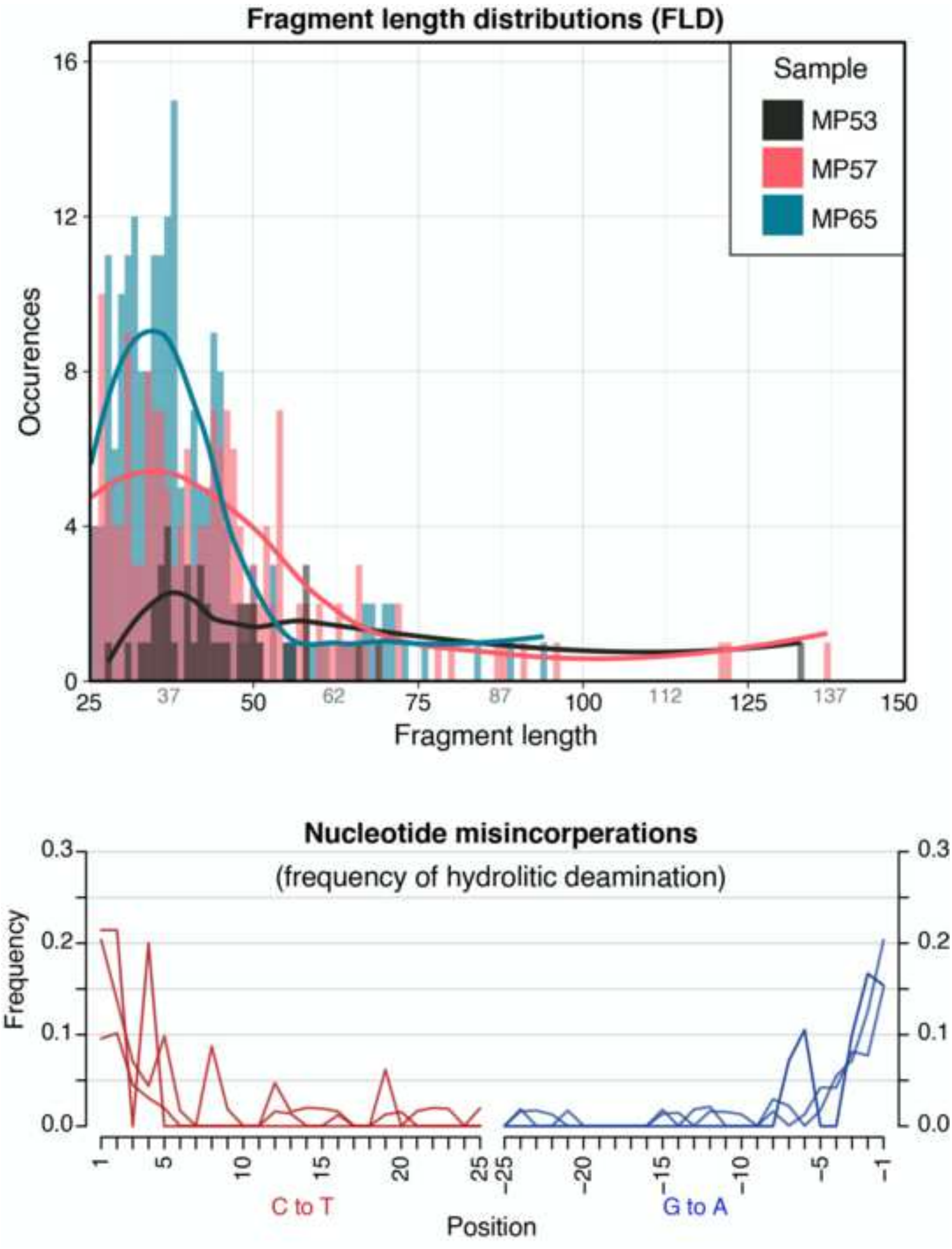

Figure 5

Click here to access/download;Figure;Fig5_Ascaris.png

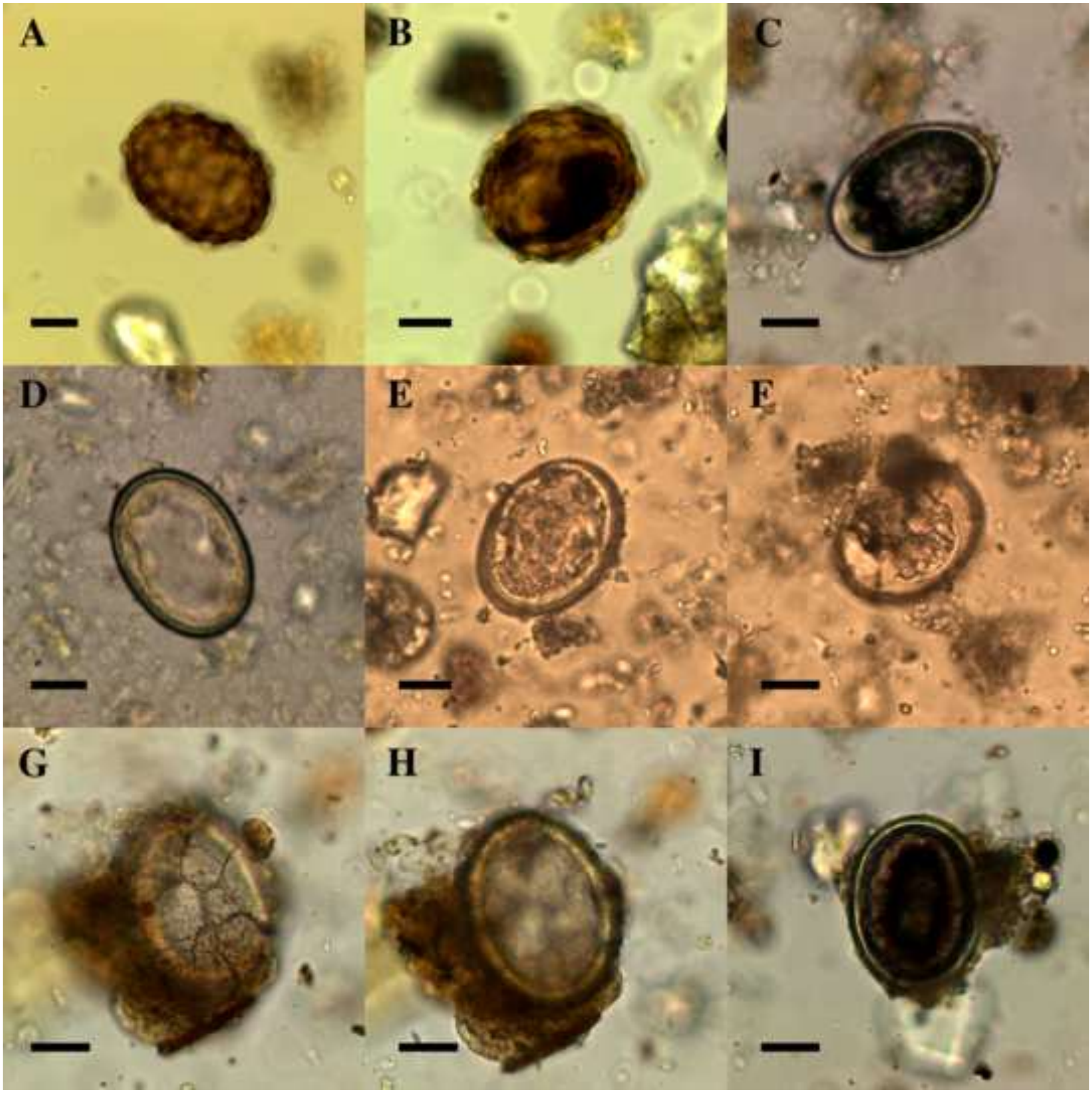

Figure 6

Click here to access/download;Figure;Fig6_TemporalChanges.png

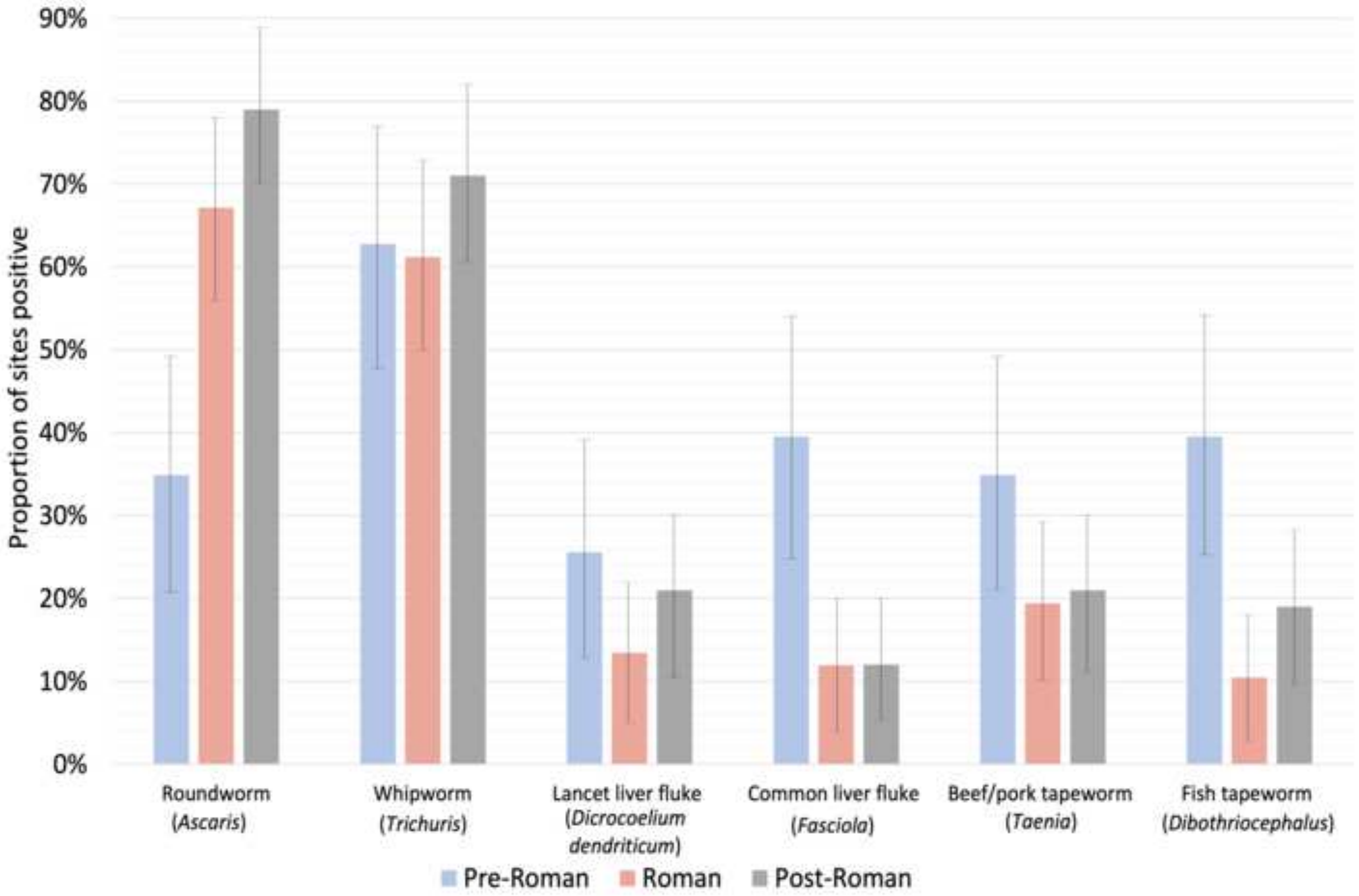

Click here to access/download
Supporting Information
S1_Appendix_Materials_Methods_TemporalData_RefsFormatted.docx

Click here to access/download
Supporting Information
S2_Appendix_ParasiteBaits.xlsx

Click here to access/download
Supporting Information
S3_Appendix_Results.xlsx

Click here to access/download
Revised Article with Changes Highlighted
Manuscript_ChangesTracked.docx
