# Supplementary material for: Sedimentary ancient DNA as part of a multimethod paleoparasitology approach reveals temporal trends in human parasitic burden in the Roman period
Source: PLoS Negl Trop Dis. 2025 Jun 10;19(6):e0013135. doi: 10.1371/journal.pntd.0013135 (PMC12151383; doi:10.1371/journal.pntd.0013135)
Supplement: Attachment — Submitted filename: Response to Reviewers2.docx [file pntd.0013135.s007.docx]

**Response to Reviewers**

PNTD-D-24-01526

Sedimentary ancient DNA as part of a multimethod paleoparasitology approach reveals temporal trends in human parasitic burden in the Roman period

PLOS Neglected Tropical Diseases

**Reviewers' Comments:**

Reviewer's Responses to Questions

**Key Review Criteria Required for Acceptance?**

As you describe the new analyses required for acceptance, please consider the following:

**Methods:**
-Are the objectives of the study clearly articulated with a clear testable hypothesis stated?
-Is the study design appropriate to address the stated objectives?
-Is the population clearly described and appropriate for the hypothesis being tested?
-Is the sample size sufficient to ensure adequate power to address the hypothesis being tested?
-Were correct statistical analysis used to support conclusions?
-Are there concerns about ethical or regulatory requirements being met?

Reviewer #2: I

Reviewer #3: The article was modified according to the suggestions made.

**Results:**
-Does the analysis presented match the analysis plan?
-Are the results clearly and completely presented?
-Are the figures (Tables, Images) of sufficient quality for clarity?

Reviewer #2: (No Response)

Reviewer #3: The article was modified according to the suggestions made.
Some taxonomic names require correction before publication. Please review the revised PDF.

Response: Changes have been made as requested.

**Conclusions:**
-Are the conclusions supported by the data presented?
-Are the limitations of analysis clearly described?
-Do the authors discuss how these data can be helpful to advance our understanding of the topic under study?
-Is public health relevance addressed?

Reviewer #2: (No Response)

Reviewer #3: The article was modified according to the suggestions made.
Some taxonomic names require correction before publication. Please review the revised PDF.

Response: Changes have been made as requested.

**Editorial and Data Presentation Modifications?**
Use this section for editorial suggestions as well as relatively minor modifications of existing data that would enhance clarity. If the only modifications needed are minor and/or editorial, you may wish to recommend “Minor Revision” or “Accept”.

Reviewer #2: (No Response)

Reviewer #3: A “Minor Revision” is suggested to address the incorrect taxonomic writing that remains.

Response: Changes have been made as requested.

**Summary and General Comments:**
Use this section to provide overall comments, discuss strengths/weaknesses of the study, novelty, significance, general execution and scholarship. You may also include additional comments for the author, including concerns about dual publication, research ethics, or publication ethics. If requesting major revision, please articulate the new experiments that are needed.

Reviewer #2: I will stick to all of my previous comments. The article is very well written and easy to follow. Thank you to the authors for responding to my comments. Nevertheless, I have searched for the public data again and have still not found it in both NCBI and ENA.

Response: The data has been submitted but is embargoed until publication.

Reviewer #3: The article was modified according to the suggestions made.
